# Supplementary material for: Discovery of a terpene synthase synthesizing a nearly non-flexible eunicellane reveals the basis of flexibility
Source: Nat Commun. 2024 Jul 15;15:5940. doi: 10.1038/s41467-024-50209-z (PMC11250809; doi:10.1038/s41467-024-50209-z)
Supplement: Supplementary file 1 — Supplementary Information [file 41467_2024_50209_MOESM1_ESM.pdf]

Supplementary Information

Discovery of a terpene synthase synthesizing a nearly non-flexible eunicellane reveals the basis of flexibility

Jinfeng Li[1,2,8], Bao Chen[1,8], Zunyun Fu[3,8], Jingjing Mao[4,5,8], Lijun Liu[1], Xiaochen Chen[1], Mingyue Zheng[3], Chang-Yun Wang[2,6*], Chengyuan Wang[4*], Yue-Wei Guo[1,7*] & Baofu Xu[1,3*]

[1]Shandong Laboratory of Yantai Drug Discovery, Bohai Rim Advanced Research Institute for Drug Discovery, Yantai, Shandong 264117, China. [2]Key Laboratory of Marine Drugs, The Ministry of Education of China, Institute of Evolution & Marine Biodiversity, School of Medicine and Pharmacy, Ocean University of China, Qingdao, 266003, China. [3]Shanghai Institute of Materia Medica, Chinese Academy of Sciences, 555 Zu Chong Zhi Road, Zhangjiang Hi-Tech Park, Shanghai, 201203, China. [4]CAS Key laboratory of Molecular Virology and Immunology, Shanghai Institute of Immunity and Infection, Shanghai, 200031, China. [5]Department of Pathogen Biology, School of Medicine and Holistic Integrative Medicine, Nanjing University of Chinese Medicine, Nanjing, 210023, China. [6]Laboratory for Marine Drugs and Bioproducts, Qingdao National Laboratory for Marine Science and Technology, Qingdao, 266237, China. [7]School of Medicine, Shanghai University, Shanghai 200444, China. [8]These authors contributed equally: Jinfeng Li, Bao Chen, Zunyun Fu, Jingjing Mao. [*]e-mail: bfxu@simm.ac.cn; ywguo@simm.ac.cn; cywang@ips.ac.cn; changyun@ouc.edu.cn

Table of contents

Supplementary Method 1
Supplementary Figure .... 错误!未定义书签。
Supplementary Fig. 1. Eunicellane diterpenoids. 12
Supplementary Fig. 2. Biosynthetic hypothesis for microeunicellols A and B. 13
Supplementary Fig. 3. Patent information of *Micromonospora* sp. HM134. 13
Supplementary Fig. 4. Diterpene overproduction system in *E. coli*. 14
Supplementary Fig. 5. HPLC analyses (210 nm) of minor products. 15
Supplementary Fig. 6. GC-MS spectra of fraction A from minor products. 16
Supplementary Fig. 7. GC-MS spectra of fraction B from minor products. 17
Supplementary Fig. 8. GC-MS spectra of fraction C from minor products. 18
Supplementary Fig. 9. GC-MS spectra of fraction D from minor products. 19
Supplementary Fig. 10. Purity report of compounds **1** and **6**–**8**. 20
Supplementary Fig. 11. ^{1}H NMR spectra (600 MHz) of compounds **1** and **6**–**8**. 20
Supplementary Fig. 12. Synthesis of GLPP, GGPP, 2*Z*-GGPP and 1,1-$^{2}H_2$-GGPP. 21
Supplementary Fig. 13. In vitro assays of Bnd4 and AlbS with GGPP and 2*Z*-GGPP. 22
Supplementary Fig. 14. DFT calculation of the hypothesized 1*S*,10*R*-**1**, 1*R*,10*S*-**1**, 2*E*-**1**, and 1*R*,10*R*-**1**. 23
Supplementary Fig. 15. Metadynamics sampling and free energy calculation of **1** (the MicA product). 23
Supplementary Fig. 16. The variable temperature (VT) NMR of compounds **6** and **7**. 24
Supplementary Fig. 17. The variable temperature (VT) NMR of compounds **8** and **1**. 25
Supplementary Fig. 18. Dihedral angles of conformations of **1**, 1*R*,10*R*-**1**, 1*R*,10*S*-**1**, 1*S*,10*R*-**1** and 2*E*-**1**. 26
Supplementary Fig. 19. Dihedral angles of conformations of **6**–**8**. 27
Supplementary Fig. 20. Mechanism on the terpene synthases (TSs) involved in eunicellane biosynthesis. 27
Supplementary Fig. 21. Catalytic route validation by in vitro reactions with hypothesized intermediates and deuterated substrate. 28
Supplementary Fig. 22. Proposed catalytic pathways for forming the 6,10-bicyclic eunicellane skeleton of **1**. 29
Supplementary Fig. 23. The GC-MS spectra of 1 and ($^{2}H_2$)-**1**. 30
Supplementary Fig. 24. In situ biosynthesis of 10F-GGPP. 31
Supplementary Fig. 25. Computed energetics for pathway 2. 32
Supplementary Fig. 26. Intermediates and transition state structures of pathway 2. 33
Supplementary Fig. 27. The IRC path in pathway 1. 34
Supplementary Fig. 28. The IRC path in pathway 2A. 35
Supplementary Fig. 29. The IRC path in pathway 2B. 36
Supplementary Fig. 30. The IRC path in pathway 2C. 37
Supplementary Fig. 31. Structural comparisons of computed MicA models. 38
Supplementary Fig. 32. Protein model quality assessment of predicted MicA models. 39
Supplementary Fig. 33. Structural alignments of MicA and other related proteins. 40

Supplementary Fig. 34. Structural comparisons of MicA docking model and CotB2 crystal structure........41
Supplementary Fig. 35. A proposed mechanistic pathway explained the cyclization of GGPP to form compounds **3**–**5** among two MicA. 41
Supplementary Fig. 36. Overview of V220 and L221 in MicA and their corresponding residue L285 in AsR6. 42
Supplementary Fig. 37. HPLC analyses (210 nm) of the mutants MicA $^{(V220M)}$ and MicA $^{(L221M)}$ in comparison with native MicA........42
Supplementary Fig. 38. SDS-PAGE of purified MicA and mutants. 43
Original spectra for compound **1** in $CDCl_3$........44
Original spectra for compound **2**........47
Original spectra for compound **1a**........50
Original spectra for compound **1b**. 53
Original spectra for compound **1c**........57
Original spectra for compound **1d**. 60
Original spectra for ($^2H_2$)-**1**........64
Original spectra for compound **3**........66
Original spectra for compound **4**........70
Original spectra for compound **5**........73
Original spectra for compound **1** in C_6D_6. 77
Original spectra for compound **6**........78
Original spectra for compound **7**........80
Original spectra for compound **8**........81
Original spectra for compound **9**........83
Original spectra for compound **10**........85
Original spectra for compound **11**........87
Original spectra for compound **12**........90
The variable temperature (VT) NMR of compound **1**. 92
VT NMR of compound **6**. 95
VT NMR of compound **7**. 98
VT NMR of compound **8**. 101
Supplementary Table........104
Supplementary Table 1. Strains used in this study. 104
Supplementary Table 2. Plasmids used in this study........104
Supplementary Table 3. Primer sequences used in this study. 105
Supplementary Table 4. DNA and protein sequence of MicA. 109
Supplementary Table 5. ^1H (600 MHz) and ^{13}C NMR (150 MHz) data of **1** and ($^2H_2$)-**1** in $CDCl_3$........110
Supplementary Table 6. ^1H (600 MHz) and ^{13}C NMR (150 MHz) data of 2 in$CDCl_3$. 111
Supplementary Table 7. Crystal cultivation conditions of **1**, **1a**–**1d**. 111
Supplementary Table 8. ^1H (600 MHz) and ^{13}C NMR (150 MHz) data of **1a** in $CDCl_3$. 112
Supplementary Table 9. ^1H (600 MHz) and ^{13}C NMR (150 MHz) data of **1b** in $CDCl_3$. 112
Supplementary Table 10. ^1H (600 MHz) and ^{13}C NMR (150 MHz) data of **1c** in $CDCl_3$......113
Supplementary Table 11. ^1H (600 MHz) and ^{13}C NMR (150 MHz) data of **1d** in $CDCl_3$. ...113

Supplementary Table 12. ^{1}H (600 MHz) and ^{13}C NMR (150 MHz) data of **3** in C_6D_6.........114
Supplementary Table 13. ^{1}H (600 MHz) and ^{13}C NMR (150 MHz) data of **4** in $CDCl_3$. 114
Supplementary Table 14. ^{1}H (600 MHz) and ^{13}C NMR (150 MHz) data of **5** in $CDCl_3$. 115
Supplementary Table 15. ^{1}H (600 MHz) and ^{13}C NMR (150 MHz) data of **9** in C_6D_6.........115
Supplementary Table 16. ^{1}H (600 MHz) and ^{13}C NMR (150 MHz) data of **10** in C_6D_6.......116
Supplementary Table 17. ^{1}H (600 MHz) and ^{13}C NMR (150 MHz) data of **11** in C_6D_6.......116
Supplementary Table 18. ^{1}H (600 MHz) and ^{13}C NMR (150 MHz) data of **12** in C_6D_6.......117
Supplementary Table 19. Energies, enthalpies, and free energies of the structures.............118
Supplementary References..120

Supplementary Method

Compound structure elucidation

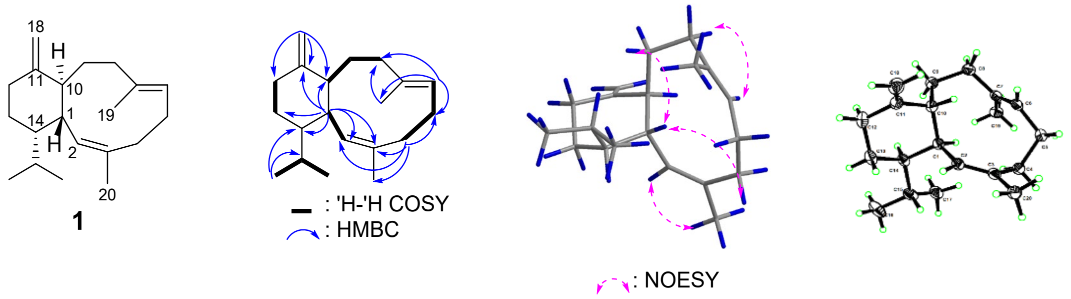

Compound **1** was obtained as a colorless crystal. Its molecular formula was deduced to be $C_{20}H_{32}$ by GC-MS exhibited an M^+ peak at *m/z* of 272.2 (Main text Fig. 2c), implying five degrees of unsaturation. Its ^{1}H NMR spectrum (Supplementary Fig. 39) showed two geminal methyls at δ_H 0.83 (6H, dd, J = 8.1, 6.6 Hz, H_3-16, 17), two vinyl methyls at δ_H 1.71 (6H, d, J = 1.6 Hz, H_3-19, 20), one exocyclic double bond at δ_H 4.56 (2H, d, J = 1.8 Hz, H_2-18), and two olefinic protons at δ_H 5.23 (1H, d, J = 11.4 Hz, H-2), δ_H 5.42 (1H, t, J = 7.9 Hz, H-6), which were attributed to 2 trisubstituted double bonds. The ^{13}C NMR and HSQC spectra (Supplementary Fig.s 40 and 41) allowed the identification of 20 carbon resonances, including six olefinic carbons (δ_C 153.5, 134.1, 132.2, 127.6, 124.8, 107.1), four methyl carbons (δ_C 24.5, 22.2, 21.2, 16.3), six methylene carbons (δ_C 41.2, 33.9, 31.5, 31.0, 26.4, 26.2) and four methyne carbons (δ_C 50.1, 42.9, 42.1, 30.6). Therefore, the five degrees of unsaturation were attributed to two trisubstituted double bonds, one exocyclic double bond, and 6,10-bicyclic ring according to the 1D NMR data. Further, the significant HMBC correlations (Supplementary Fig. 42) from H-1 (δ_H 2.12) to C-2 (δ_C 127.6)/C-3 (δ_C 132.2)/C-10 (δ_C 50.1)/C-11 (δ_C 153.5)/C-13 (δ_C 26.4)/C-14 (δ_C 42.9), H-4a (δ_H 2.60) to C-2/C-3/C-5 (δ_C 26.2)/C-20 (δ_C 24.5), H-6 (δ_H 5.42) to C-5/C-7 (δ_C 134.1)/C-19 (δ_C 16.3), H_3-16 (δ_H 0.83) to C-14 (δ_C 42.9)/C-15 (δ_C 30.6), H_3-17 (δ_H 0.83) to C-10/C-11/C-12 (δ_C 33.9), H-19 (δ_H 1.71) to C-7, and the ^{1}H-^{1}H COSY spectrum (Supplementary Fig. 43) from H-2 (δ_H 5.23) to H-1, H-5a (δ_H 2.35) to H-4, H-5 to H-6, H-13b (δ_H 1.08) to H-12a (δ_H 2.24), H-15 (δ_H 1.27) to H_3-16/H_3-17, constructed the eunicellane skeleton as depicted above. The planar structure was further confirmed by X-ray diffraction analysis using Cu Kα (λ=1.5417 Å) [Flack parameters: 0.60(4)], and meanwhile assigned its relative configuration. Epoxides **1a**–**1d** were synthesized by epoxidation with metachloroperoxybenzoic acid (*m*CPBA). X-ray diffraction analysis using Cu Kα (λ=1.5417 Å) unambiguously determines the absolute configurations of **1b**–**1d** (Main text Fig. 2f). Therefore, the absolute configurations of **1** can be determined, 1*S*, 2*Z*, 6*E* 10*S*, 14*S*, which is consistent with **1b**–**1d**. The structure of compound **1** was drawn, as shown above, named microeunicellene.

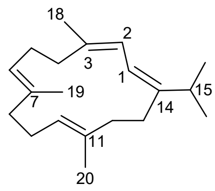

2

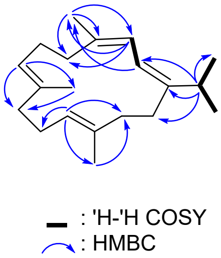

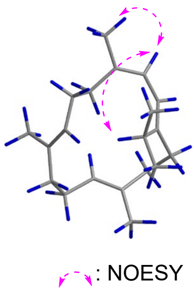

Compound **2** was obtained as colorless oil. Its ^{1}H NMR spectrum (Supplementary Fig. 45) showed two geminal methyls at δ_H 1.01 (3H, d, J = 6.9 Hz, H$_3$-16) and δ_H 1.02 (3H, d, J = 6.9 Hz, H$_3$-17), 3 vinyl methyls at 1.80 (3H, s, H$_3$-18), 1.60 (3H, d, J = 1.0 Hz, H$_3$-19) and 1.59 (3H, s, H$_3$-20), typical signals for a cembrane nucleus. The observed HMBC ^{1}H-^{1}H COSY and NOESY correlations are shown above (Supplementary Fig.s 48–50). The chemical shift of the C-18, C-19, C-20 methyl groups (δ_C 24.0, 15.6, 16.5)[1] as well as the NOESY cross-peaks of H-2/H-15(δ_H 3.07), H-3/H$_3$-16, H-7/H$_3$-18 established the Z geometries of the $\Delta^{1,14}$, $\Delta^{2,3}$, and the E geometries of the $\Delta^{6,7}$, $\Delta^{10,11}$.

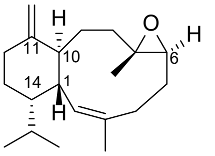

1a

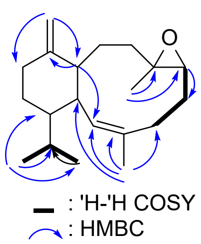

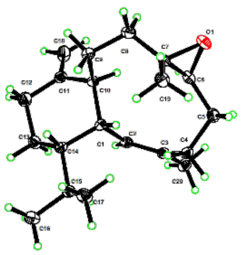

Compound **1a** was obtained as a colorless crystal. The ^{13}C NMR and HSQC spectra of **1a** (Supplementary Fig.s 52 and 53) revealed the presence of twenty carbon signals, including four methyl carbons, six sp^3 methylene carbons, one sp^2 methylene carbon, five sp^3 methine carbons, one sp^2 methine carbon, and three quaternary carbons. The diagnostic ^{1}H and ^{13}C NMR resonances (Supplementary Fig.s 51 and 52), as well as the coupling constants of the connected protons, suggested the presence of one trisubstituted double bond [δ_H 5.31 (1H, d, J=11.8 Hz)/δ_C 128.0 (CH), δ_C 132.1 (C)], one exocyclic double bond [δ_H 4.57 (1H, d, J=1.9 Hz), 4.53 (1H, d, J=1.9 Hz)/δ_C 107.9 (CH$_2$), δ_C 152.2 (C)] and one epoxy ring [δ_H 3.07 (1H, dd, J=9.5, 5.4 Hz)/δ_C 61.9 (CH), δ_C 59.6 (C)]. Careful comparison of the ^{1}H and ^{13}C NMR data of **1a** and **1** (Supplementary Fig.s 39, 40, 51, and 52), found that **1a** was similar to **1**, except that epoxidation of $\Delta^{6,7}$ in **1a** replaced $\Delta^{6,7}$ in **1**. Due to the epoxidation, the ^{13}C NMR chemical shift of C-4 (δ_C 28.7 in **1a**, δ_C 31.5 in **1**) were apparently upfield shifted ($\Delta\delta$ -2.8), while C-5 (δ_C 28.2 in **1a**, δ_C 26.2 in **1**), C-9 (δ_C 32.3 in **1a**, δ_C 31.0 in **1**) were apparently downfield shifted ($\Delta\delta$ +2, +1.3). The above observations were further supported by the 2D NMR spectra as well as X-ray diffraction analysis using Cu Kα (λ=1.5417 Å) [Flack parameters: 0.00(7)] and meanwhile assigned its relative configuration (Main text Fig. 2f and Supplementary Fig.s 53–56). The absolute configurations of it (1S, 2Z, 6S, 7S, 10S, 14S) were the same as **1b**–**1d**. The structure of compound **1a** was drawn as shown above, named (6S,7S)-epoxy-microeunicellene.

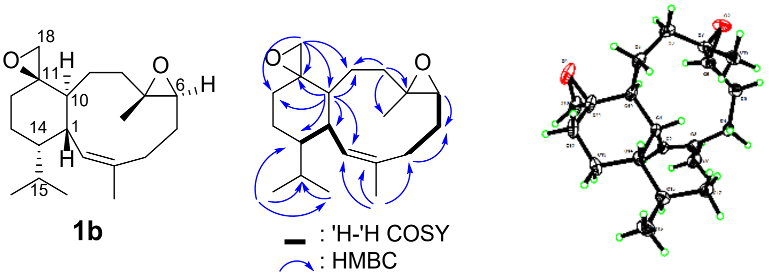

Compound **1b** was obtained as a colorless crystal. Its ^{13}C NMR and HSQC spectra (Supplementary Fig.s 58 and 59) implied the presence of two olefinic carbons (δ_C 127.0, 133.3), four methyl carbons (δ_C 16.3, 21.0, 22.5, 24.0), one oxygenated methylene carbon (δ_C 57.5), one oxygenated methine carbon (δ_C 61.7) and two oxygenated quaternary carbons (δ_C 59.5, 60.7). Careful comparison of the ^{1}H and ^{13}C NMR data of **1a** and **1b** (Supplementary Fig.s 51, 52, 58, and 59), found that **1b** was similar to **1a**, except that epoxidation of $\Delta^{11,18}$ in **1b** replaced $\Delta^{11,18}$ in **1a**. Due to the epoxidation, the ^{13}C NMR chemical shifts of C-9 (δ_C 27.0 in **1b**, δ_C 32.3 in **1a**), C-10 (δ_C 48.6 in **1b**, δ_C 50.5 in **1a**), C-12 (δ_C 31.3 in **1b**, δ_C 33.2 in **1a**), C-13 (δ_C 24.2 in **1b**, δ_C 26.1 in **1a**) were apparently upfield shifted ($\Delta\delta$ -5.3, -1.9, -1.9, -1.9). The above observations were further supported by the 2D NMR spectra as well as X-ray diffraction analysis using Cu Kα (λ=1.5417 Å) [Flack parameters: 0.09(11)] and meanwhile assigned its absolute configuration (1*S*, 2*Z*, 6*S*, 7*S*, 10*S*, 11*R*, 14*S*) (Main text Fig. 2f and Supplementary Fig.s 60–63). The structure of compound **1b** was drawn as shown above, named (6*S*,7*S*,11*R*)-*bis*-epoxy-microeunicellene.

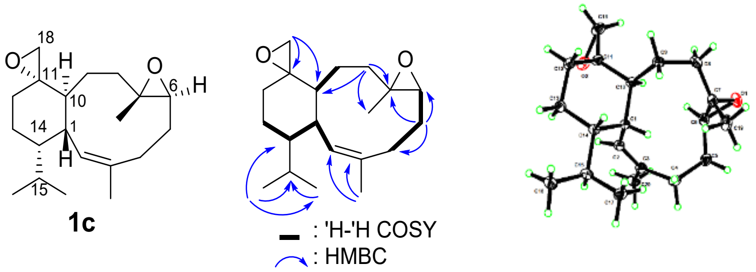

Compound **1c** was obtained as a colorless crystal. Detailed comparison of the 1D and 2D NMR data of **1c** with **1b** indicated that both molecules share the same planar structure. The main differences between them were at C-1 (δ_C 41.5 in **1c**, δ_C 42.5 in **1b**), C-9 (δ_C 29.0 in **1c**, δ_C 27.0 in **1b**), C-10 (δ_C 49.2 in **1c**, δ_C 48.6 in **1b**), C-13 (δ_C 22.0 in **1c**, δ_C 24.2 in **1b**), and C-18 (δ_C 51.8 in **1c**, δ_C 57.5 in **1b**), and the chemical shifts of them were obviously shifted in **1c**, which was deduced to be an epimer of **1b** at the C-11 position. The above observations were further supported by the 2D NMR spectra as well as X-ray diffraction analysis using Cu Kα (λ=1.5417 Å) [Flack parameters: -0.10(15)] and meanwhile assigned its absolute configuration (1*S*, 6*S*, 7*S*, 10*S*, 11*S*, 14*S*) (Main text Fig. 2f). The structure of compound **1c** was drawn as shown above, named (6*S*,7*S*,11*S*)-*bis*-epoxy-microeunicellene.

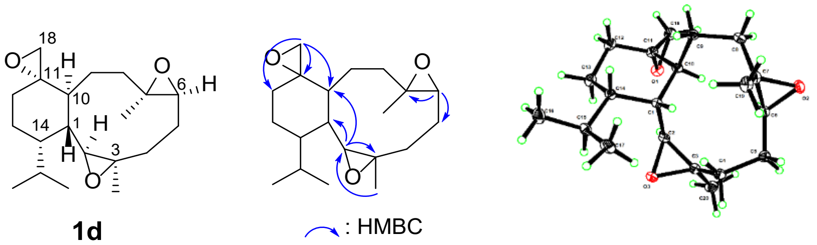

1d

Compound **1d** was obtained as a colorless crystal. Its ^{13}C NMR and HSQC spectra (Supplementary Fig.s 72 and 73) implied the presence of four methyl carbons (δ_C 15.9, 21.8, 21.9, 22.5), one oxygenated methylene carbon (δ_C 51.2), two oxygenated methine carbons (δ_C 64.1, 61.2) and three oxygenated quaternary carbons (δ_C 58.2, 59.2, 61.0). There were no signals of olefinic carbons, which was deduced to be further epoxidation. The hypothesized was further supported by the 2D NMR spectra as well as X-ray diffraction analysis using Cu Kα (λ=1.5417 Å) [Flack parameters: -0.02(10)] and meanwhile assigned its absolute configuration (1*R*, 2*S*, 3*R*, 6*S*, 7*S*, 10*S*, 11*S*, 14*S*) (Main text Fig. 2f and Supplementary Fig.s 74–77). The structure of compound **1d** was drawn as shown above, named (2*S*,3*R*,6*S*,7*S*,11*S*)-*tri*-epoxy-microeunicellene.

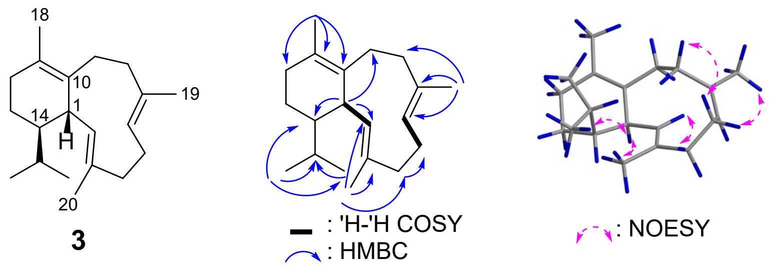

3

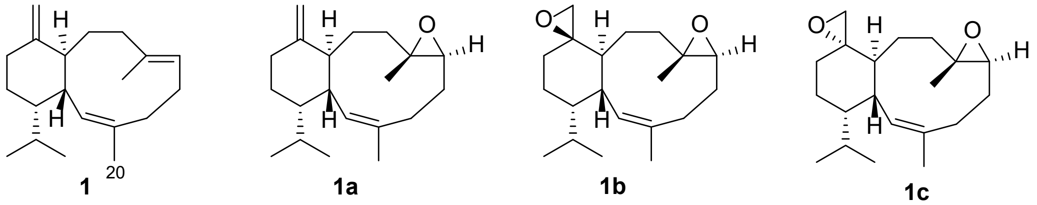

1 C-20 (δ_C 24.5)　**1a** C-20 (δ_C 23.9)　**1b** C-20 (δ_C 24.0)　**1c** C-20 (δ_C 24.0)

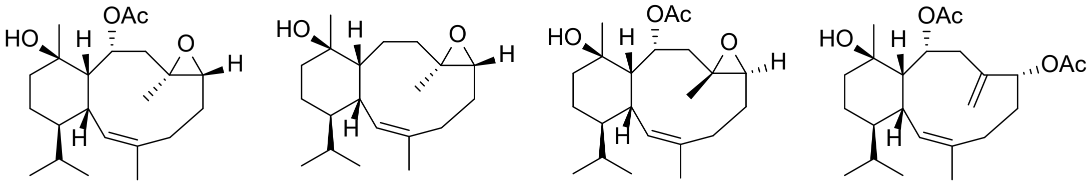

solenopodin A C-20 (δ_C 24.7)　**solenopodin C** C-20 (δ_C 24.9)　**solenopodin B** C-20 (δ_C 23.1)　**solenopodin D** C-20 (δ_C 22.8)

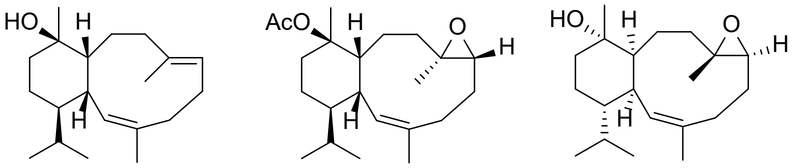

klysimplexin R C-20 (δ_C 25.0)　**klysimplexin Q** C-20 (δ_C 25.8)　**(-)-solenopodin C** C-20 (δ_C 24.9)

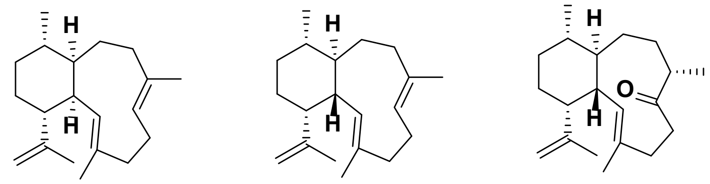

6 C-20 (δ_C 15.6)　**7** C-20 (δ_C 16.3)　**albireticulone A** C-20 (δ_C 18.8)

Compound **3** was obtained as colorless oil. Its 1D NMR spectra (Supplementary Fig.s 84 and 85) showed two geminal methyls at δ_H 0.99 (3H, d, J = 6.6 Hz, H$_3$-16) and 0.92 (3H, d, J = 6.6 Hz, H$_3$-17), three vinyl methyls at 1.62 (3H, s, H$_3$-18) and 1.54 (6H, s, H$_3$-19, 20), included two trisubstituted double bonds [δ_H 4.76 (d, 10.1), δ_C 135.7 (CH), δ_C 129.1 (C); δ_H 4.80 (d, 10.1), δ_C 127.2 (CH), δ_C 136.7 (C)], one tetrasubstituted double bond [δ_C 134.6 (C), 128.4 (C)]. The observed HMBC correlations and ^{1}H-^{1}H COSY correlations were shown above (Supplementary Fig.s 87 and 88). Upon careful analysis, it was observed that the vinyl methyls at C-20 [δ_C 24.5 in **1**, δ_C 23.9 in **1a**, δ_C 24.0 in **1b**, δ_C 24.0 in **1c**, δ_C 24.7 in solenopodin A, δ_C 23.1 in solenopodin B, δ_C 24.9 in solenopodin C, δ_C 22.8 in solenopodin D, δ_C 25.0 in klysimplexin R, δ_C 25.8 in klysimplexin Q, δ_C

24.9 in (-)-solenopodin C] were all greater than 20 (*ref.* 2–4). Conversely, the vinyl methyls at C-20 (δ_C 15.6 in **6**, δ_C 16.3 in **7**, δ_C 18.8 in albireticulone A) were less than 20 (*ref.* 5–8). Based on this inference, it could be concluded that compound **3** has an *E* configuration for its $\Delta^{2,3}$, since the vinyl methyl at C-20 (δ_C 16.2) and further supported by the NOE correlations of H-1 (δ_H 2.79)/H_3-20, H-2 /H-4b (δ_H 1.96). The double bonds, $\Delta^{6,7}$ and $\Delta^{10,11}$ were also in favor of *E* configurations, supported by the NOE correlations of H-6 /H-8b (δ_H 2.14), H_3-19 /H-5a (δ_H 2.26). The NOE correlations of H-1/H_3-16/H_3-17 suggested that the H-1 and isopropyl at C-14 have the same orientation (Supplementary Fig. 89).

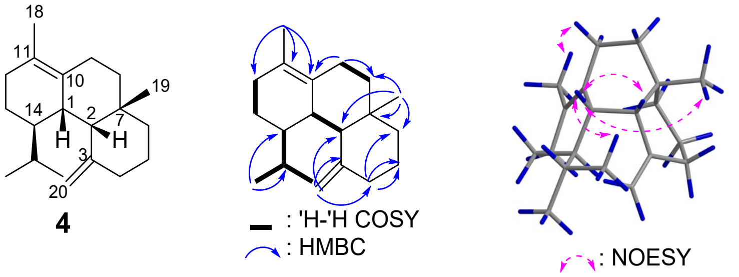

Compound **4** was obtained as colorless oil. Its ^{1}H NMR spectrum (Supplementary Fig. 91) showed two geminal methyls at δ_H 0.90 (3H, d, J = 6.6 Hz, H_3-16) and δ_H 0.99 (3H, d, J = 6.6 Hz, H_3-17), one vinyl methyl at δ_H 1.62 (3H, d, J = 1.3 Hz, H_3-18), and one methyl at δ_H 0.91 (3H, d, J = 0.8 Hz, H_3-19). In addition, one exocyclic double bond at δ_H 4.91 (1H, d, J = 1.8 Hz, H-20) and δ_H 4.48 (1H, d, J = 1.8 Hz, H-20). The ^{13}C NMR and HSQC spectra (Supplementary Fig.s 92 and 93) allowed the identification of 20 carbon resonances, including four olefinic carbons (δ_C 149.2, 133.5, 123.1, 108.1), four methyl carbons (δ_C 21.9, 21.5, 19.2, 18.4), seven methylene carbons (δ_C 44.2, 42.6, 39.2, 27.9, 27.2, 25.2, 21.3), four methyne carbons (δ_C 57.1, 37.5, 37.5, 27.9) and one quaternary carbon (δ_C 38.4). The observed HMBC correlations and ^{1}H-^{1}H COSY correlations are shown above (Supplementary Fig.s 94 and 95). The NOE correlations of H-1 (δ_H 2.17)/H-2 (δ_H 2.02)/H_3-16/H_3-17/ H_3-19, suggested the H-1, H-2, H_3-19 and isopropyl at C-14 have the same orientation (Supplementary Fig. 96).

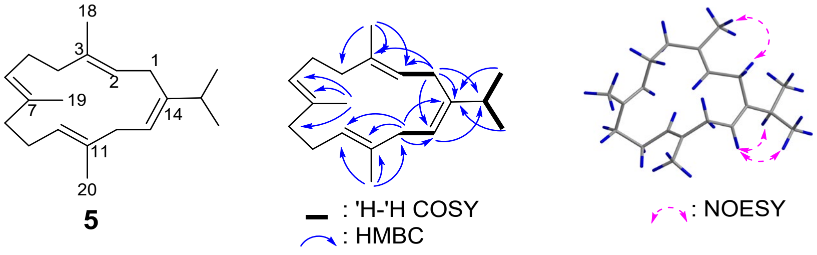

Compound **5** was obtained as colorless oil. Its ^{1}H NMR spectrum (Supplementary Fig. 98) showed two geminal methyls at δ_H 1.03 (6H, d, J = 6.9 Hz, H_3-16, 17), three vinyl methyls at 1.58 (3H, s, H_3-18), 1.51 (3H, s, H_3-19), and 1.61 (3H, d, J = 1.0 Hz, H_3-20), one sp^3 methines at 2.29 (1H, m, H-15), and four *sp*^{2} methines at 4.95 (1H, m, H-2), 4.85 (1H, overlapped, H-6), 4.85 (1H, overlapped, H-10) and 5.27 (1H, m, H-13), typical signals for a cembrane nucleus. The ^{13}C NMR, and HSQC spectra (Supplementary Fig.s 99 and 100) allowed the identification of 20 carbon resonances, including eight olefinic carbons (δ_C 142.3, 134.1, 133.4, 132.2, 126.2, 124.3, 123.5 119.6), five methyl carbons (δ_C 22.3, 22.3, 17.5, 16.2, 15.3), six methylenes (δ_C 39.3, 38.4, 37.2, 28.2, 24.8, 24.7)

and one methynes (δ_C 35.6). The observed HMBC correlations and ^{1}H-^{1}H COSY correlations are shown above (Supplementary Fig.s 101 and 102). The double bonds $\Delta^{2,3}$, $\Delta^{6,7}$, $\Delta^{10,11}$, $\Delta^{13,14}$ were in favor of *E* configurations since the shielded carbon resonances of the three vinyl methyls at C-18 (δ_C 16.2), C-19 (δ_C 15.3), C-20 (δ_C 17.5)[2] and further supported by the NOE correlations of H-1 (δ_H 2.70)/H_3-18, and H-13 /H_3-15/H_3-16/H_3-17 (Supplementary Fig. 103).

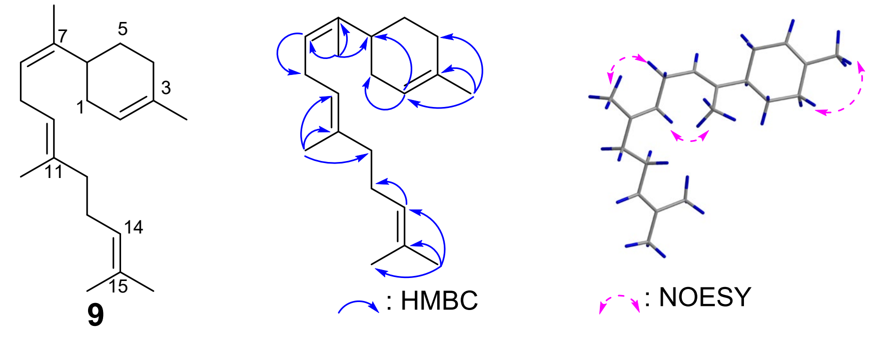

Compound **9** was readily identified as *E*,*E*-2,6-dimethyl-10-(4-methyl-3-cyclohexenyl)-2,6,9-undecatriene by comparing its NMR spectroscopic data (Supplementary Fig.s 117–120) with those reported in the literature[9] and further confirmed by 2D NMR correlations.

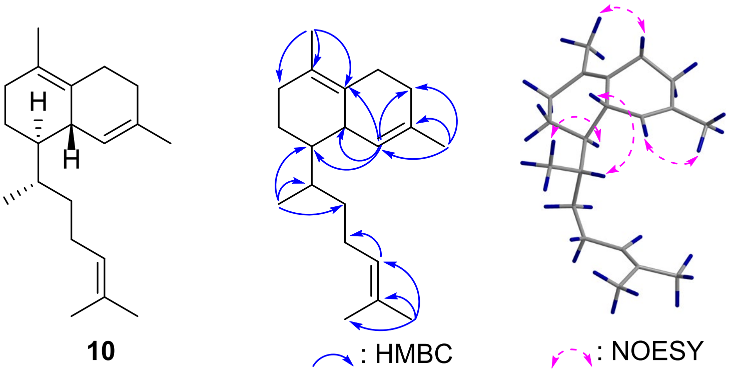

Compound **10**'s planar structure was readily identified as identical to that of isoelisabethatriene B by comparing its NMR spectroscopic data with previously reported data[10]. This identification was further corroborated through 2D NMR correlations. The relative configuration was determined using NOE correlations, as depicted above.

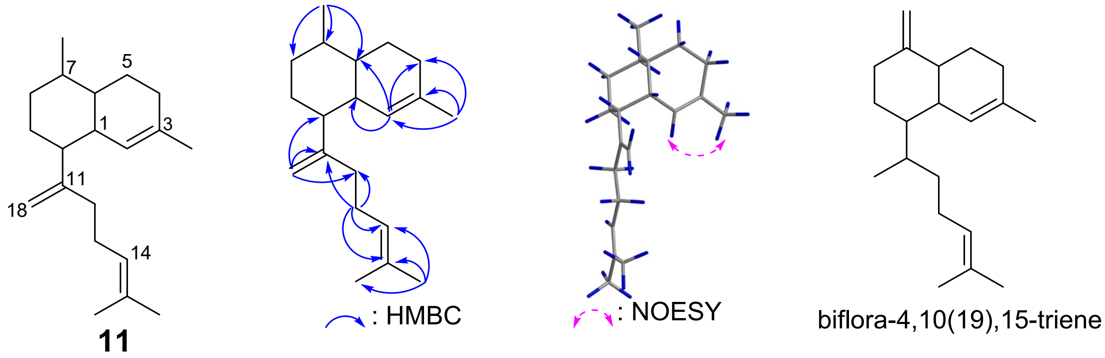

Compound **11** showed high similarity to model compound biflora-4,10(19),15-triene, a diterpene isolated from the frontal gland secretion of soldiers of the termite species *Cubitermes umbratus*

Williams[11]. **11** differs from biflora-4,10(19),15-triene only at the position of the terminal double bonds, where the former is located at 11(12)-double bond and the latter at 10(19)-double bond. The hypothesis was supported by the HMBC correlation from H_2-12 (δ_H 2.10) to C-10 (δ_C 47.6), C-11 (δ_C 153.0), and C-13 (δ_C 27.1), and from H-14 (δ_H 5.27) to C-11, C-13, and C-15 (δ_C 131.2). Thus, the planer structure of **11** was identified as shown above, namely biflora-4,11(12),15-triene.

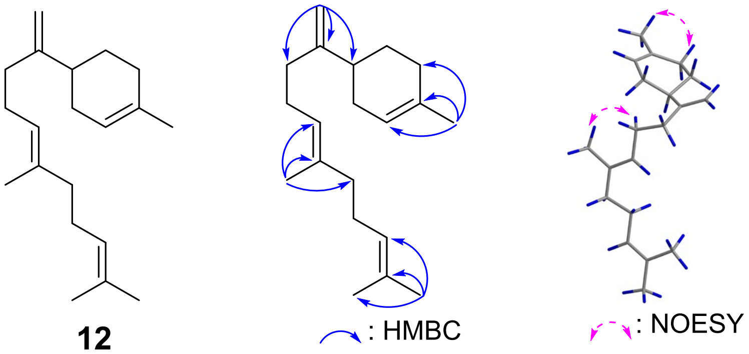

Compound **12** was readily identified as (-)-axinyssene by comparing its NMR spectroscopic data (Supplementary Fig. 131–134) and optical rotation value with those reported in the literature[9] and confirmed by 2D NMR correlations.

Synthesis of 10F-GGPP

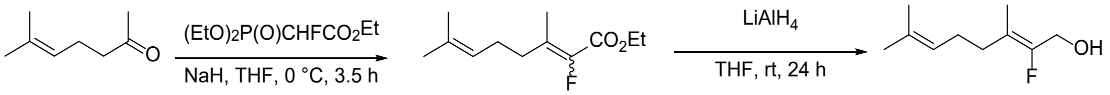

(2*Z*)-2-Fluoro-3,7-dimethylocta-2,6-dien-1-ol. Conditions for the following reaction were based on those described by Jin Yinghua[12]. To a cooled (0 °C) and well-stirred suspension of NaH (1.65 g, 60% dispersion in mineral oil, 41.2 mmol) in dry THF (100 mL) was added triethyl 2-fluoro-2-phosphonoacetate (8.3 mL, 41.2 mmol) slowly. 2.64 mL 6-methyl-5-hepten-2-one (17.88 mmol) was added to the resulting yellow solution. The mixture was stirred for three and a half hours at room temperature and poured into cold water (80 mL). The organic layer was separated, and the aqueous layer was extracted with Et_2O (3 × 50 mL). The combined organic extracts were washed with water (2 × 100 mL) and brine (2 × 100 mL) and dried over $MgSO_4$. Evaporation of the solvent afforded the fluoro ester, which was purified by flash CC on silica gel with PE/EA (200:1) to yield 3.62 g pure fluoro ester as mixtures of 2*E* and 2*Z* diastereomers (1:1). The ester (3.62 g, 16.89 mmol) in THF (60 mL) was stirred and cooled at 0 °C as $LiAlH_4$ (1.28 g, 33.78 mmol) was added in one portion. The suspension was stirred at room temperature for two hours and quenched by the addition of 1.3 mL water dropwise, 1.3 mL 15% NaOH, and 3.9 mL water. The mixture was extracted three times with Et_2O. The combined organic layers were dried with $MgSO_4$ and concentrated under reduced pressure. The residue was purified by CC on silica gel with PE/EA (20:1) to give the (2*Z*)-2-fluoro-3,7-dimethylocta-2,6-dien-1-ol (1.80 g, 10.45 mmol). ^{1}H NMR ($CDCl_3$, 600 MHz) of (2*Z*)-2-fluoro-3,7-dimethylocta-2, 6-dien-1-ol: δ 1.59 (brs, 3H, CH_3), 1.65 (d, 3H, J = 3.1 Hz, CH_3), 1.67 (s, 3H, CH_3), 2.04–2.13 (m, 4H, CH_2), 4.21 (dd, 2H, J = 22.6, 3.8 Hz, CH_2OH), 5.10 (t, 1H, J = 6.9 Hz, vinyl H).

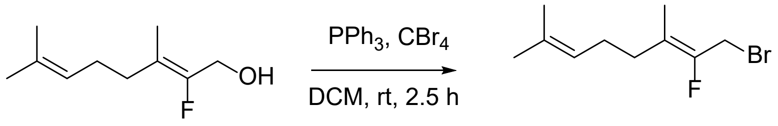

(2*Z*)-1-Bromo-2-fluoro-3,7-dimethylocta-2,6-diene. A solution of alcohol (1.80 g, 10.45 mmol) in dry DCM (50 mL) was stirred and cooled to 0 °C. 1.5 Eq. CBr_4 (5.19 g, 15.67 mmol) was added, and 4.11 g PPh_3 (1.5 eq., 15.67 mmol) was added slowly. The suspension was stirred at room temperature for two and a half hours and concentrated under reduced pressure, at which time the reaction was judged complete by TLC analysis. The residue was purified by CC on silica gel with PE to give the (2*Z*)-1-bromo-2-fluoro-3,7-dimethylocta-2,6-diene (2.38 g, 10.12 mmol). ^{1}H NMR ($CDCl_3$, 600 MHz) of (2*Z*)-1-bromo-2-fluoro-3,7-dimethylocta-2,6-diene: δ 1.60 (brs, 3H, CH_3), 1.67 (d, 3H, $J = 3.0$ Hz, CH_3), 1.68 (s, 3H, CH_3), 2.06–2.16 (m, 4H, CH_2), 4.07 (d, 2H, $J = 23.0$ Hz, CH_2Br), 5.08 (t, 1H, $J = 6.7$ Hz, vinyl H).

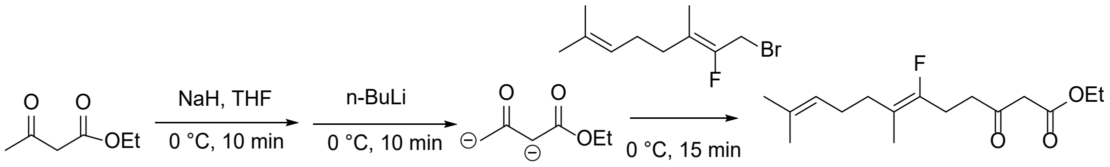

(6*Z*)-6-Fluoro-7,11-dimethyl-3-oxododeca-6,10-dienoic acid, ethyl ester. The procedure reported by Jin Yinghua[12] was followed. To a suspension of NaH (1.34 g, 60% dispersion in mineral oil, 33.40 mmol) in dry THF (40 mL) was added ethyl acetoacetate (3.84 mL, 30.36 mmol) dropwise at 0 °C. After 10 min, *n*-BuLi (1.6 M in hexane, 19.92 mL, 31.88 mmol) was added slowly over 5 min. It was stirred for an additional 10 min at 0 °C, as (2*Z*)-1-bromo-2-fluoro-3,7-dimethylocta-2,6-diene (2.38 g, 10.12 mmol) was added. After 15 min, the TLC analysis showed the reaction was complete and HCl (3 M, 10 mL) was added. Water (50 mL) was added, and the organic layer were separated. The aqueous layer was extracted with Et_2O (3 × 50 mL). The combined organic layers were washed with water (2 × 100 mL) and saturated NaCl solution (1 × 100 mL) and then dried over Na_2SO_4. Removal of the solvent gave the crude product, and purification by CC afforded (6*Z*)-6-fluoro-7,11-dimethyl-3-oxo dodeca-6,10-dienoic acid, ethyl ester (1.29 g, 4.55 mmol).

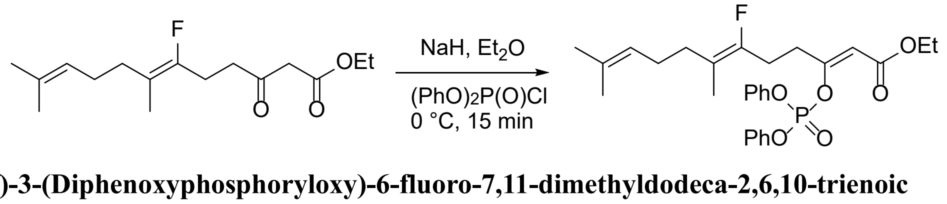

(2*Z*,6*Z*)-3-(Diphenoxyphosphoryloxy)-6-fluoro-7,11-dimethyldodeca-2,6,10-trienoic acid, ethyl ester. The enol phosphorylation procedure by Jin Yinghua[12] was followed to convert (6*Z*)-6-fluoro-7,11-dimethyl-3-oxo dodeca-6,10-dienoic acid, ethyl ester to the enol phosphate with certain modifications. A suspension of NaH (200 mg, 5.00 mmol, 60 % in mineral oil) in Et_2O (60 mL) was stirred and cooled to 0 °C as a solution of (6*Z*)-6-fluoro-7,11-dimethyl-3-oxododeca-6,10-dienoic acid, ethyl ester (1.29 g, 4.55 mmol) in Et_2O (5 mL) was added slowly. The mixture was then stirred at 0 °C for 30 min until the bubbling stopped. Diphenyl chlorophosphate (1.83 g, 6.82 mmol) was added slowly and stirring was continued at 0 °C for additional 15 min at which time TLC analysis showed that all the substrate was consumed. The reaction was quenched by adding saturated NH_4Cl

solution (60 mL). The mixture was diluted with water (30 mL), and the organic layer was separated. The aqueous layer was extracted with Et_2O (3 × 80 mL). The combined ethereal extracts were washed with water (2 × 100 mL), dried over $MgSO_4$, concentrated to give light yellow oil, and purification by CC afforded (2*Z*,6*Z*)-3-(diphenoxyphosphoryloxy)-6-fluoro-7,11-dimethyldodeca-2,6,10-trienoic acid, ethyl ester (2.14 g, 4.15 mmol).

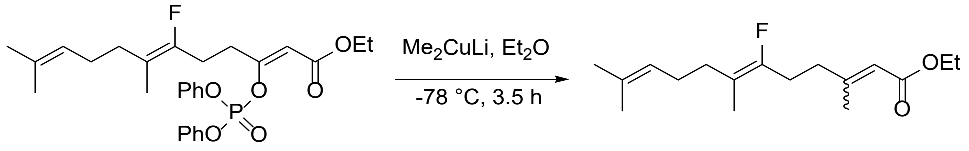

(2*E*/*Z*, 6*Z*)-6-Fluoro-3,7,11-trimethyldodeca-2,6,10-trienoicacid, ethyl ester. The procedure reported by Jin Yinghua[12] was followed with certain modifications. A suspension of (2*Z*, 6*Z*)-3-(diphenoxyphosphoryloxy)-6-fluoro-7,11-dimethyldodeca-2,6,10,14-trienoic acid, ethyl ester (2.14 g, 4.15 mmol) in Et_2O (40 mL) was stirred and cooled at -78 °C as Me_2CuLi (16.60 mL, 8.30 mmol, 0.5 M in Et_2O) was added. The resulting yellow solution was stirred for three and a half hours at -78 °C. MeI (5.89 g, 41.50 mmol) was added, and the mixture was stirred for 15 min at -78 °C. The mixture was poured into an ice-cooled mixture of saturated NH_4Cl solution and concentrated NH_4OH (100 mL, 1:1, v/v mixture). The product was extracted with Et_2O (3 × 100 mL). The combined organic layers were washed with water (3 × 100 mL), dried over Na_2SO_4, concentrated, and purification by CC to give a mixture of *trans*/*cis* isomers (913.68 mg, 3.24 mmol).

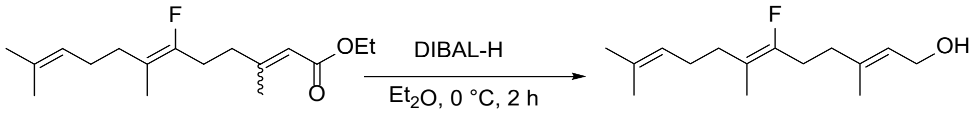

(2*E*,6*Z*)-6-Fluoro-3,7,11-trimethyldodeca-2,6,10-trien-1-ol. The (2*E*/*Z*, 6*Z*)-6-fluoro-3,7,11-trimethyldodeca-2,6,10-trienoic acid, ethyl ester (913.68 mg, 3.24 mmol) was dissolved in dry Et_2O and cooled to 0 °C. After the addition of DIBALH (1 M in hexane, 7.13 mL, 7.13 mmol, 2.2 eq.), the reaction mixture was stirred for two hours. The reaction was quenched by adding 0.3 mL water dropwise, 0.3 mL 15% NaOH, and 1 mL water. The mixture was extracted three times with Et_2O. The combined organic layers were dried with $MgSO_4$ and concentrated under reduced pressure. The residue was purified by CC on silica gel with PE/EA (20:1) to give the (2*E*,6*Z*)-6-fluoro-3,7,11-trimethyldodeca-2,6,10-trien-1-ol. ^1H NMR ($CDCl_3$, 600 MHz) of (2*E*,6*Z*)-6-fluoro-3,7,11-trimethyldodeca-2,6,10-trien-1-ol: δ 1.56 (d, 3H, J = 2.7 Hz, CH_3), 1.60 (s, 3H, CH_3), 1.68 (s, 3H, CH_3), 1.70 (s, 3H, CH_3), 2.02–2.09 (m, 4H, CH_2), 2.17–2.21 (m, 2H, CH_2), 2.30–2.37 (m, 2H, CH_2), 4.14 (s, 1H, CH_2), 4.15 (s, 1H, CH_2), 5.10 (m, 1H, vinyl H), 5.44 (m, 1H, vinyl H).

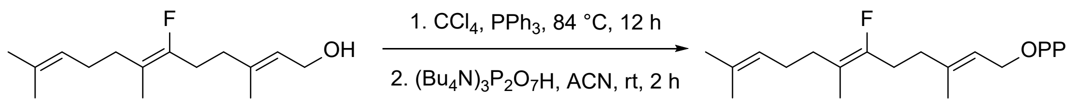

6F-FPP. Diphosphorylation of (2*E*,6*Z*)-6-fluoro-3,7,11-trimethyldodeca-2,6,10-trien-1-ol was carried out as described above for GLPP. ^1H NMR (D_2O, 600 MHz) of 6F-FPP: δ 1.59 (d, 3H, J = 2.5 Hz, CH_3), 1.62 (s, 3H, CH_3), 1.69 (s, 3H, CH_3), 1.74 (s, 3H, CH_3), 2.08–2.12 (m, 4H, CH_2), 2.22–2.26 (m, 2H, CH_2), 2.38–2.46 (m, 2H, CH_2), 4.47 (t, 2H, J = 6.7 Hz, CH_2), 5.19 (m, 1H, vinyl H), 5.48 (m, 1H, vinyl H)

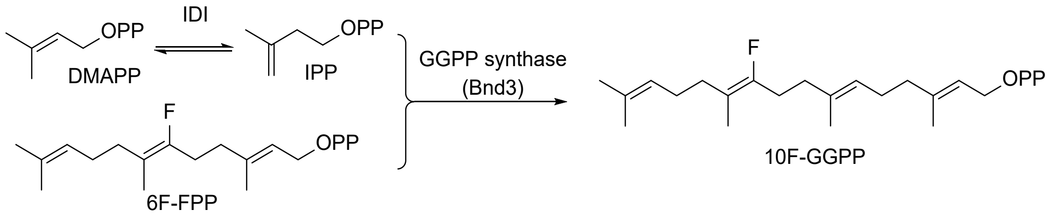

10F-GGPP. To in situ biosynthesis of 10F-GGPP, we purified isopentenyl-pyrophosphate delta isomerase (IDI), which can catalyze abstraction of one proton from water to DMAPP at C2 in the pro-R position to yield IPP, and GGPP synthase Bnd3, which can produce GGPP from FPP and IPP. Incubation of Bnd3 and IDI together with DMAPP and 6F-FPP would give 10F-GGPP[13].

Metadynamic system construction and simulation method

The CHARMM-GUI server[14] was used to prepare the simulation inputs, including TIP3P solvent with 0.15 M Na^+/Cl^- ions, and the CHARMM36 force field[15]. The force filed of the ligands was generated by CGenFF program[16]. All MD simulations were performed using GROMACS-2019.4 (*ref.* 17). Energy minimization was performed for 5000 steps by the steepest descent algorithm and then a 125 ps NVT simulation at 300K was generated for the solvent equilibration using the Berendsen thermostat with heavy atoms restrained at 10.0 kcal mol^{-1} $Å^{-2}$. The equilibration was applied at 300 K and 1 atm for 1 ns in NPT ensemble, using the Berendsen thermostat and barostat. The production simulations were run at 300 K and 1 atm in the NPT ensemble for 100 ns with a time step of 2 fs, using the Nosé–Hoover Langevin thermostat and the Parrinello-Rahman barostat. A cutoff of 12 Å was used for the van der Waals and short-range electrostatic interactions. Long-range electrostatic interactions were treated by the Particle mesh Ewald algorithm[18]. The covalent bonds containing hydrogen atoms were constrained using the LINCS algorithm[19]. Gromacs-2019.4 and plumed-2.5 (*ref.* 20). was used to conduct the well-tempered metadynamics[21] with a Gaussian height of 0.125 kj/mol, a sigma value of 0.15 rad. New Gaussians was added every 0.001 ps. The collective variables were defined as follows: CV1 was defined as the dihedral about C-18, C-11, C-13, C-12 and CV2 as the dihedral about C-11, C-12, C-13, C-14.

Supplementary Figure

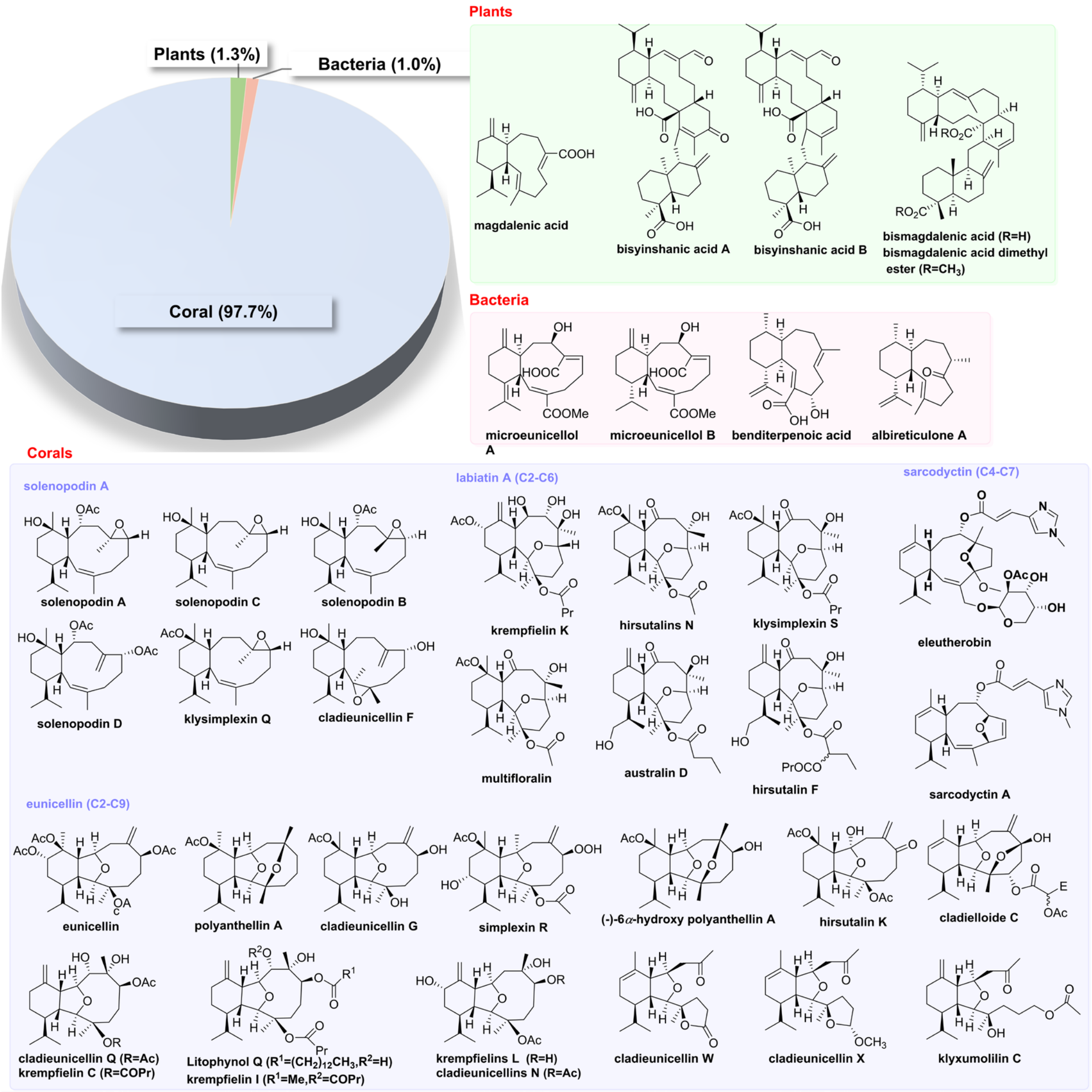

Supplementary Fig. 1. Eunicellane diterpenoids. Up to now, nearly 400 eunicellanes have been reported, mostly from soft corals (accounts for 97.7%), only a few examples from plants (accounts for 1.3%) and, most recently, bacteria (1.0%). Based on the presence or absence of transannular ether bridges, eunicellanes can be classified into four classes: compounds without transannular ether bridges (known as solenopodin A-like compounds), compounds with an ether bridge between C2 and C6 (referred to labiatin A-like compounds), compounds with a C4-C7 ether bridge (known as sarcodyctin A-like compounds), and compounds with a C2-C9 ether bridge (referred as eunicellin). Among these compounds, eunicellin occupies the highest proportion and is the predominant structural type. With their diverse range of biological activities, including anti-inflammatory, anticancer, antibacterial, and antifouling properties, this family of natural products presents enticing prospects for pharmaceutical and agricultural applications[22–26].

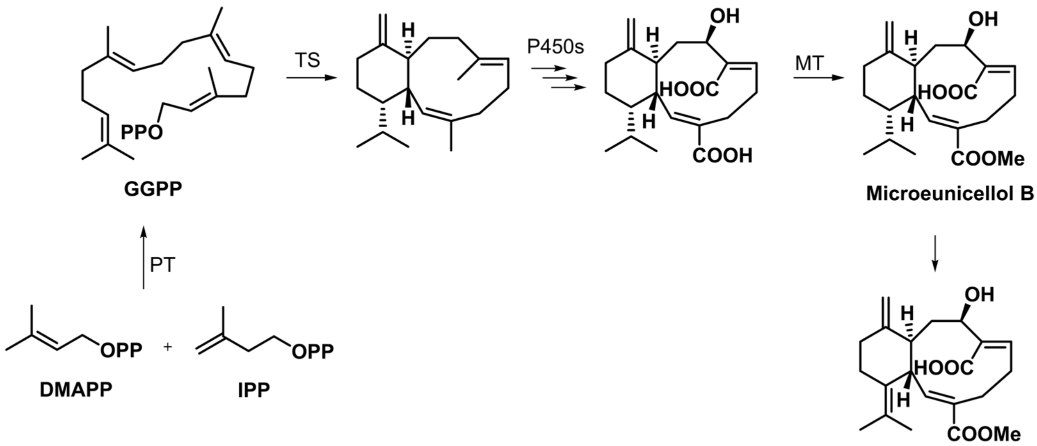

Supplementary Fig. 2. Biosynthetic hypothesis for microeunicellols A and B. The term “PT” refers to “polyprenyl diphosphate synthase”, “TS” denotes “terpene synthase”, “MT” is an abbreviation for “methyltransferase”.

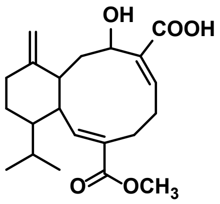

Patent Number: CN109651154

Title: Preparation method of diterpenoid compound and its application.

Strain: *Micromonospora* sp.HM134

https://patents.google.com/patent/CN109651154A/en?oq=CN109651154

‘The obtained bacterium’ mentioned in the article was *Streptomyces albogriseolus* SY67903 (*ref.* 27). We acquired the strain from one of the authors of the paper, then proceeded to sequence and analyze it. Regrettably, we were unable to pinpoint the pertinent biosynthetic gene cluster within this strain.

Supplementary Fig. 3. Patent information of *Micromonospora* sp. HM134. The Chinese patent detailed the production of a microeunicellol-like compound by a bacterium named *Micromonospora* sp. HM134. The reason why we called it microeunicellol-like compound is that it did not show stereochemistries.

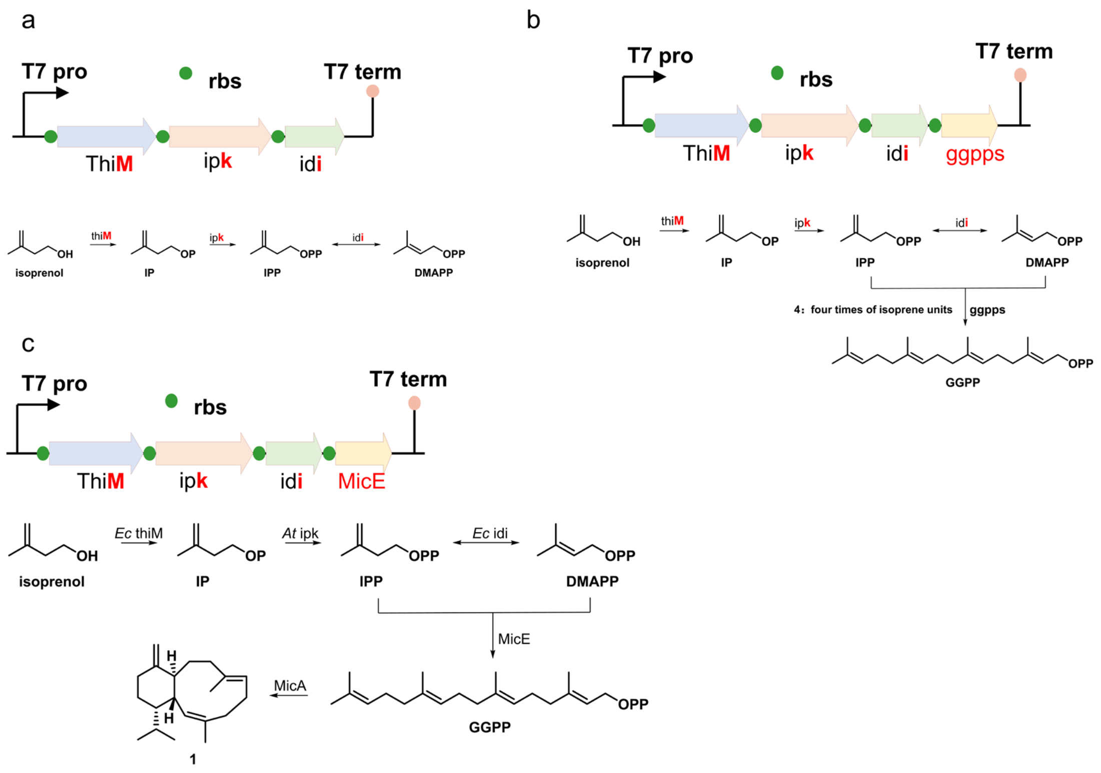

Supplementary Fig. 4. Diterpene overproduction system in *E. coli*. (a) Plasmid design of CDF-MKI. The "MKI" expresses three genes that convert isoprenol into DMAPP, where "MKI" is an abbreviation for these three genes. “M” refers to a kinase, hydroxyethylthiazole kinase (ThiM) from *E. coli*; “K” refers to a kinase, isopentenyl phosphate kinase (ipk) from *Arabidopsis thaliana*; “I” refers to isopentenyl diphosphate isomerase (idi) from *E. coli*[13]. All genes are under a single T7 promoter-*lacO* transcription/regulation module. Ribosome binding sites (rbs) were included before each gene to ensure maximum translation. (b) Plasmid design of CDF-MKI4. The "MKI4" encodes for four genes responsible for converting isoprenol into GGPP, with "MKI" denoting an abbreviation for the three genes previously mentioned. The "4" signifies the capability of the recombinant strain to produce four times the isoprene units. In another word, a GGPP synthase (bnd3) has been introduced in the “MKI” system for generating C20 polyprenyl pyrophosphate substrate. (c) The production pathway of **1** in *E. coli* that carries CDF-MKI-MicE and pET28a-MicA. MicE, is a polyprenyl diphosphate synthase from *Micromonospora* sp. HM134.

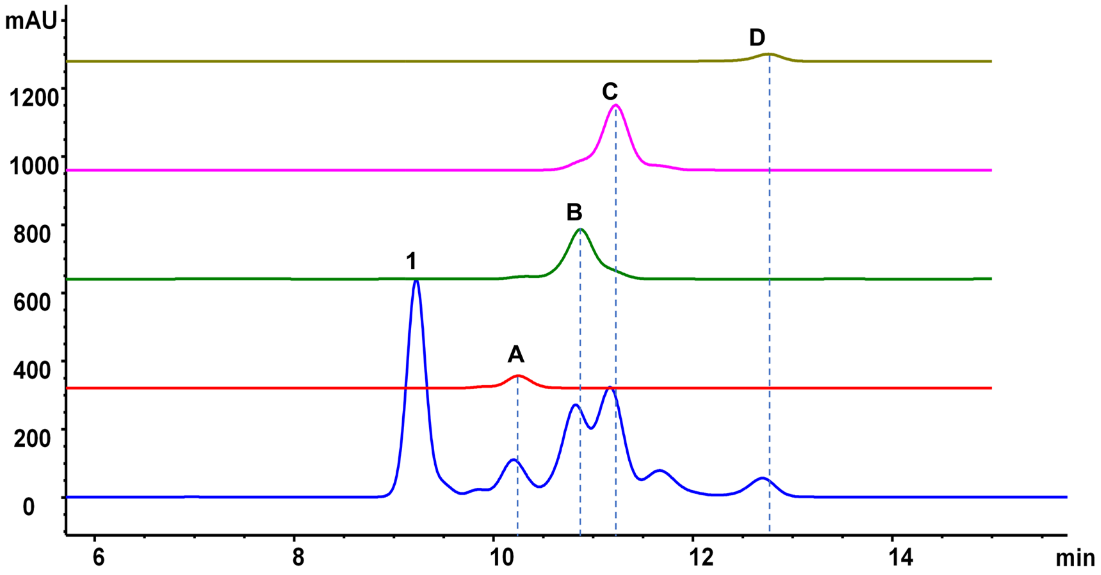

Supplementary Fig. 5. HPLC analyses (210 nm) of minor products. Minor products were fractionated by reversed-phase HPLC [(CH_3CN)/H_2O (95:5)], yielding four fractions (A–D).

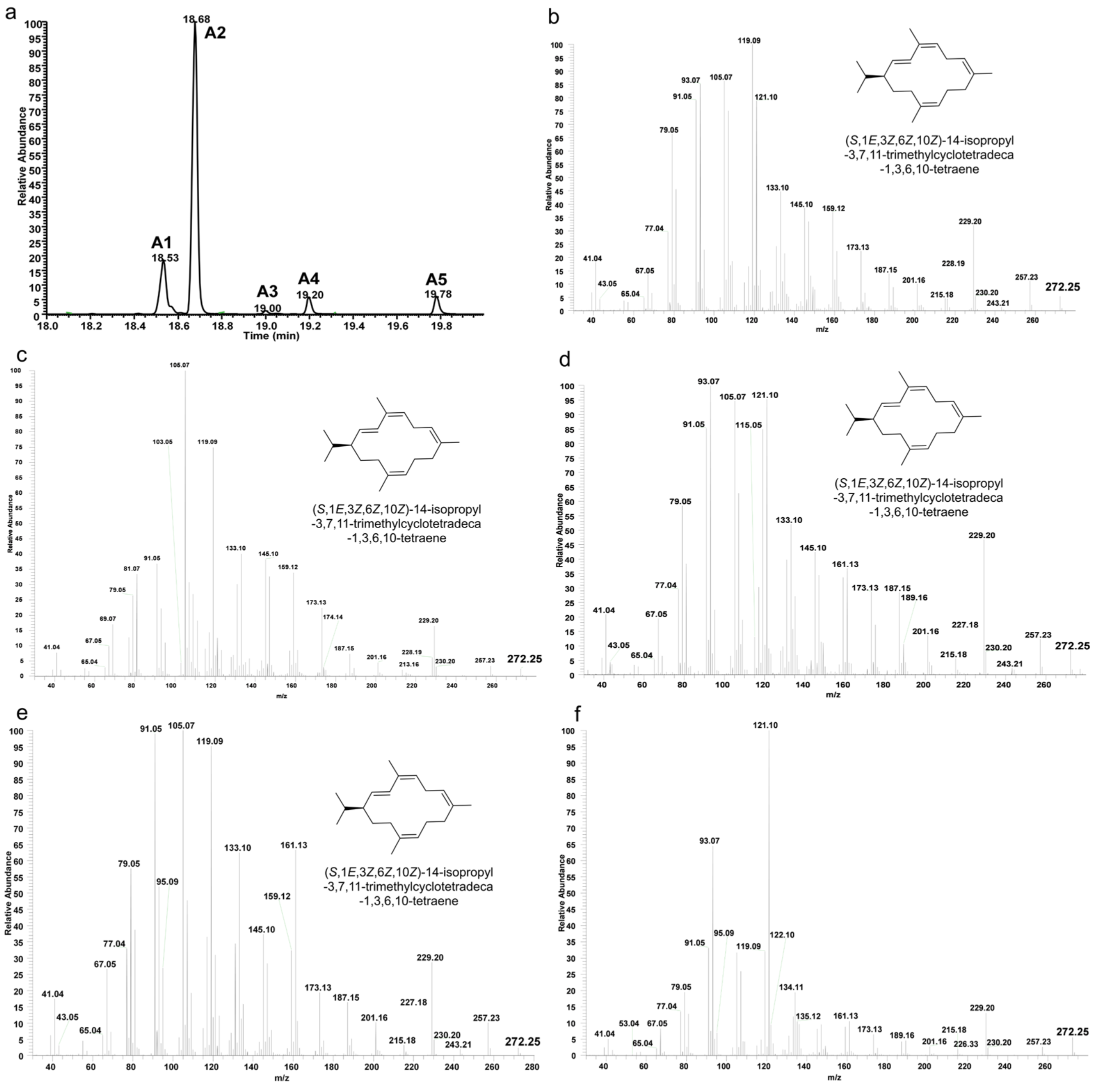

Supplementary Fig. 6. GC-MS spectra of fraction A from minor products. (a) Total ion chromatogram (TIC) of fraction A. (b) Mass spectra for peak A1. Comparison with the NIST database annotated it as (*S*,1*E*,3*Z*,6*Z*,10*Z*)-14-isopropyl-3,7,11-trimethylcyclotetradeca-1,3,6,10-tetraene. The RHRF score was 99.7896. (c) Mass spectra for peak A2. The RHRF score was 99.8921. (d) Mass spectra for peak A3. The RHRF score was 98.9727. (e) Mass spectra for peak A4. The RHRF score was 99.743. (f) Mass spectra for peak A5.

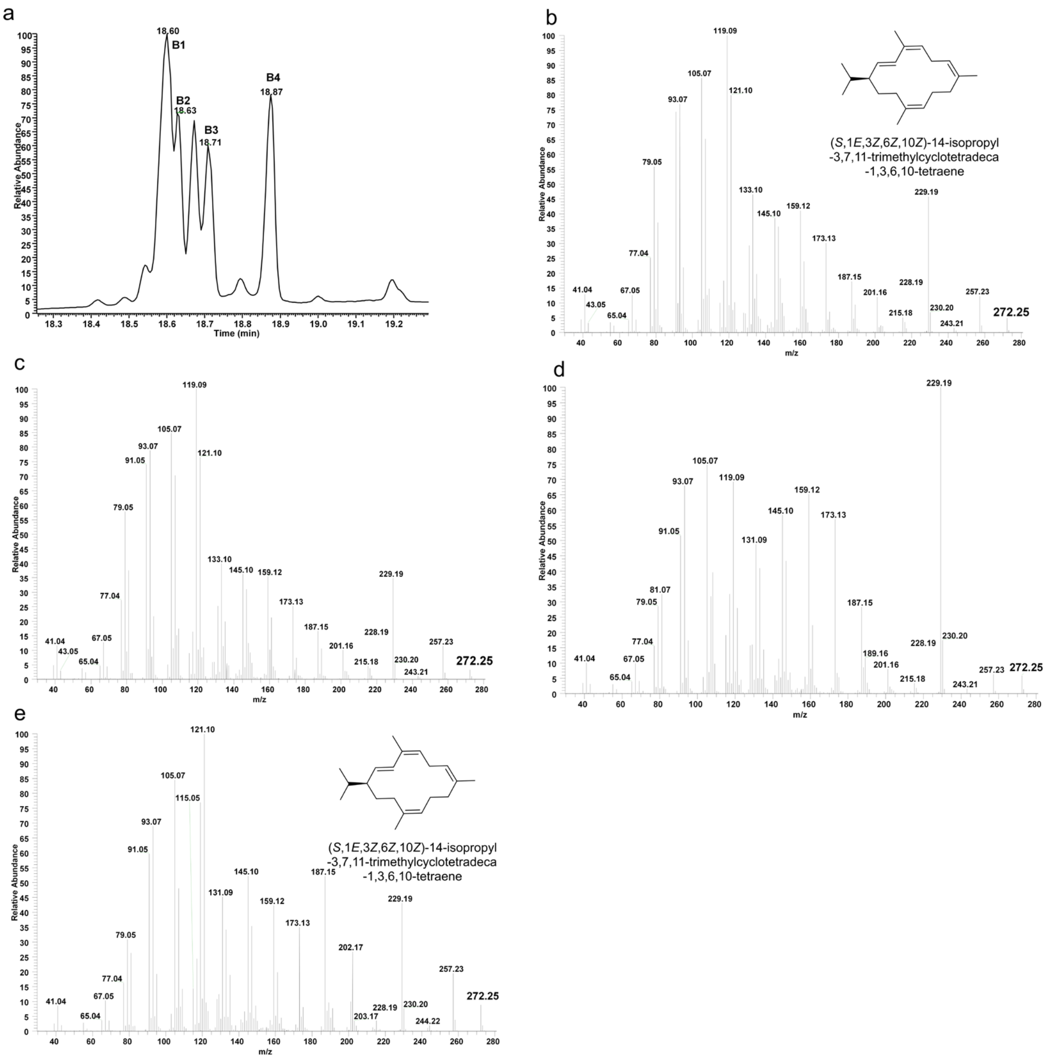

Supplementary Fig. 7. GC-MS spectra of fraction B from minor products. (a) Total ion chromatogram (TIC) of fraction B. (b) Mass spectra for peak B1. The RHRF score was 99.7492. (c) Mass spectra for peak B2. (d) Mass spectra for peak B3. (e) Mass spectra for peak B4. The RHRF score was 99.8447.

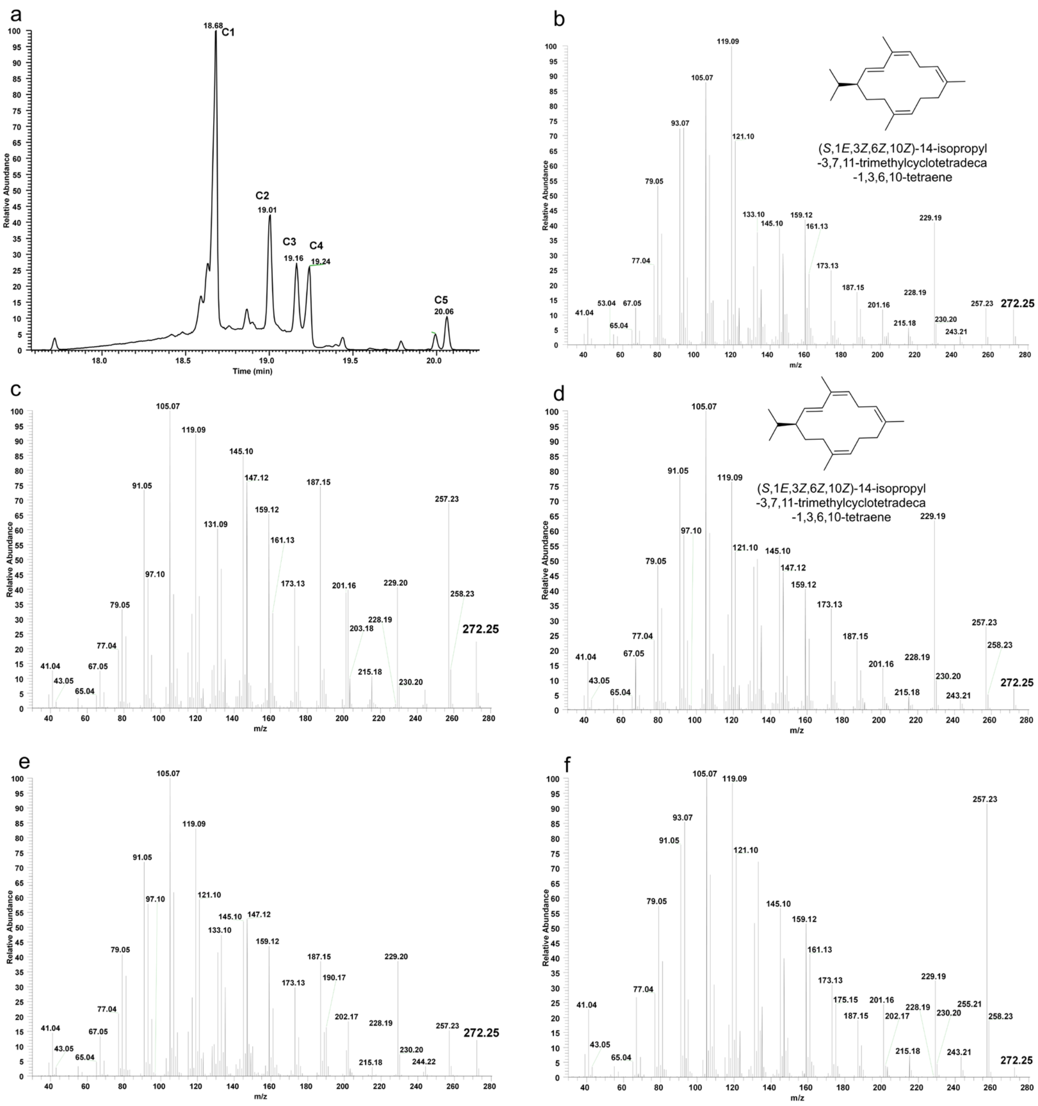

Supplementary Fig. 8. GC-MS spectra of fraction C from minor products. (a) Total ion chromatogram (TIC) of fraction C. (b) Mass spectra for peak C1. The RHRF score was 99.7547. (c) Mass spectra for peak C2. (d) Mass spectra for peak C3. The RHRF score was 99.864. (e) Mass spectra for peak C4. (f) Mass spectra for peak C5.

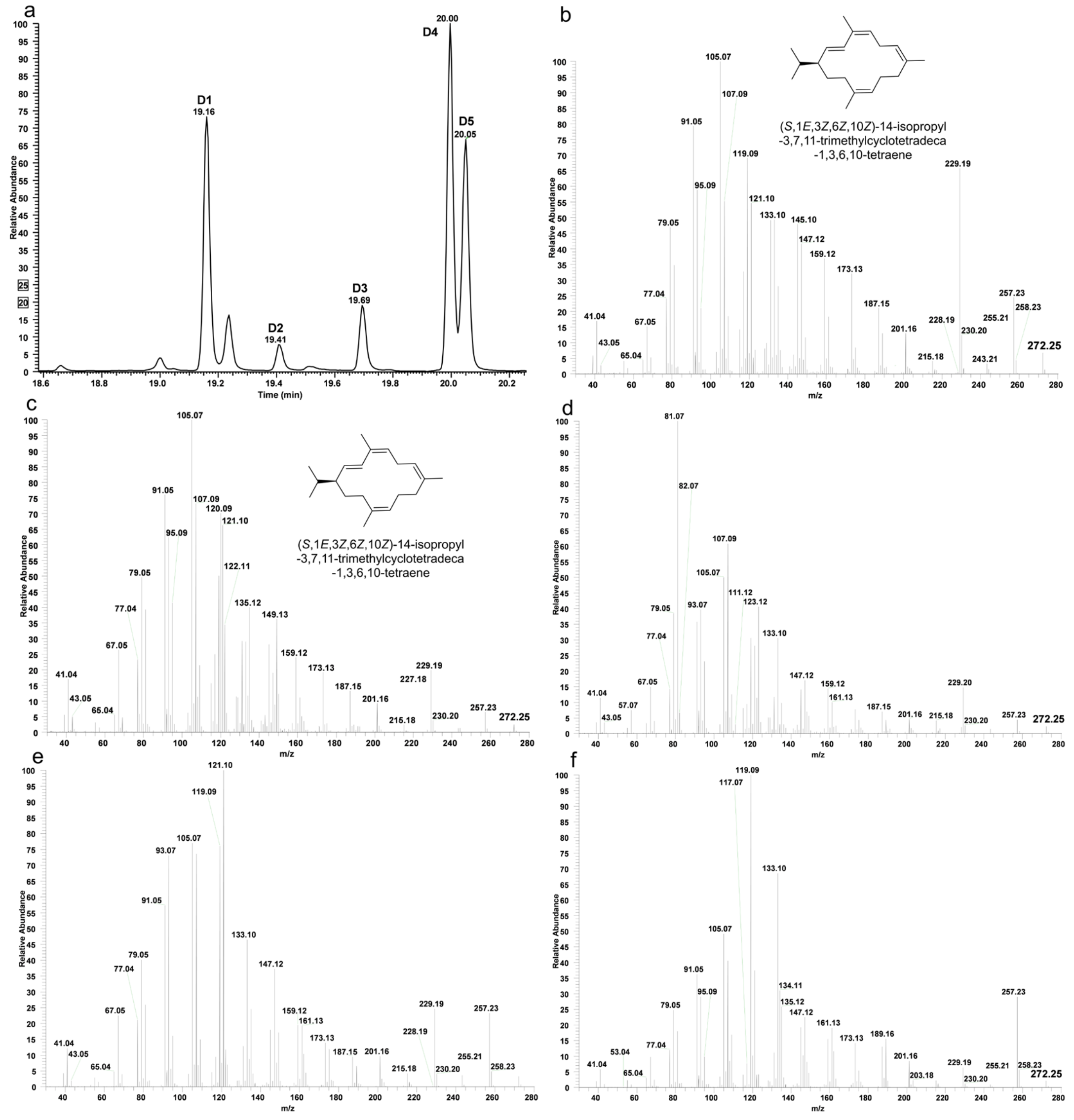

Supplementary Fig. 9. GC-MS spectra of fraction D from minor products. (a) Total ion chromatogram (TIC) of fraction D. (b) Mass spectra for peak D1. The RHRF score was 99.8488. (c) Mass spectra for peak D2. The RHRF score was 98.9965. (d) Mass spectra for peak D3. (e) Mass spectra for peak D4. (f) Mass spectra for peak D5.

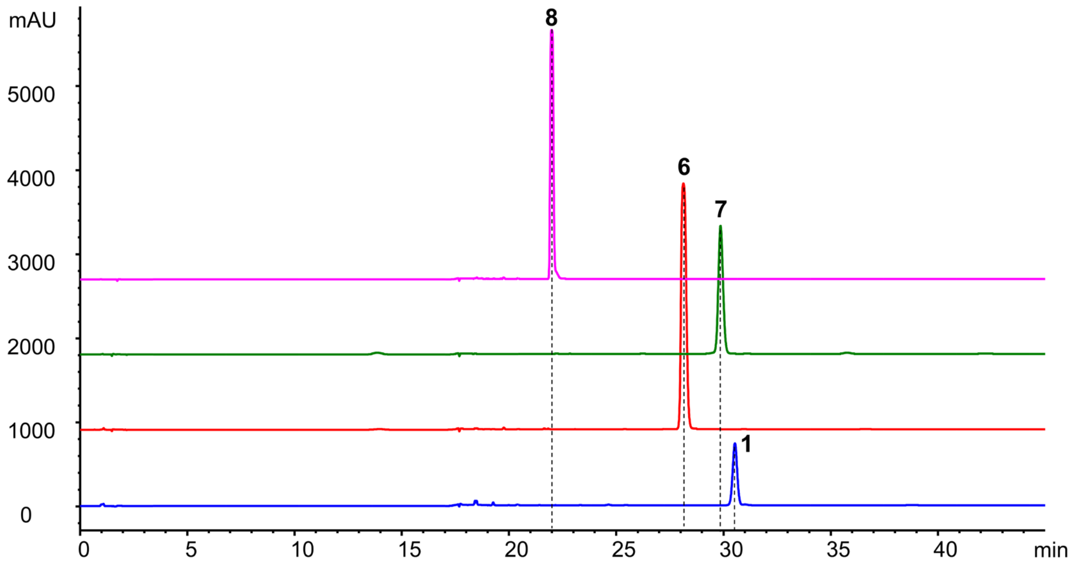

Supplementary Fig. 10. Purity report of compounds 1 and 6–8. They were analyzed by HPLC (210 nm) at 35°C with a flow rate of 1 mL/min. The linear gradient program was run as follows: 0−15 min, 5% CH_3CN; 15–16 min, 5–95% CH_3CN; 16−45 min, 95% CH_3CN. The purity of compounds **1** and **6–8** was 94.8%, 99.8%, 95.4%, and 99.3% respectively.

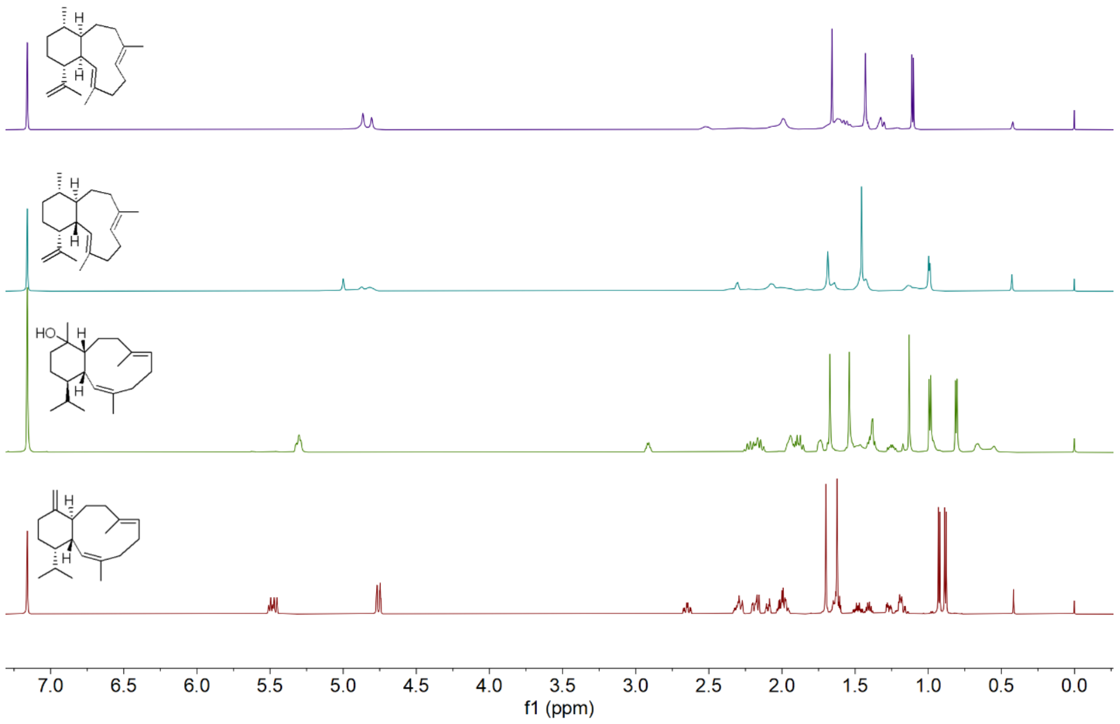

Supplementary Fig. 11. ^{1}H NMR spectra (600 MHz) of compounds 1 and 6–8. The spectra were recorded under identical conditions using the same instrument. Each spectrum was acquired with 4 scans and the sample concentration was standardized to 10 mg in 500 μL of deuterated benzene at 298K.

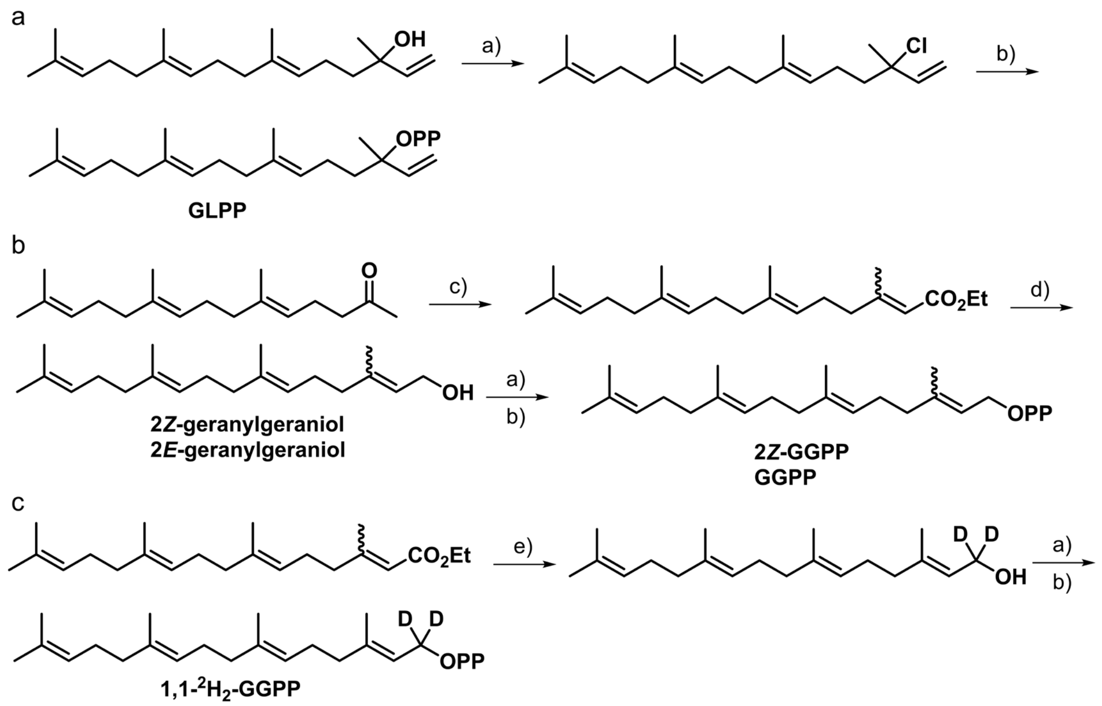

Supplementary Fig. 12. Synthesis of GLPP, GGPP, 2*Z*-GGPP and 1,1-$^{2}H_{2}$-GGPP. (a) The synthesis of GLPP. (b) The synthesis of GGPP and 2*Z*-GGPP. (c) Synthesis of 1,1-$^{2}H_{2}$-GGPP. Reaction conditions: a) CCl_4, PPh_3, 84 °C, 12 h; b) $(Bu_4N)_3P_2O_7H$, CH_3CN, rt, 2 h; c) $(EtO)_2P(O)CH_2CO_2Et$, LDA, THF, -78 °C, 12 h; d) DIBAlH, Et_2O, 0 °C, 2 h; e) $LiAl^{2}H_4$, THF, 0 °C, 4 h.

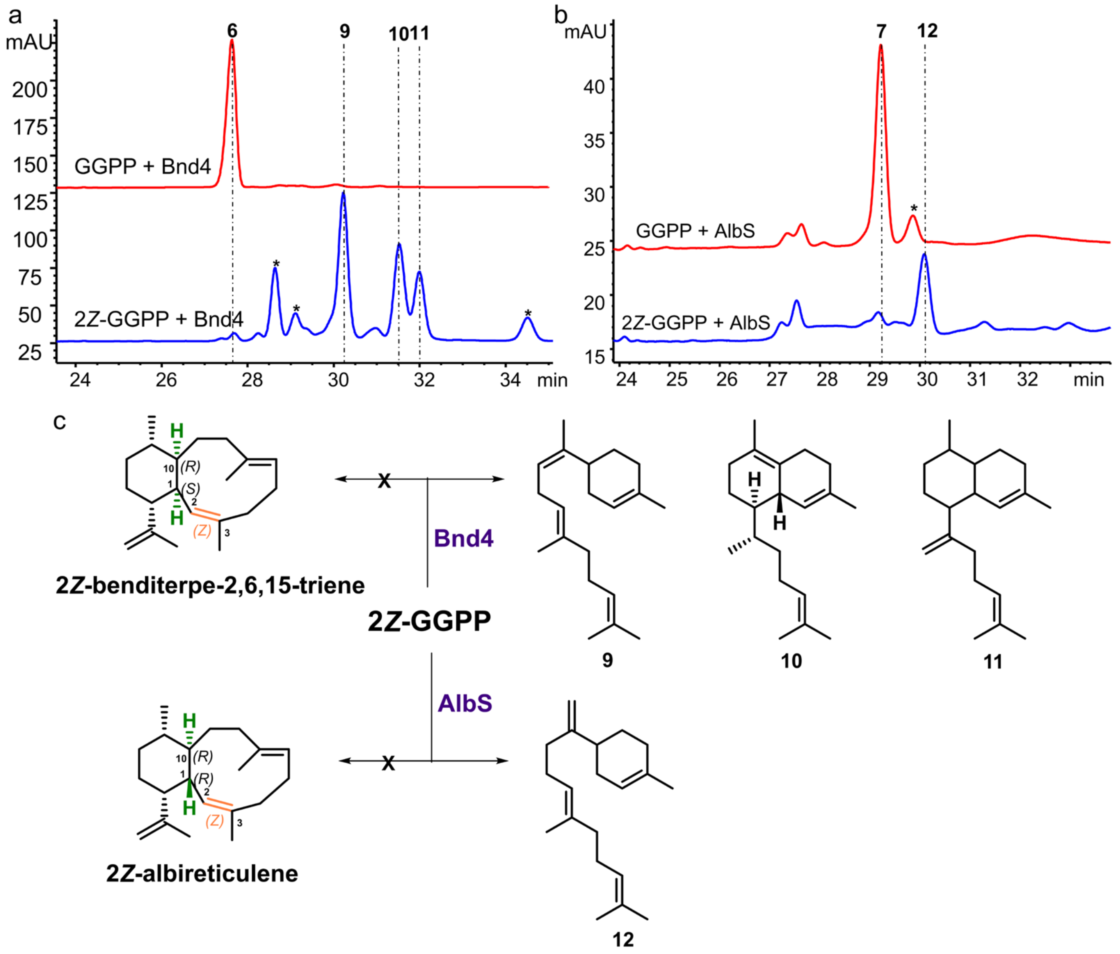

Supplementary Fig. 13. In vitro assays of Bnd4 and AlbS with GGPP and 2*Z*-GGPP. (a) Incubation of Bnd4 with GGPP and 2*Z*-GGPP, and its HPLC traces (210 nm) (b) Incubation of AlbS with GGPP and 2*Z*-GGPP, and its HPLC traces (210 nm) (c) Compound identification of 2*Z*-GGPP incubated with Bnd4 and AlbS individually led to the isolation of compounds (**9**–**12**). Peaks labeled with asterisks (*) were uncharacterized.

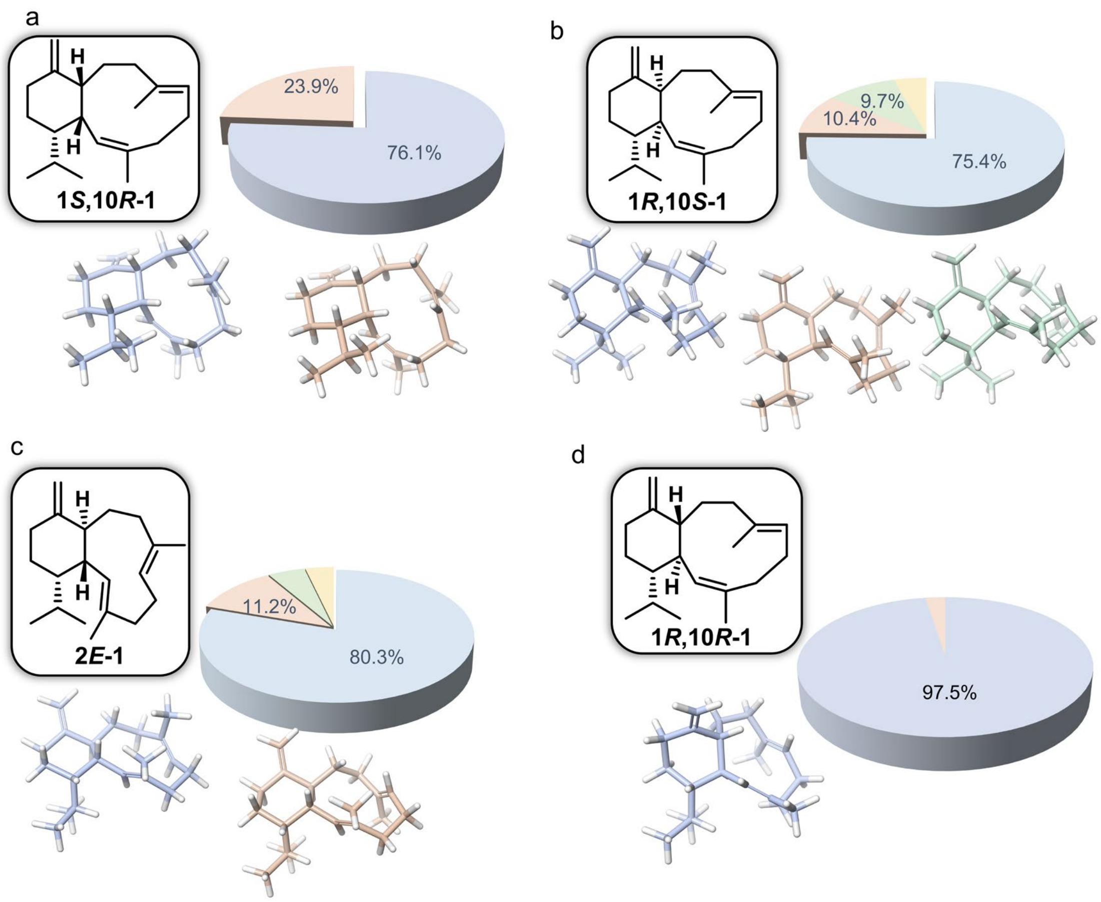

Supplementary Fig. 14. DFT calculation of the hypothesized 1*S*,10*R*-1, 1*R*,10*S*-1, 2*E*-1, and 1*R*,10*R*-1. (a) Boltzmann populations of **1*S*,10*R*-1**. (b) Boltzmann populations of **1*R*,10*S*-1**. (c) Boltzmann populations of **2*E*-1**. (d) Boltzmann populations of **1*R*,10*R*-1**. Boltzmann populations were computed using DFT calculation at the mPW1PW91/6-31+G(d,p)//B3LYP/6-31+G(d,p) level. A pie chart was generated to statistically analyze all conformations of the compound. Only conformations with a percentage above 10% were displayed, where the colors of the pie chart correspond to the colors assigned to the conformation.

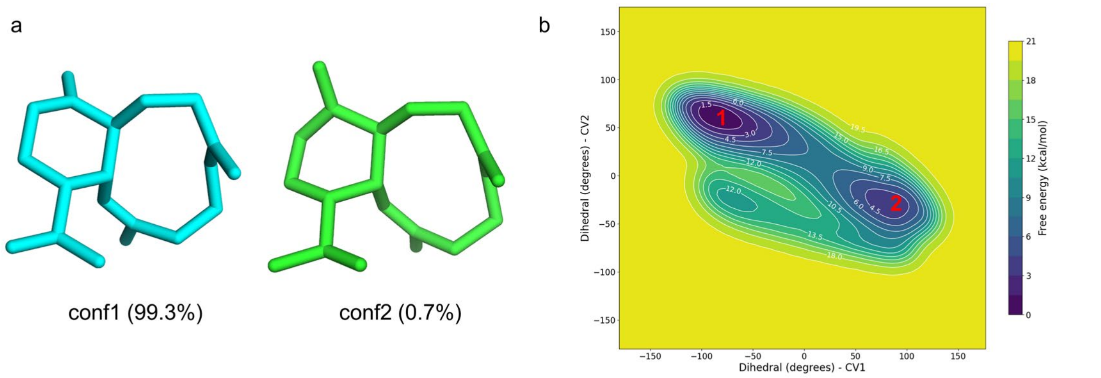

Supplementary Fig. 15. Metadynamics sampling and free energy calculation of 1 (the MicA product). (a) Two conformations of compound **1** (b) Metadynamics free energy calculation results.

Metadynamics simulations indicated that Δ*G* between them is approximately 3 kcal/mol, with a corresponding energy barrier of about 7.5 kcal/mol.

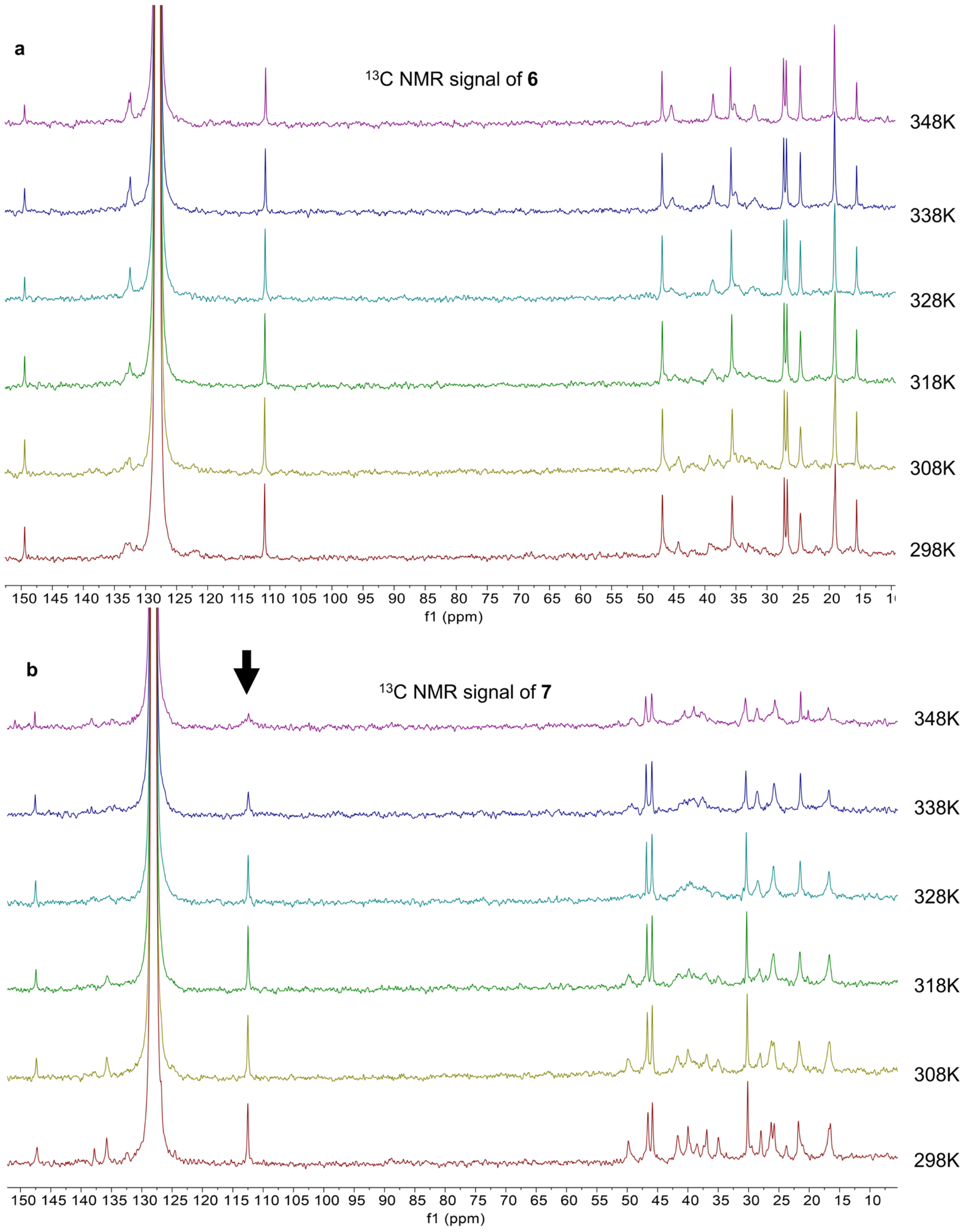

Supplementary Fig. 16. The variable temperature (VT) NMR of compounds 6 and 7. The spectra were recorded under deuterated benzene using the same instrument. (a) VT NMR of compound **6**. (b) VT NMR of compound **7**.

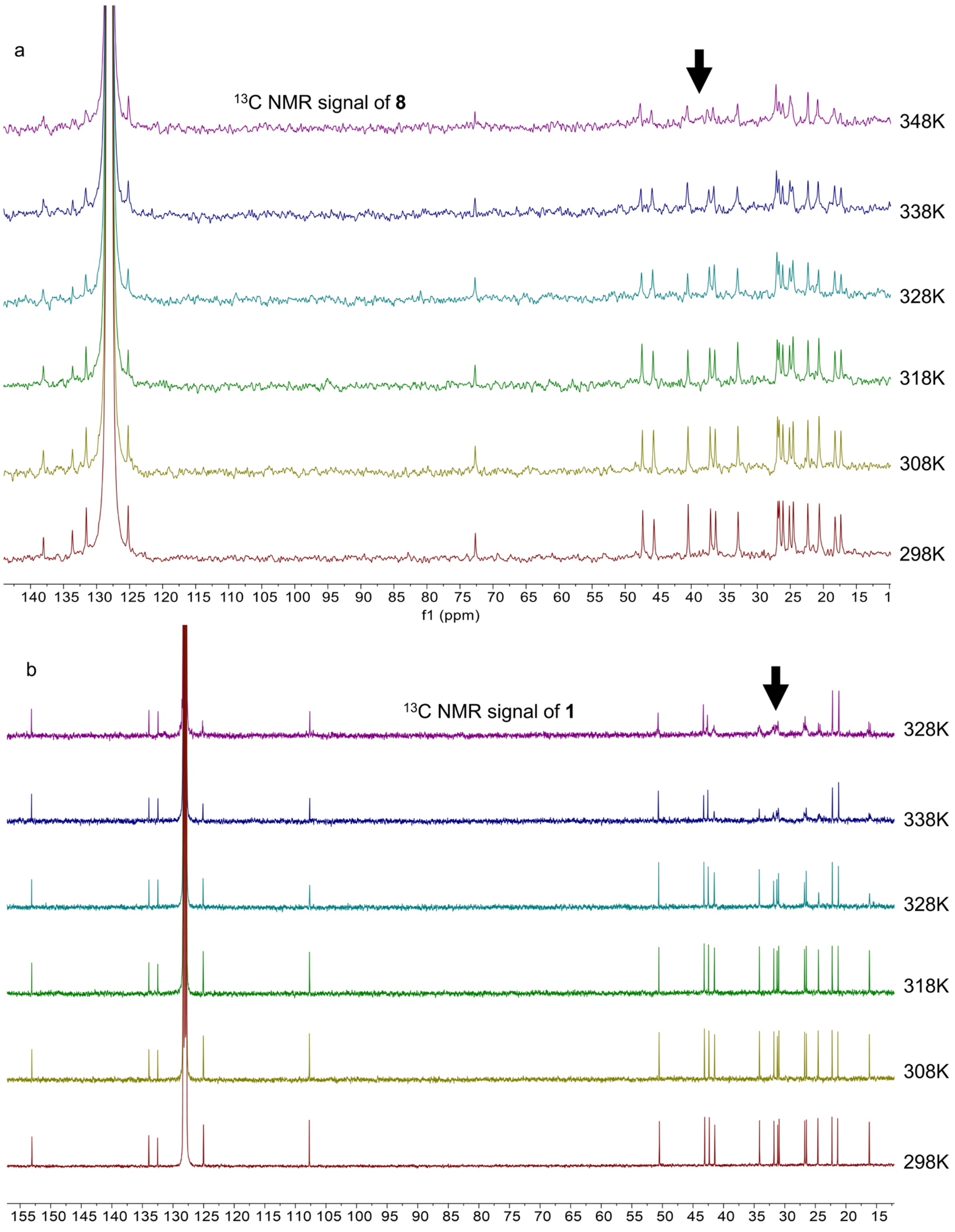

Supplementary Fig. 17. The variable temperature (VT) NMR of compounds 8 and 1. The spectra were recorded under deuterated benzene using the same instrument. (a) VT NMR of compound **8**. (b) VT NMR of compound **1**.

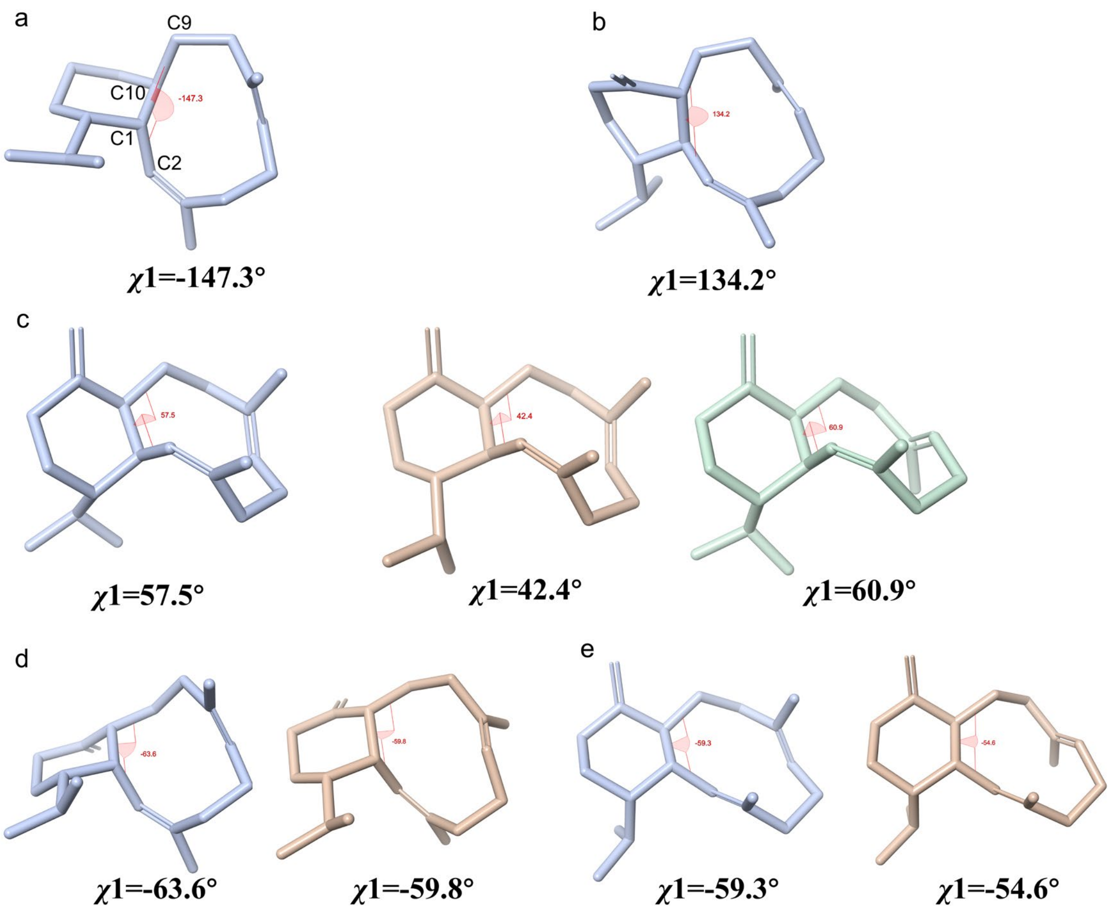

Supplementary Fig. 18. Dihedral angles of conformations of 1, 1*R*,10*R*-1, 1*R*,10*S*-1, 1*S*,10*R*-1 and 2*E*-1. (a) Dihedral angle of conformation **1**_1. (b) Dihedral angle of conformation **1*R*,10*R*-1**_1. (c) Dihedral angles of conformations of **1*R*,10*S*-1**. (d) Dihedral angles of conformations of **1*S*,10*R*-1**. (e) Dihedral angles of conformations of **2*E*-1**.

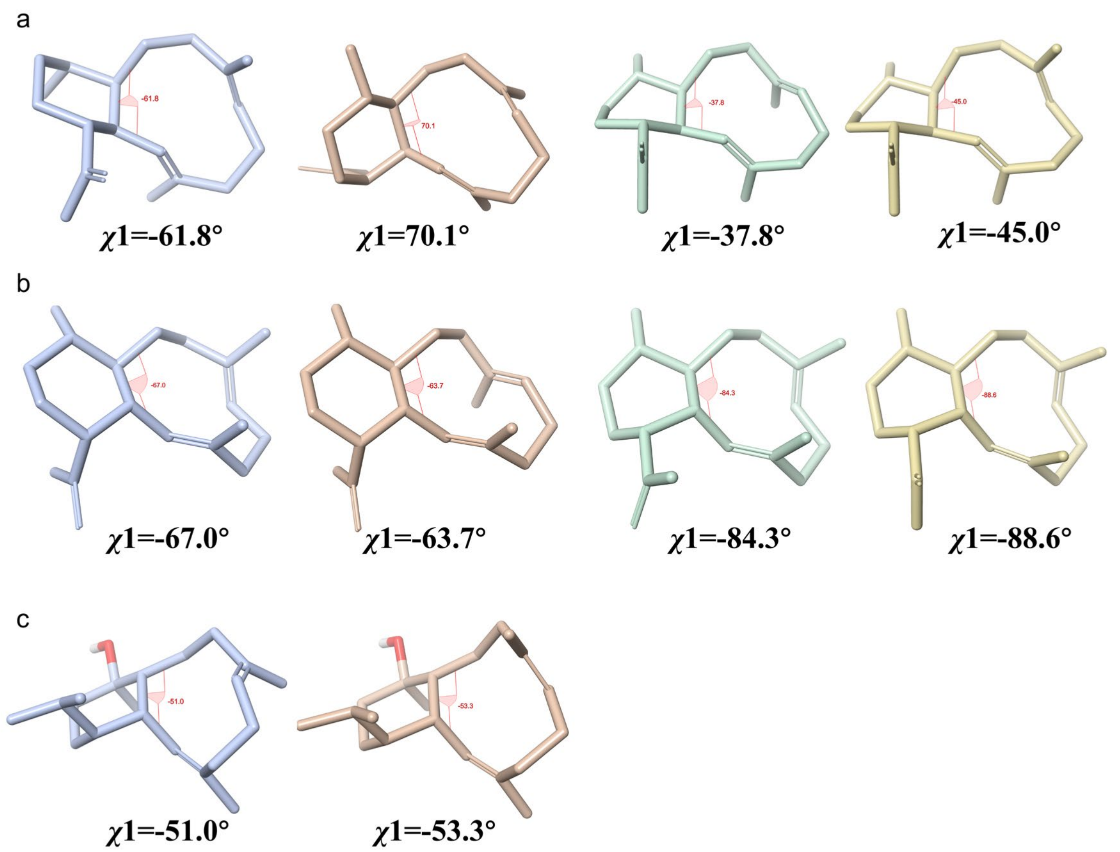

Supplementary Fig. 19. Dihedral angles of conformations of 6–8. (a) Dihedral angles of conformations of **6**. (b) Dihedral angles of conformations of **7**. (c) Dihedral angles of conformations of **8**.

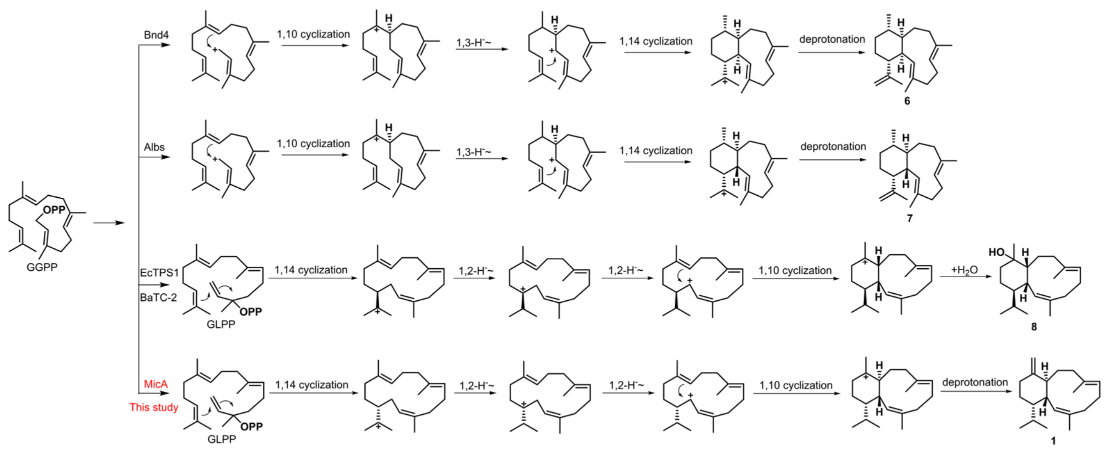

Supplementary Fig. 20. Mechanism on the terpene synthases (TSs) involved in eunicellane biosynthesis. To date, only four eunicellane terpene synthases have been documented, namely Bnd4, AlbS, *Ec*TPS1, and *Ba*TC-2 (*refs.* 6, 7, 28–30). Highlighted in red is the subject of this study.

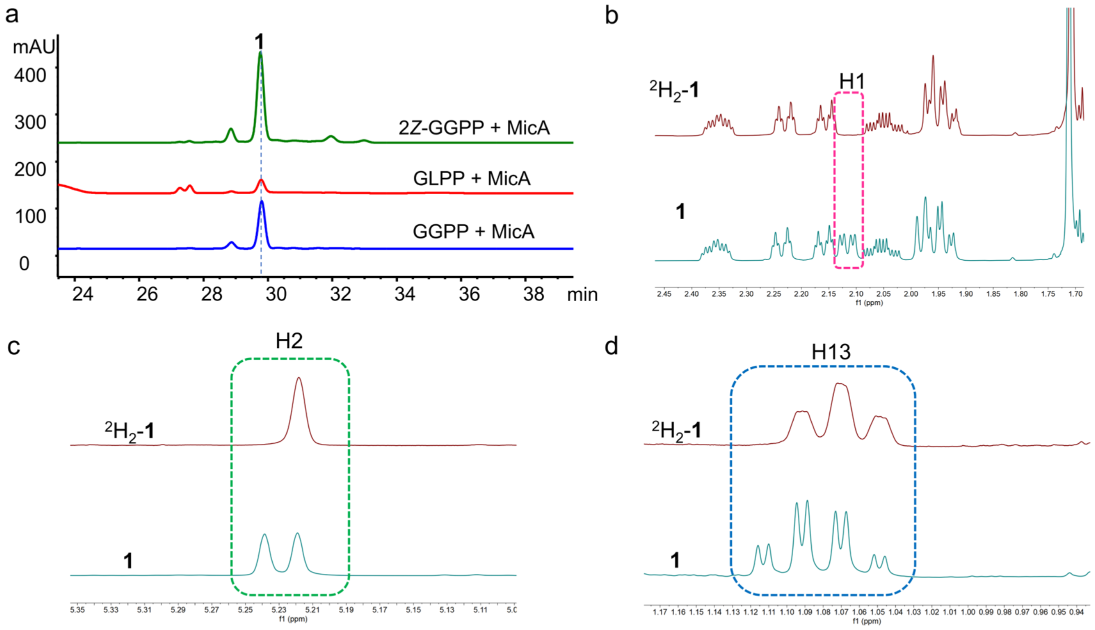

Supplementary Fig. 21. Catalytic route validation by in vitro reactions with hypothesized intermediates and deuterated substrate. (a) Incubation of purified MicA with GLPP and 2*Z*-GGPP yielded **1**, demonstrating the generation of GLPP and 2*Z*-GGPP during the cyclization process. (b–d) ^{1}H NMR spectra of **1** and ($^2H_2$)-**1** (600 MHz, $CDCl_3$). ^{1}H NMR of ($^2H_2$)-**1** closely resembled that of **1**, except for the absence of signals for H-1 (δ_H 2.12) and H-14 (δ_H 1.21) (Supplementary Fig.s 39 and 79). H-2 displays a singlet rather than a respective doublet pattern in **1**. H-13 exhibits a triplet of doublets splitting pattern instead of a quartet of doublets pattern in **1**.

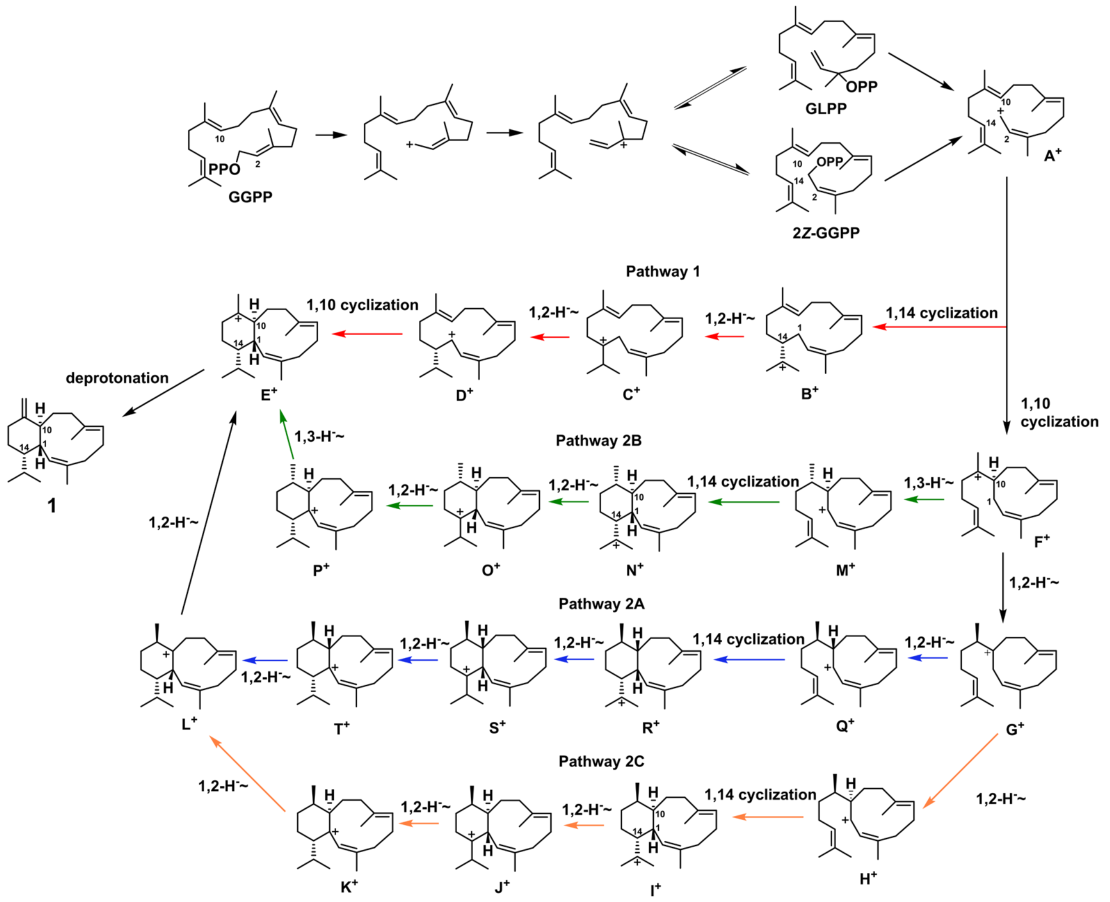

Supplementary Fig. 22. Proposed catalytic pathways for forming the 6,10-bicyclic eunicellane skeleton of 1. Four pathways were proposed, the red arrows denotes pathway 1 ($A^+ \rightarrow B^+ \rightarrow C^+ \rightarrow D^+ \rightarrow E^+$), the blue arrows signifies pathway 2A ($A^+ \rightarrow F^+ \rightarrow G^+ \rightarrow Q^+ \rightarrow R^+ \rightarrow S^+ \rightarrow T^+ \rightarrow L^+ \rightarrow E^+$), the green arrows represents pathway 2B ($A^+ \rightarrow F^+ \rightarrow M^+ \rightarrow N^+ \rightarrow O^+ \rightarrow P^+ \rightarrow E^+$), and the orange arrows corresponds to pathway 2C ($A^+ \rightarrow F^+ \rightarrow G^+ \rightarrow H^+ \rightarrow I^+ \rightarrow J^+ \rightarrow K^+ \rightarrow L^+ \rightarrow E^+$).

a

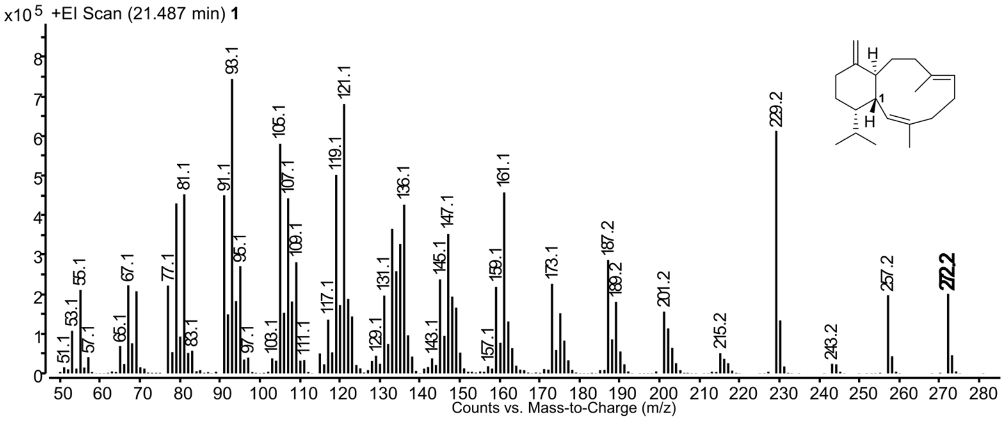

b

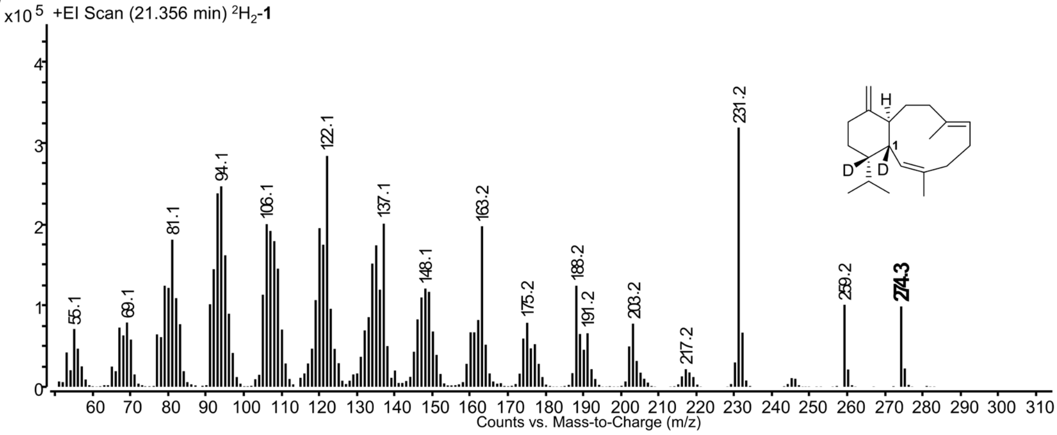

Supplementary Fig. 23. The GC-MS spectra of 1 and ($^2H_2$)-1. (a) The GC-MS spectra of **1**. (b) The GC-MS spectra of ($^2H_2$)-**1**. The M^+ ion and fragmentation ions that show clear differences of *m/z* +2 between **1** and ($^2H_2$)-**1** were shown in bold.

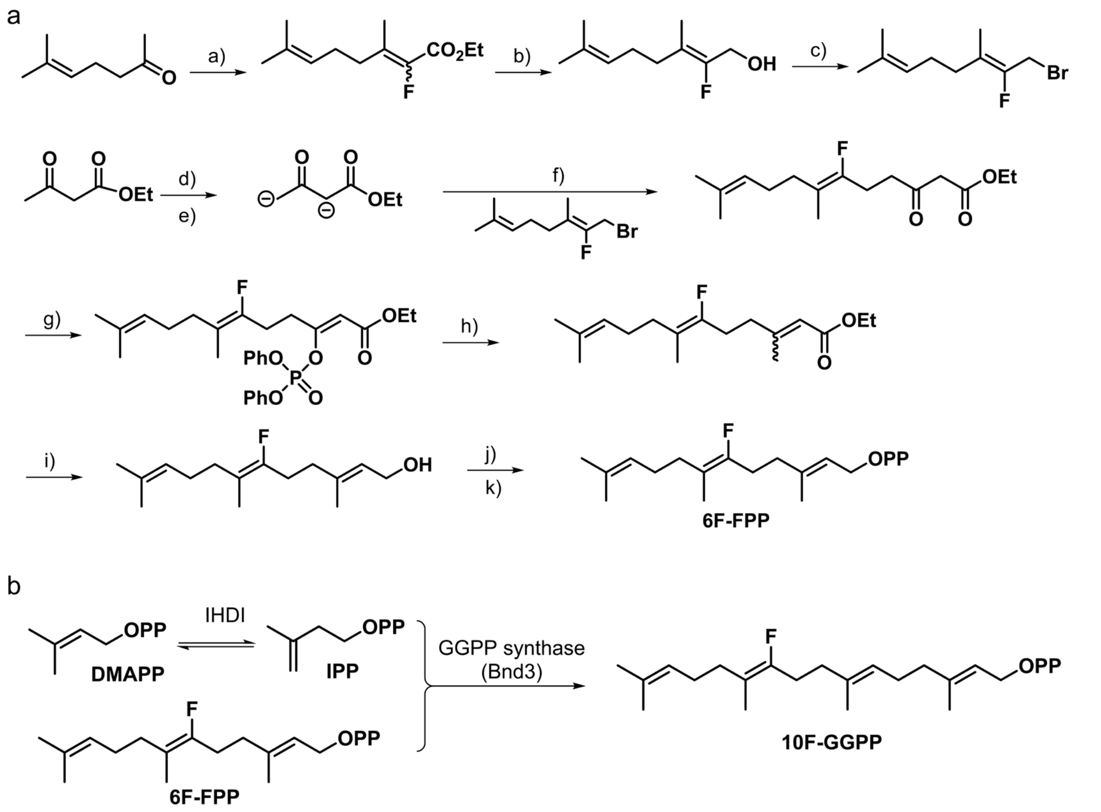

Supplementary Fig. 24. In situ biosynthesis of 10F-GGPP. (a) Synthesis of 6F-GGPP. (b) Synthesis of 10F-GGPP. Reaction conditions: a) $(EtO)_2P(O)CHFCO_2Et$, NaH, THF, 0 °C, 3.5 h; b) $LiAlH_4$, THF, rt, 2 h; c) PPh_3, CBr_4, DCM, rt, 2.5 h; d) NaH, THF, 0 °C, 10 min; e); n-BuLi, 0 °C, 10 min; f) 0 °C, 15 min; g) NaH, Et_2O, $(PhO)_2P(O)Cl$, 0 °C, 15 min; h) Me_2CuLi, Et_2O, -78 °C, 3.5 h; i) DIBAL-H, Et_2O, 0 °C, 2 h; j) CCl_4, PPh_3, 84 °C, 12 h; k) $(Bu_4N)_3P_2O_7H$, CH_3CN, rt, 2 h.

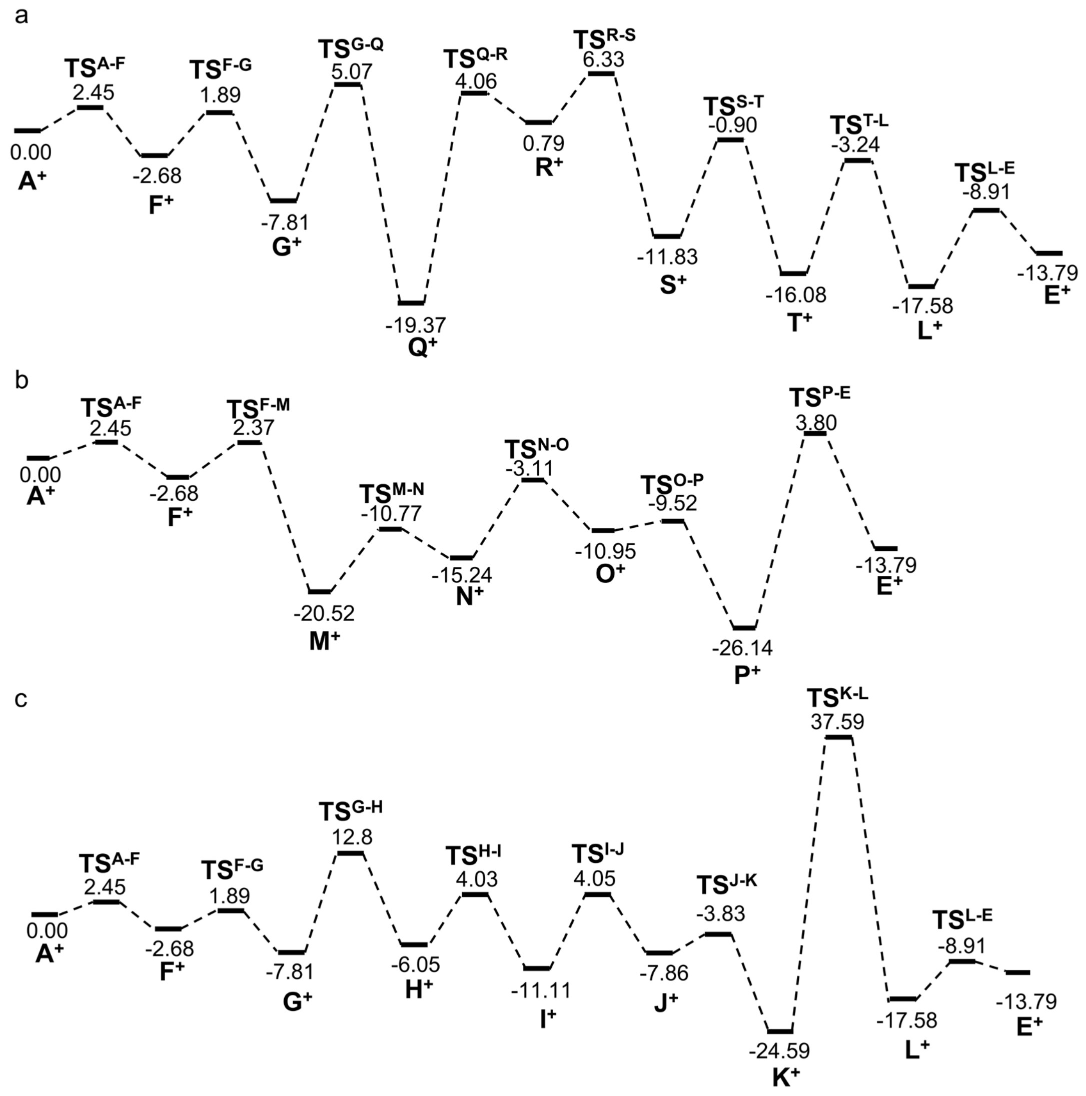

Supplementary Fig. 25. Computed energetics for pathway 2. Relative free energies of intermediates and transition state structures in kcal/mol, calculated with DFT calculations utilizing the mPW1PW91/6-31+G(d,p)//B3LYP/6-31+G(d,p) method. (a) Pathway 2A. (b) Pathway 2B. (c) Pathway 2C.

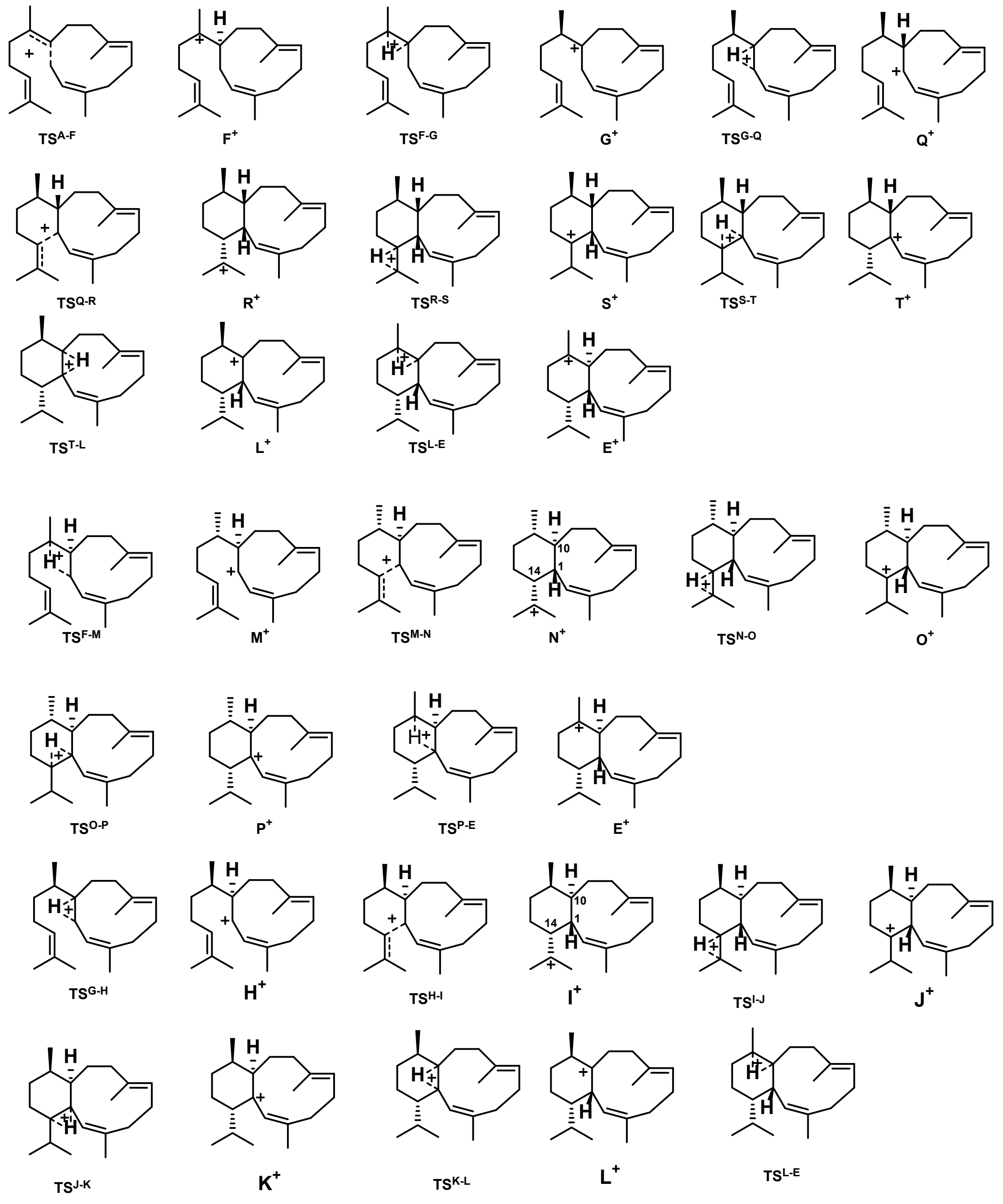

Supplementary Fig. 26. Intermediates and transition state structures of pathway 2. In the given context, F^+ denotes the intermediate stage, and $TS^{A\text{-}F}$ signifies the transition state. This convention is sustained for the subsequent stages as well.

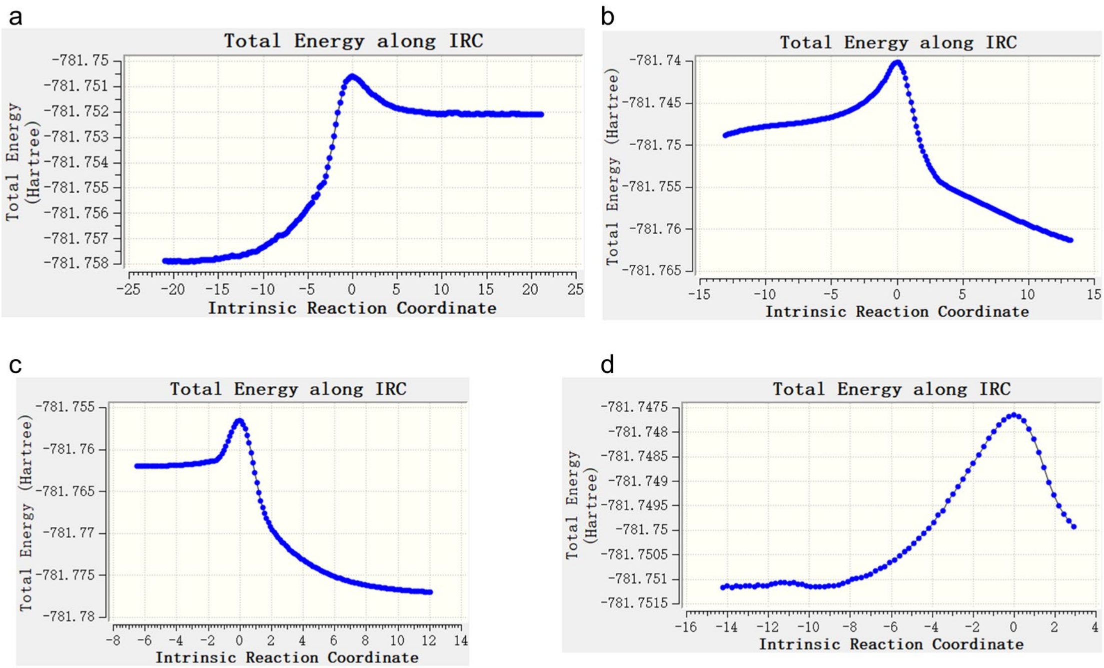

Supplementary Fig. 27. The IRC path in pathway 1. (a) The IRC path of $TS^{A\text{-}B}$. (b) The IRC path of $TS^{B\text{-}C}$. (c) The IRC path of $TS^{C\text{-}D}$. (d) The IRC path of $TS^{D\text{-}E}$.

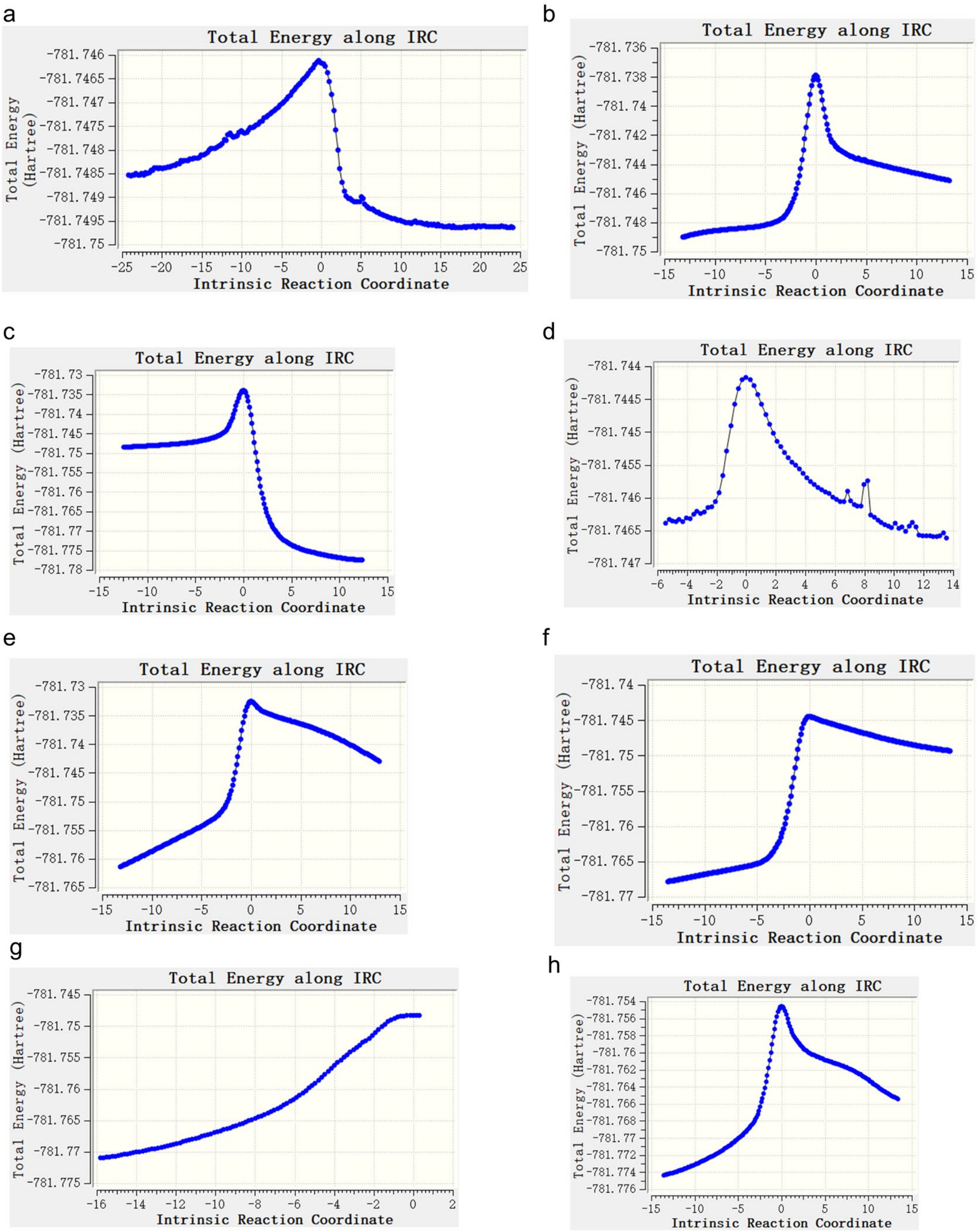

Supplementary Fig. 28. The IRC path in pathway 2A. (a) The IRC path of $TS^{A\text{-}F}$. (b) The IRC path of $TS^{F\text{-}G}$. (c) The IRC path of $TS^{G\text{-}Q}$. (d) The IRC path of $TS^{Q\text{-}R}$. (e) The IRC path of $TS^{R\text{-}S}$. (f) The IRC path of $TS^{S\text{-}T}$. (g) The IRC path of $TS^{T\text{-}L}$. (h) The IRC path of $TS^{L\text{-}E}$.

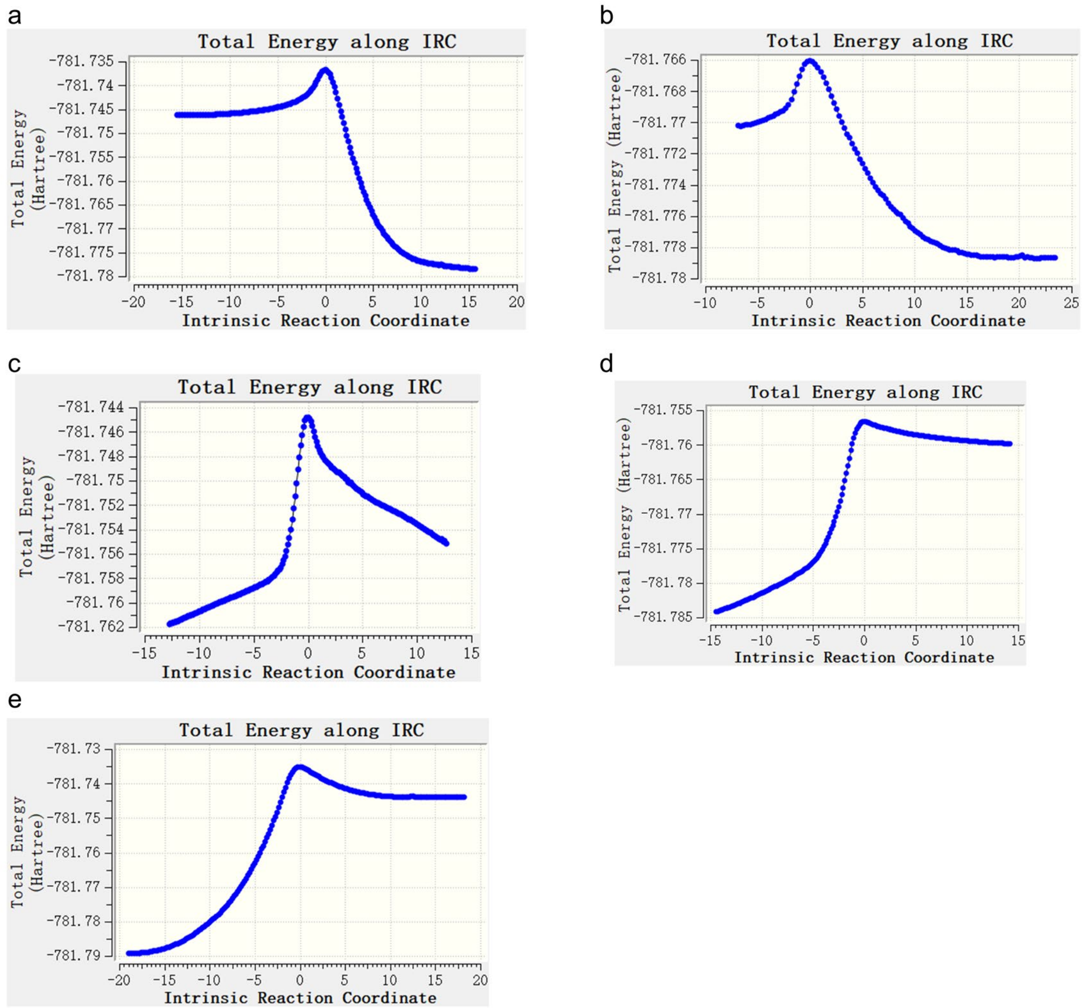

Supplementary Fig. 29. The IRC path in pathway 2B. (a) The IRC path of $TS^{F\text{-}M}$. (b) The IRC path of $TS^{M\text{-}N}$. (c) The IRC path of $TS^{N\text{-}O}$. (d) The IRC path of $TS^{O\text{-}P}$. (e) The IRC path of $TS^{P\text{-}E}$.

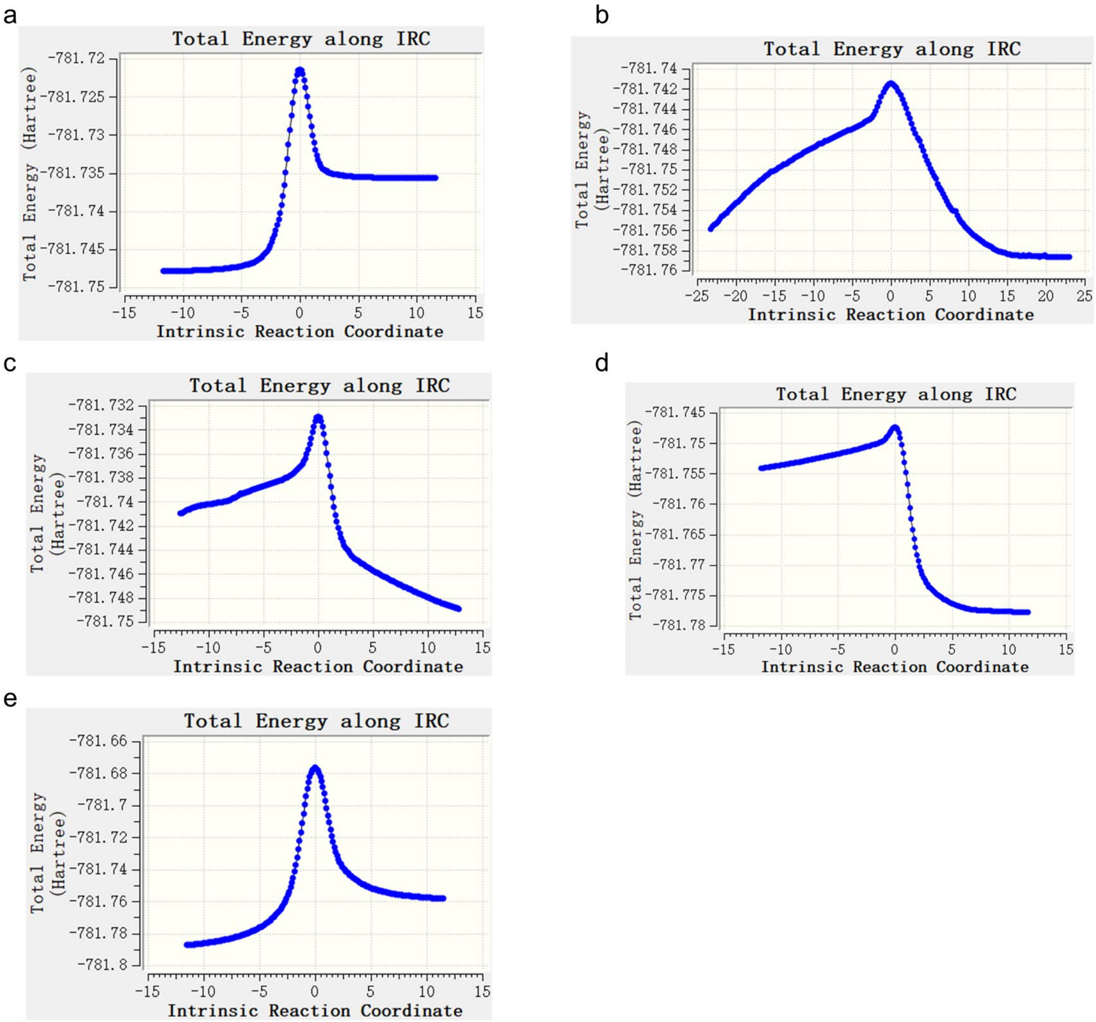

Supplementary Fig. 30. The IRC path in pathway 2C. (a) The IRC path of $TS^{G\text{-}H}$. (b) The IRC path of $TS^{H\text{-}I}$. (c) The IRC path of $TS^{I\text{-}J}$. (d) The IRC path of $TS^{J\text{-}K}$. (e) The IRC path of $TS^{K\text{-}L}$.

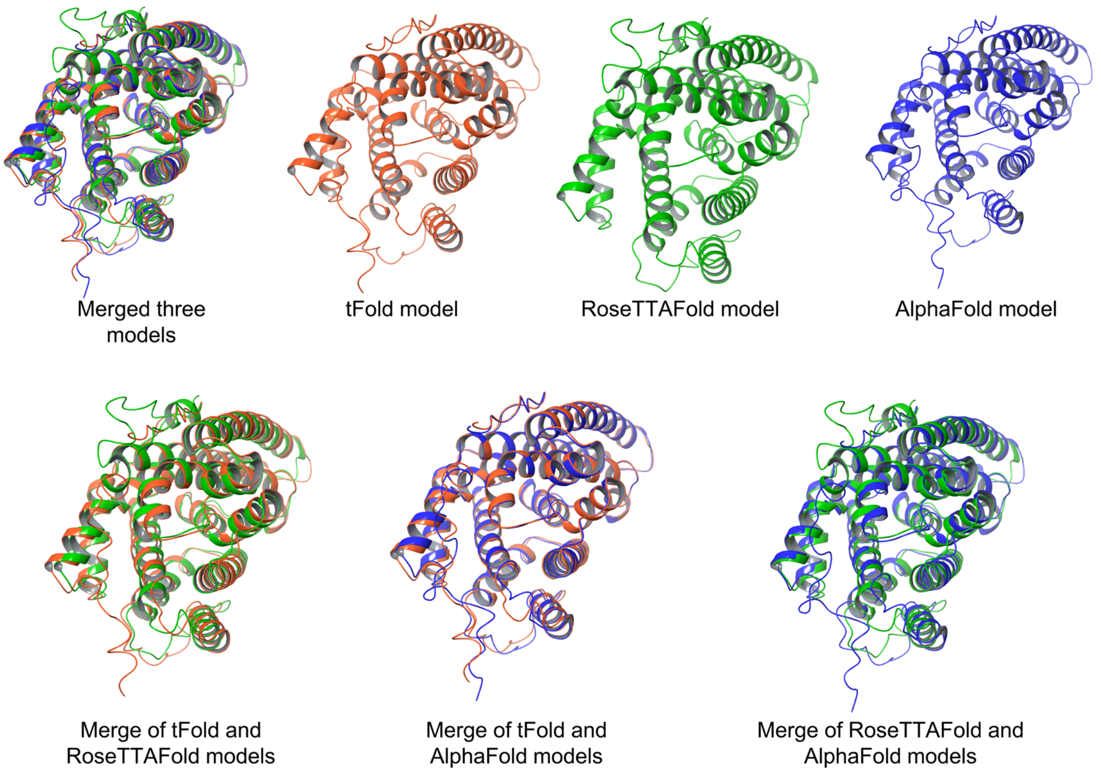

Supplementary Fig. 31. Structural comparisons of computed MicA models. Three de novo predicted models of MicA: the tFold model (red), the RoseTTAFold model (green), and the AlphaFold model (blue), were evaluated and compared for selecting the best one for the downstream analysis. According to the "Protein Model Quality Assessment" system embedded in the tFold platform, the global scores of these models from tFold, RoseTTAFold, and AlphaFold were 0.57, 0.52, and 0.55, respectively (bigger is better), indicating the tFold model is the best one. The RMSD between models from tFold and RoseTTAFold, tFold and AlphaFold, and RoseTTAFold and AlphaFold are 1.30 Å, 1.11 Å, and 1.44 Å, respectively.

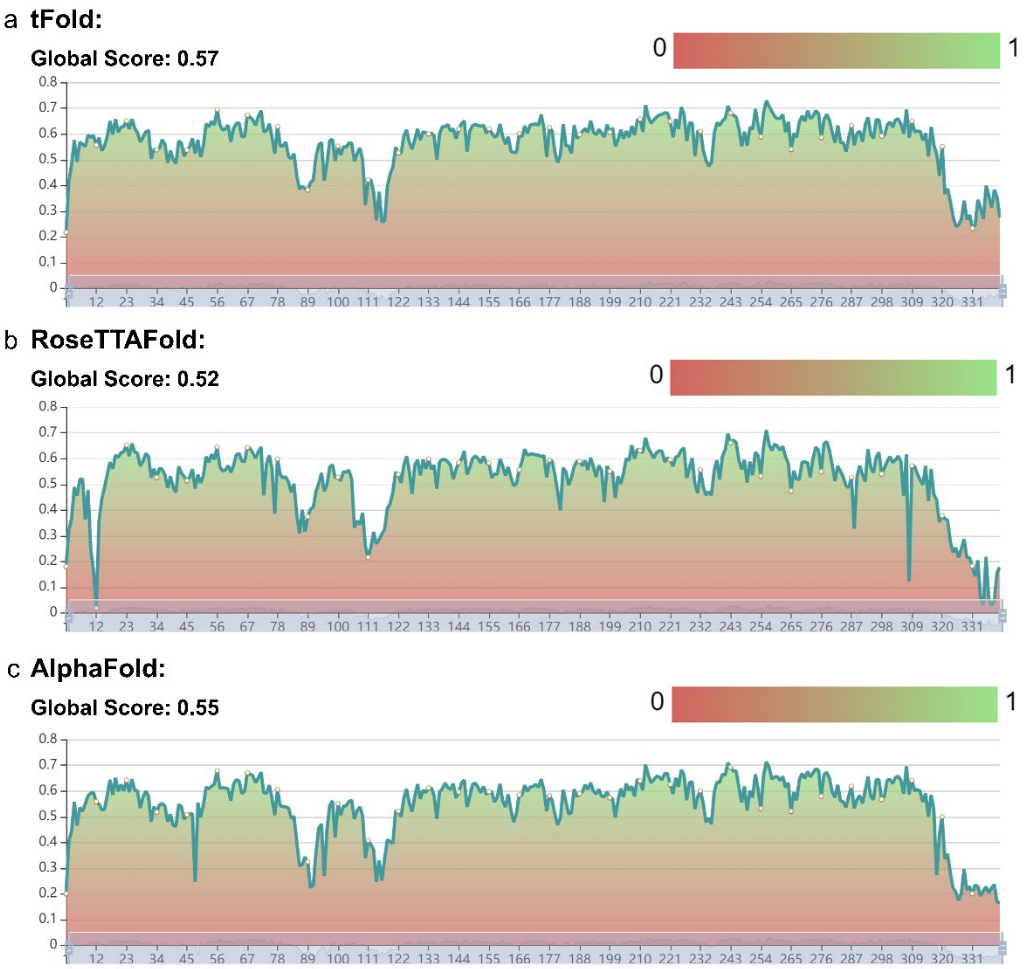

Supplementary Fig. 32. Protein model quality assessment of predicted MicA models. (a) The "Global Score" of predicted MicA model using the tFold[31] computational methods. (b) The "Global Score" of predicted MicA model using the RoseTTAFold[32] computational methods. (c) The "Global Score" of predicted MicA model using the AlphaFold[33] computational methods. The "Protein Model Quality Assessment" system embedded in the tFold platform was used to assess the quality of the MicA models, which were predicted using the tFold, RoseTTAFold, and AlphaFold computational methods. The protein structure model was evaluated using the "Global Score", representing the average score across all residues. A score of zero for a residue indicates its absence in the protein file, affecting the global score. A higher global score is indicative of better quality. In this context, the protein structure model predicted for MicA using tFold obtained the highest score of 0.57, suggesting its superior quality.

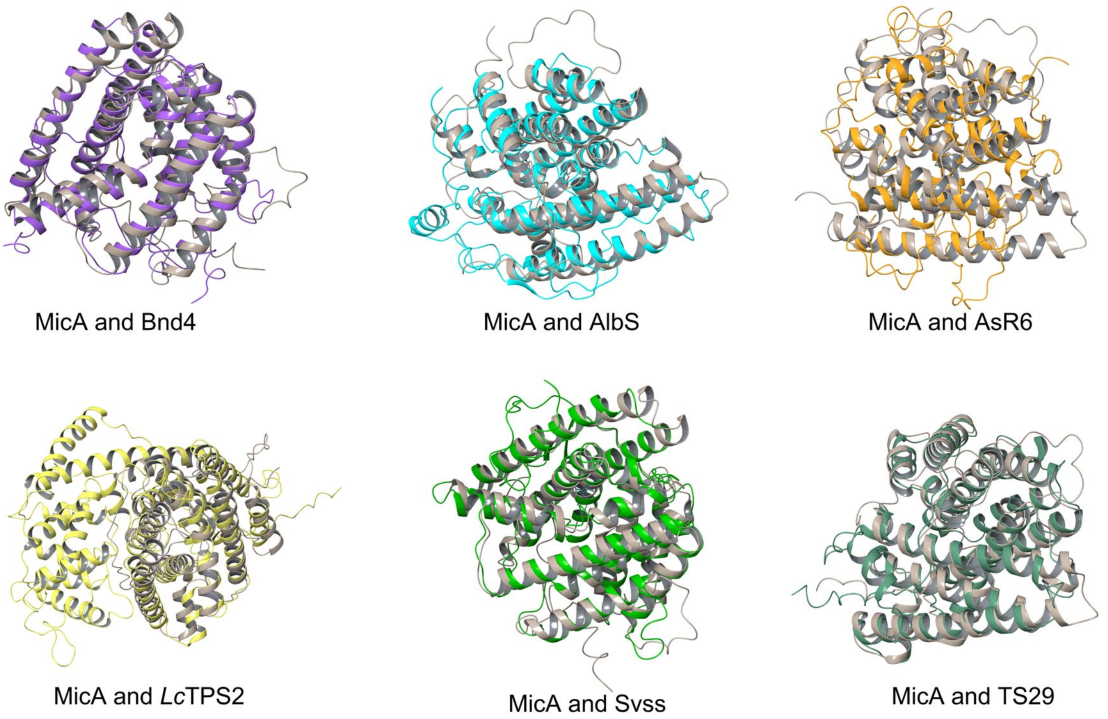

Supplementary Fig. 33. Structural alignments of MicA and other related proteins. The structural alignments of MicA (predicted model) with Bnd4 (predicted model)[6], AlbS (predicted model)[7], α-humulene synthase (AsR6, PDB ID: 7OC5)[35], *Lc*TPS2 (predicted model)[36], SvSS (predicted model)[37], and TS29 (predicted model)[38] resulted in a RMSD of 2.20 Å, 2.35 Å, 3.88 Å, 3.08 Å, 2.35 Å and 1.79 Å, respectively.

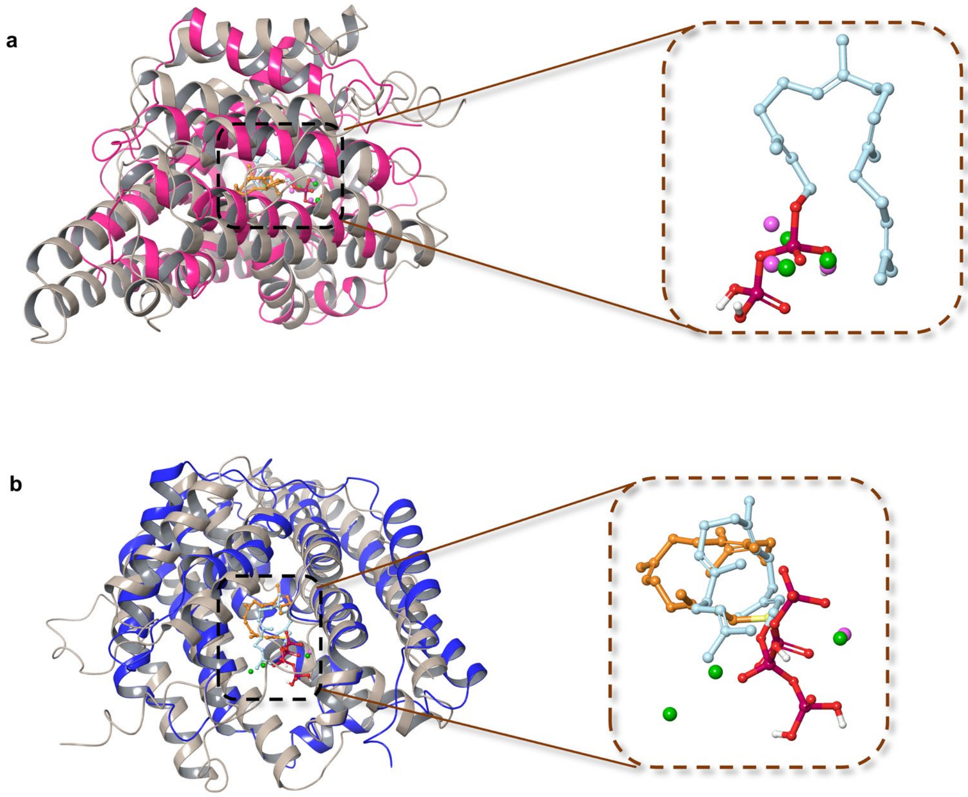

Supplementary Fig. 34. Structural comparisons of MicA docking model and CotB2 crystal structure. (a) Structural alignments of the MicA docking model (grey) and CotB2 crystal structure (6GGI, pink). The ligand containing Mg^{2+} ions (green) originates from MicA docking, while Mg^{2+} ions (pink) is derived from 6GGI. (b) Structural alignments of the MicA docking model and CotB2 crystal structure (5GUE, blue). The ligand featuring Mg^{2+} ions (green) is from MicA docking, while the ligand (orange) is sourced from 5GUE.

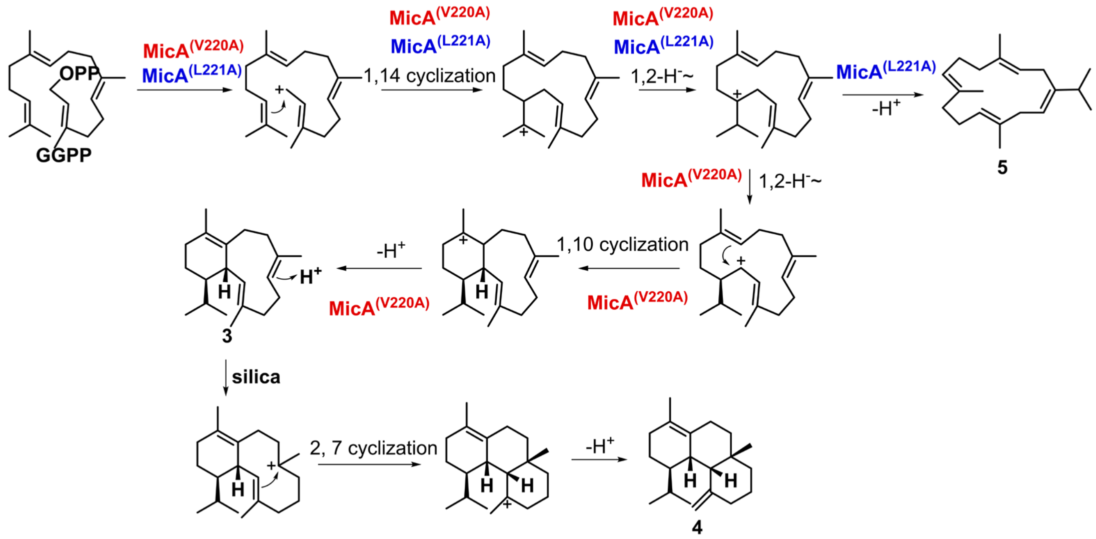

Supplementary Fig. 35. A proposed mechanistic pathway explained the cyclization of GGPP to form compounds 3–5 among two MicA.

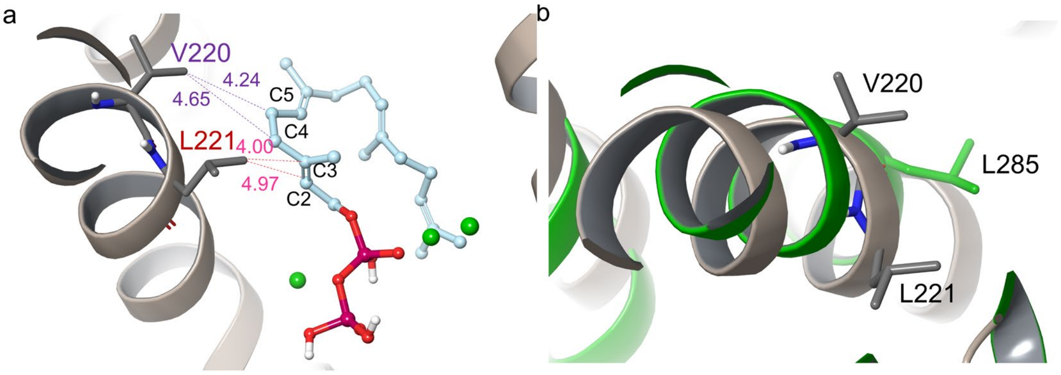

Supplementary Fig. 36. Overview of V220 and L221 in MicA and their corresponding residue L285 in AsR6. (a) V220 and L221 were selectively shown in the docking model of MicA-Mg^{2+}-GGPP. Distances of V220 to C4 and C5, and L221 to C2 and C3 were measured and shown in Å. (b) Structural alignment of the MicA model and AsR6 structure (PDB ID: 7OC5) with L285 (green) in AsR6 and V220 (grey) together with L221 (grey) in MicA were shown.

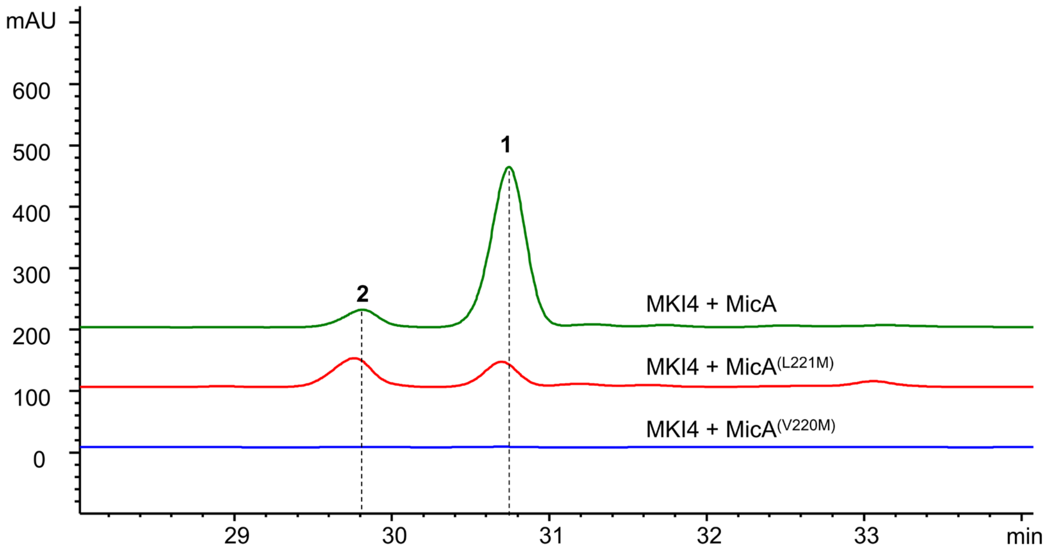

Supplementary Fig. 37. HPLC analyses (210 nm) of the mutants MicA $^{(V220M)}$ and MicA $^{(L221M)}$ in comparison with native MicA. The mutations led to primary product reduction (L221M) and elimination (V220M).

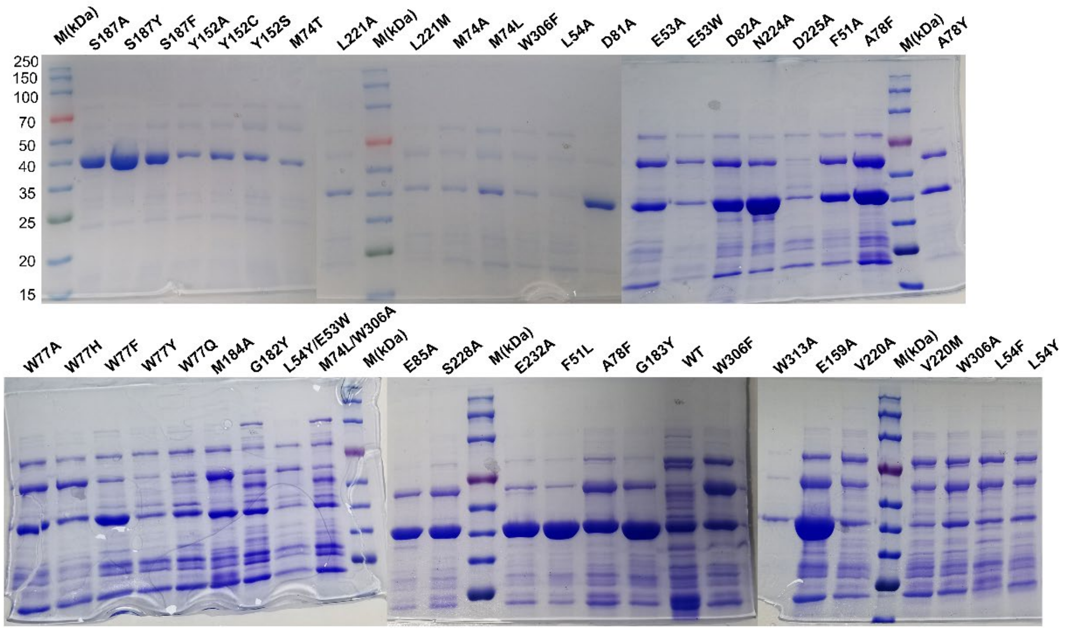

Supplementary Fig. 38. SDS-PAGE of purified MicA and mutants. The MicA has a size of 39.2 kDa. Both MicA and its mutants are soluble. However, the mutants have varying effects on protein yield. Specifically, mutants MicA $^{(D225A)}$, MicA $^{(W313A)}$, MicA $^{(L54A)}$, and MicA $^{(L54Y/E53W)}$ exhibit a significant reduction in protein yield. Source data are provided as a Source Data file.

Original spectra for compound 1 in $CDCl_3$.

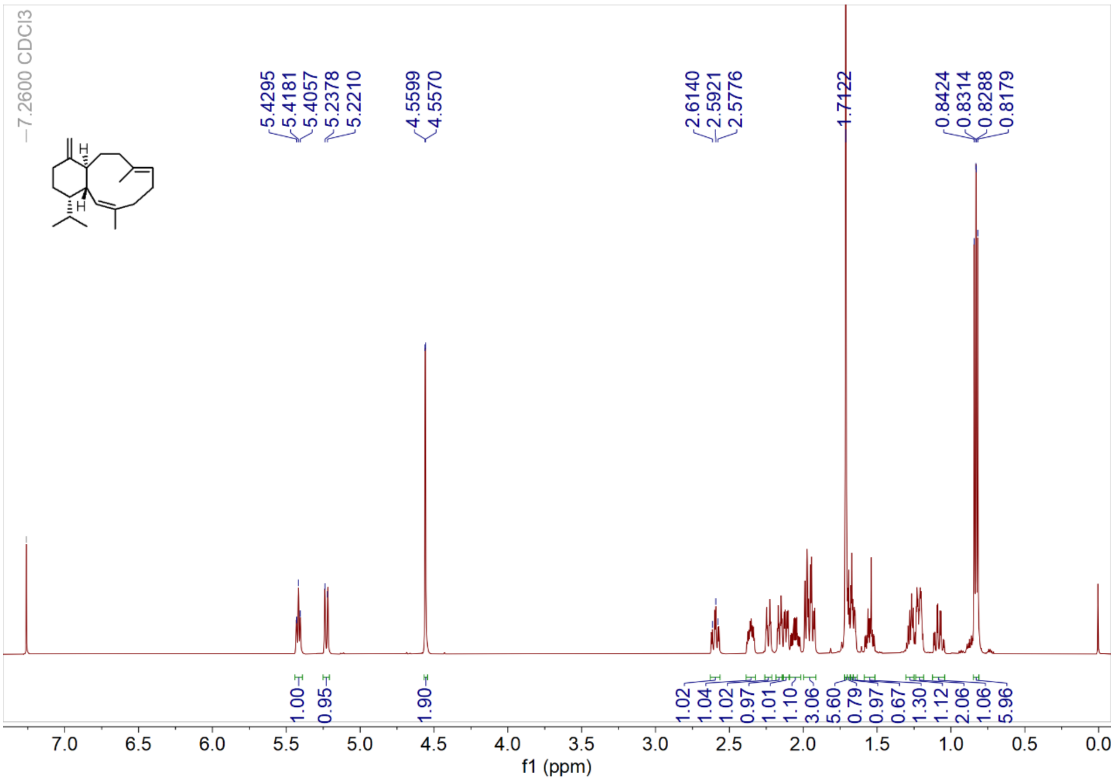

Supplementary Fig. 39. ^{1}H NMR spectrum (600 MHz) of **1** in $CDCl_3$.

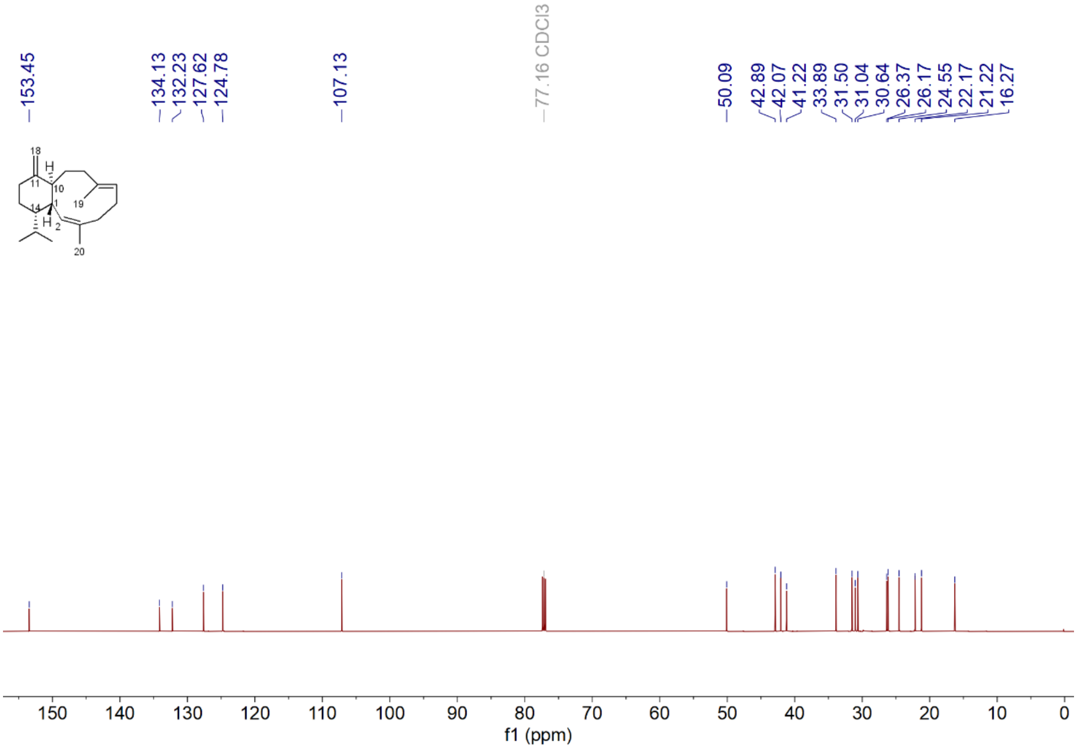

Supplementary Fig. 40. ^{13}C NMR spectrum (150 MHz) of **1** in $CDCl_3$.

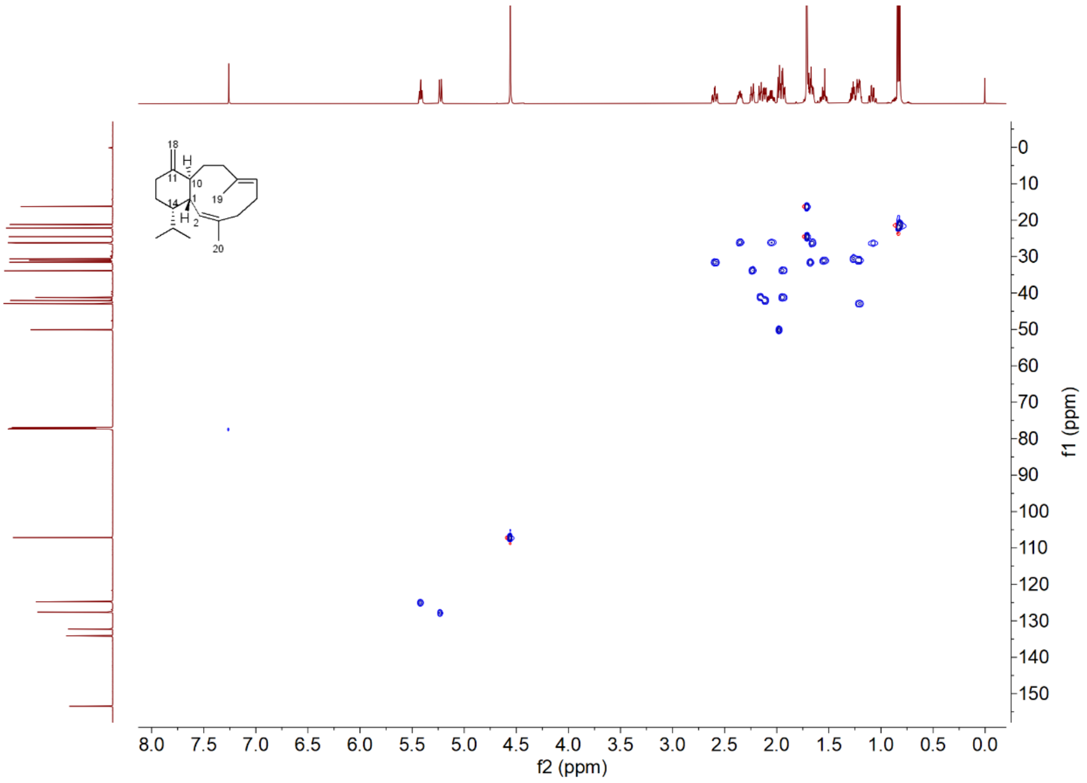

Supplementary Fig. 41. HSQC NMR spectrum of **1** in $CDCl_3$.

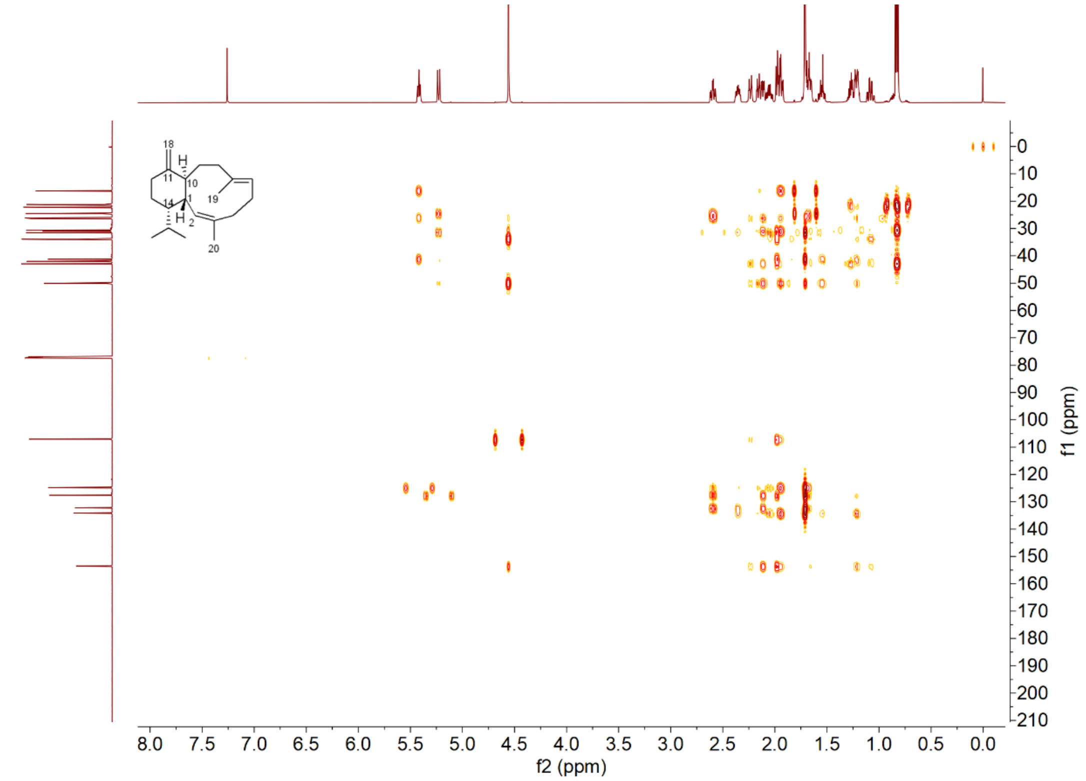

Supplementary Fig. 42. HMBC NMR spectrum of **1** in $CDCl_3$.

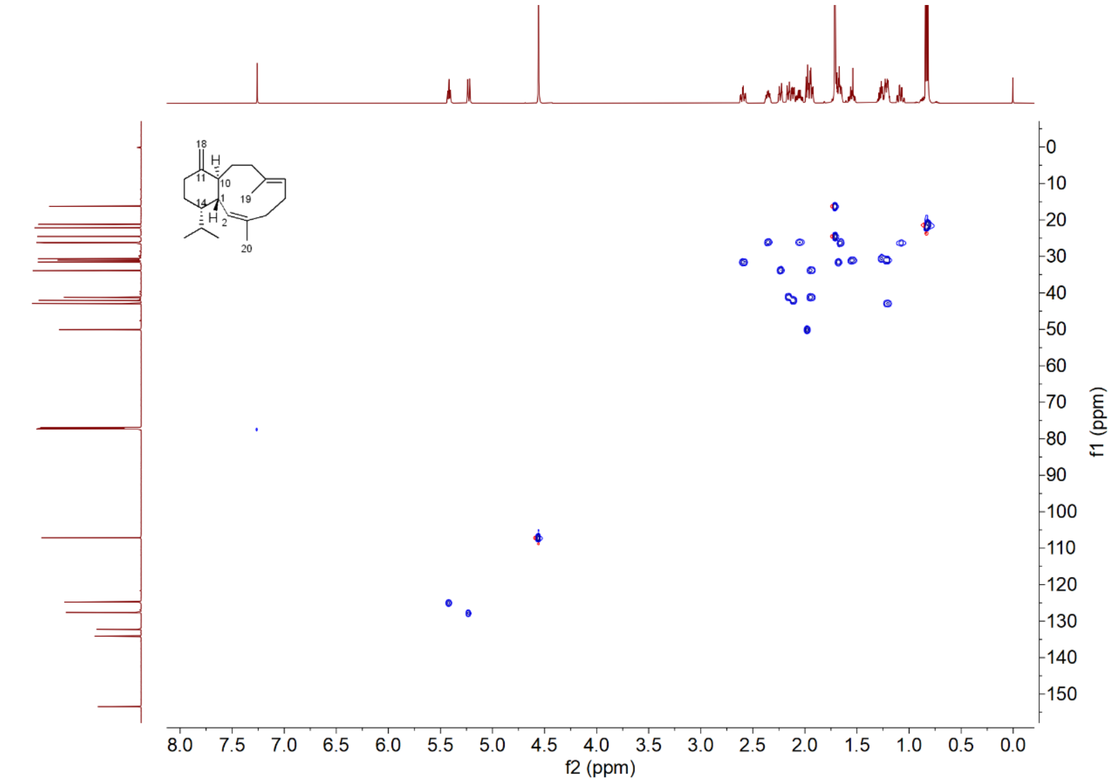

Supplementary Fig. 43. ^{1}H-^{1}H COSY NMR spectrum of **1** in $CDCl_3$.

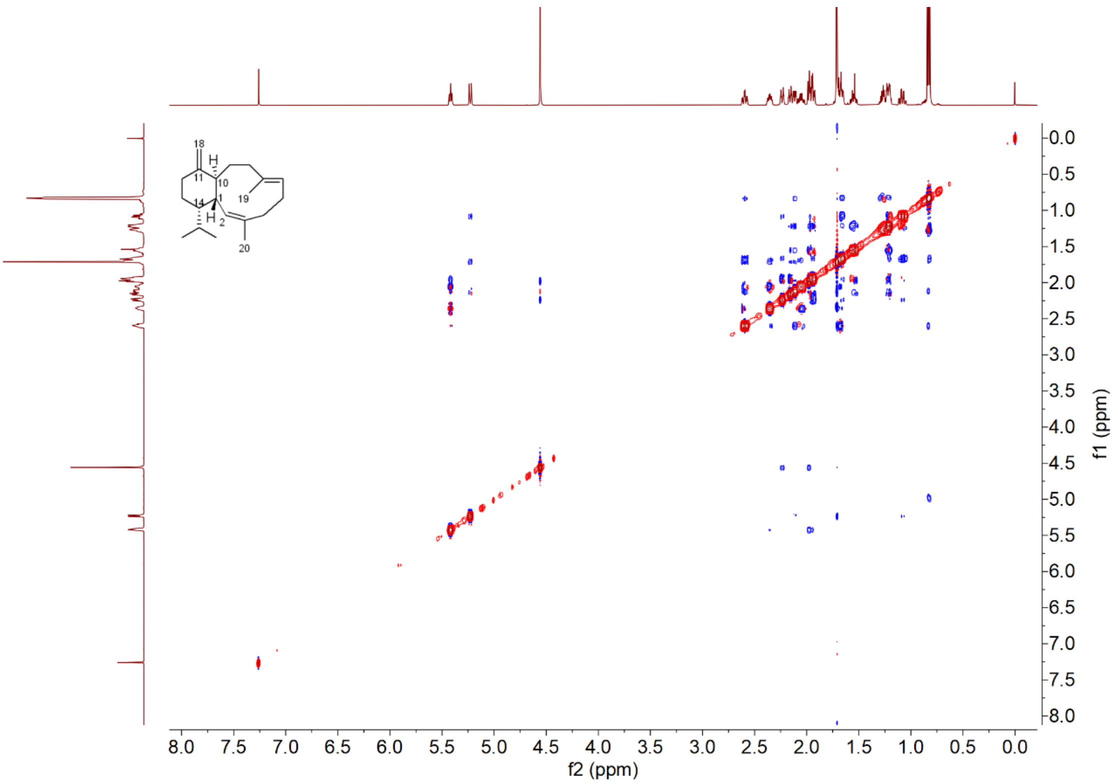

Supplementary Fig. 44. NOESY NMR spectrum of **1** in $CDCl_3$.

Original spectra for compound 2.

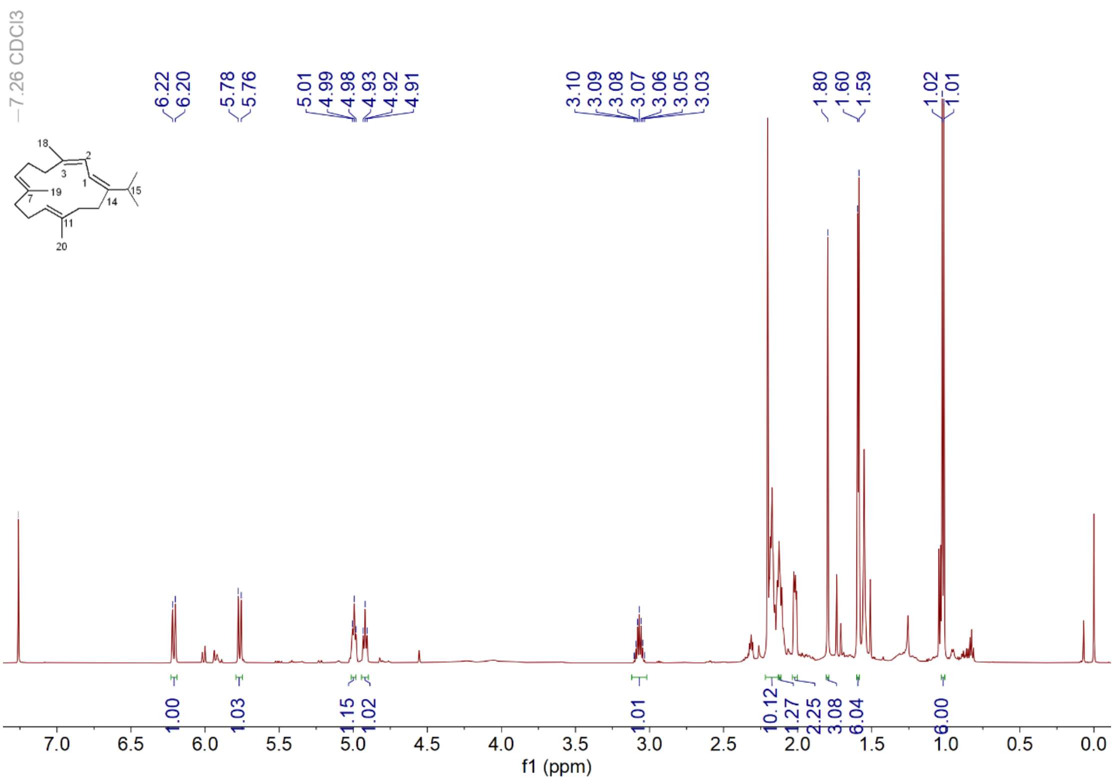

Supplementary Fig. 45. ^{1}H NMR spectrum (600 MHz) of **2** in $CDCl_3$.

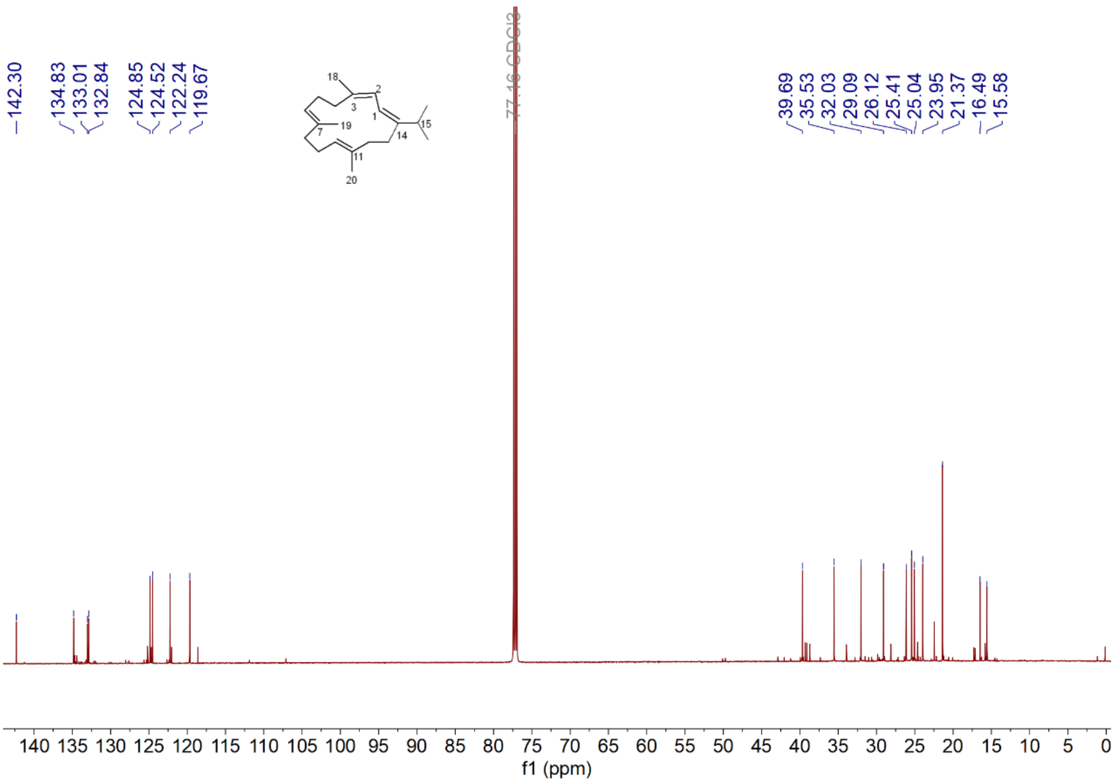

Supplementary Fig. 46. ^{13}C NMR spectrum (150 MHz) of **2** in $CDCl_3$.

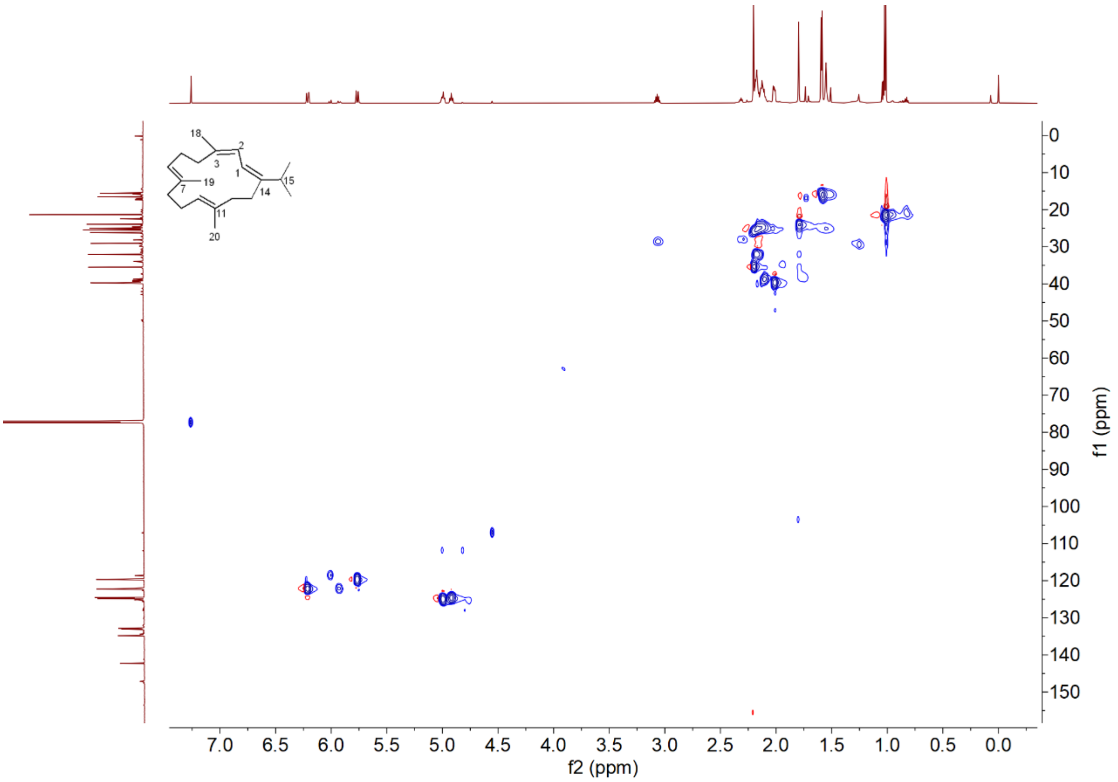

Supplementary Fig. 47. HSQC NMR spectrum of **2** in $CDCl_3$.

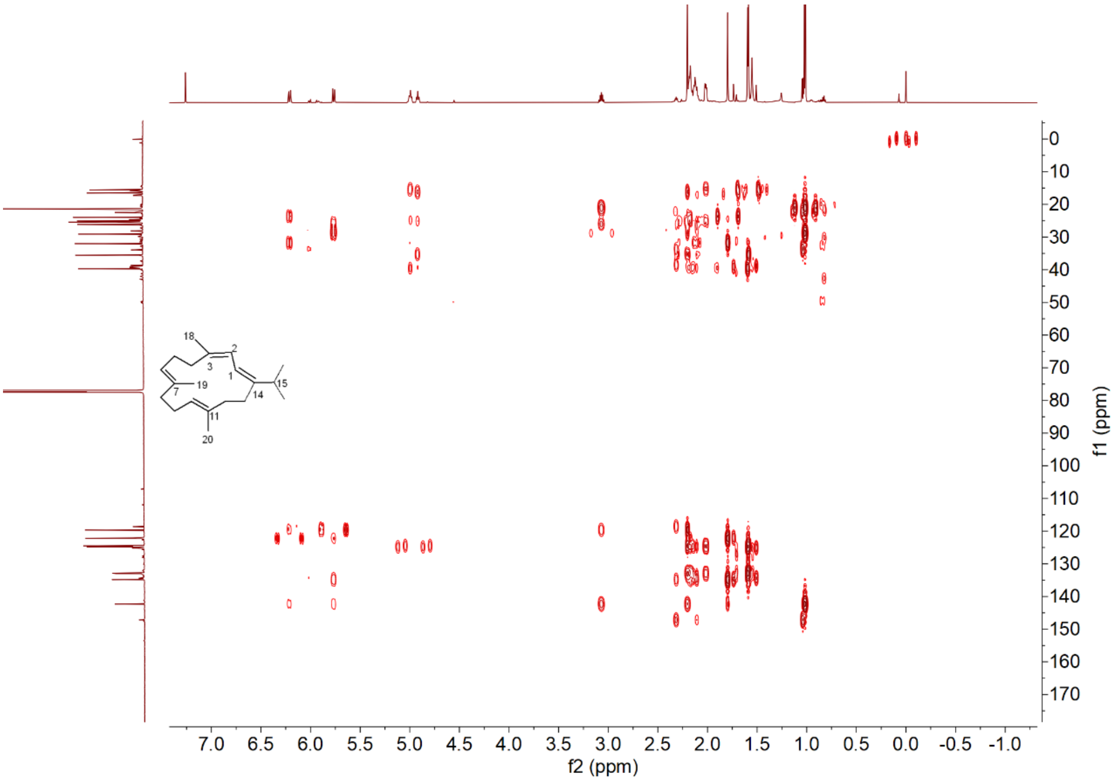

Supplementary Fig. 48. HMBC NMR spectrum of **2** in $CDCl_3$.

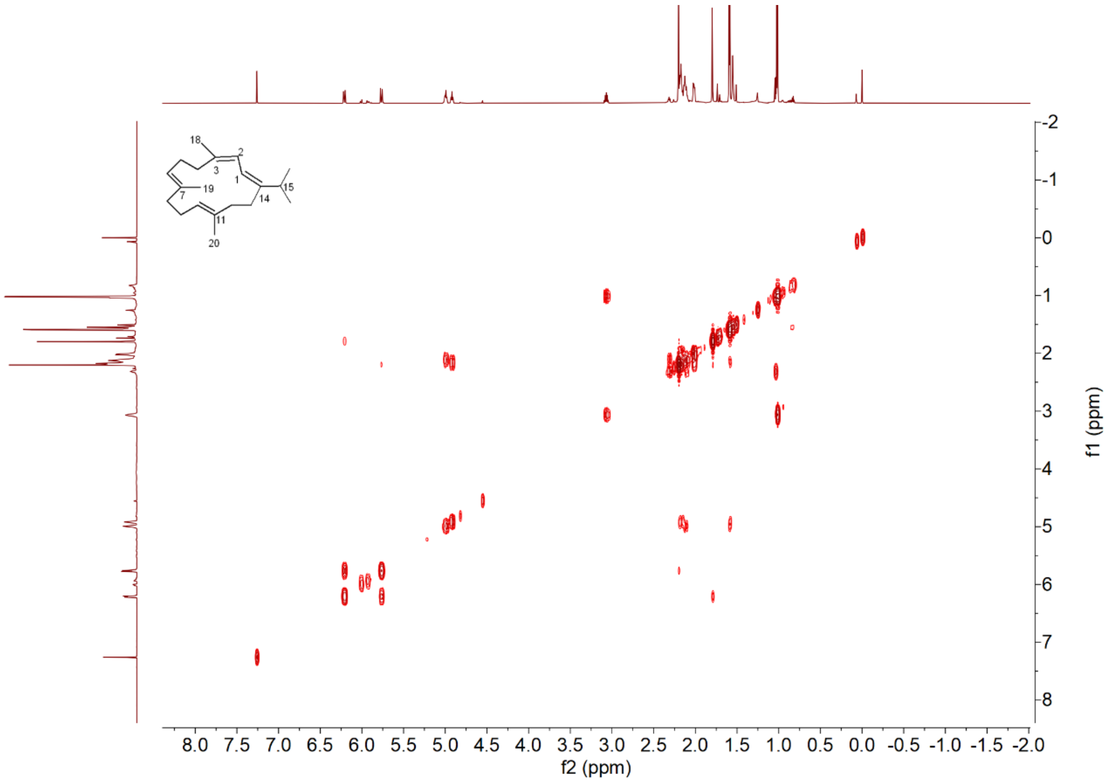

Supplementary Fig. 49. ^{1}H-^{1}H COSY NMR spectrum of **2** in $CDCl_3$.

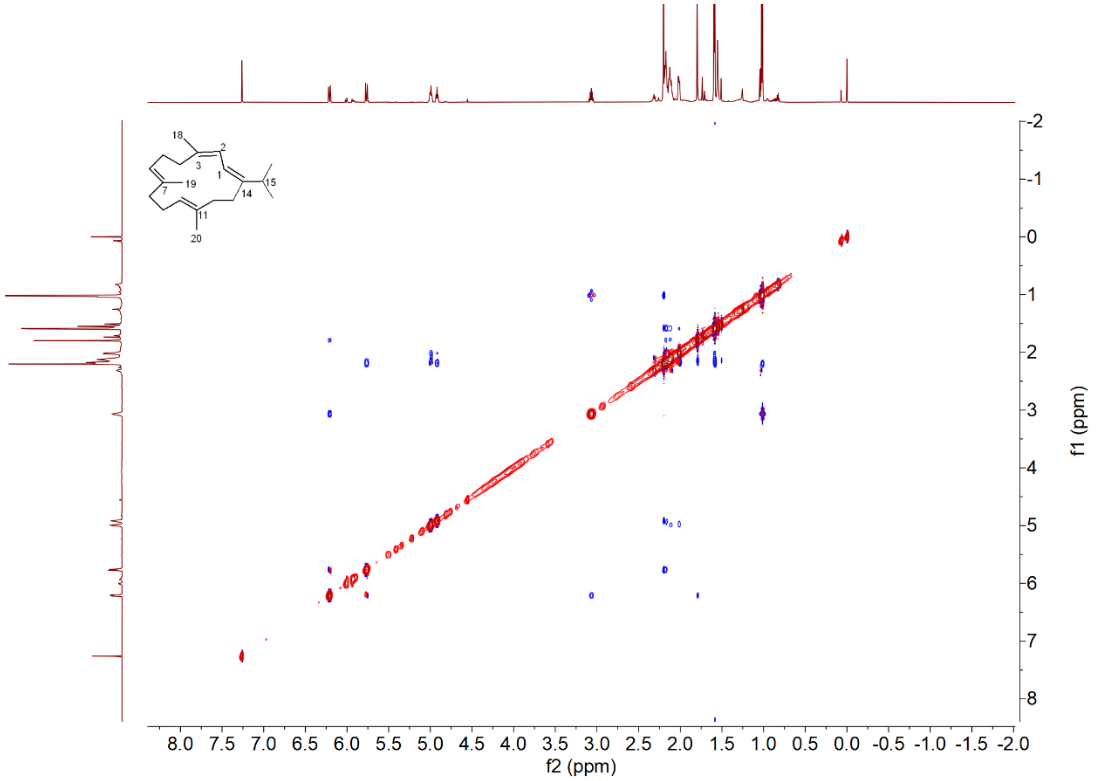

Supplementary Fig. 50. NOESY NMR spectrum of **2** in $CDCl_3$.

Original spectra for compound 1a.

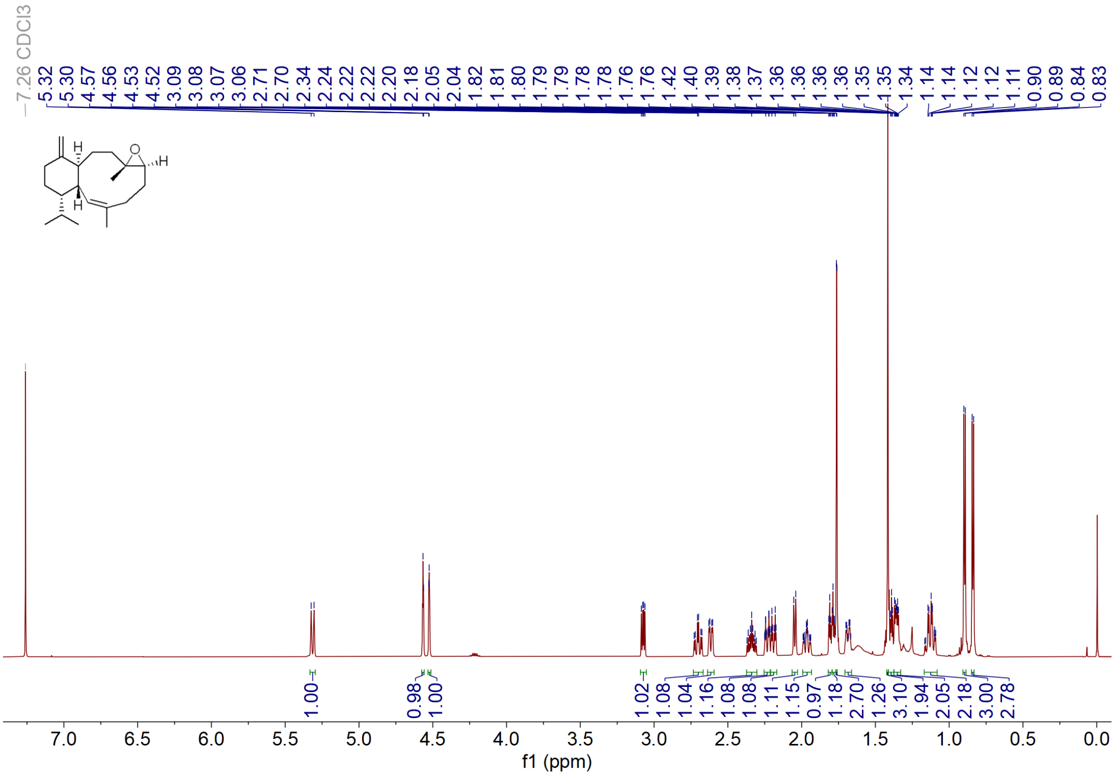

Supplementary Fig. 51. ^{1}H NMR spectrum (600 MHz) of **1a** in $CDCl_3$.

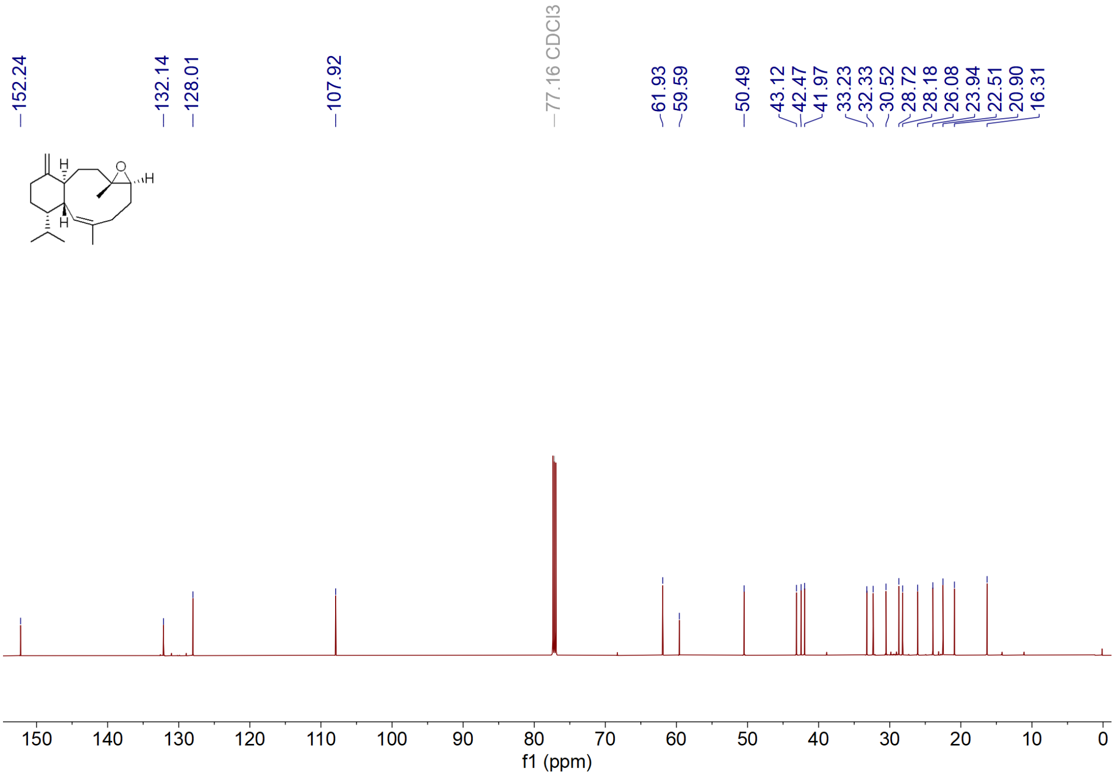

Supplementary Fig. 52. ^{13}C NMR spectrum (150 MHz) of **1a** in $CDCl_3$.

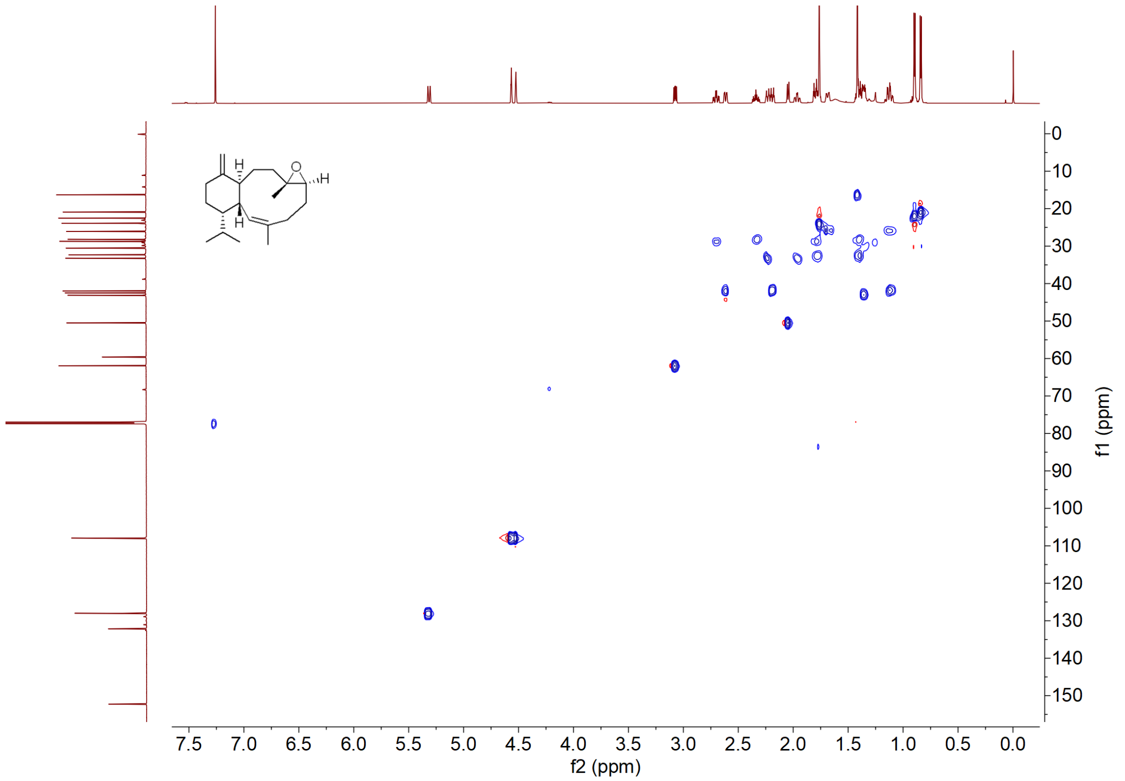

Supplementary Fig. 53. HSQC NMR spectrum of **1a** in $CDCl_3$.

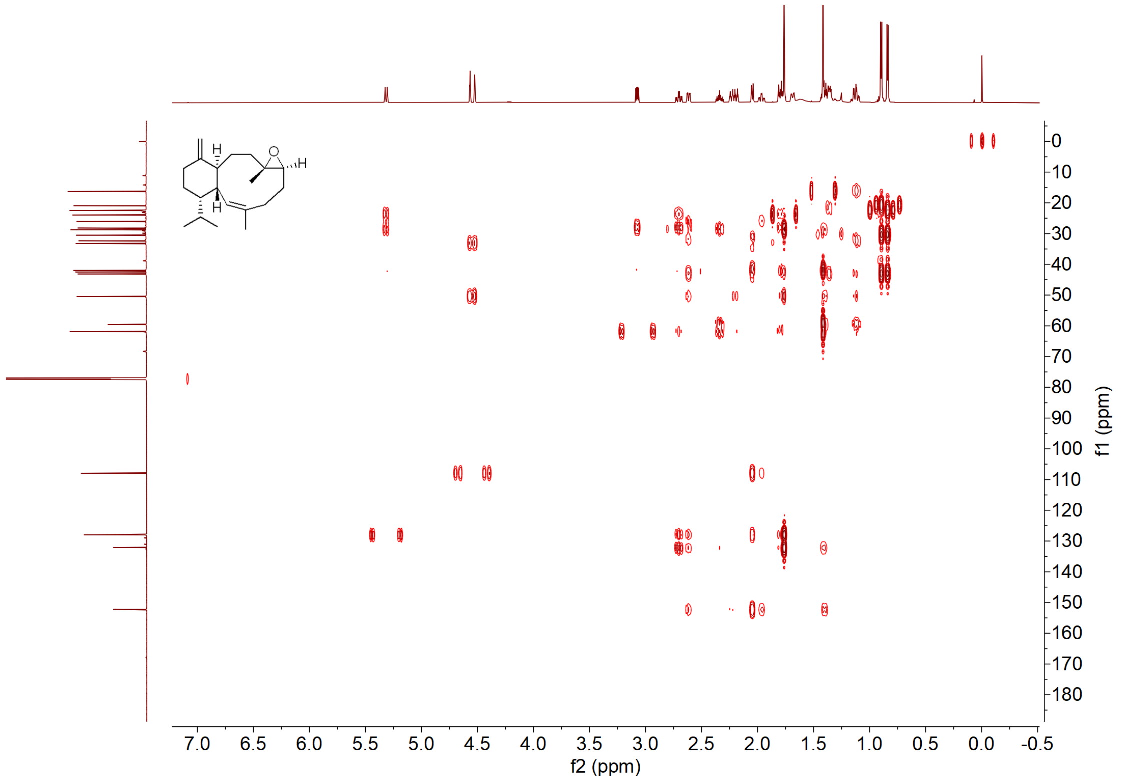

Supplementary Fig. 54. HMBC NMR spectrum of **1a** in $CDCl_3$.

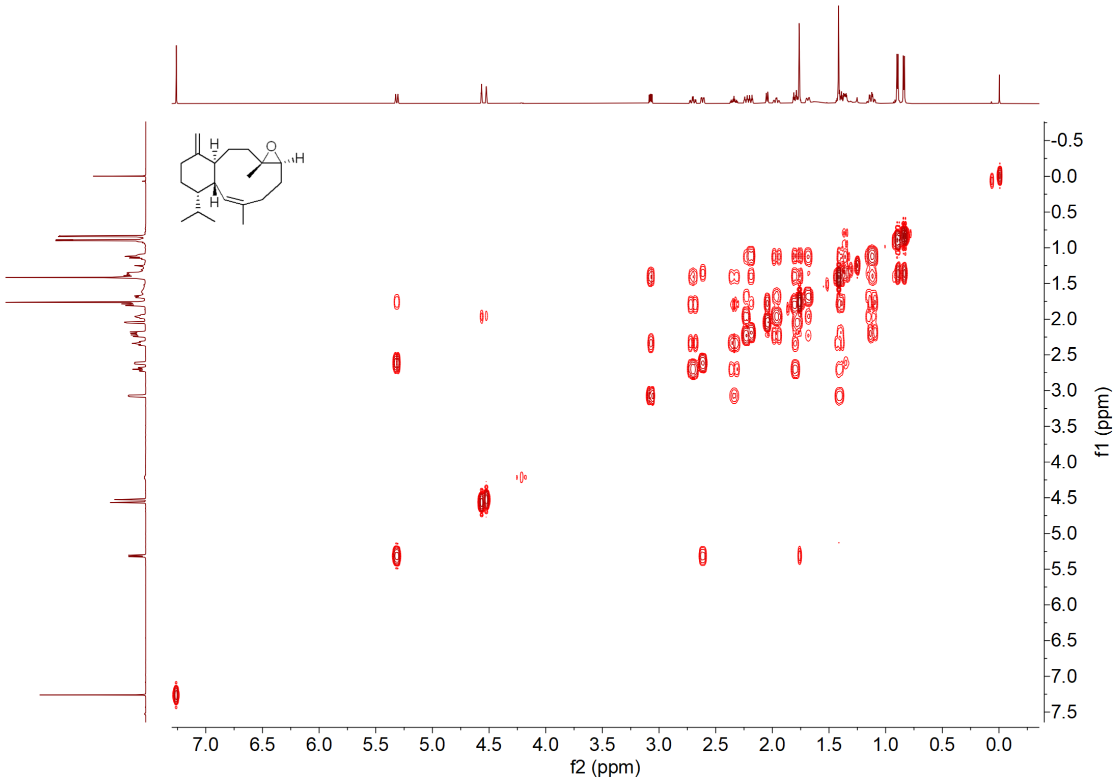

Supplementary Fig. 55. ^{1}H-^{1}H COSY NMR spectrum of **1a** in $CDCl_3$.

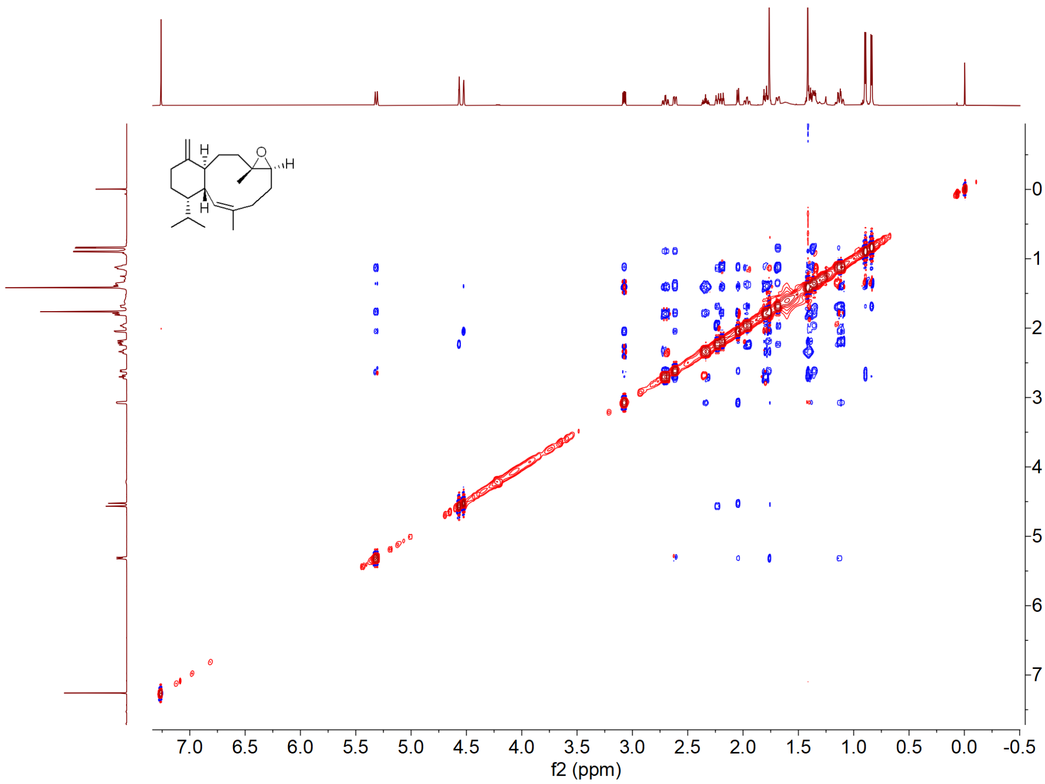

Supplementary Fig. 56. NOESY NMR spectrum of **1a** in $CDCl_3$.

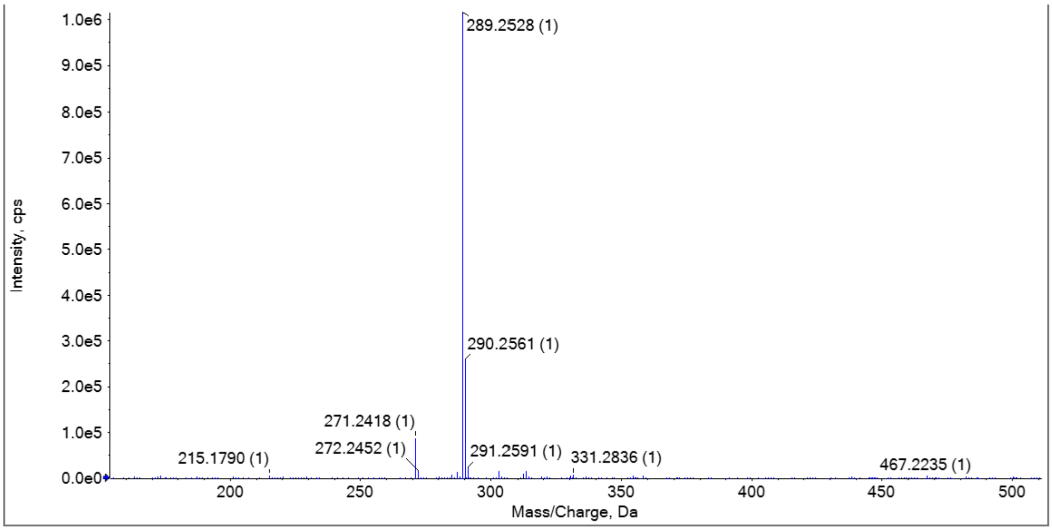

Formula Calculator Results

Measure m/z	Cal m/z	Error(mmu)	Error(ppm)	Ion Formula	Ion
289.2528	289.2526	0.2	0.7	C20H32O	$[M+H]^+$

Supplementary Fig. 57. HR-ESIMS of **1a**.

Original spectra for compound 1b.

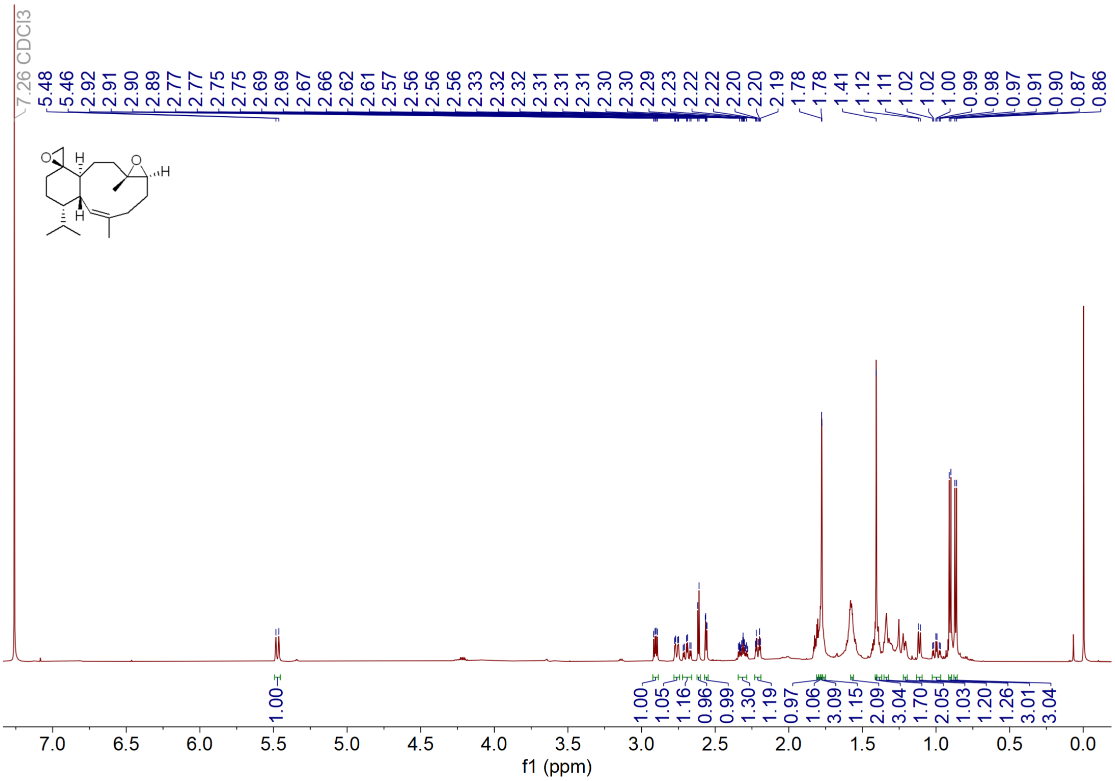

Supplementary Fig. 58. ^{1}H NMR spectrum (600 MHz) of **1b** in $CDCl_3$.

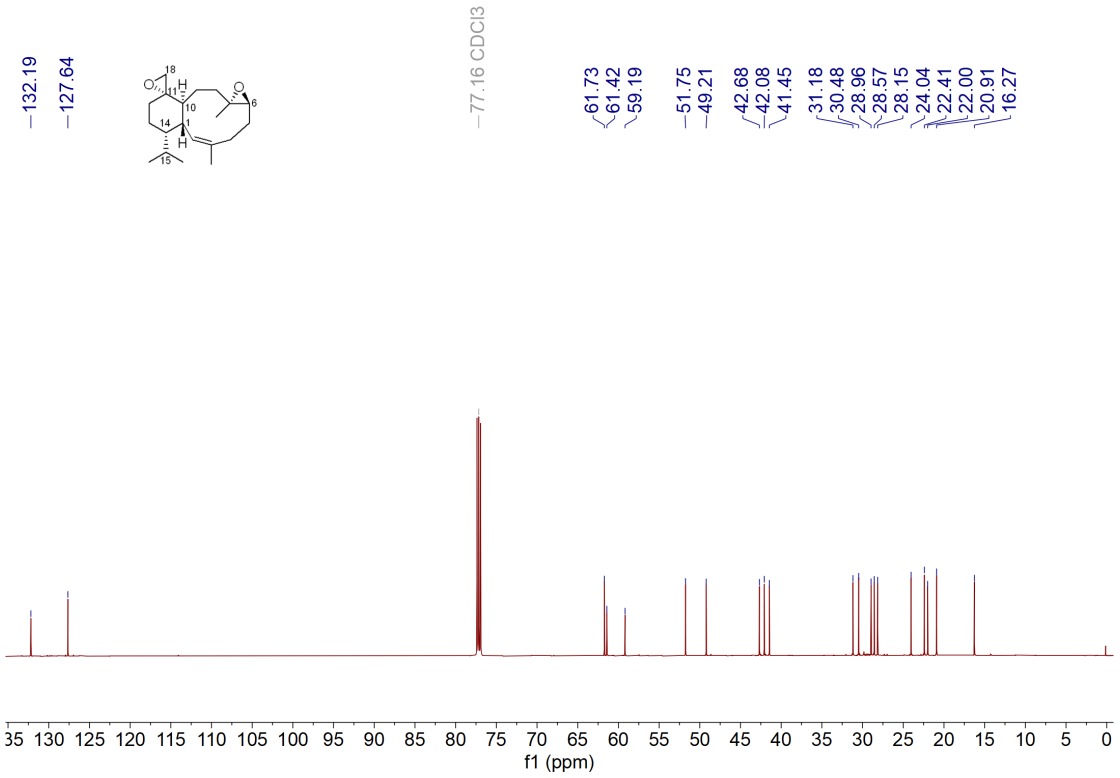

Supplementary Fig. 59. ^{13}C NMR spectrum (150 MHz) of **1b** in $CDCl_3$.

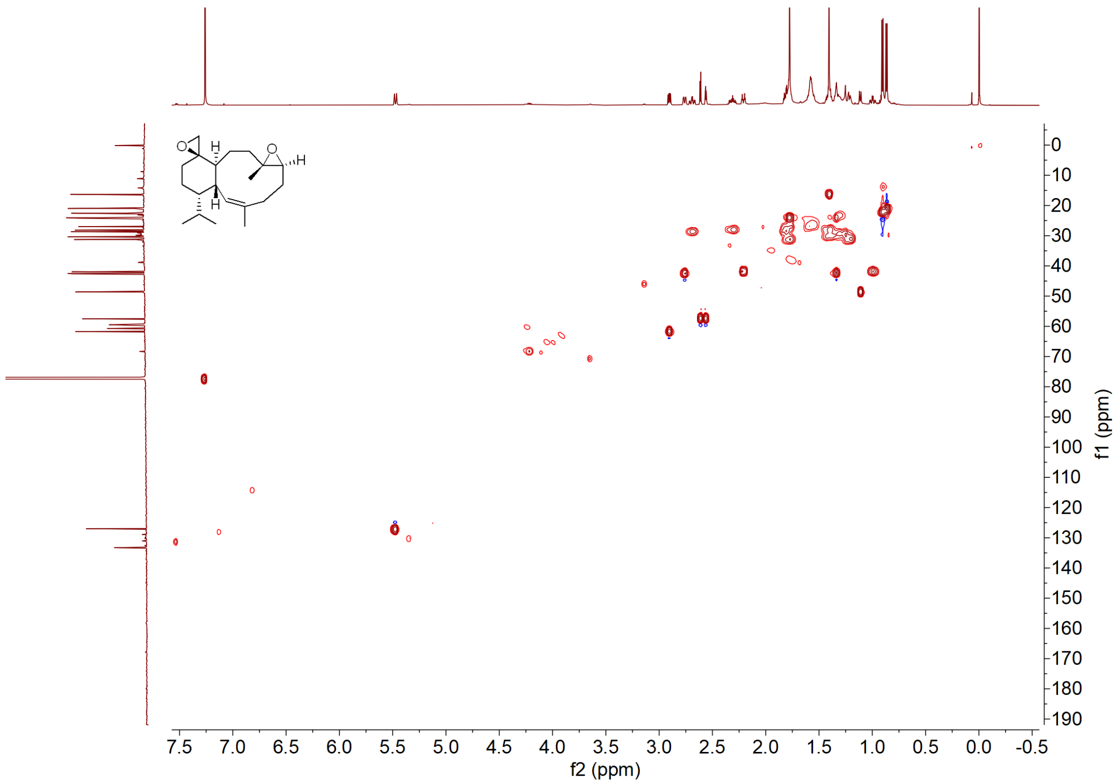

Supplementary Fig. 60. HSQC NMR spectrum of **1b** in $CDCl_3$.

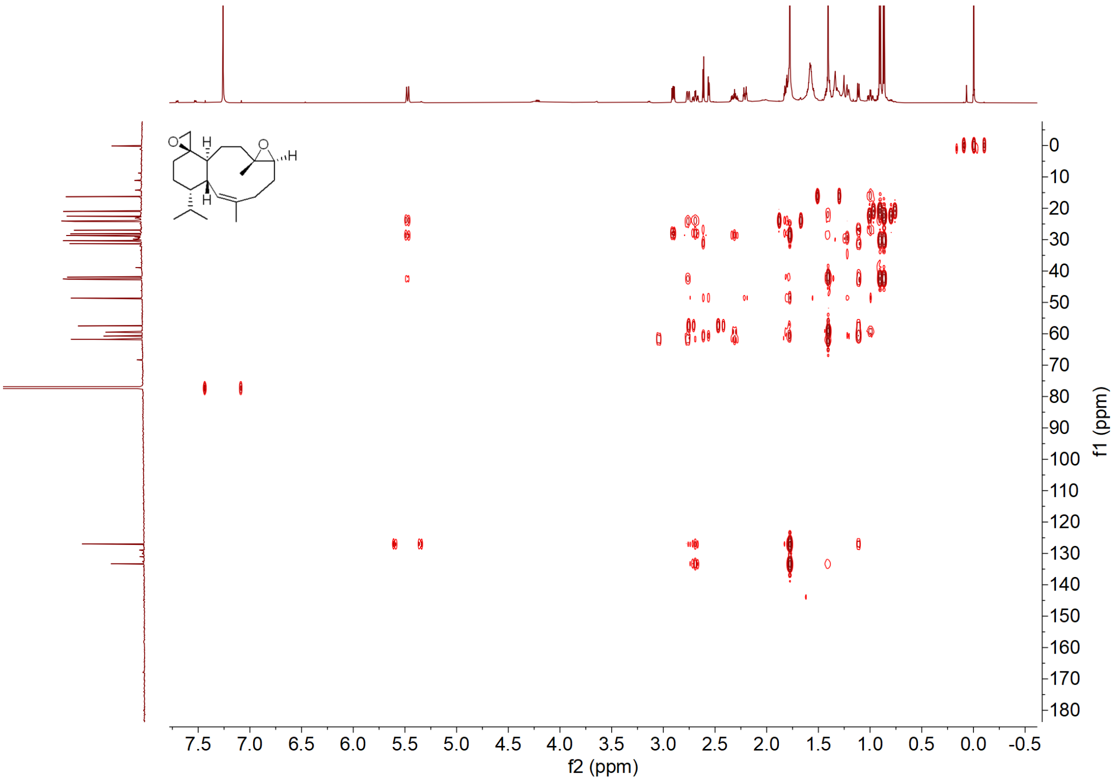

Supplementary Fig. 61. HMBC NMR spectrum of **1b** in $CDCl_3$.

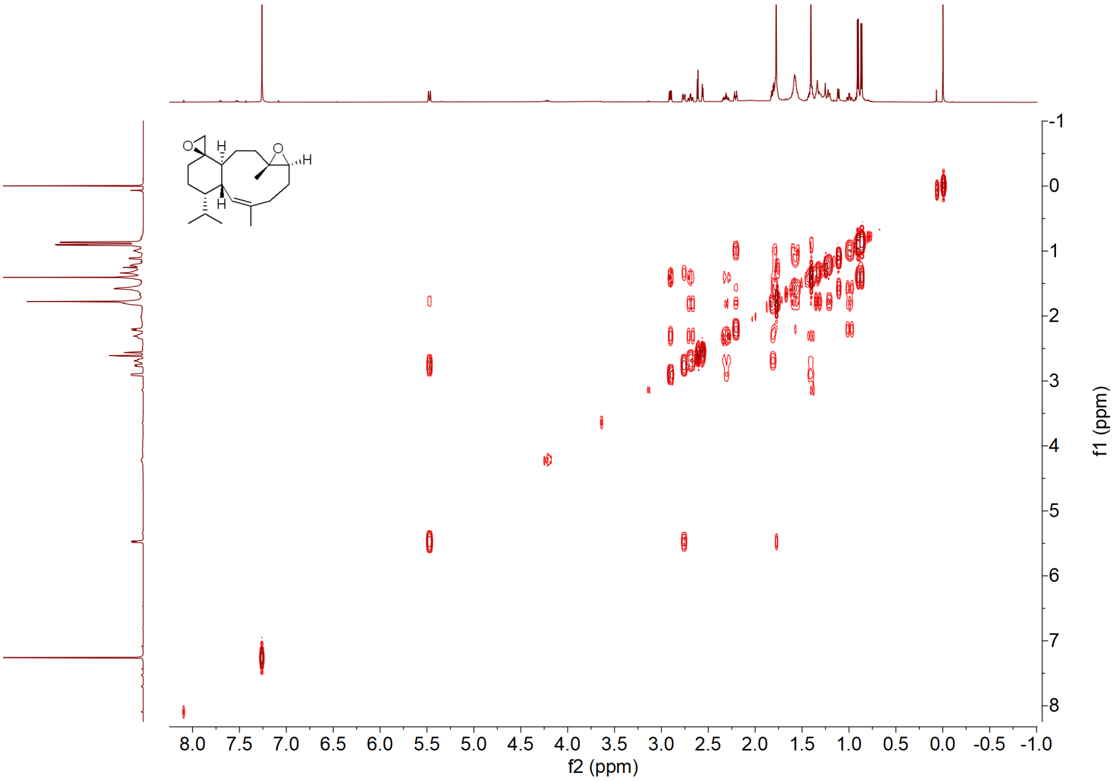

Supplementary Fig. 62. ^{1}H-^{1}H COSY NMR spectrum of **1b** in $CDCl_3$.

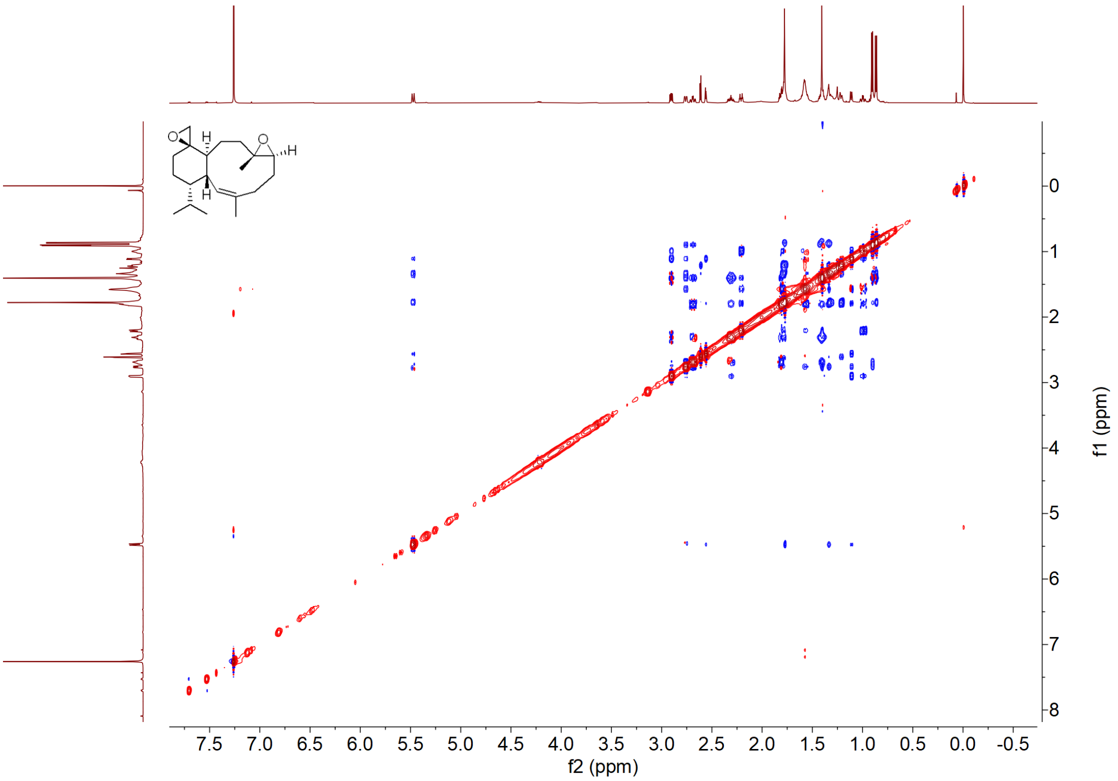

Supplementary Fig. 63. NOESY NMR spectrum of **1b** in $CDCl_3$.

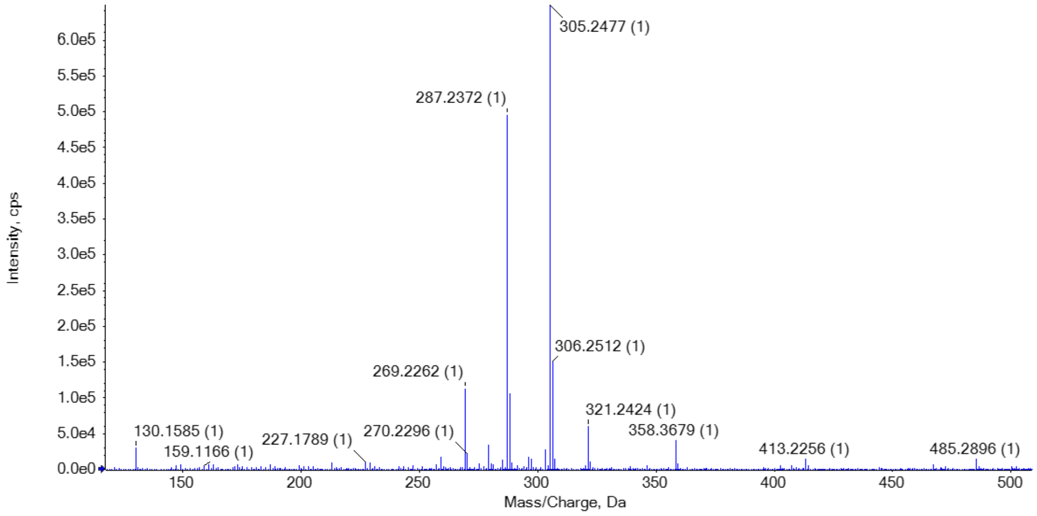

Formula Calculator Results

Measure m/z	Cal m/z	Error(mmu)	Error(ppm)	Ion Formula	Ion
305.2477	305.2475	0.2	0.6	C20H32O2	$[M+H]^+$

Supplementary Fig. 64. HR-ESIMS of **1b**.

Original spectra for compound 1c.

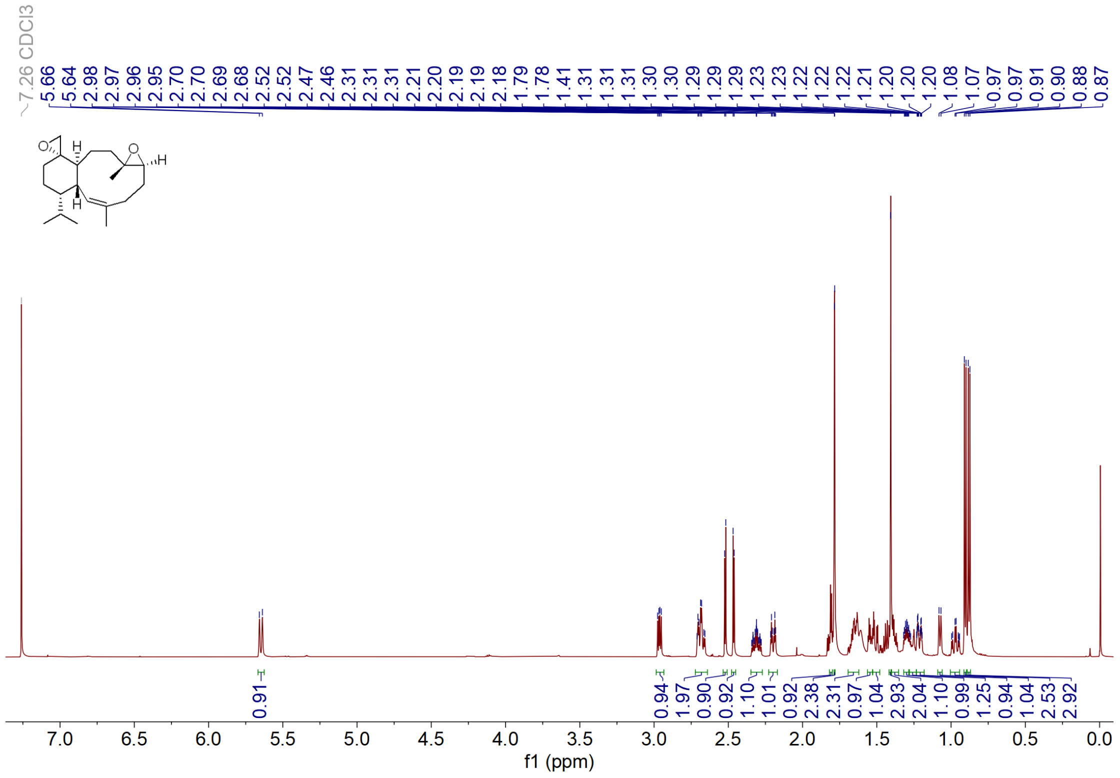

Supplementary Fig. 65. ^{1}H NMR spectrum (600 MHz) of **1c** in $CDCl_3$.

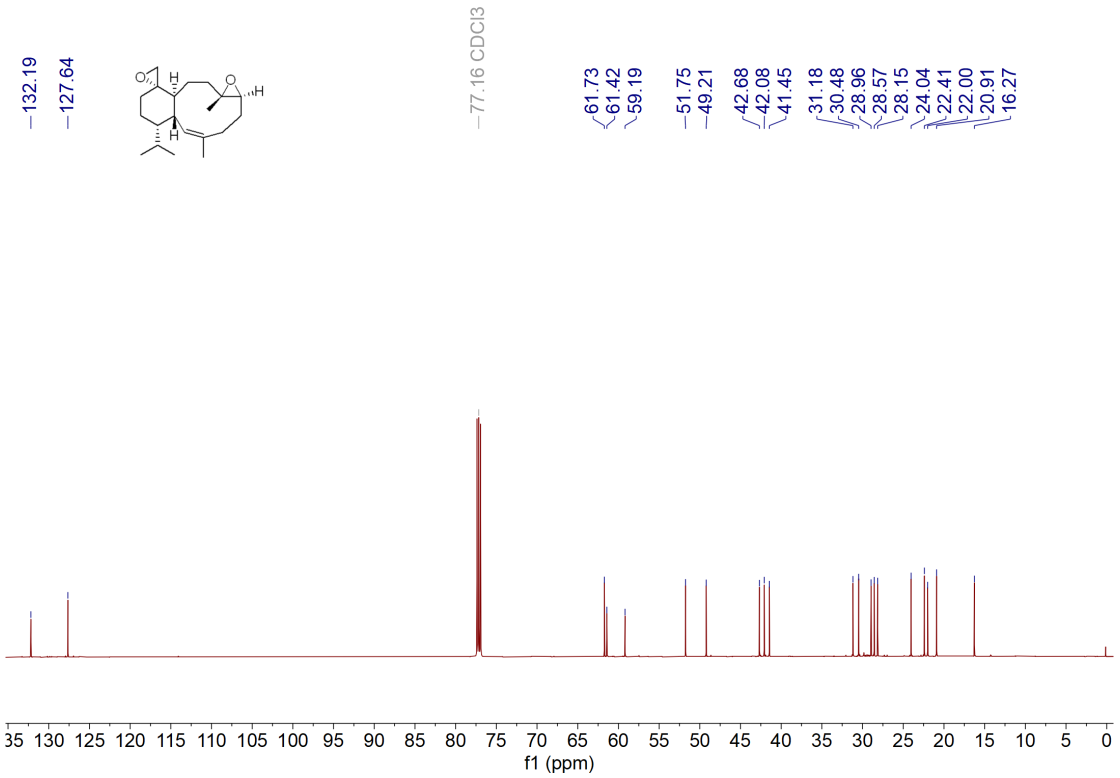

Supplementary Fig. 66. ^{13}C NMR spectrum (150 MHz) of **1c** in $CDCl_3$.

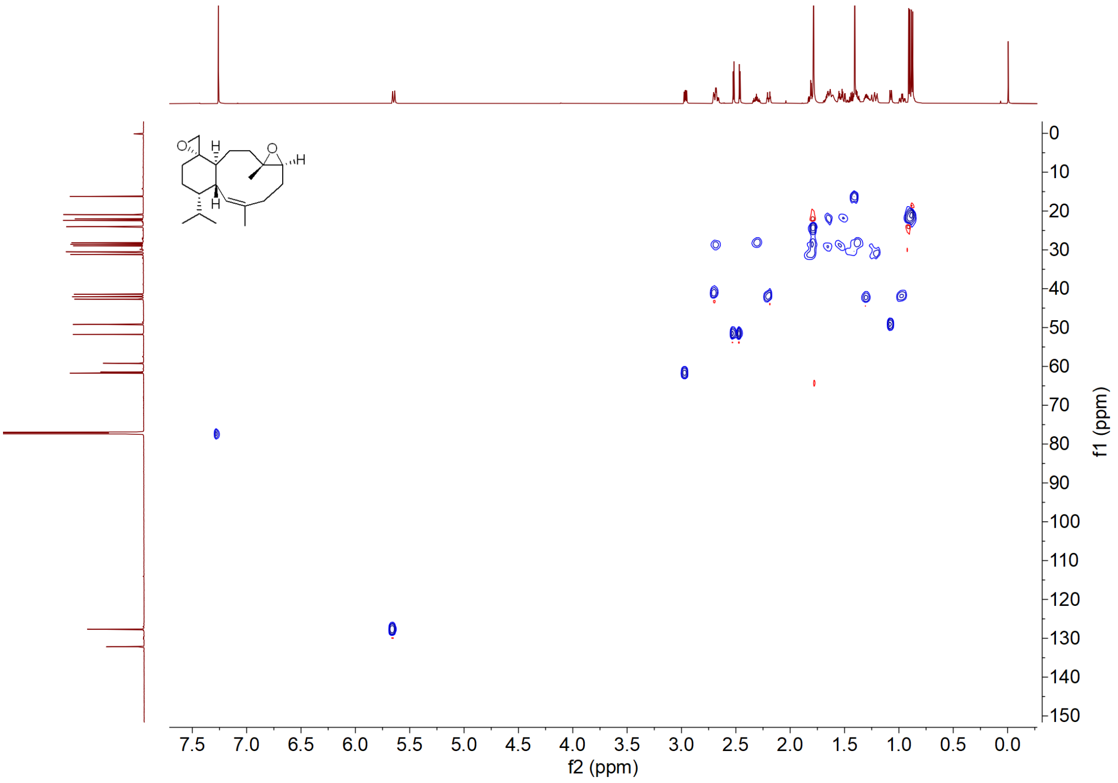

Supplementary Fig. 67. HSQC NMR spectrum of **1c** in $CDCl_3$.

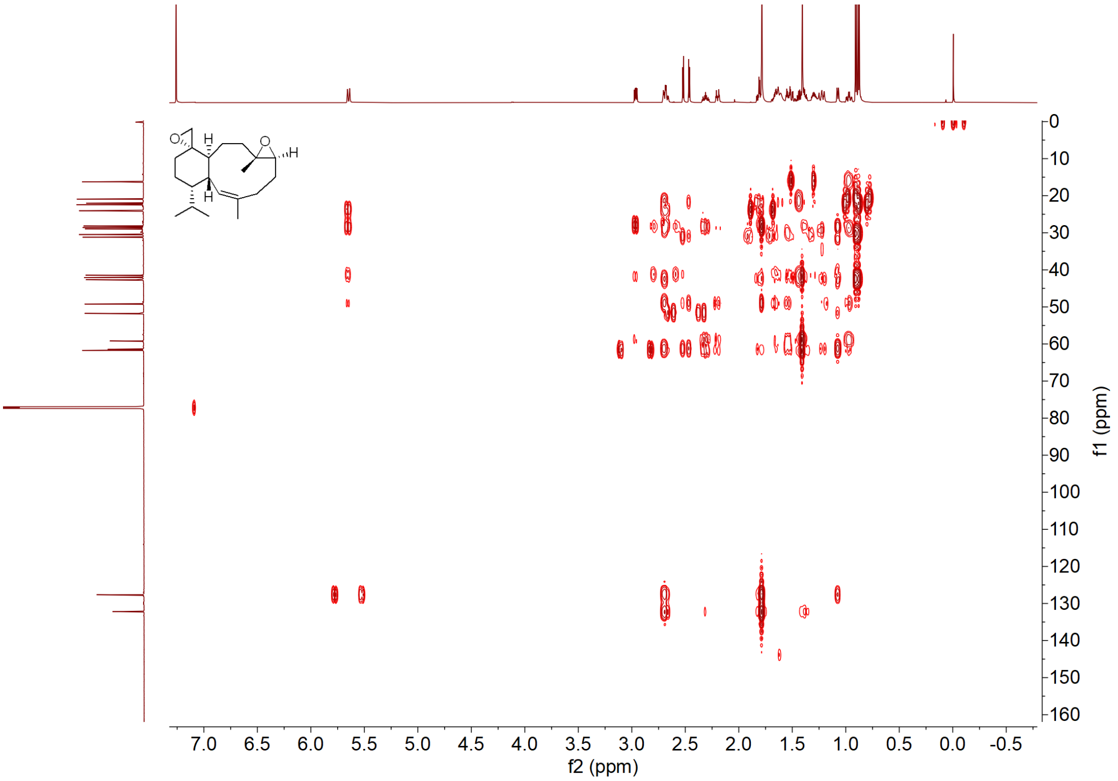

Supplementary Fig. 68. HMBC NMR spectrum of **1c** in $CDCl_3$.

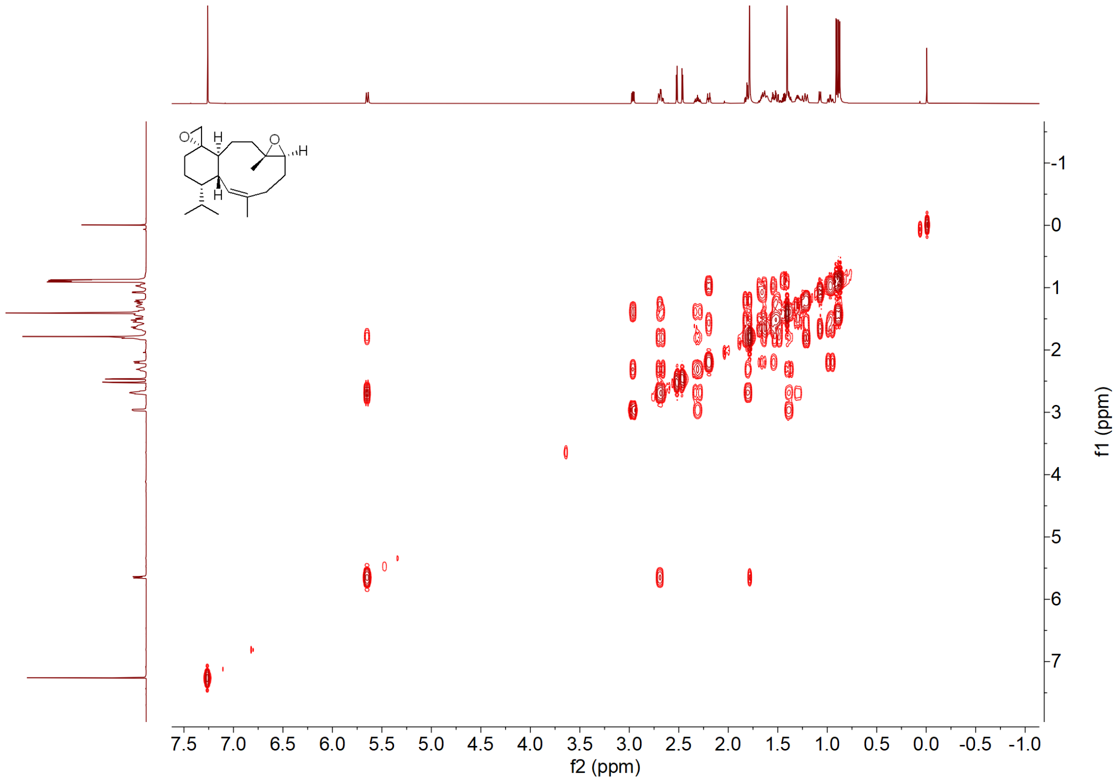

Supplementary Fig. 69. ^{1}H-^{1}H COSY NMR spectrum of **1c** in $CDCl_3$.

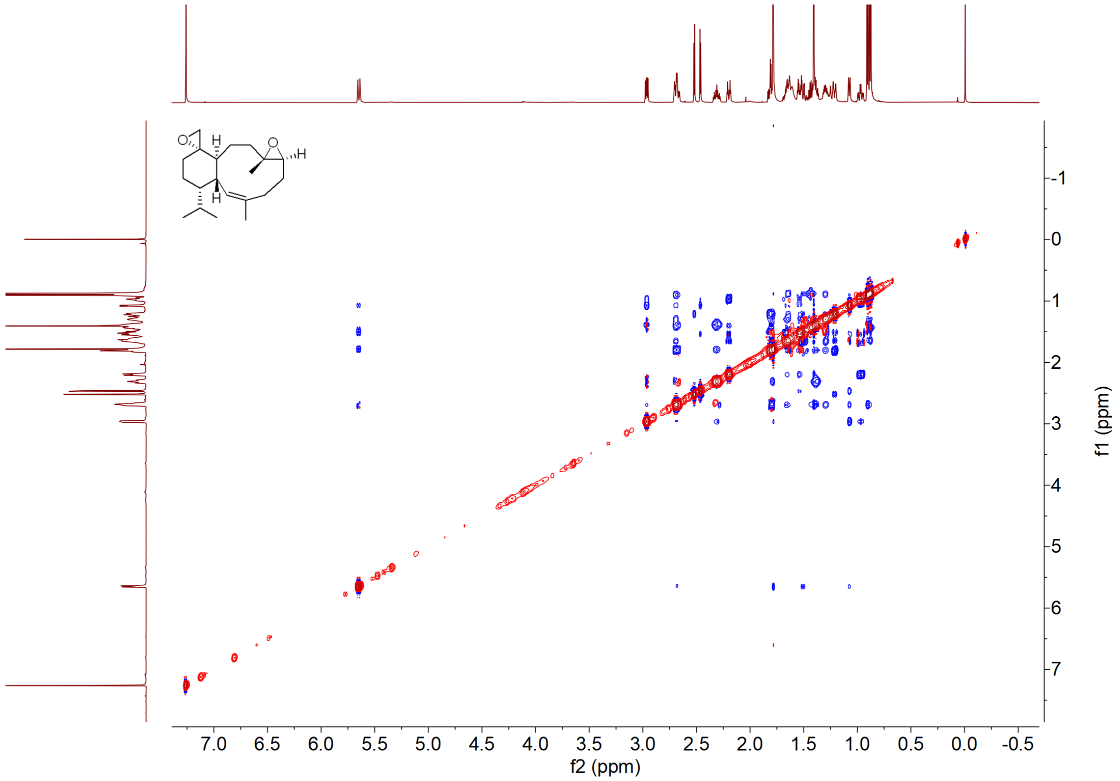

Supplementary Fig. 70. NOESY NMR spectrum of **1c** in $CDCl_3$.

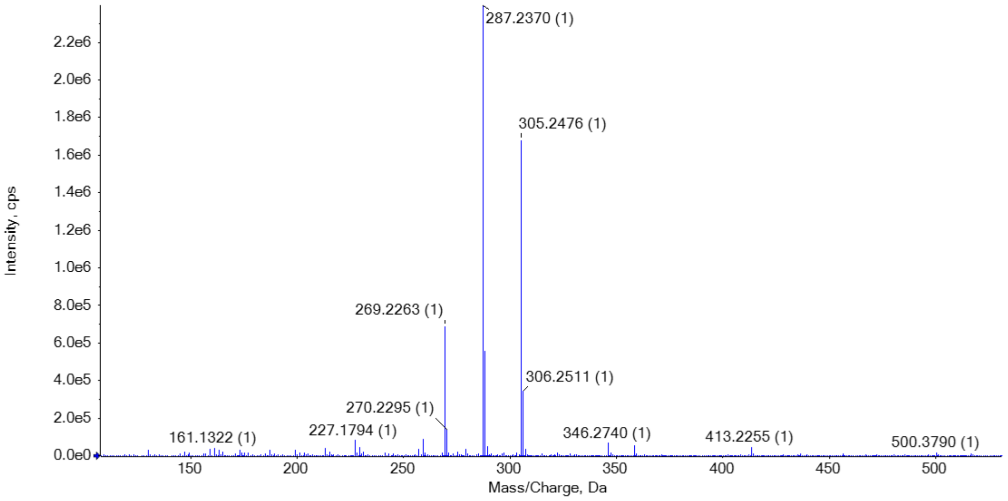

Formula Calculator Results

Measure m/z	Cal m/z	Error(mmu)	Error(ppm)	Ion Formula	Ion
305.2476	305.2475	0.1	0.3	C20H32O2	$[M+H]^+$

Supplementary Fig. 71. HR-ESIMS of **1c**.

Original spectra for compound 1d.

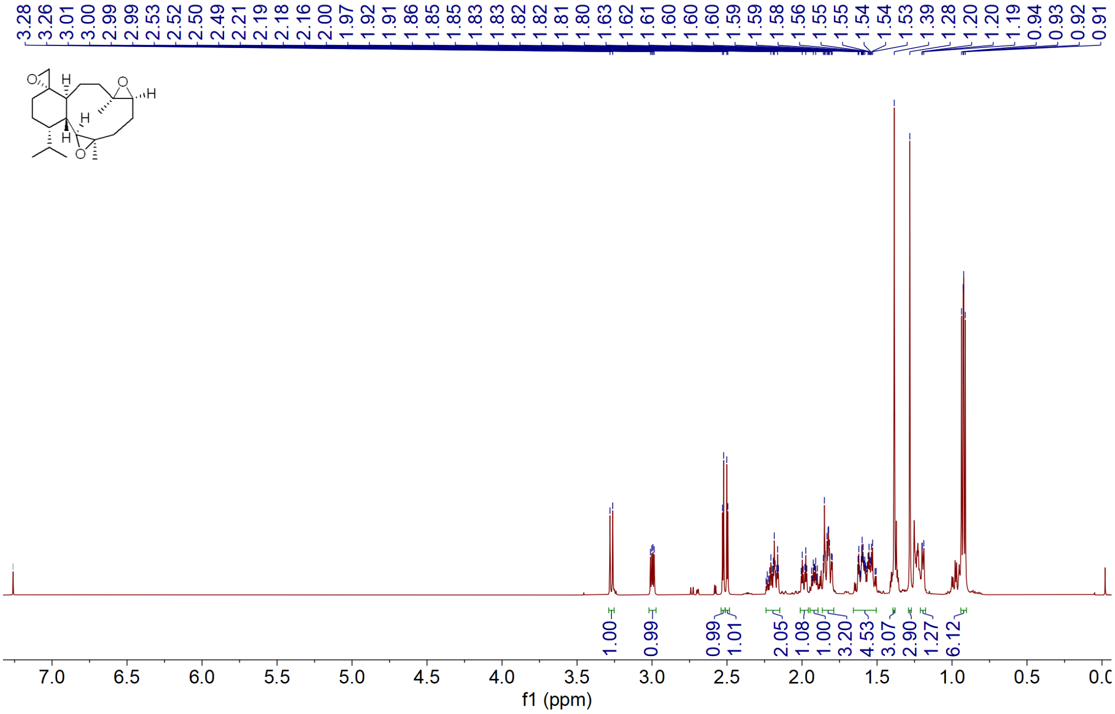

Supplementary Fig. 72. ^{1}H NMR spectrum (600 MHz) of **1d** in $CDCl_3$.

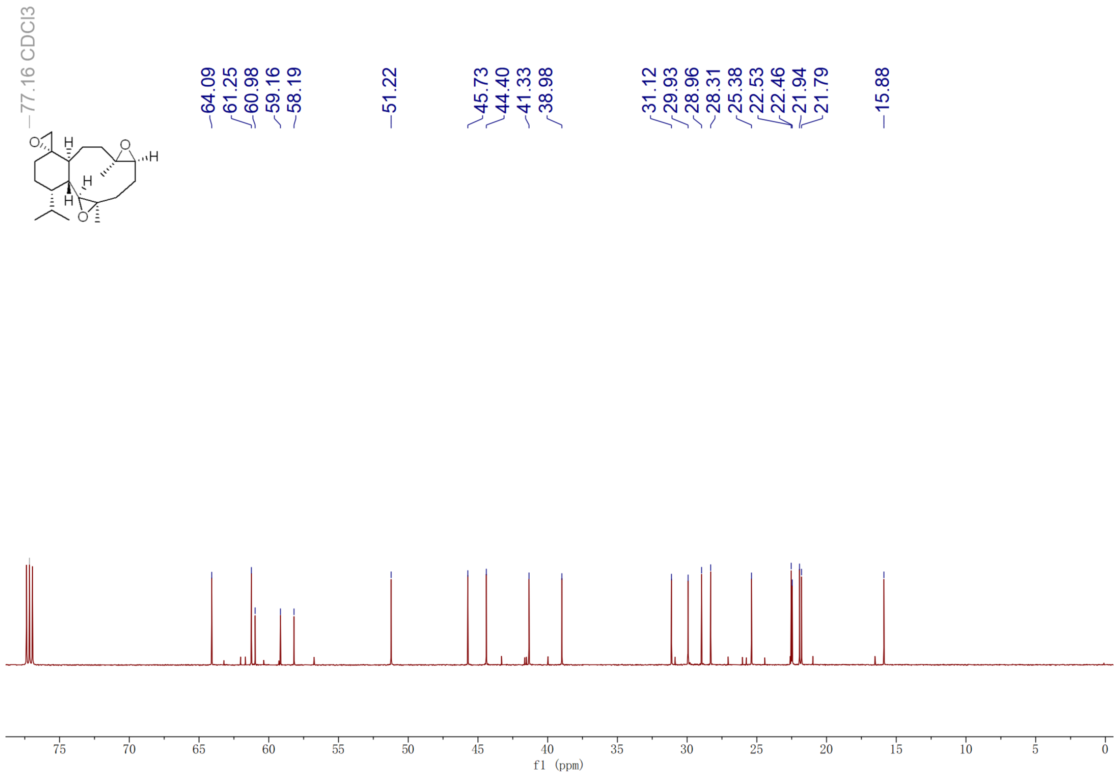

Supplementary Fig. 73. ^{13}C NMR spectrum (150 MHz) of **1d** in $CDCl_3$.

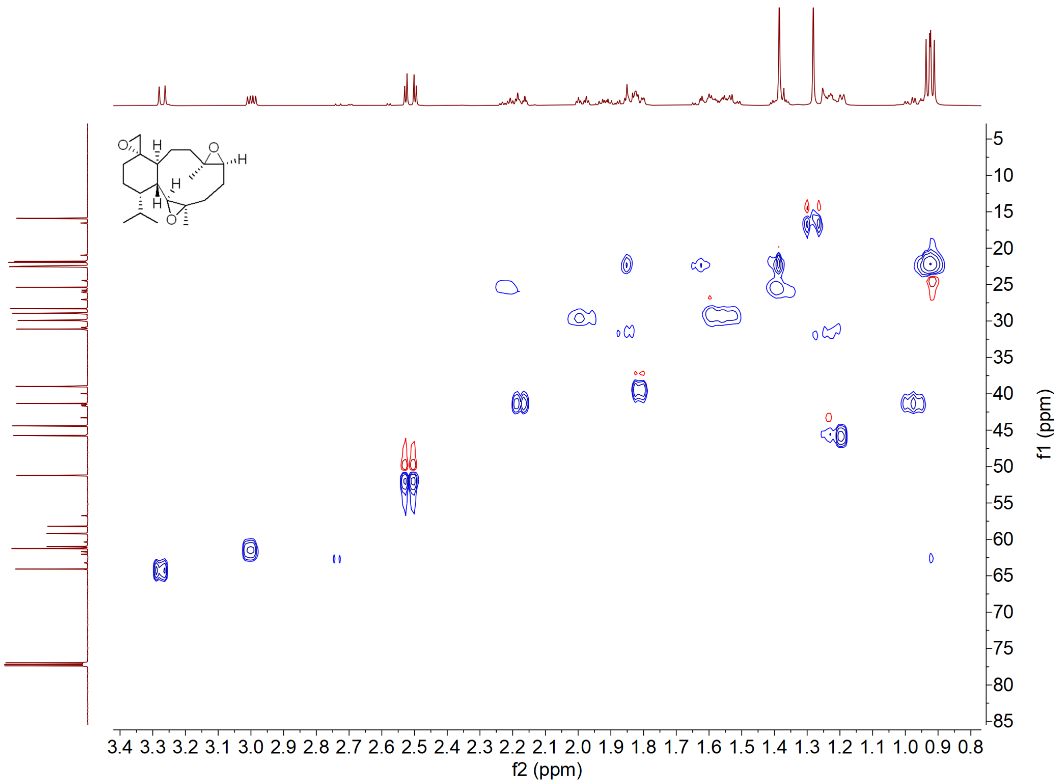

Supplementary Fig. 74. HSQC NMR spectrum of **1d** in $CDCl_3$.

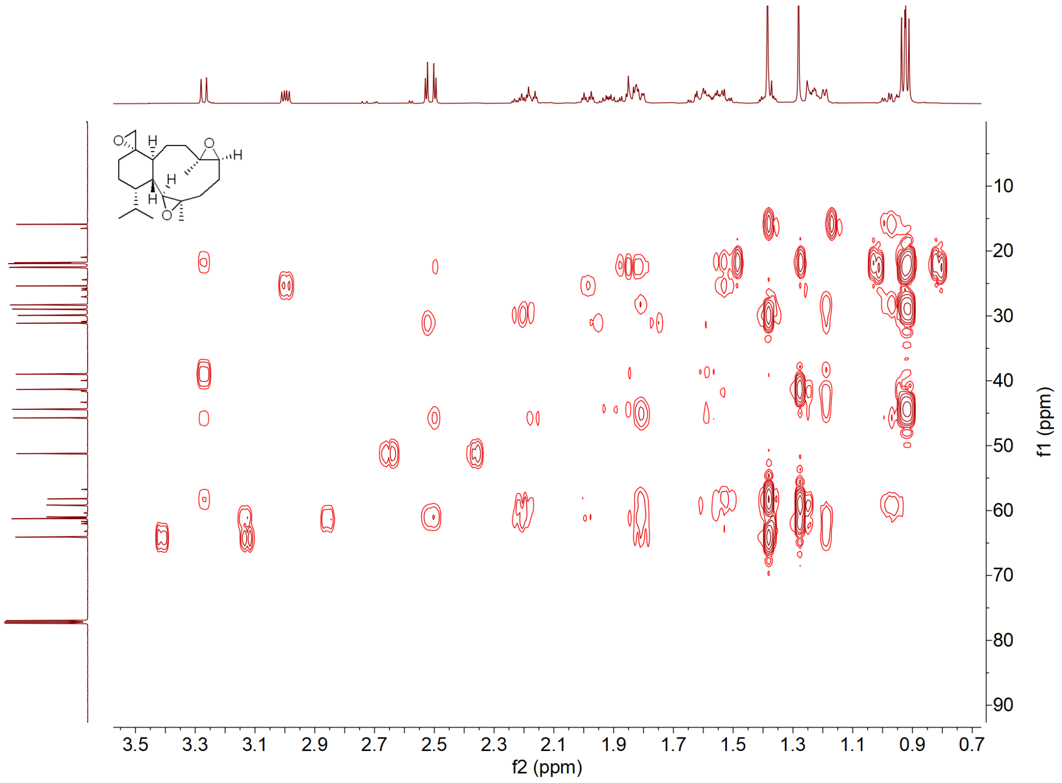

Supplementary Fig. 75. HMBC NMR spectrum of **1d** in $CDCl_3$.

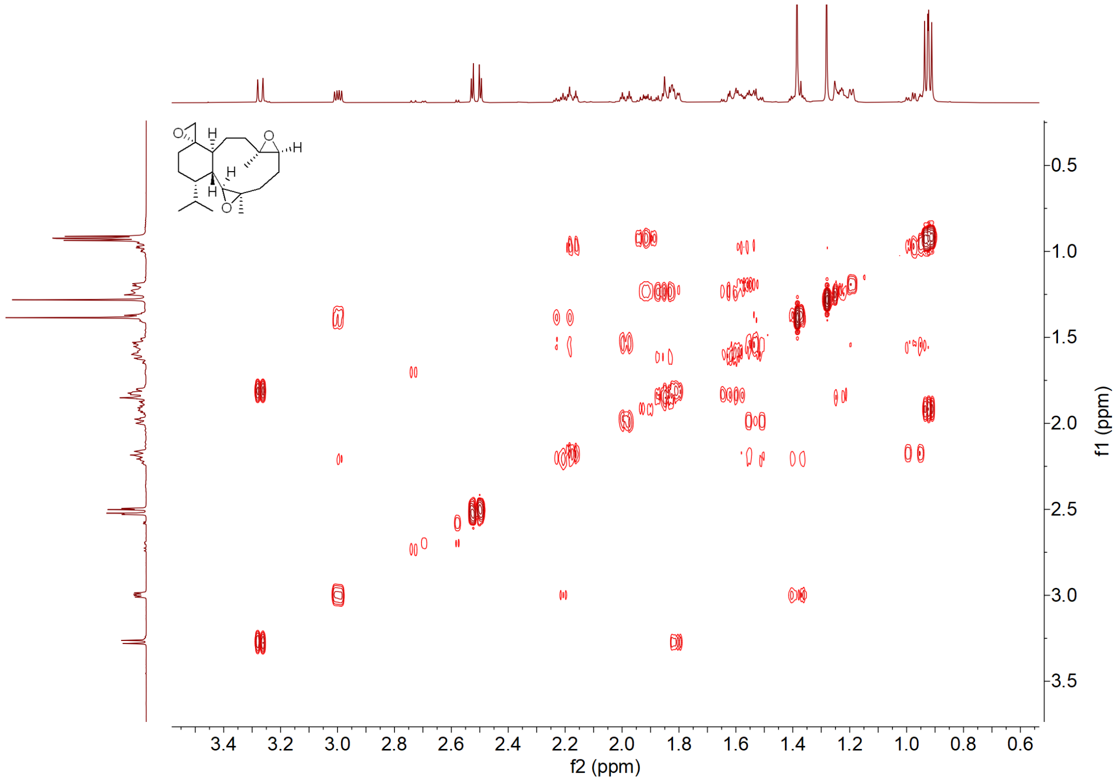

Supplementary Fig. 76. ^1H-^1H COSY NMR spectrum of **1d** in $CDCl_3$.

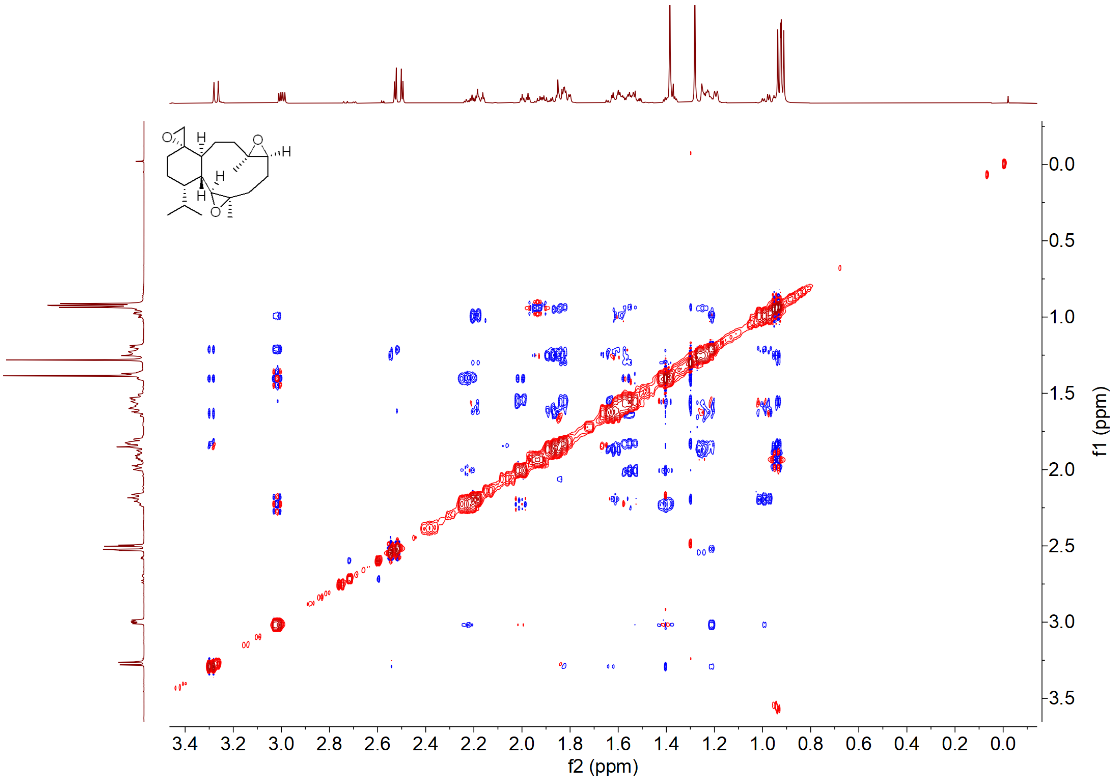

Supplementary Fig. 77. NOESY NMR spectrum of **1d** in $CDCl_3$.

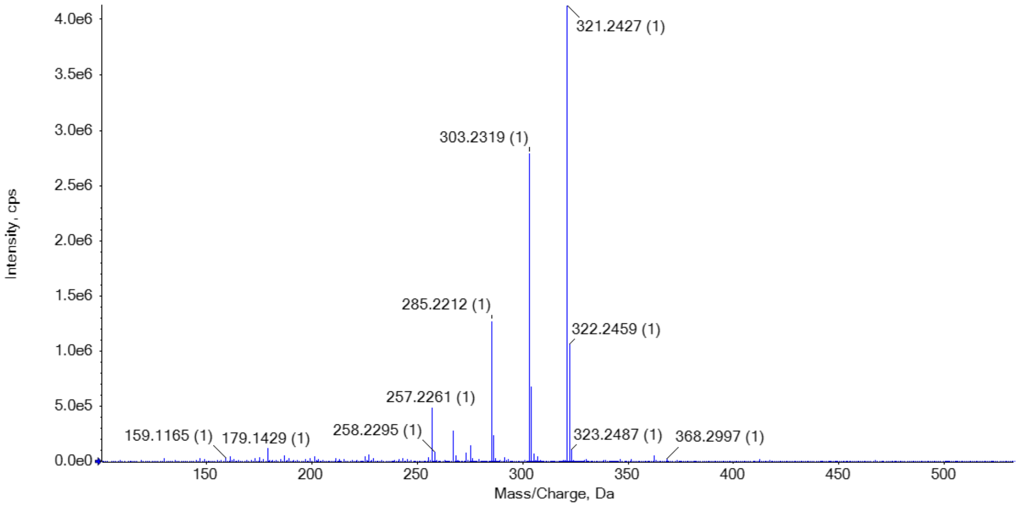

Formula Calculator Results

Measure m/z	Cal m/z	Error(mmu)	Error(ppm)	Ion Formula	Ion
321.2427	321.2424	0.3	0.9	C20H32O3	$[M+H]^+$

Supplementary Fig. 78. HR-ESIMS of **1d**.

Original spectra for ($^{2}H_{2}$)-1.

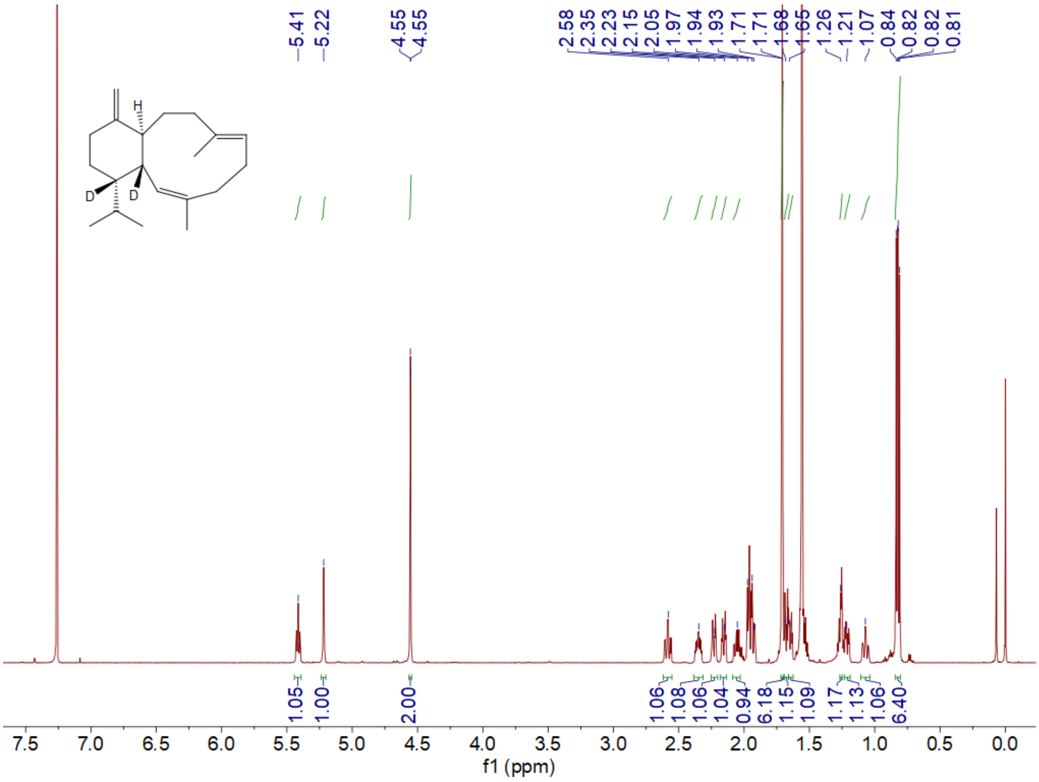

Supplementary Fig. 79. ^{1}H NMR spectrum (600 MHz) of ($^{2}H_{2}$)-**1** in $CDCl_3$.

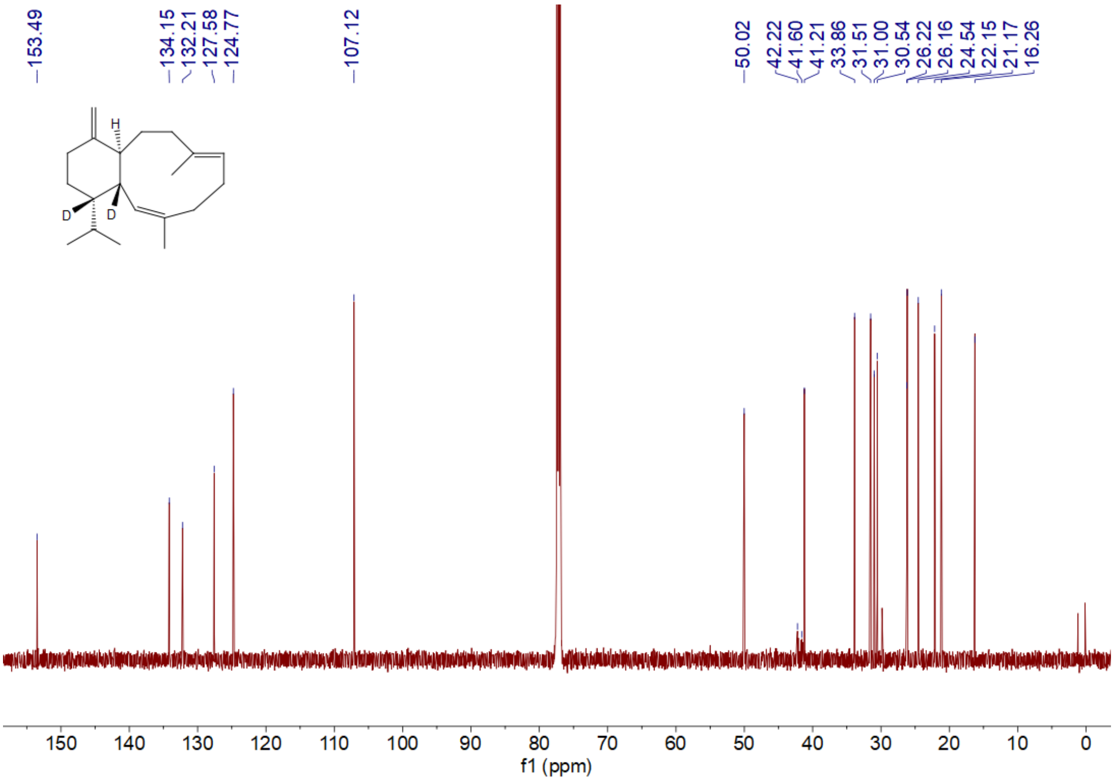

Supplementary Fig. 80. ^{13}C NMR spectrum (150 MHz) of ($^{2}H_{2}$)-**1** in $CDCl_3$.

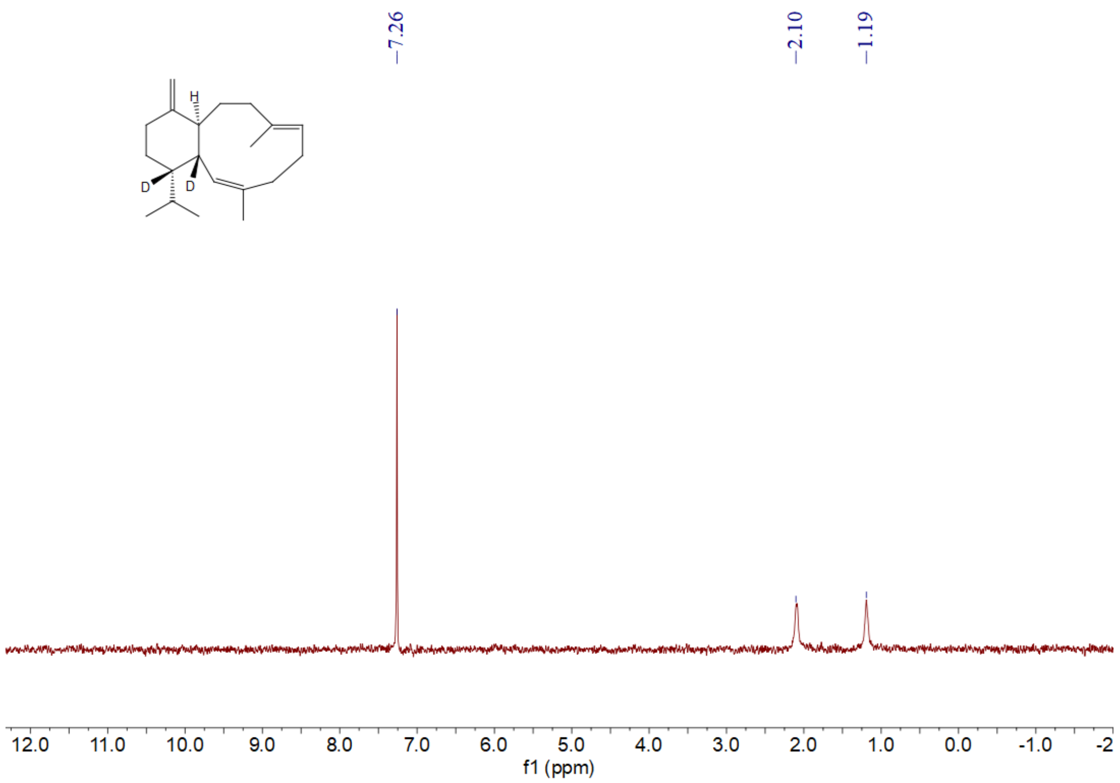

Supplementary Fig. 81. ^2H NMR spectrum of ($^2H_2$)-**1** in $CDCl_3$.

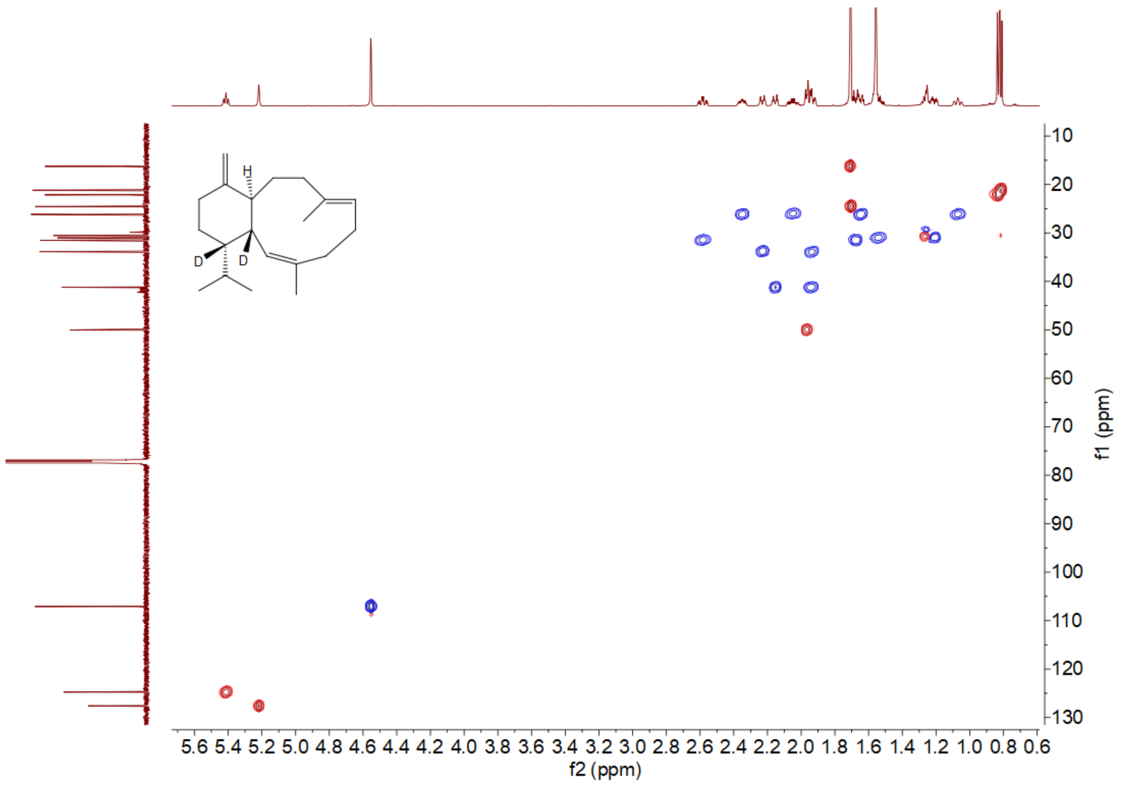

Supplementary Fig. 82. HSQC NMR spectrum of ($^2H_2$)-**1** in $CDCl_3$.

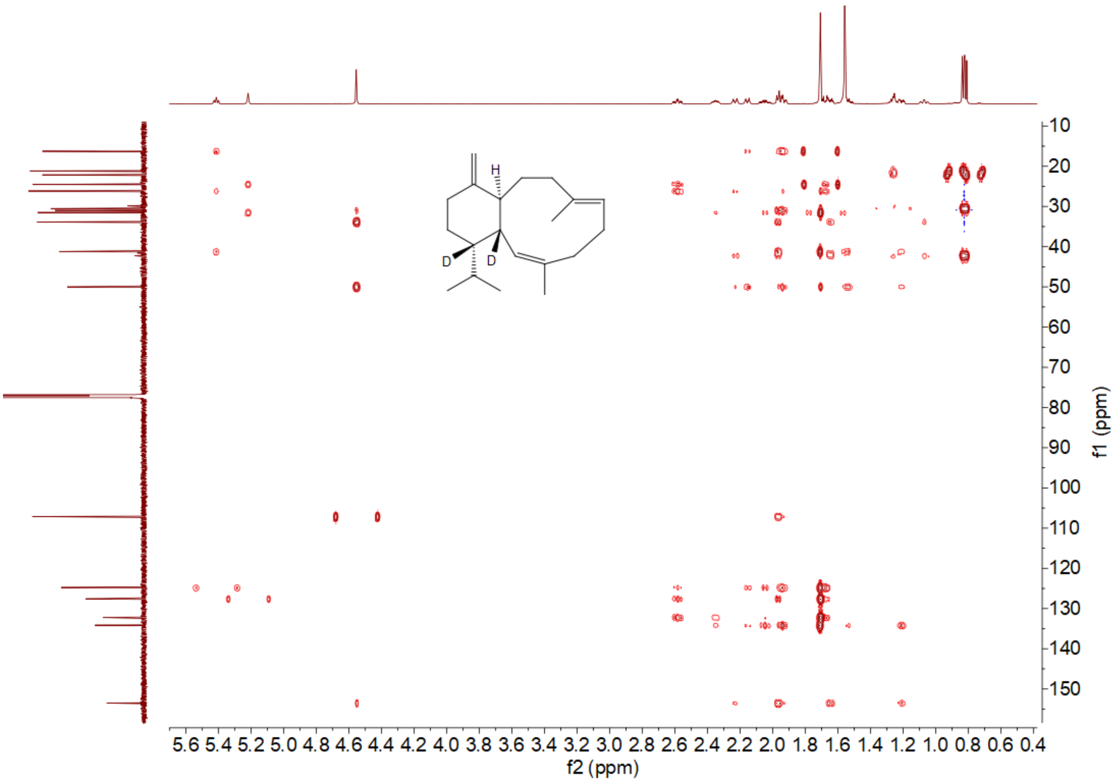

Supplementary Fig. 83. HMBC NMR spectrum of ($^{2}H_2$)-**1** in $CDCl_3$.

Original spectra for compound 3.

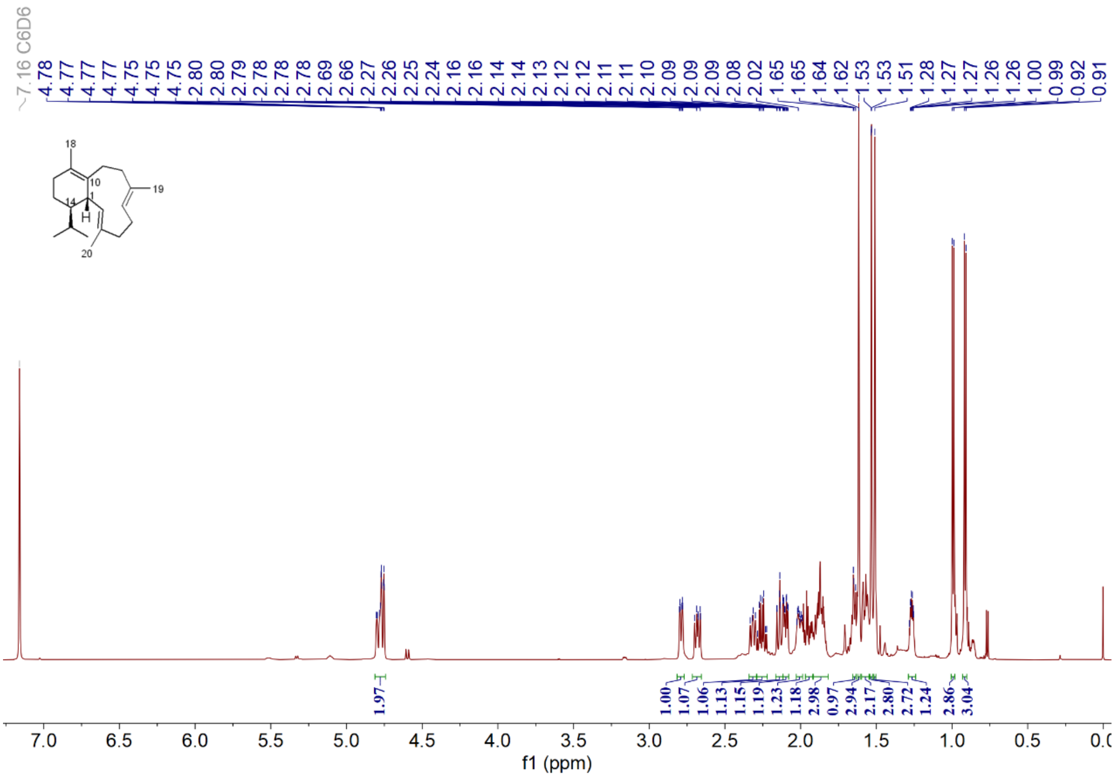

Supplementary Fig. 84. ^{1}H NMR spectrum (600 MHz) of **3** in C_6D_6.

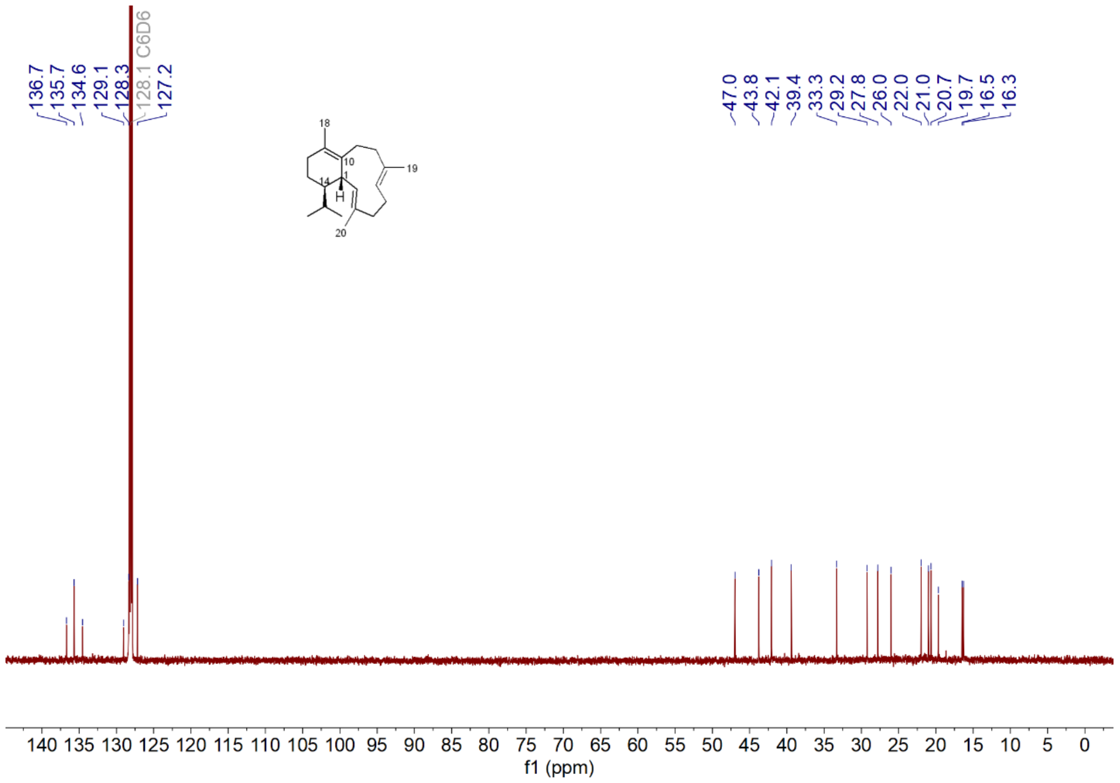

Supplementary Fig. 85. ^{13}C NMR spectrum (150 MHz) of **3** in C_6D_6.

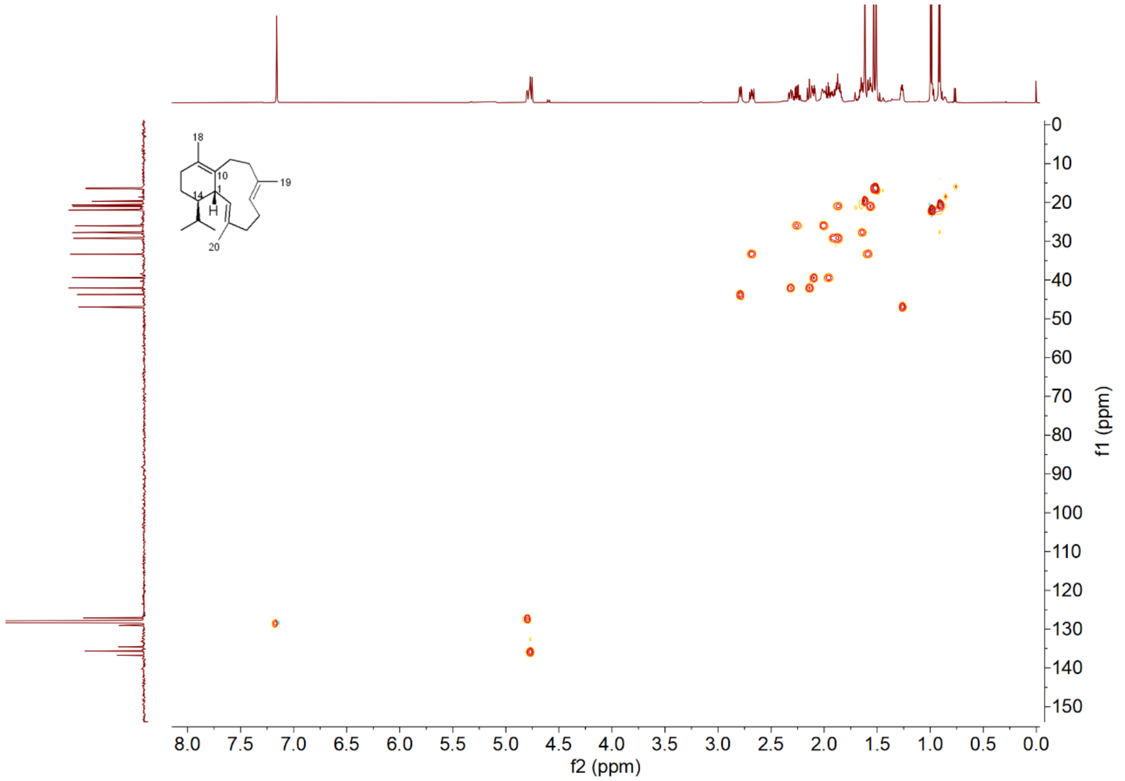

Supplementary Fig. 86. HSQC NMR spectrum of **3** in C_6D_6.

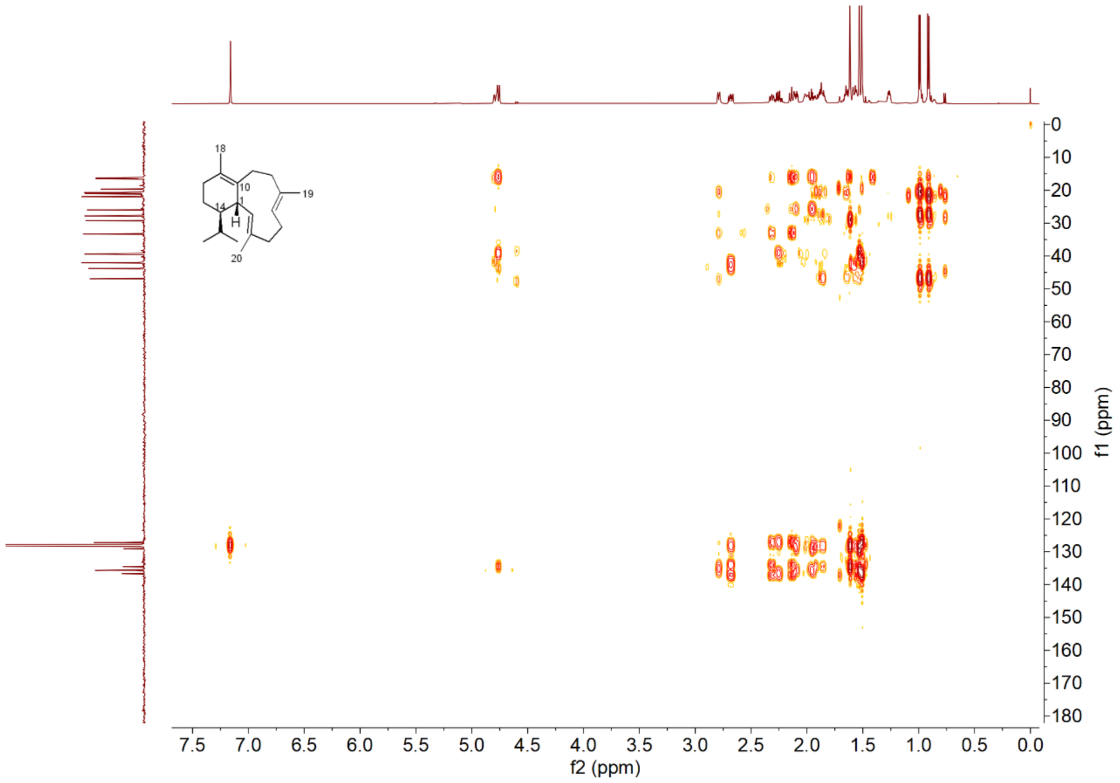

Supplementary Fig. 87. HMBC NMR spectrum of **3** in C_6D_6.

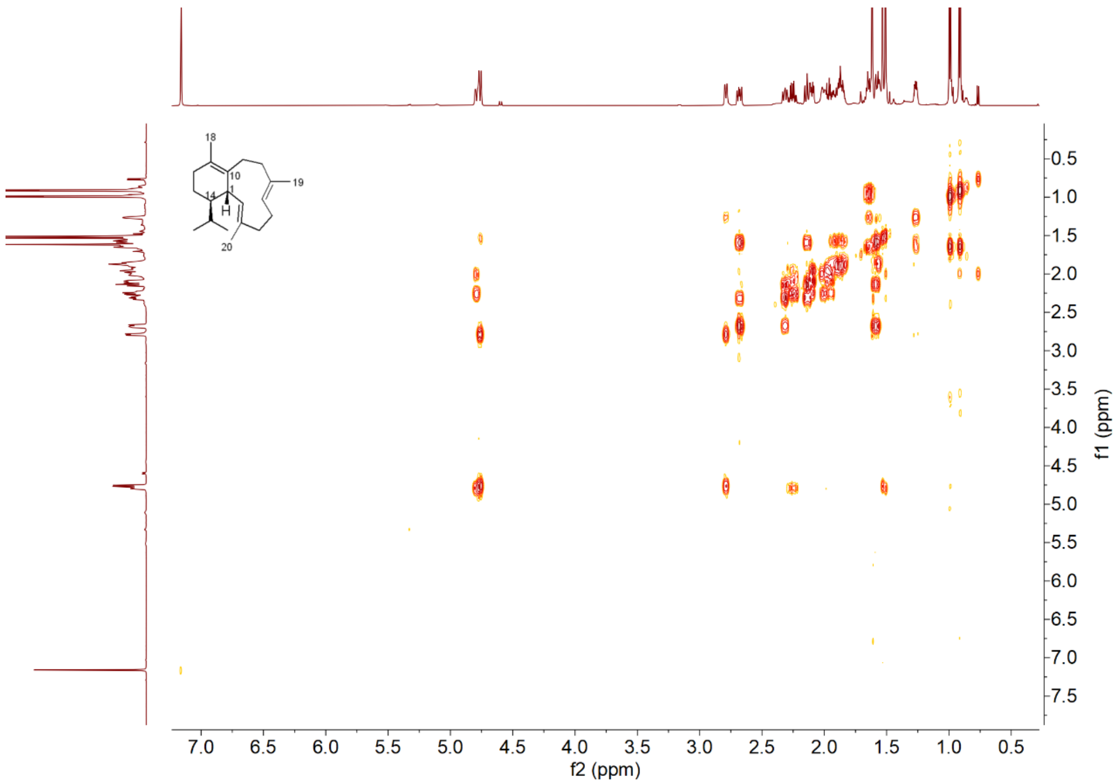

Supplementary Fig. 88. ^1H-^1H COSY NMR spectrum of **3** in C_6D_6.

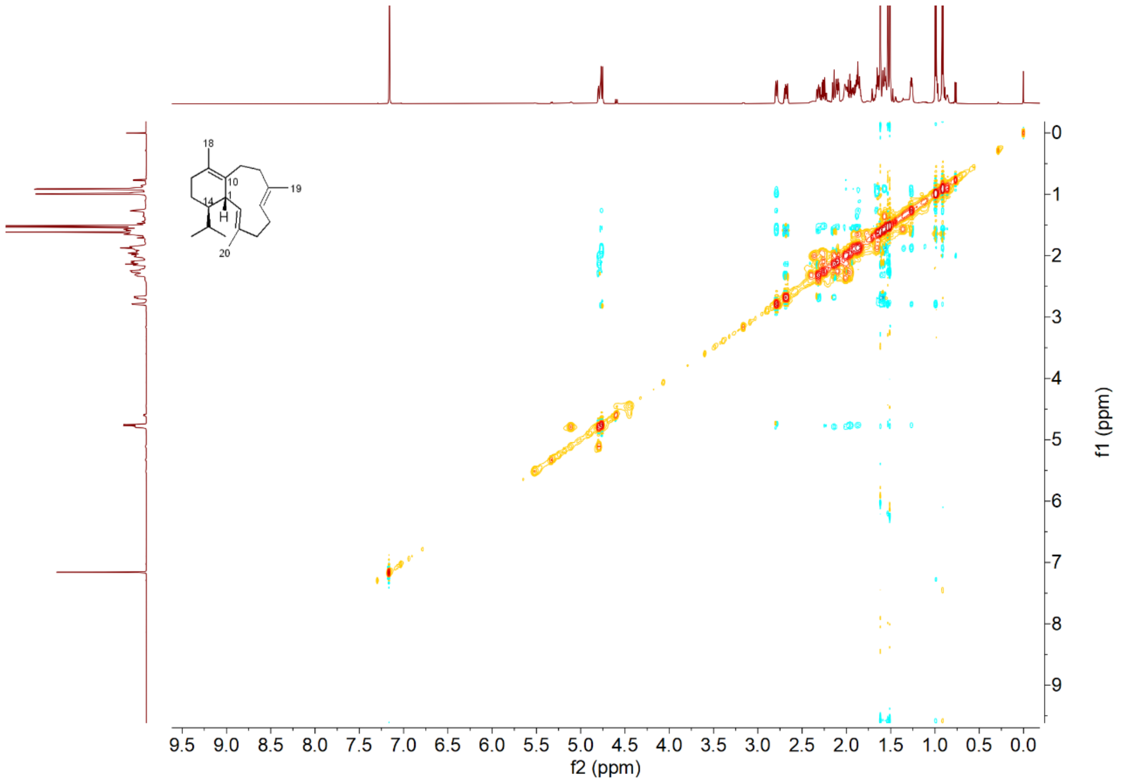

Supplementary Fig. 89. NOESY NMR spectrum of **3** in C_6D_6.

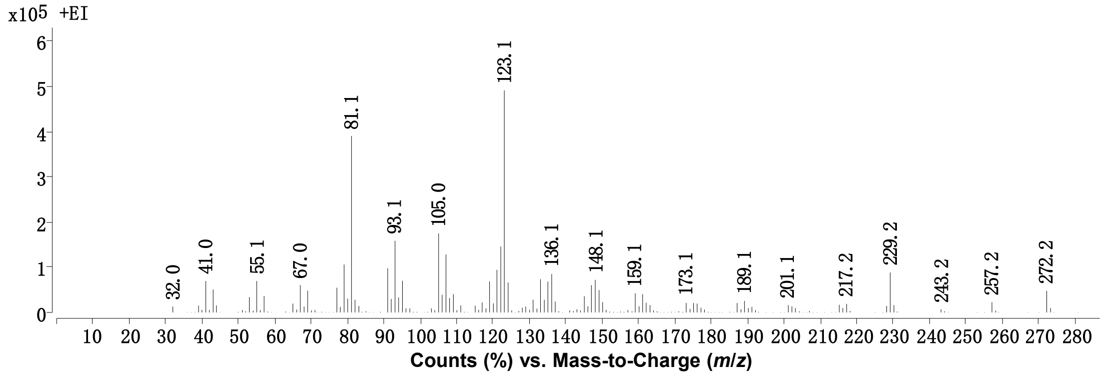

Supplementary Fig. 90. GC-MS spectra of **3**.

Original spectra for compound 4.

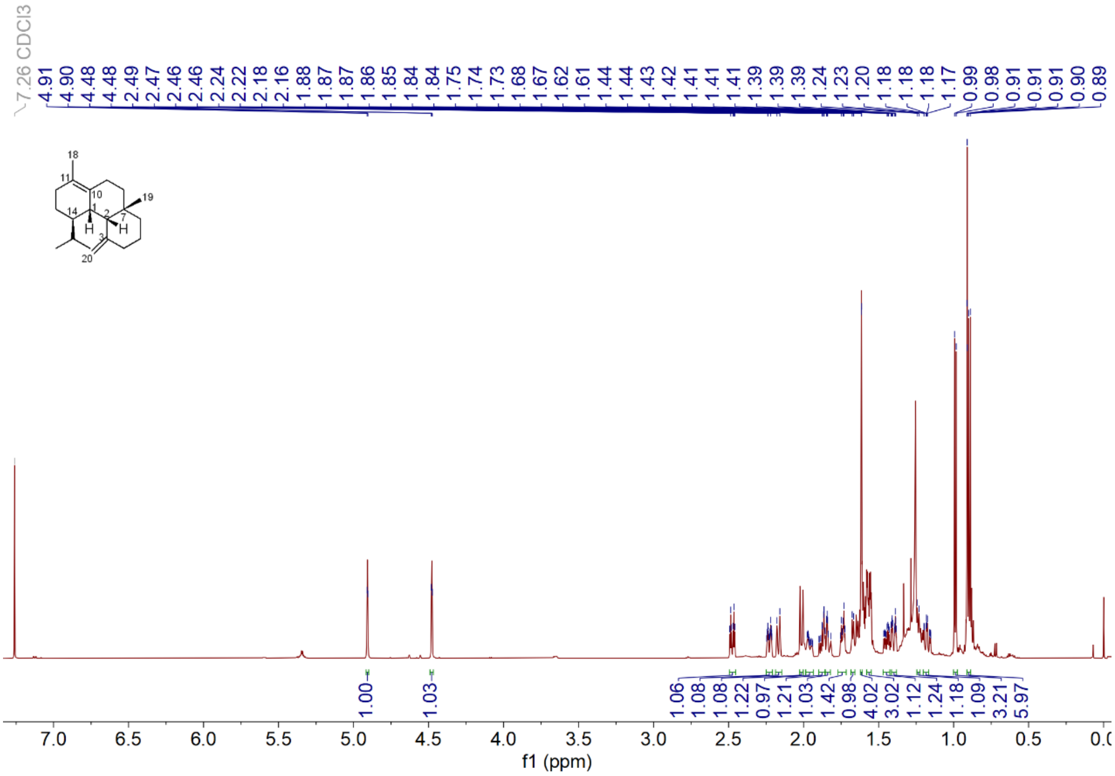

Supplementary Fig. 91. ^{1}H NMR spectrum (600 MHz) of **4** in $CDCl_3$.

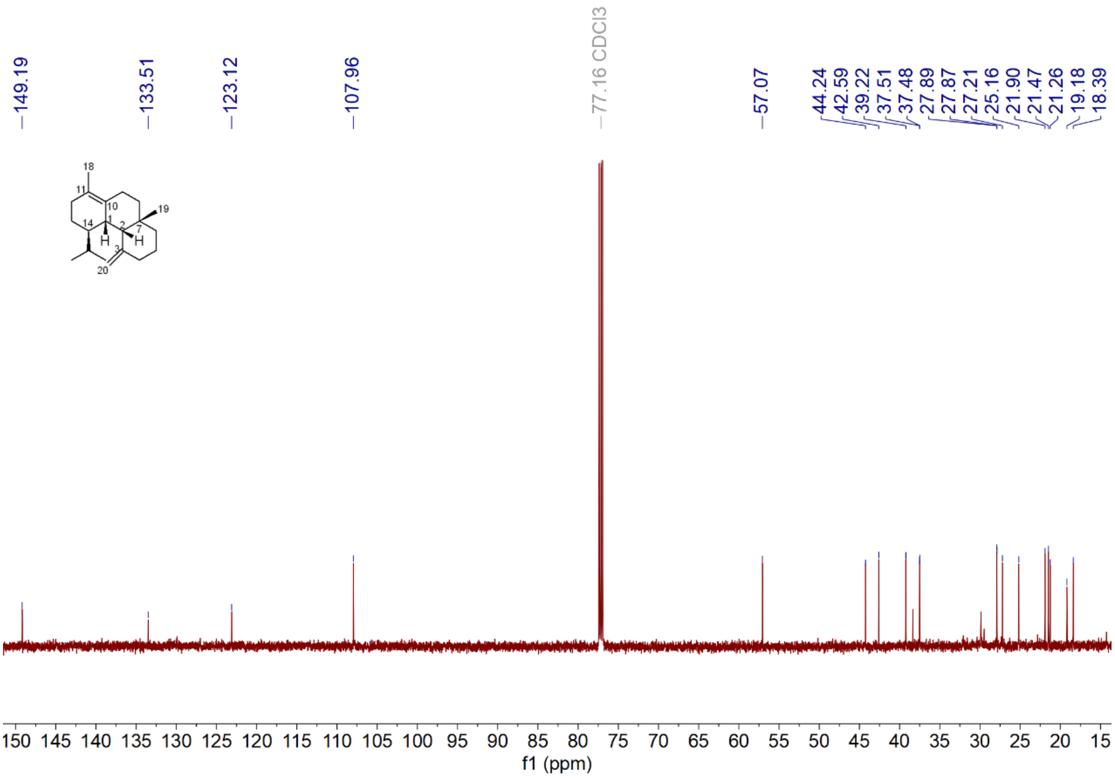

Supplementary Fig. 92. ^{13}C NMR spectrum (150 MHz) of **4** in $CDCl_3$.

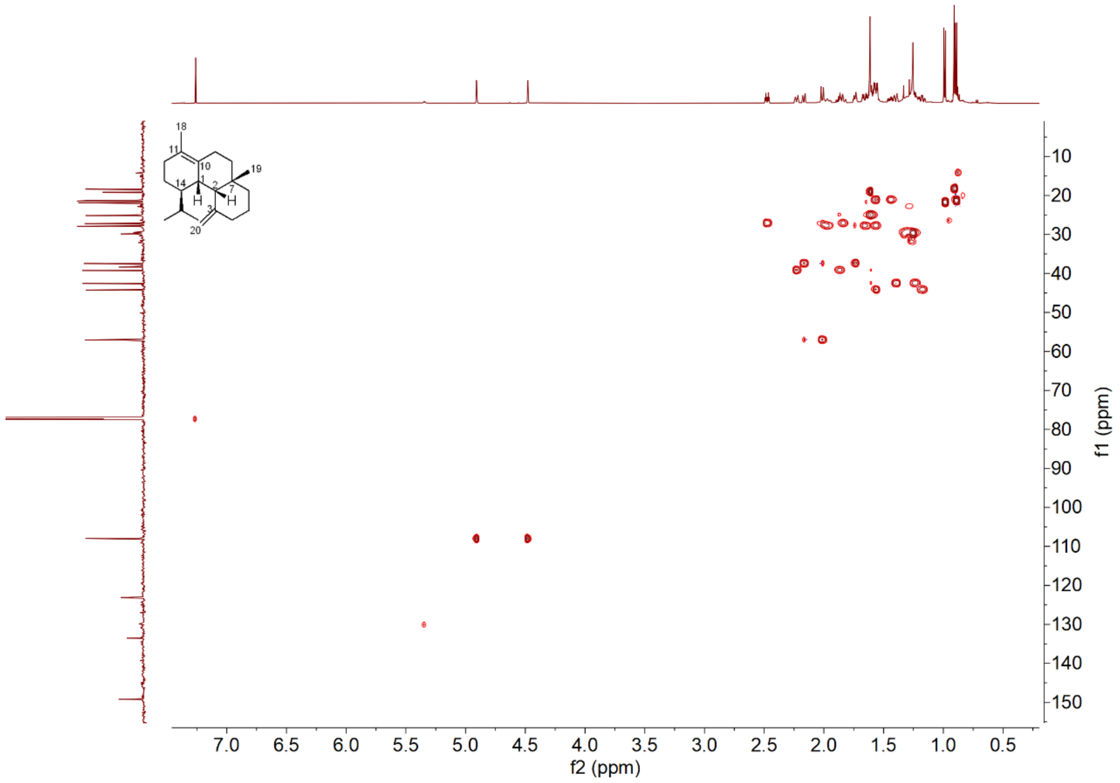

Supplementary Fig. 93. HSQC NMR spectrum of **4** in $CDCl_3$.

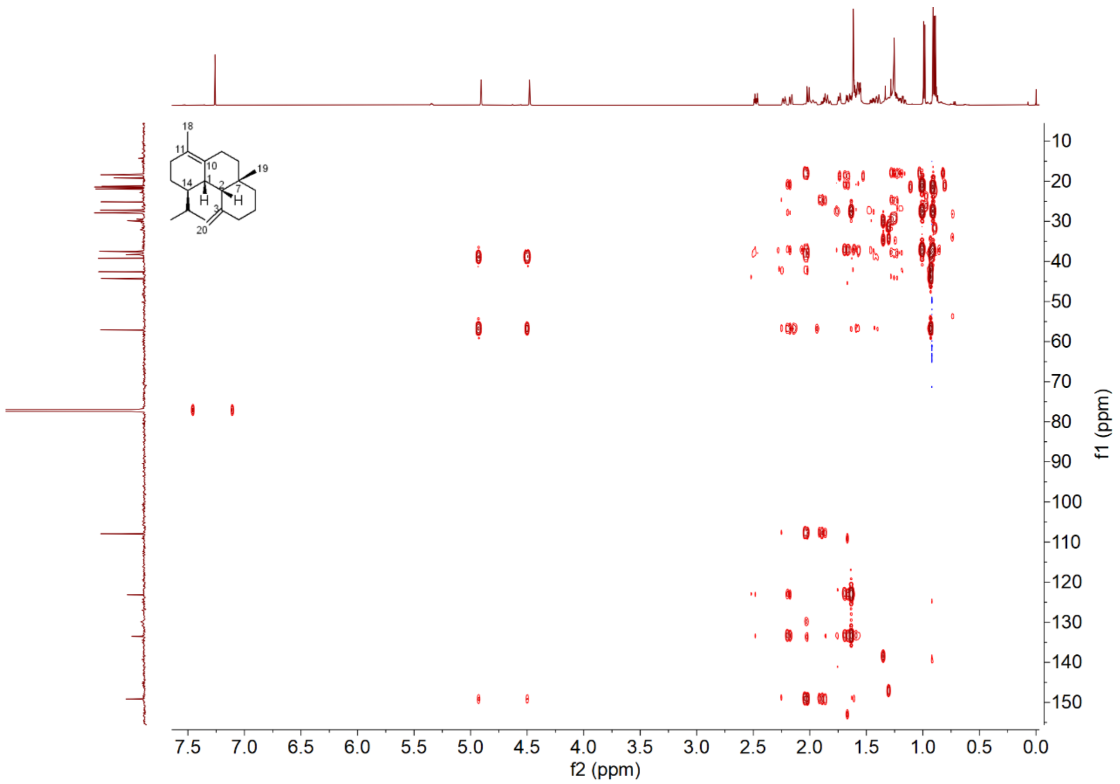

Supplementary Fig. 94. HMBC NMR spectrum of **4** in $CDCl_3$.

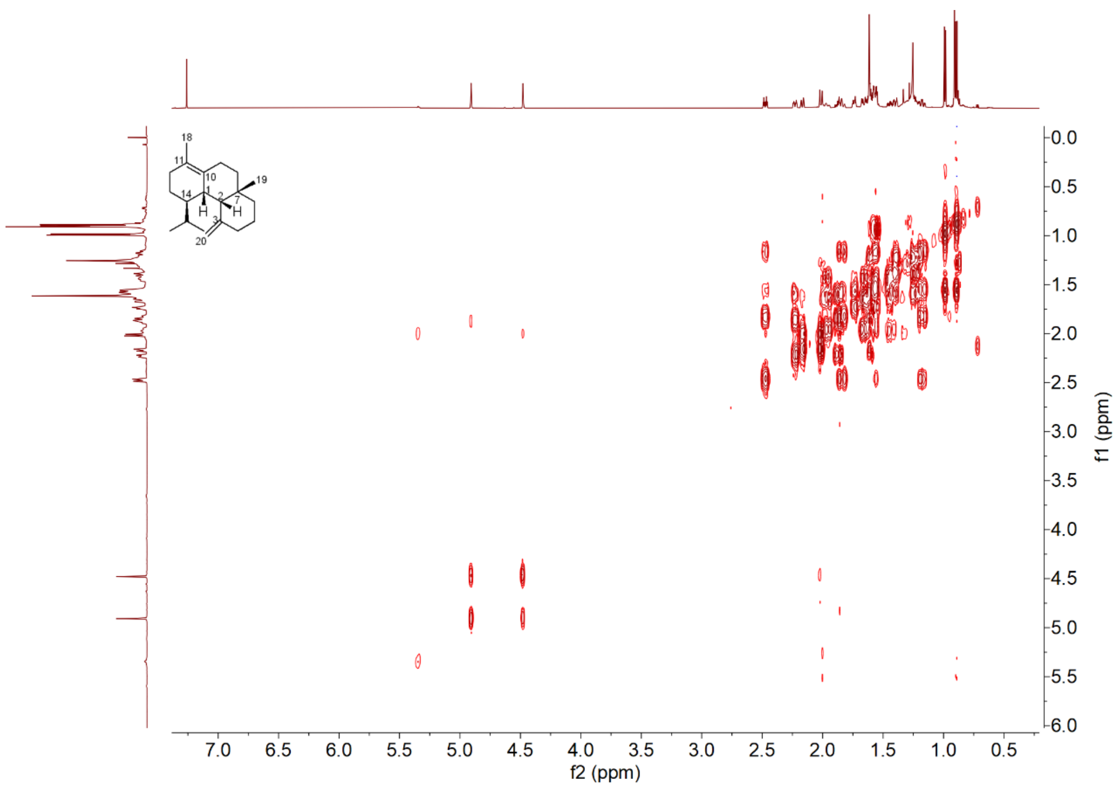

Supplementary Fig. 95. ^{1}H-^{1}H COSY NMR spectrum of **4** in $CDCl_3$.

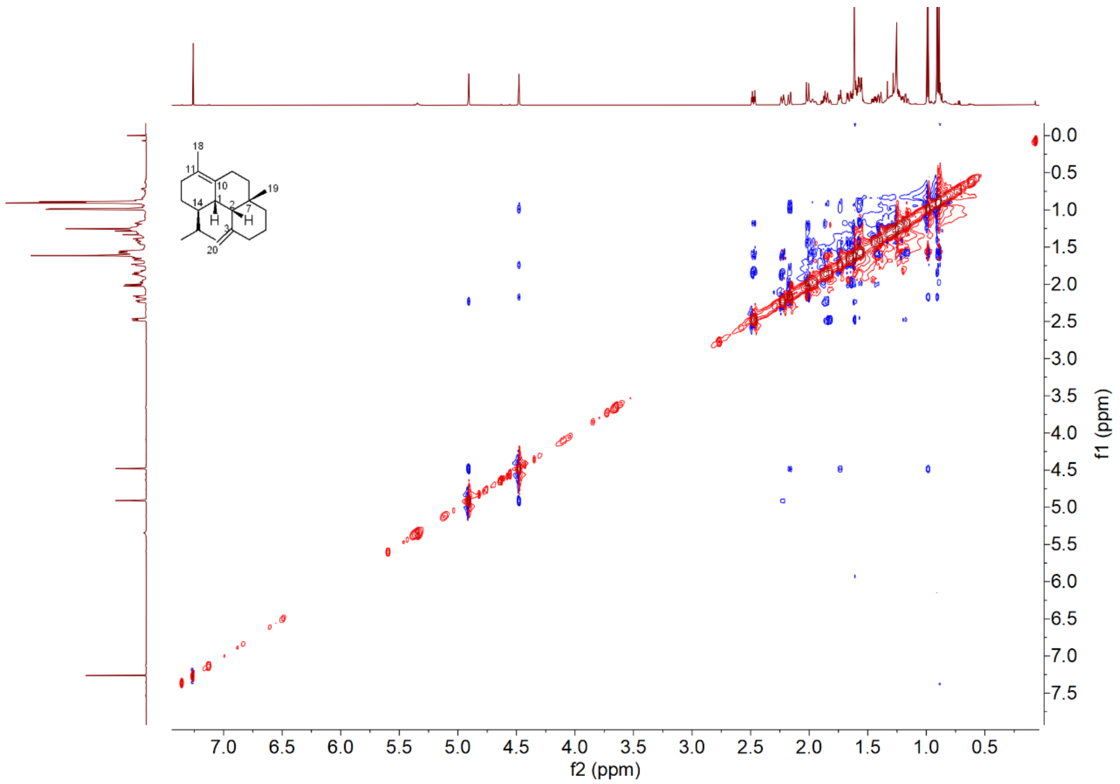

Supplementary Fig. 96. NOESY NMR spectrum of **4** in $CDCl_3$.

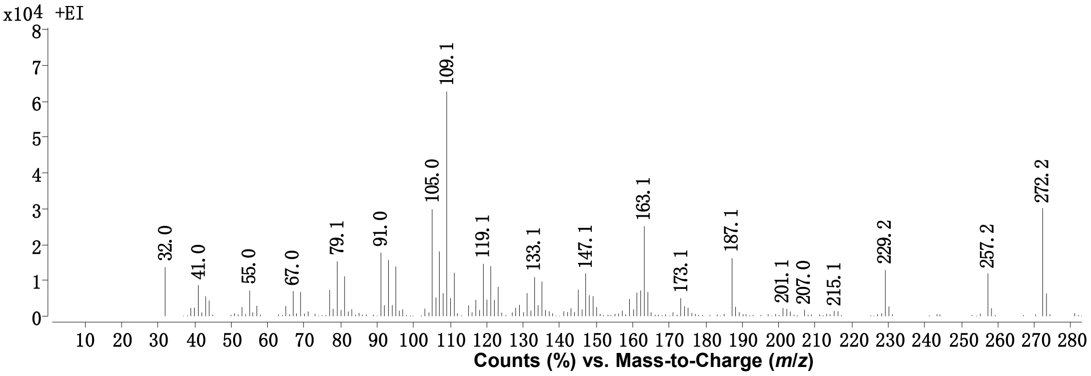

Supplementary Fig. 97. GC-MS spectra of **4**.

Original spectra for compound 5.

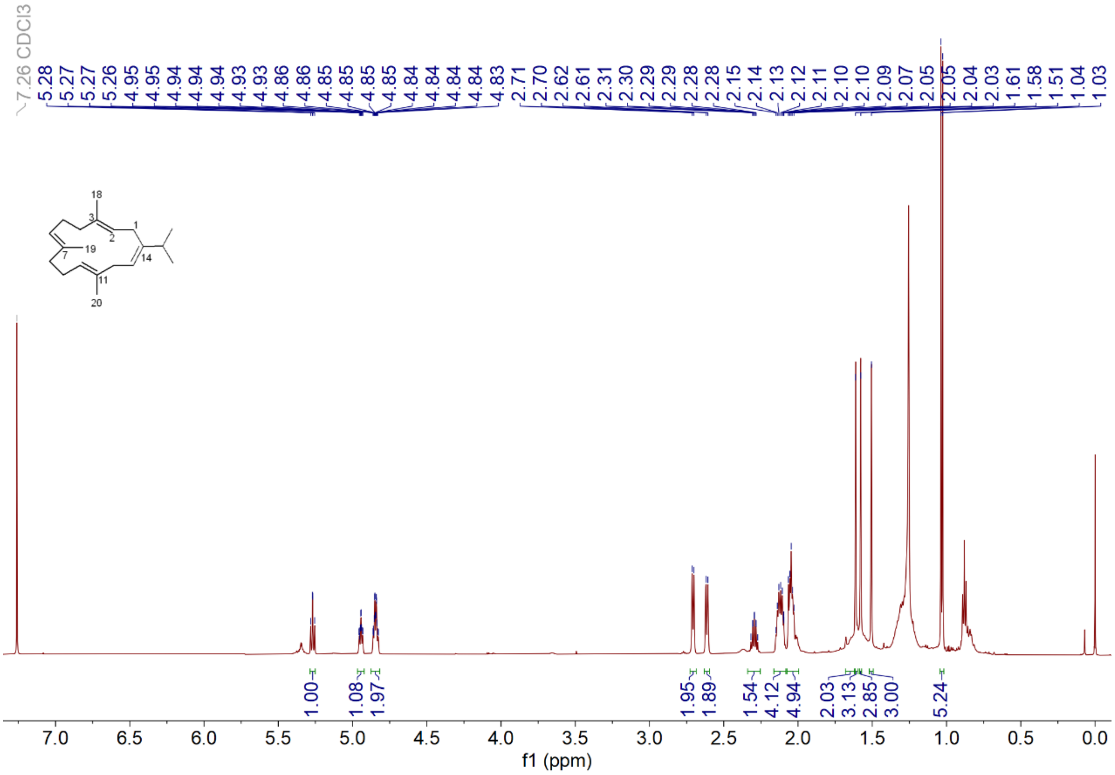

Supplementary Fig. 98. ^{1}H NMR spectrum (600 MHz) of **5** in $CDCl_3$.

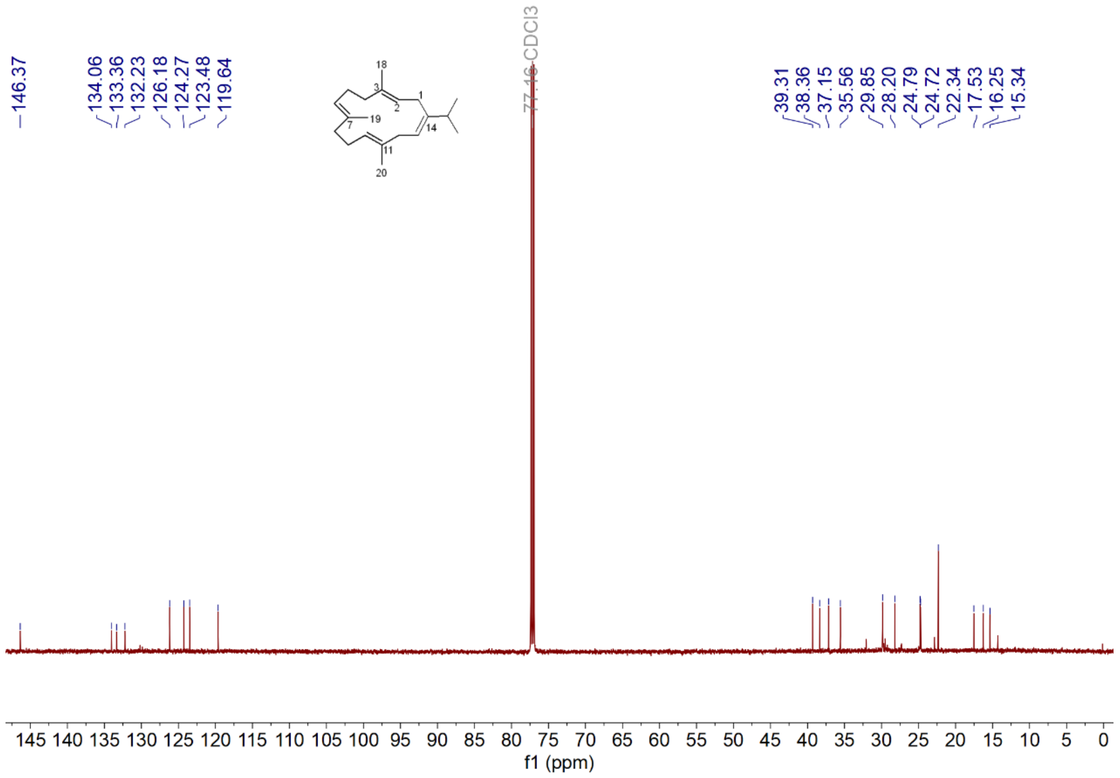

Supplementary Fig. 99. ^{13}C NMR spectrum (150 MHz) of **5** in $CDCl_3$.

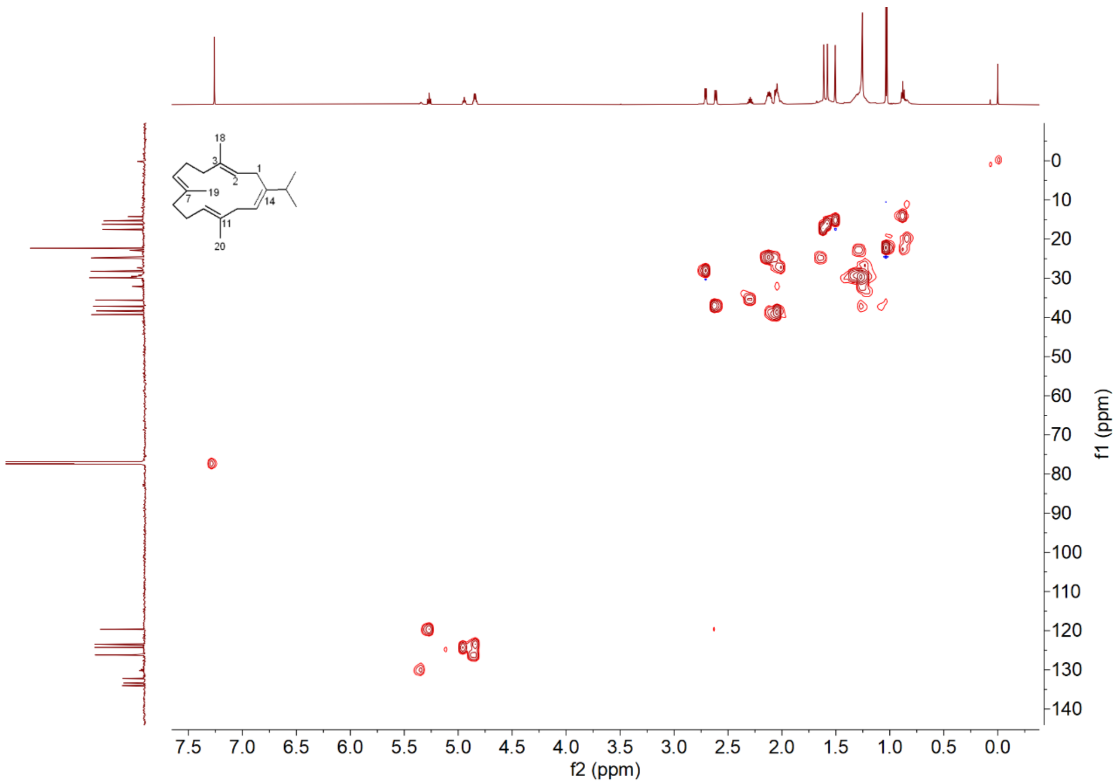

Supplementary Fig. 100. HSQC NMR spectrum of **5** in $CDCl_3$.

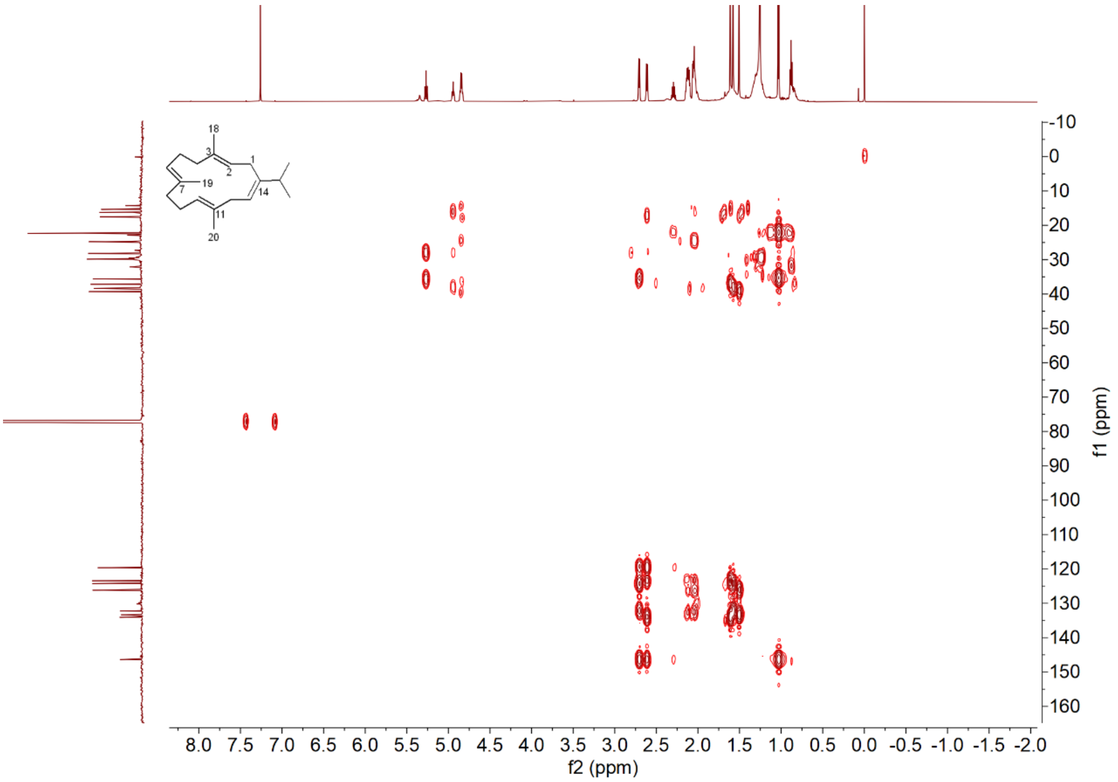

Supplementary Fig. 101. HMBC NMR spectrum of **5** in $CDCl_3$.

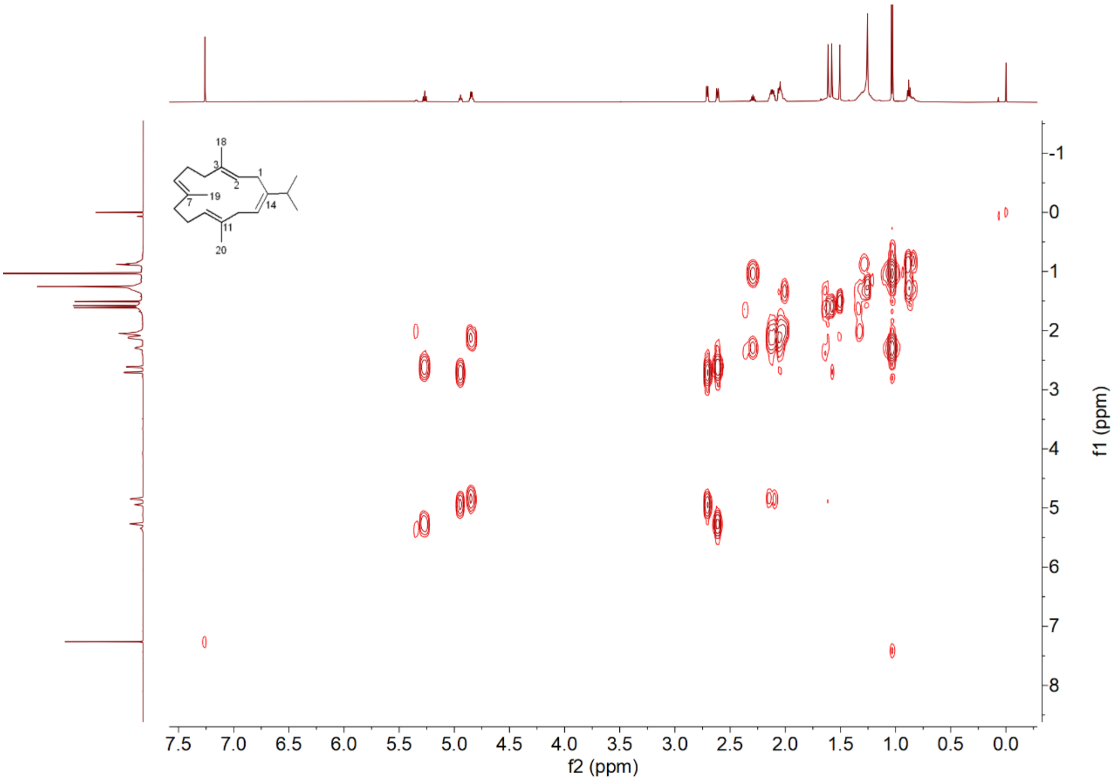

Supplementary Fig. 102. ^1H-^1H COSY NMR spectrum of **5** in $CDCl_3$.

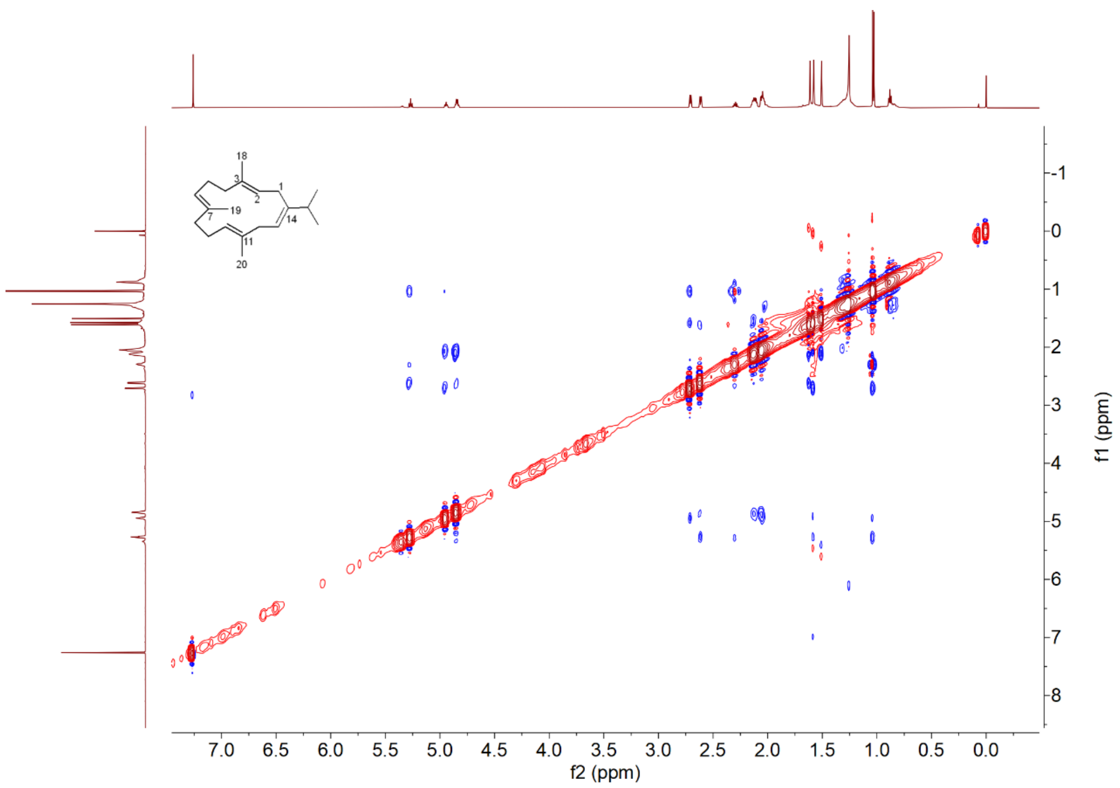

Supplementary Fig. 103. NOESY NMR spectrum of **5** in $CDCl_3$.

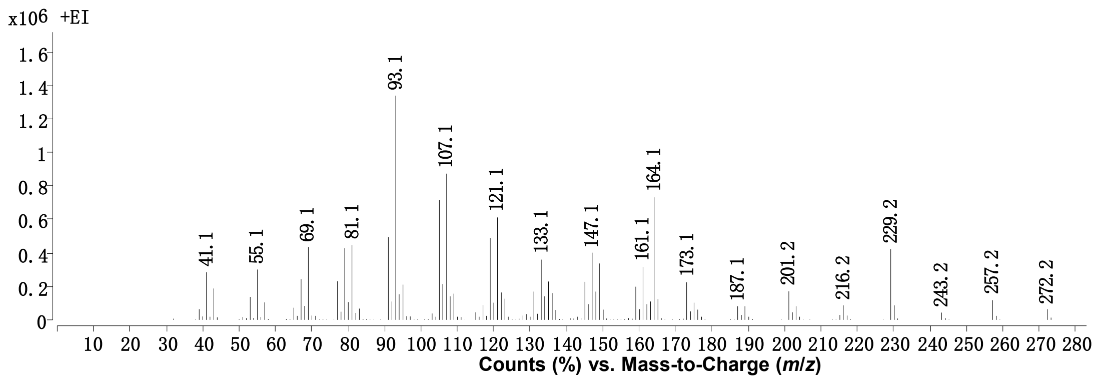

Supplementary Fig. 104. GC-MS spectra of **5**.

Original spectra for compound 1 in C_6D_6.

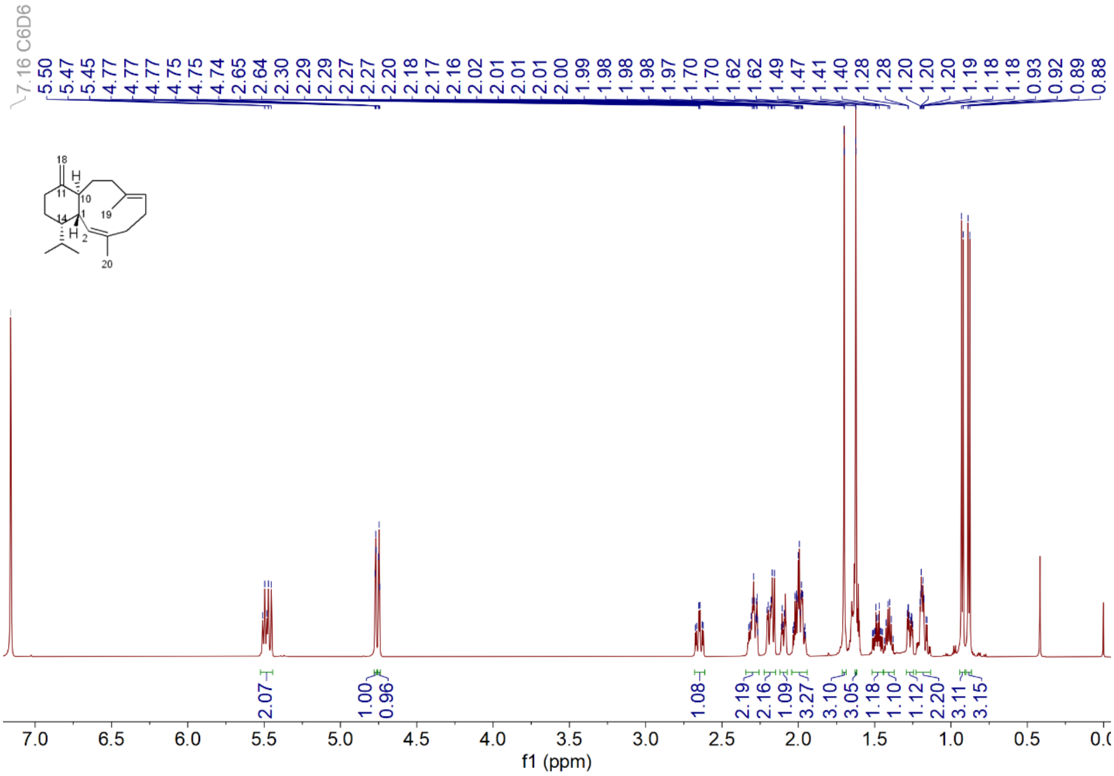

Supplementary Fig. 105. ^{1}H NMR spectrum (600 MHz) of **1** in C_6D_6.

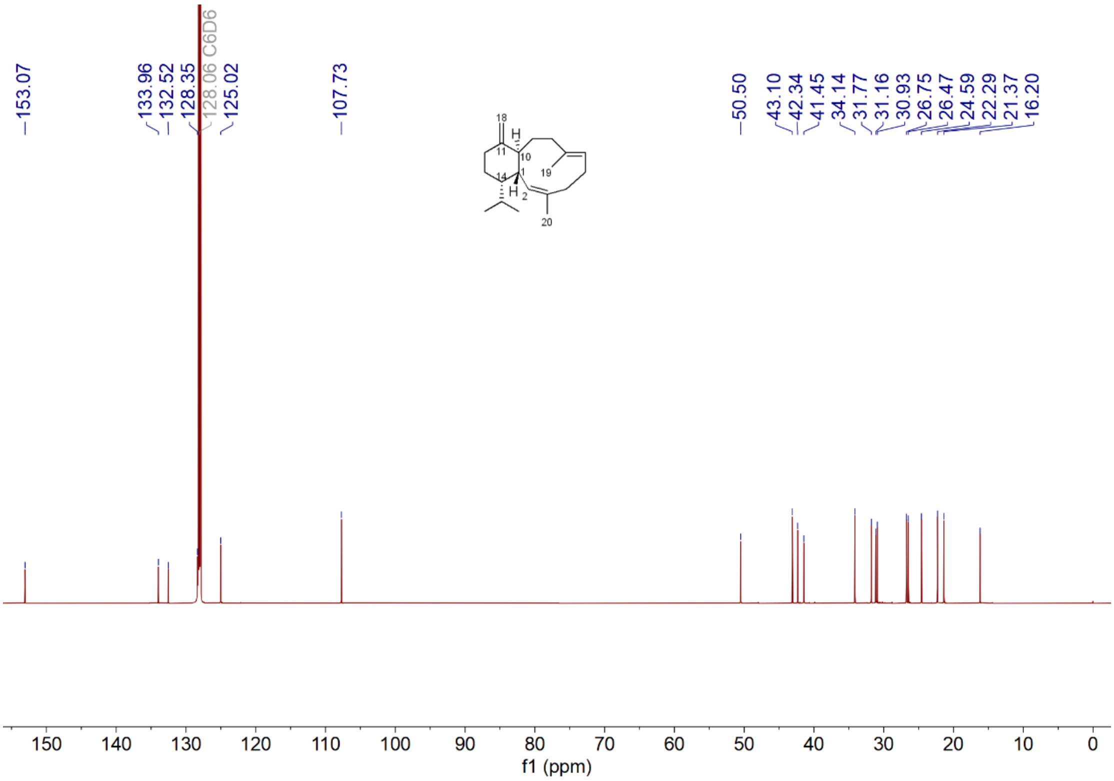

Supplementary Fig. 106. ^{13}C NMR spectrum (150 MHz) of **1** in C_6D_6.

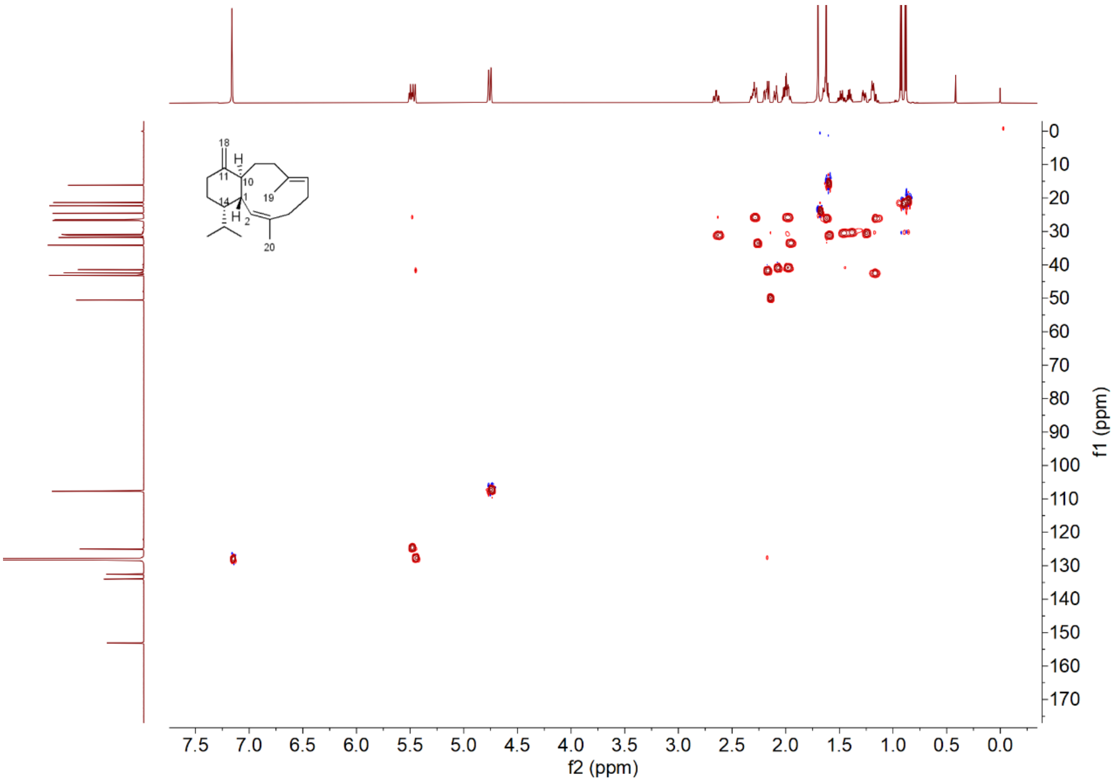

Supplementary Fig. 107. HSQC NMR spectrum of **1** in C_6D_6.

Original spectra for compound 6.

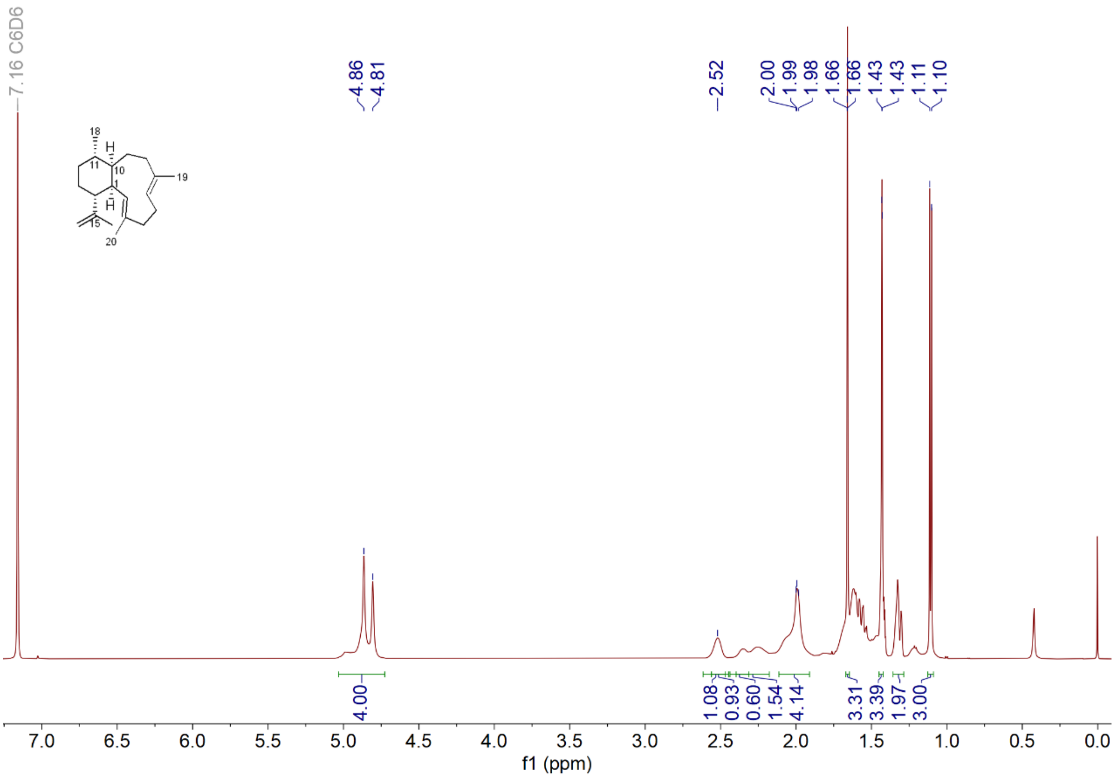

Supplementary Fig. 108. ^{1}H NMR spectrum (600 MHz) of **6** in C_6D_6.

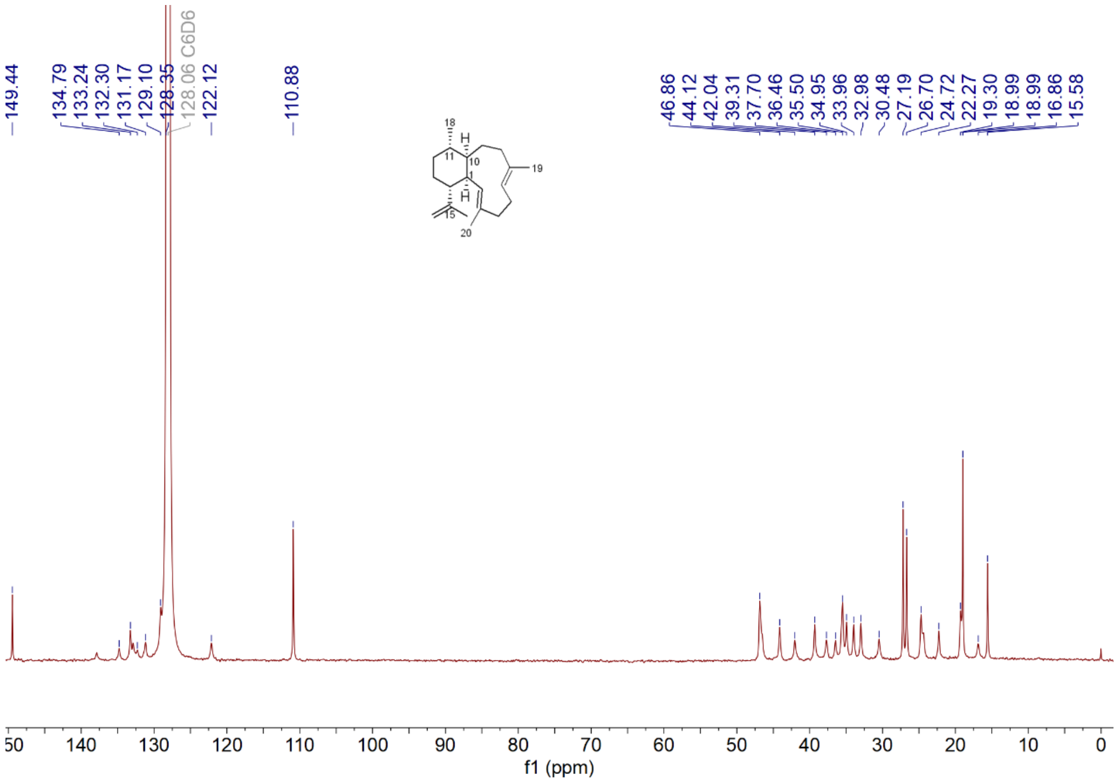

Supplementary Fig. 109. ^{13}C NMR spectrum (150 MHz) of **6** in C_6D_6.

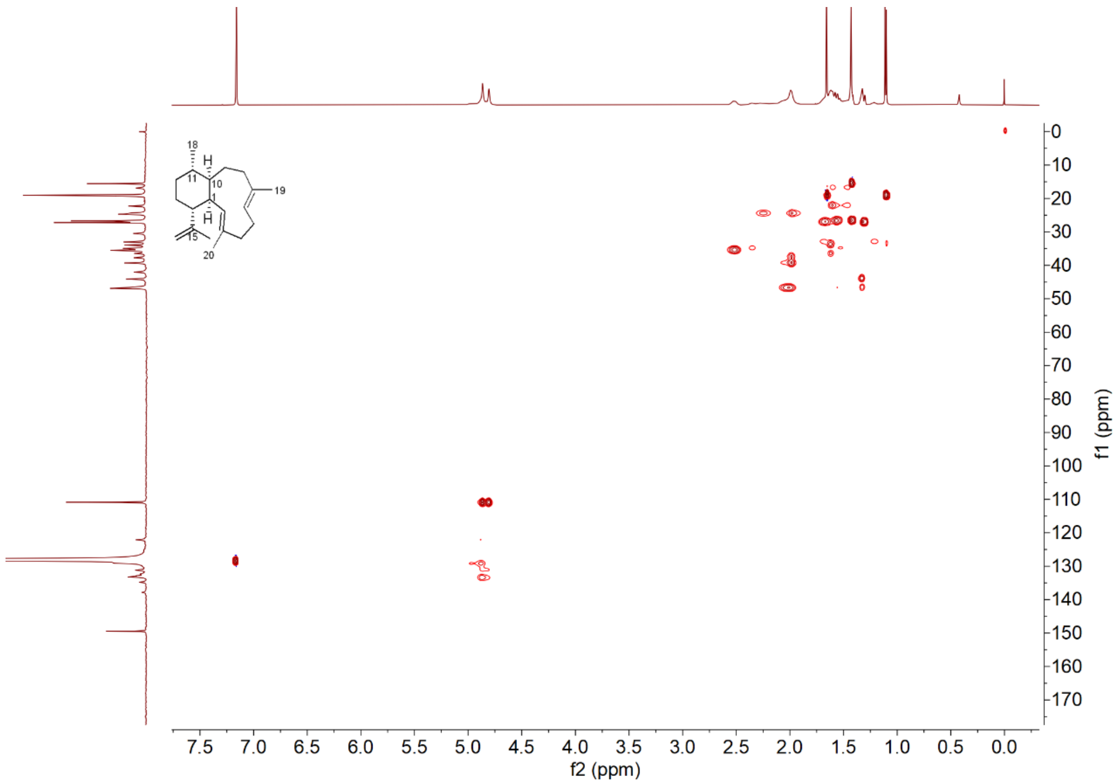

Supplementary Fig. 110. HSQC NMR spectrum of **6** in C_6D_6.

Original spectra for compound 7.

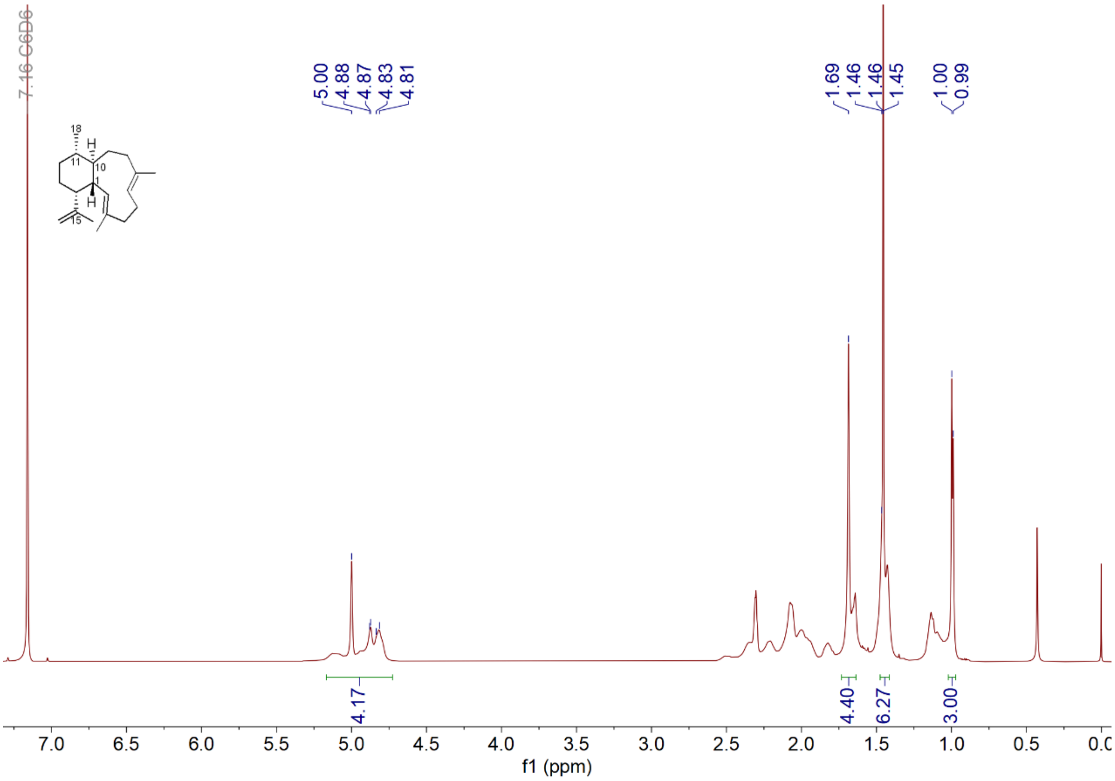

Supplementary Fig. 111. ^{1}H NMR spectrum (600 MHz) of **7** in C_6D_6.

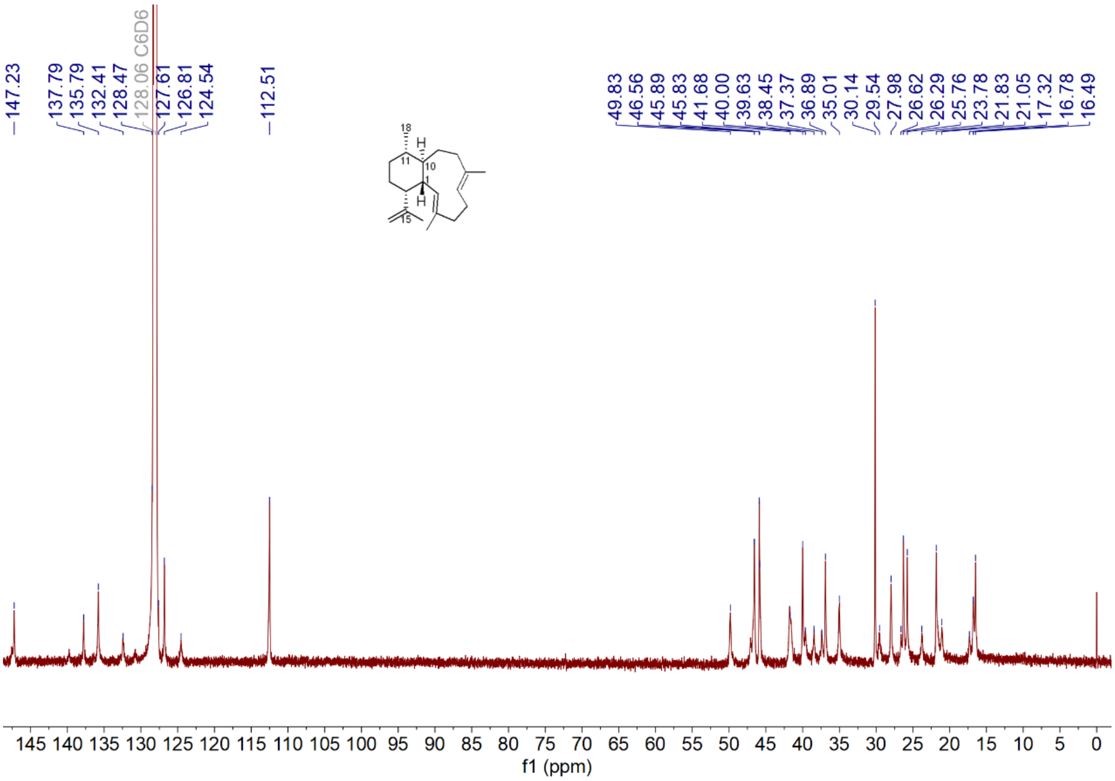

Supplementary Fig. 112. ^{13}C NMR spectrum (150 MHz) of **7** in C_6D_6.

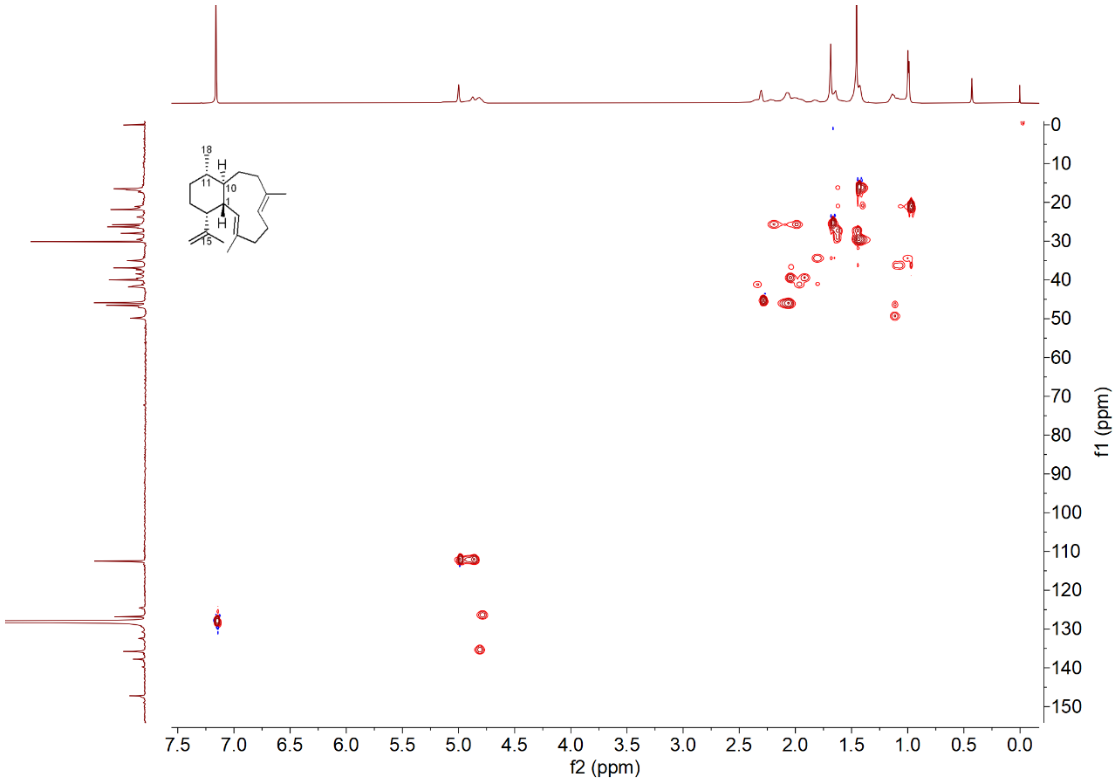

Supplementary Fig. 113. HSQC NMR spectrum of **7** in C_6D_6.

Original spectra for compound 8.

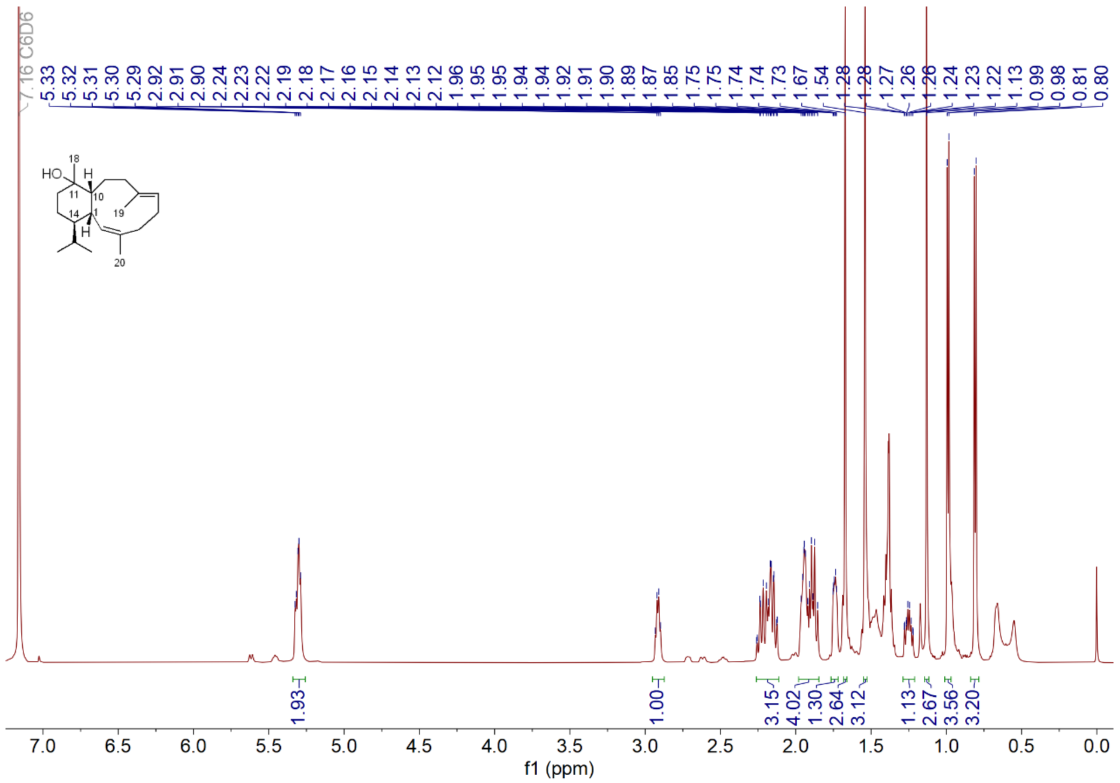

Supplementary Fig. 114. ^{1}H NMR spectrum (600 MHz) of **8** in C_6D_6.

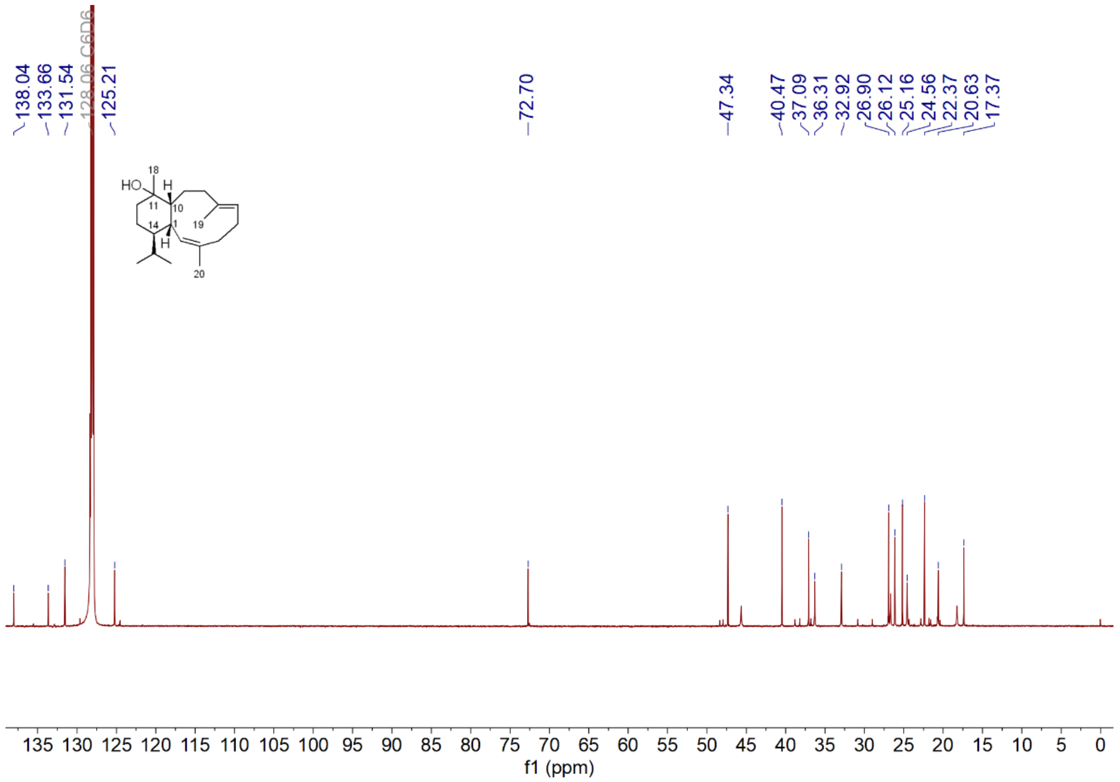

Supplementary Fig. 115. ^{13}C NMR spectrum (150 MHz) of **8** in C_6D_6.

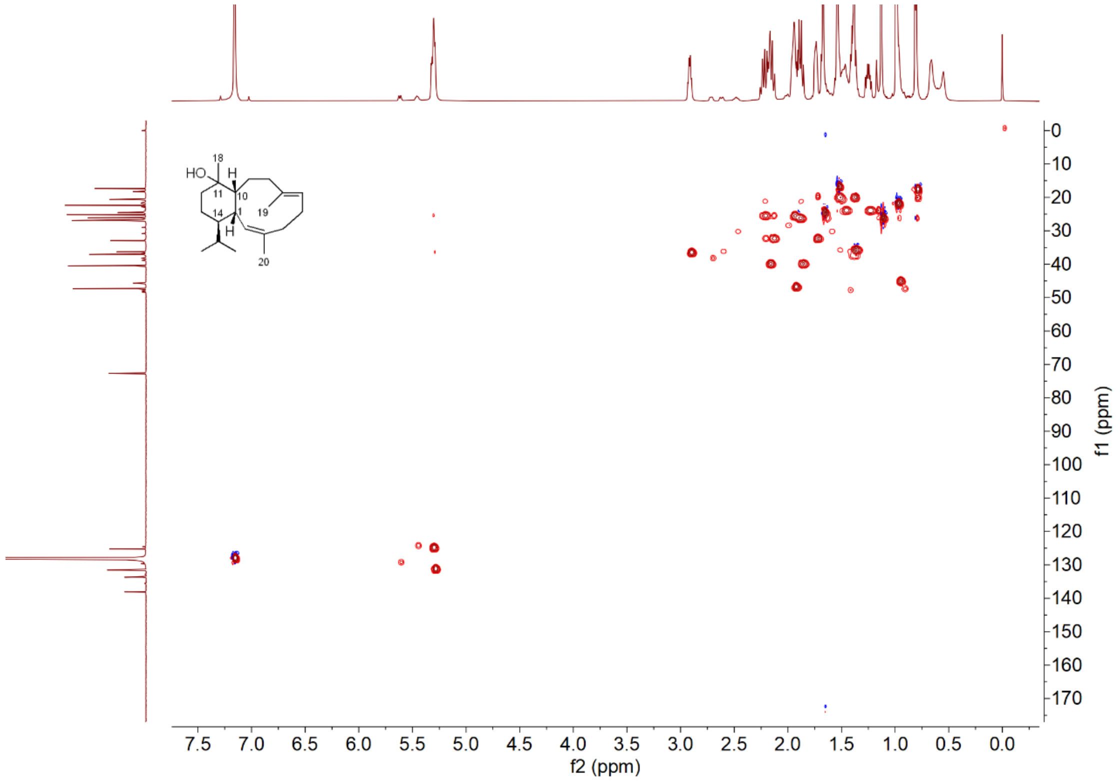

Supplementary Fig. 116. HSQC NMR spectrum of **8** in C_6D_6.

Original spectra for compound 9.

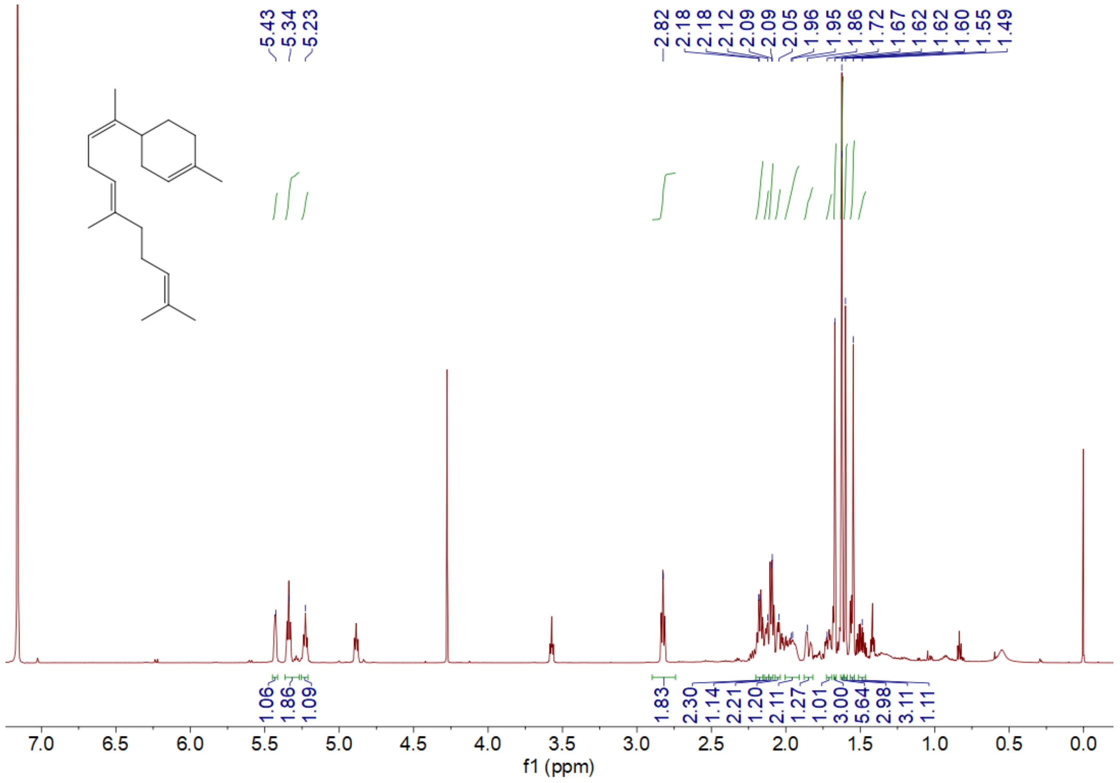

Supplementary Fig. 117. ^{1}H NMR spectrum (600 MHz) of **9** in C_6D_6.

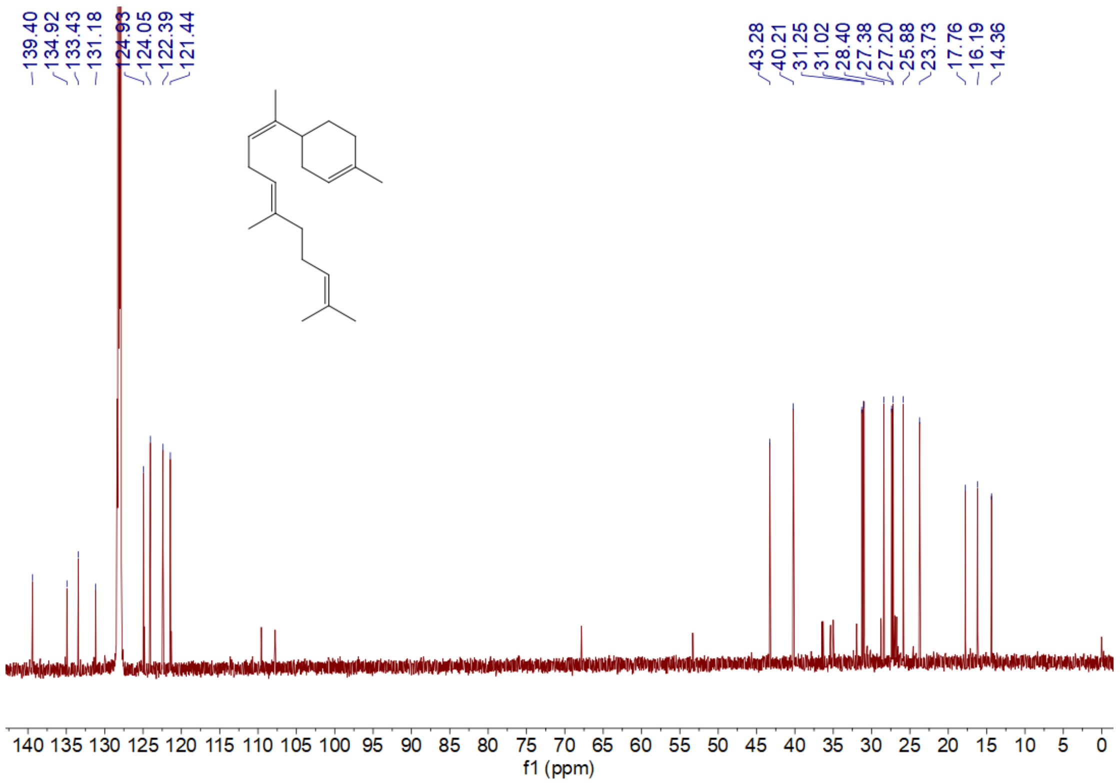

Supplementary Fig. 118. ^{13}C NMR spectrum (150 MHz) of **9** in C_6D_6.

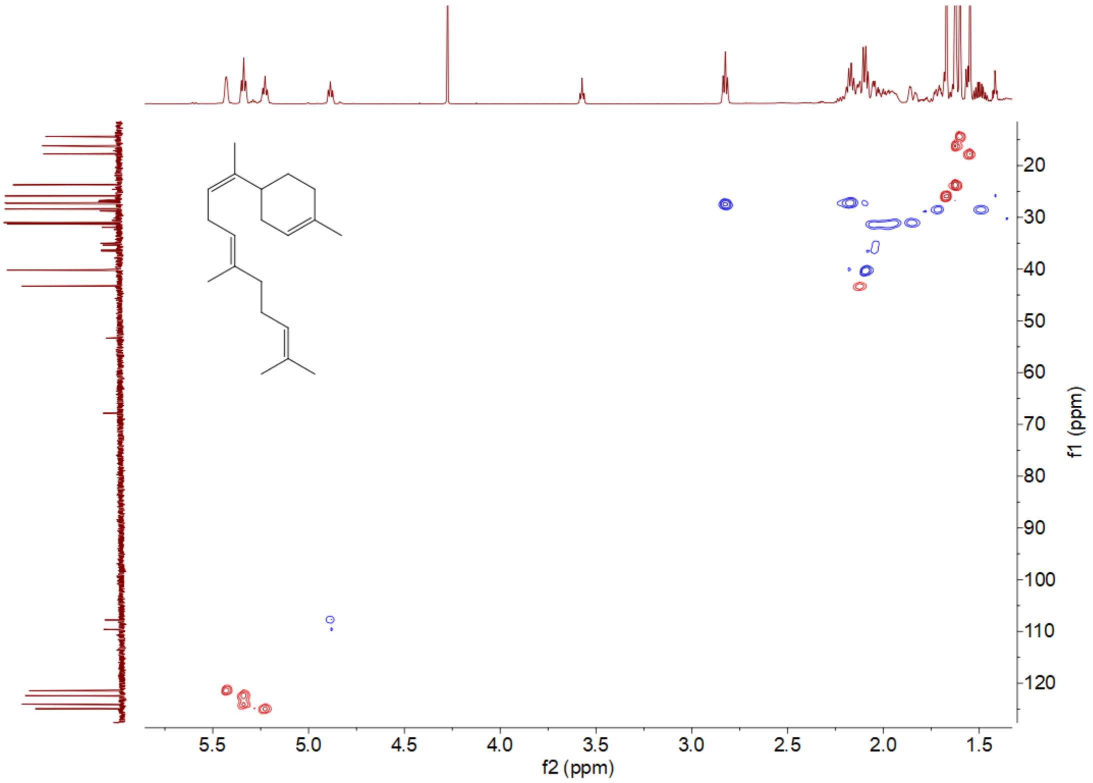

Supplementary Fig. 119. HSQC NMR spectrum of **9** in C_6D_6.

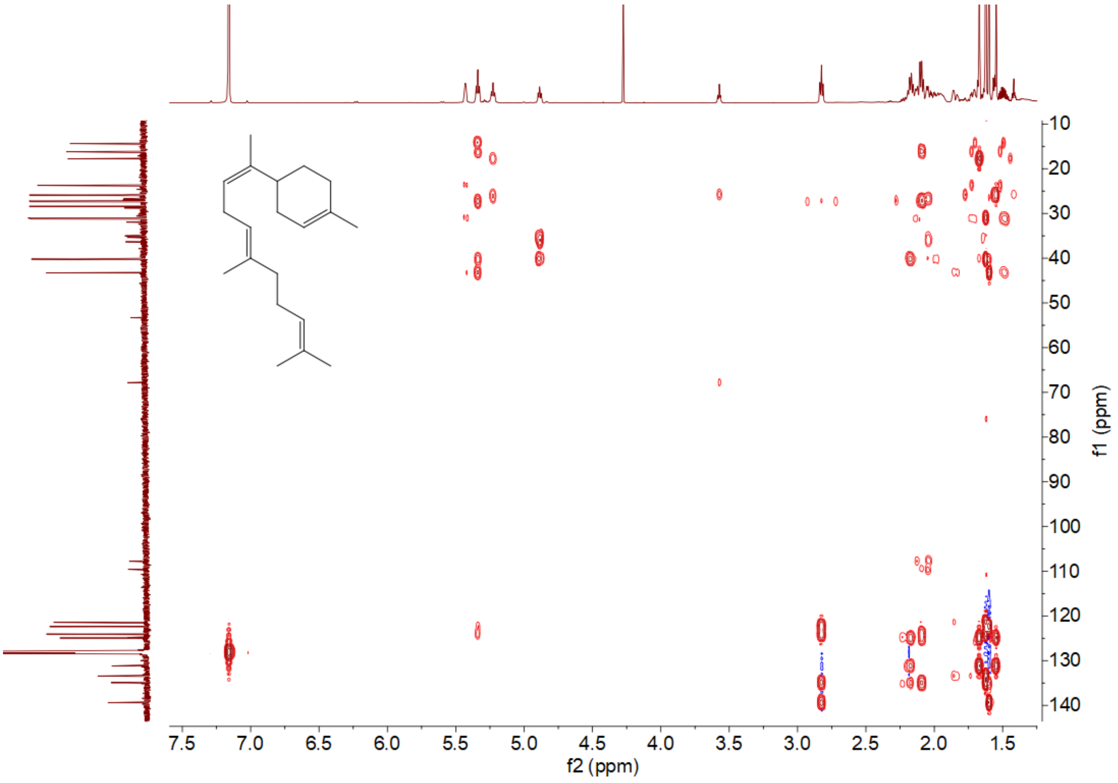

Supplementary Fig. 120. HMBC NMR spectrum of **9** in C_6D_6.

Original spectra for compound 10.

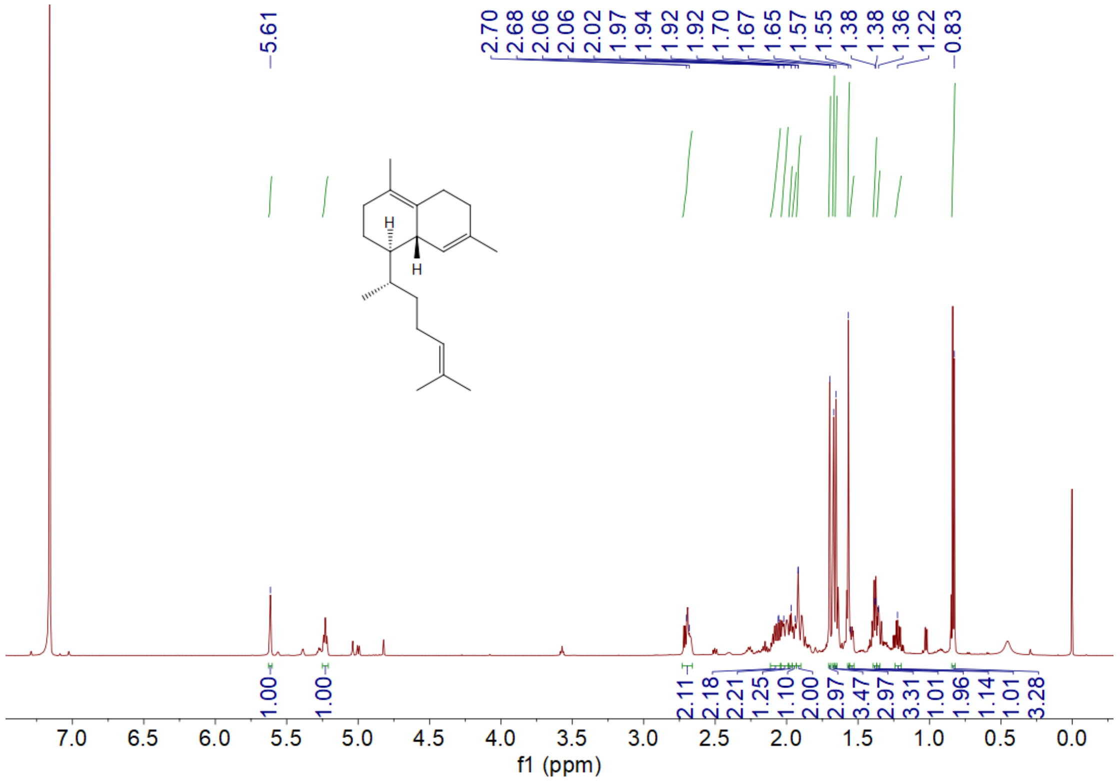

Supplementary Fig. 121. ^{1}H NMR spectrum (600 MHz) of **10** in C_6D_6.

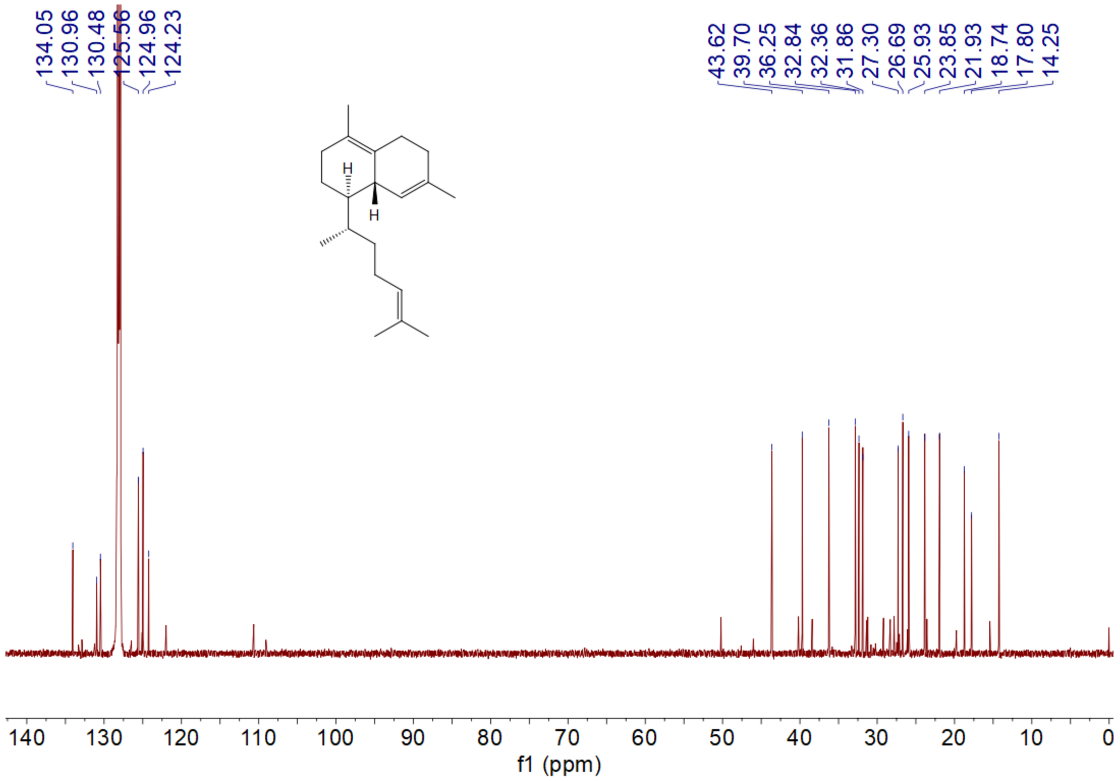

Supplementary Fig. 122. ^{13}C NMR spectrum (150 MHz) of **10** in C_6D_6.

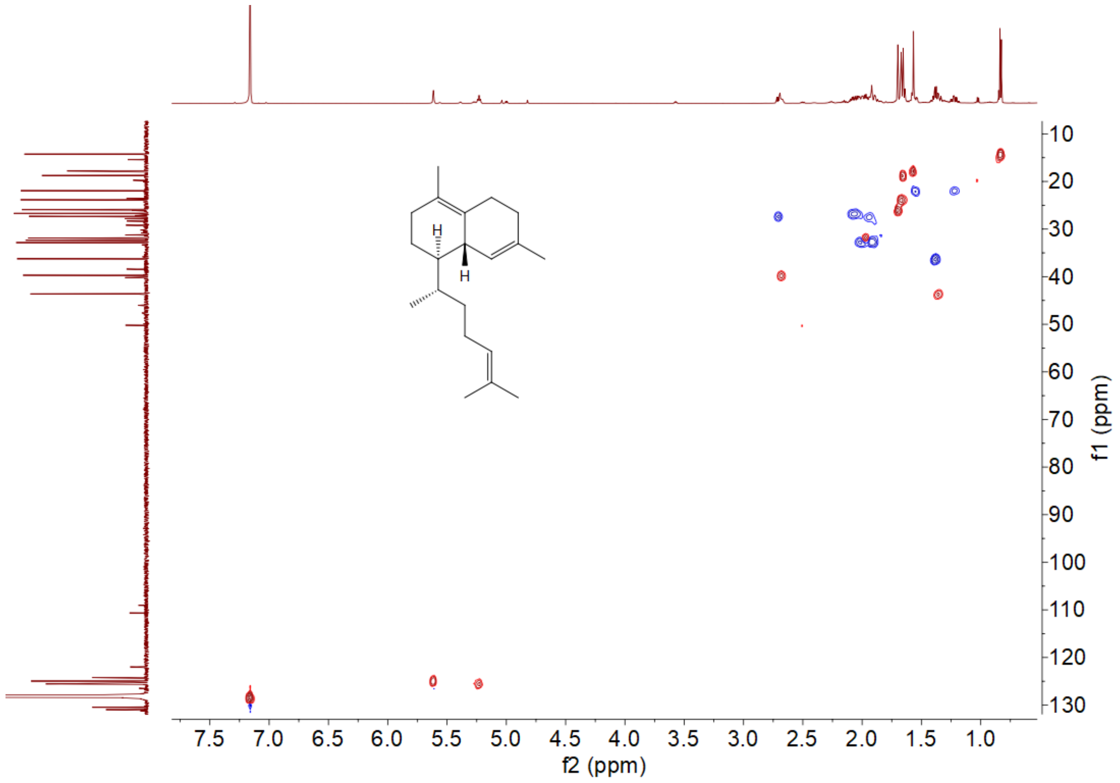

Supplementary Fig. 123. HSQC NMR spectrum of **10** in C_6D_6.

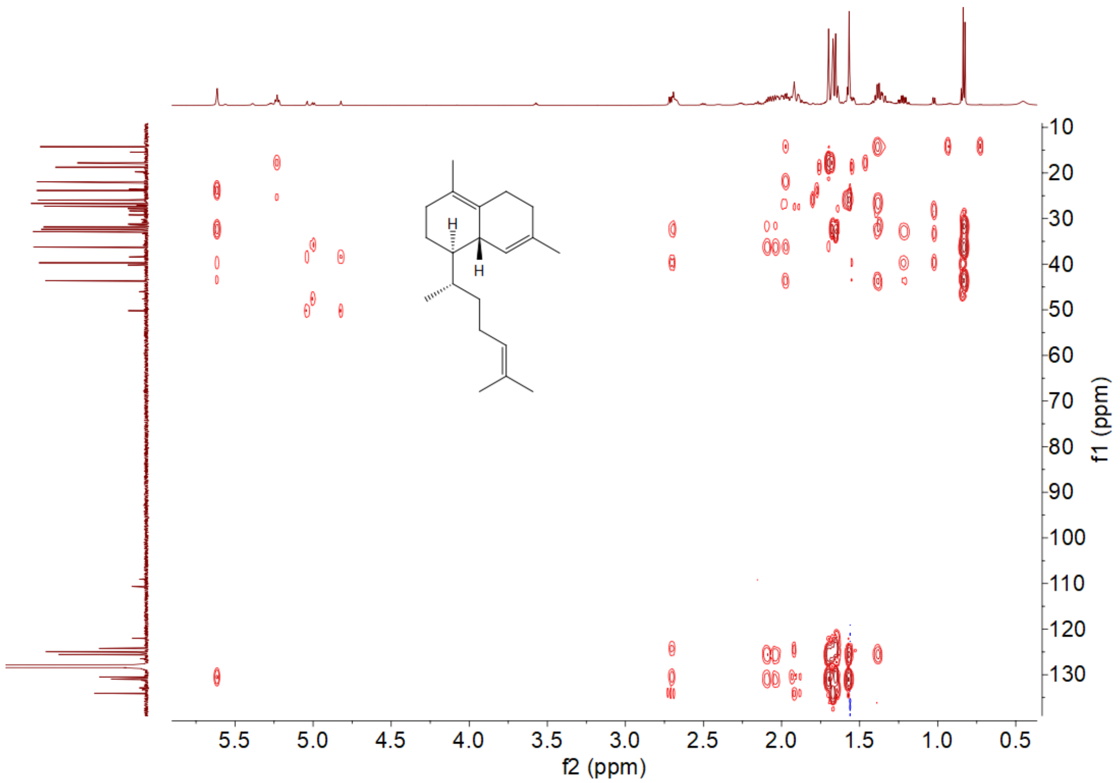

Supplementary Fig. 124. HMBC NMR spectrum of **10** in C_6D_6.

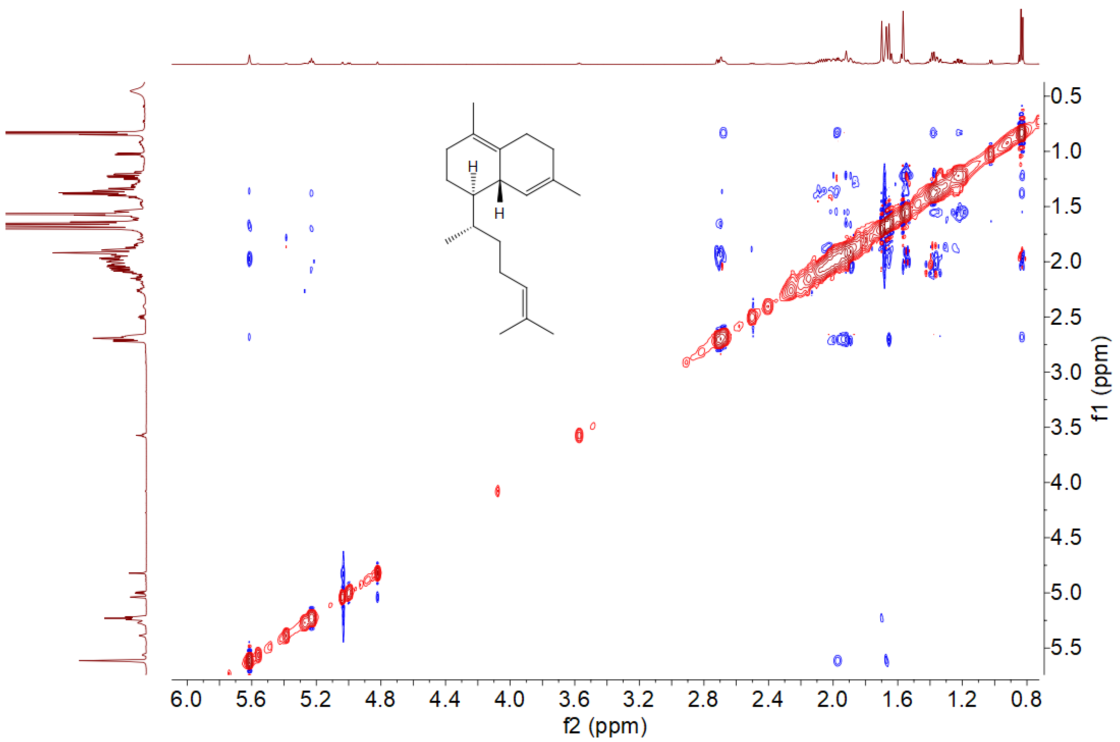

Supplementary Fig. 125. NOESY NMR spectrum of **10** in C_6D_6.

Original spectra for compound 11.

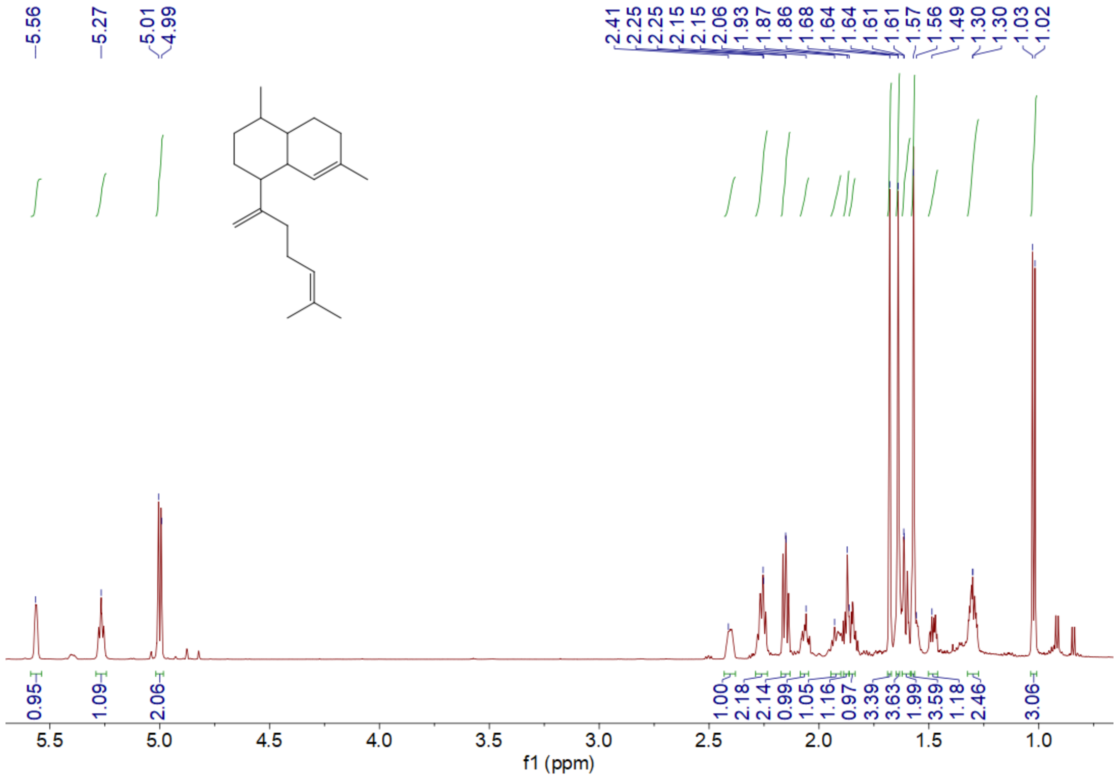

Supplementary Fig. 126. ^{1}H NMR spectrum (600 MHz) of **11** in C_6D_6.

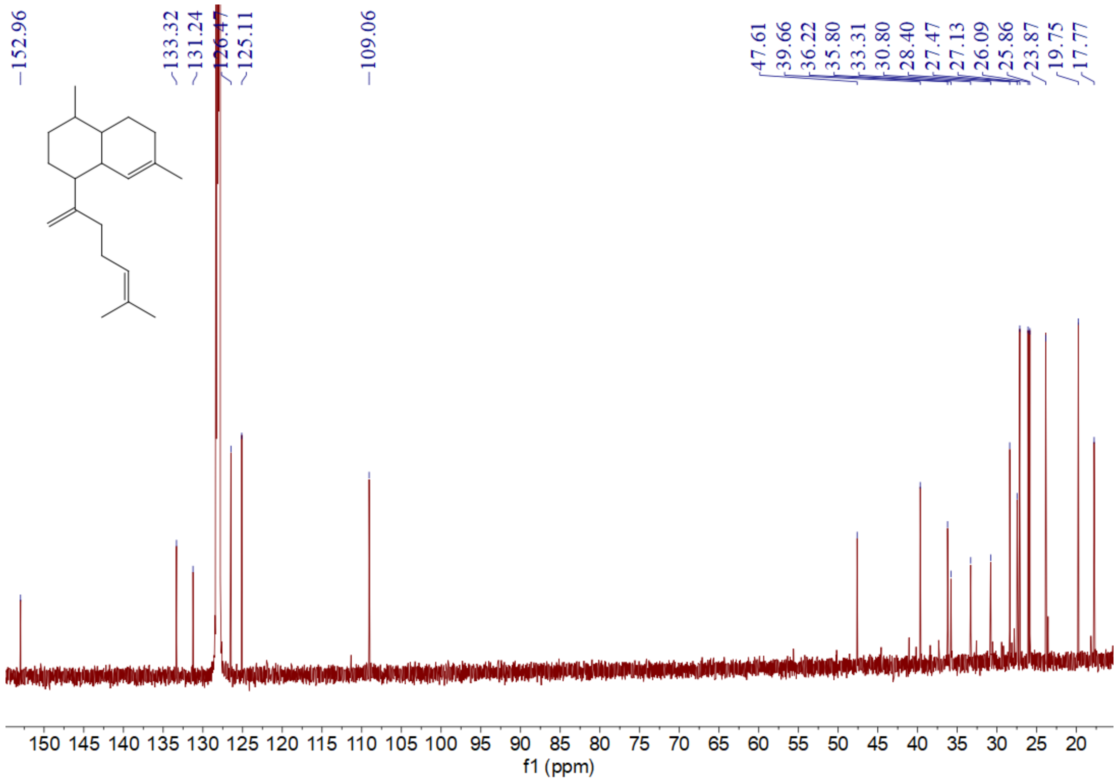

Supplementary Fig. 127. ^{13}C NMR spectrum (150 MHz) of **11** in C_6D_6.

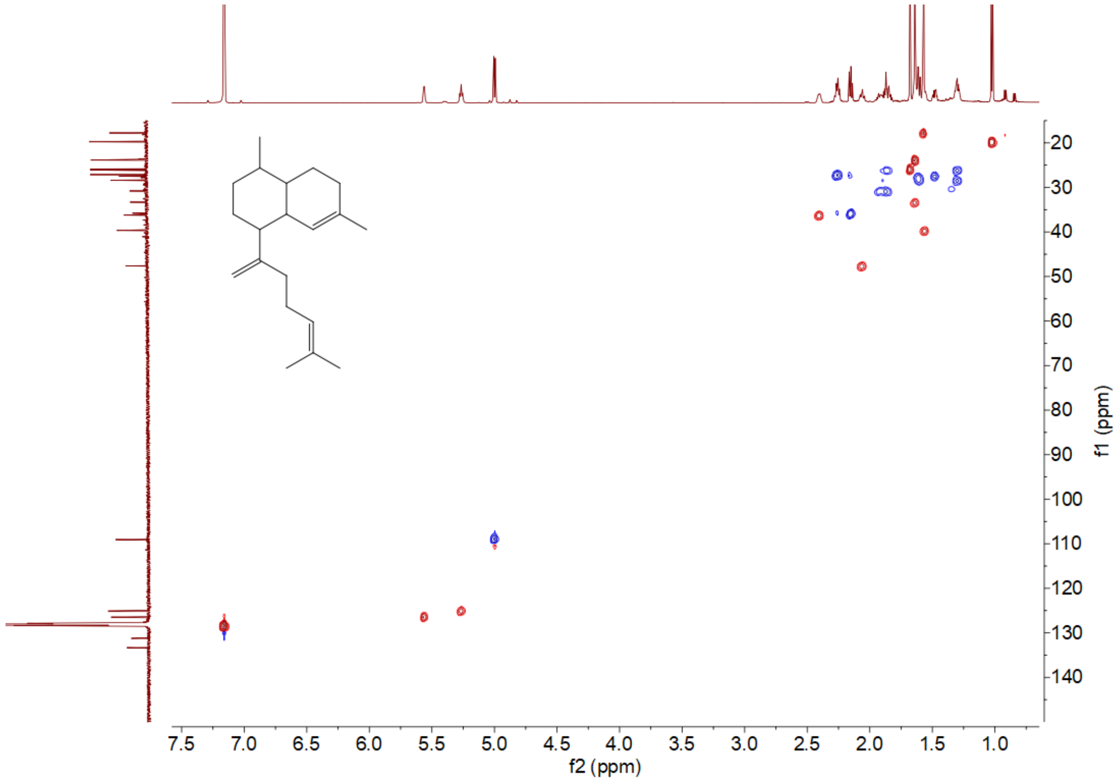

Supplementary Fig. 128. HSQC NMR spectrum of **11** in C_6D_6.

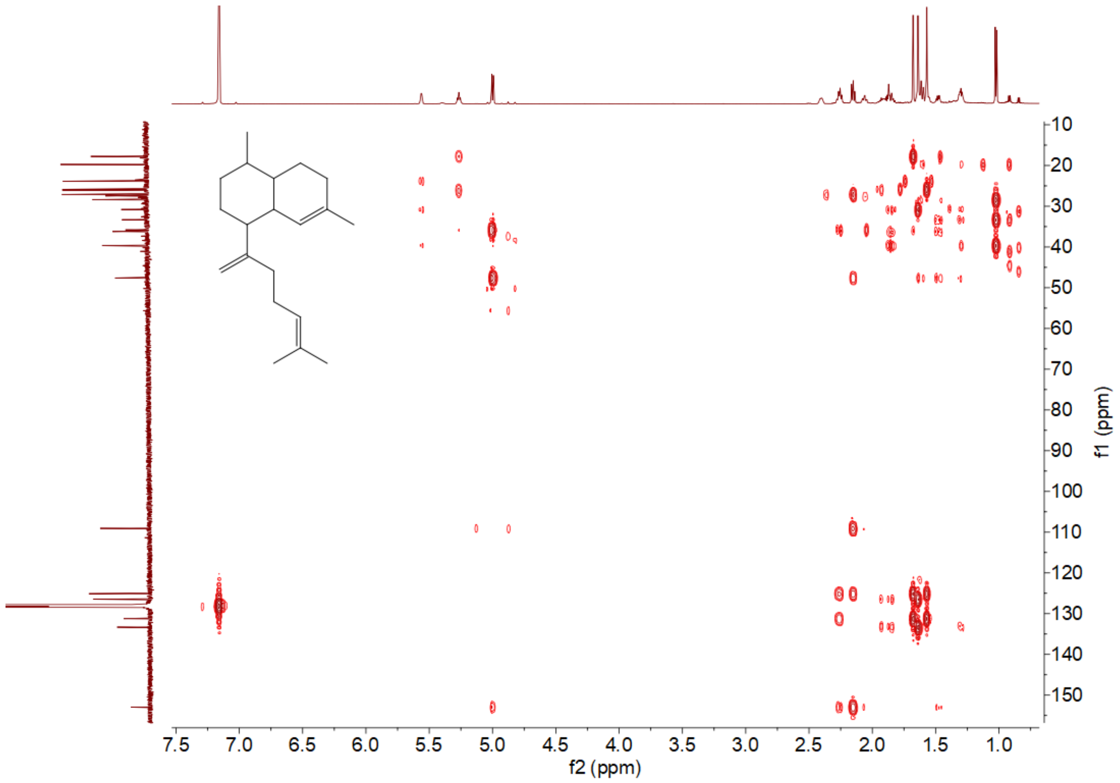

Supplementary Fig. 129. HMBC NMR spectrum of **11** in C_6D_6.

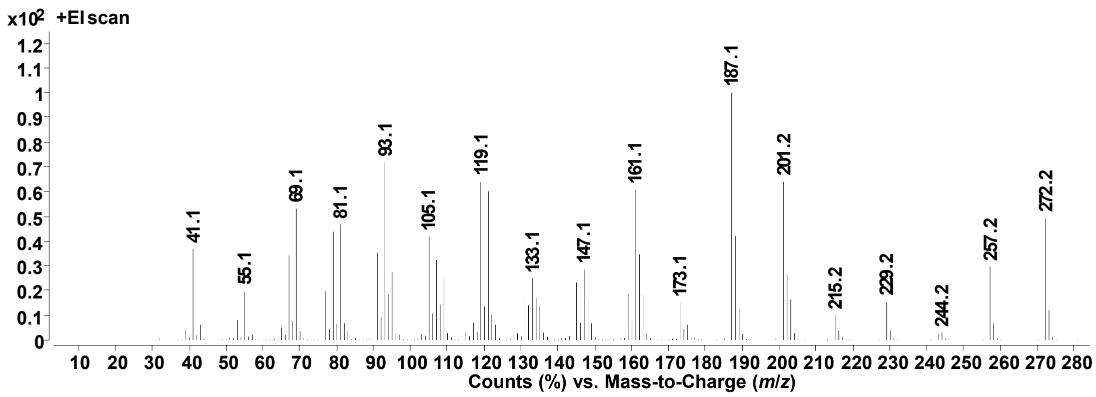

Supplementary Fig. 130. GC-MS spectra of **11**.

Original spectra for compound 12.

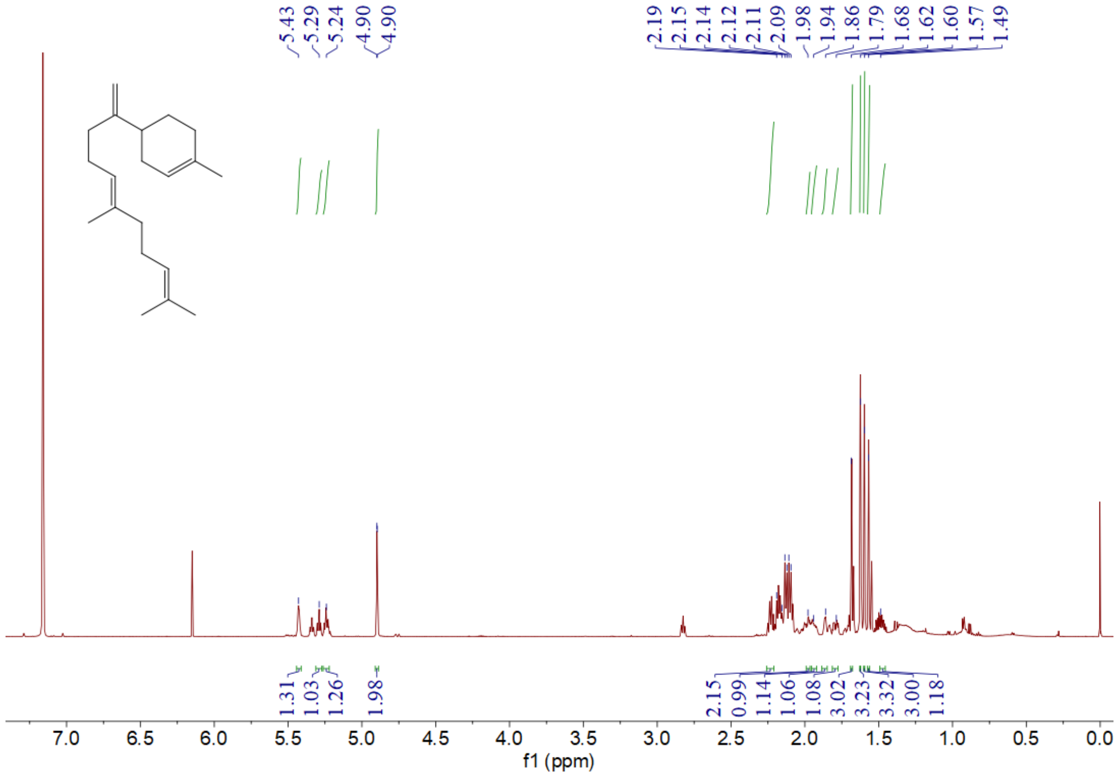

Supplementary Fig. 131. ^{1}H NMR spectrum (600 MHz) of **12** in C_6D_6.

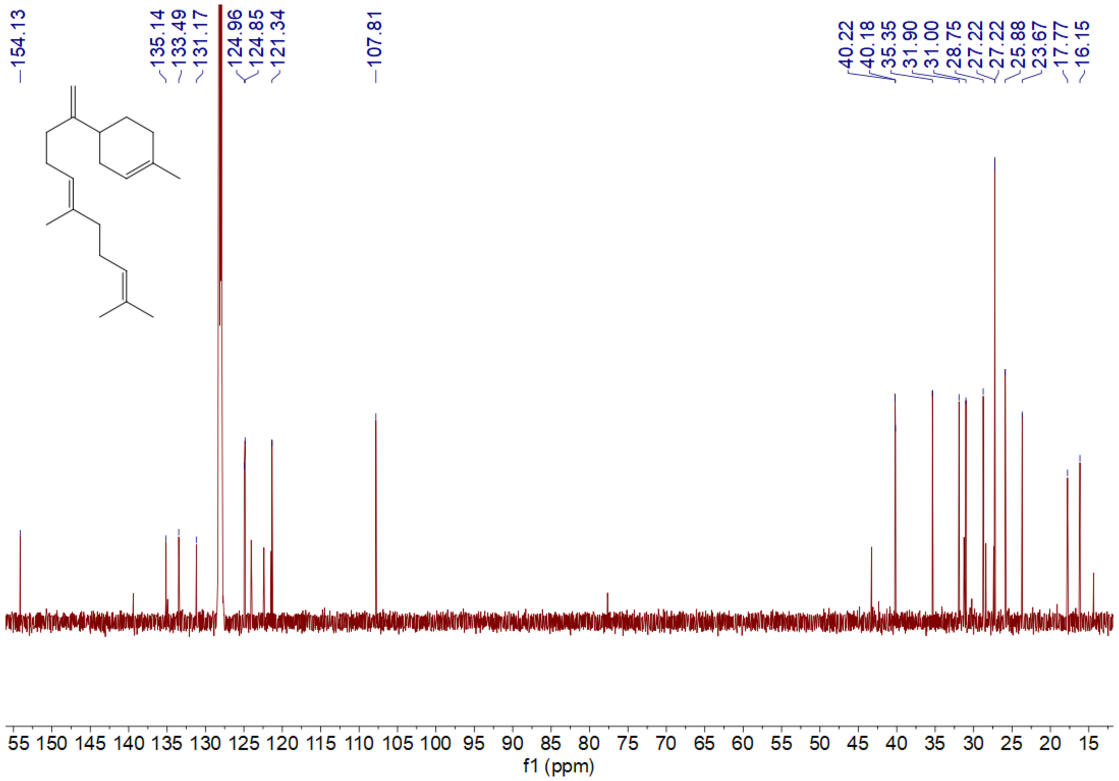

Supplementary Fig. 132. ^{13}C NMR spectrum (150 MHz) of **12** in C_6D_6.

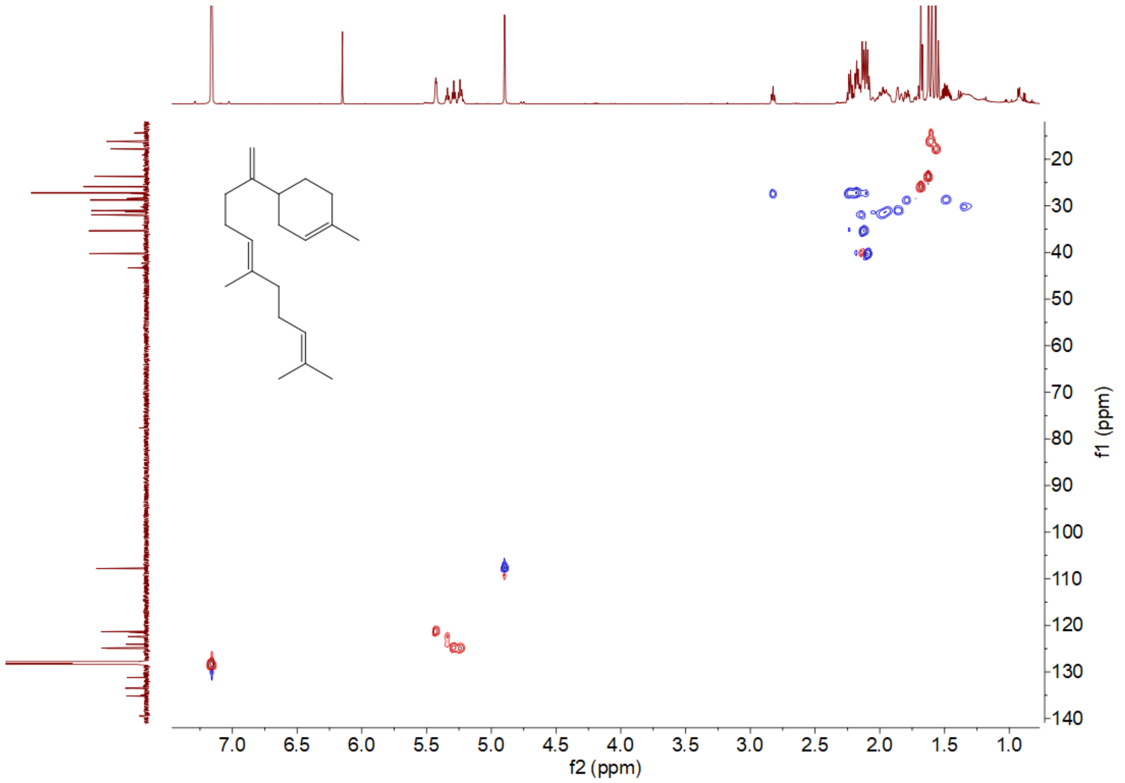

Supplementary Fig. 133. HSQC NMR spectrum of **12** in C_6D_6.

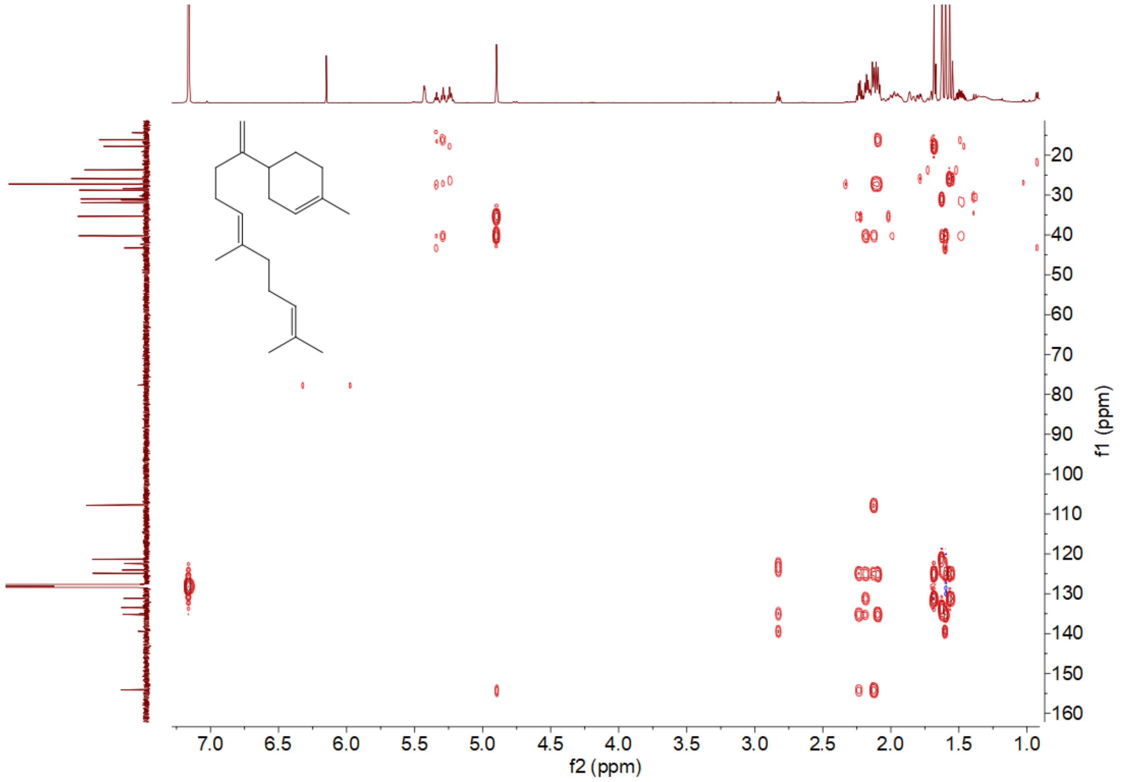

Supplementary Fig. 134. HMBC NMR spectrum of **12** in C_6D_6.

The variable temperature (VT) NMR of compound 1.

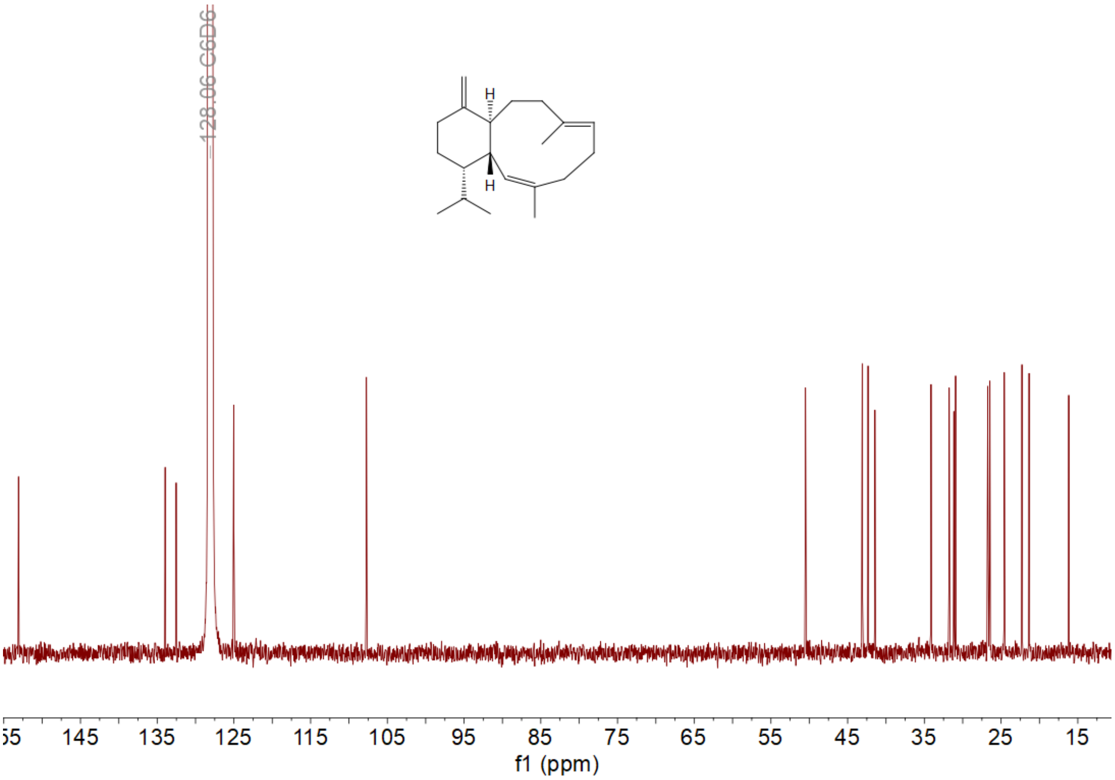

Supplementary Fig. 135. ^{13}C NMR spectrum of **1** in C_6D_6 at 298 K.

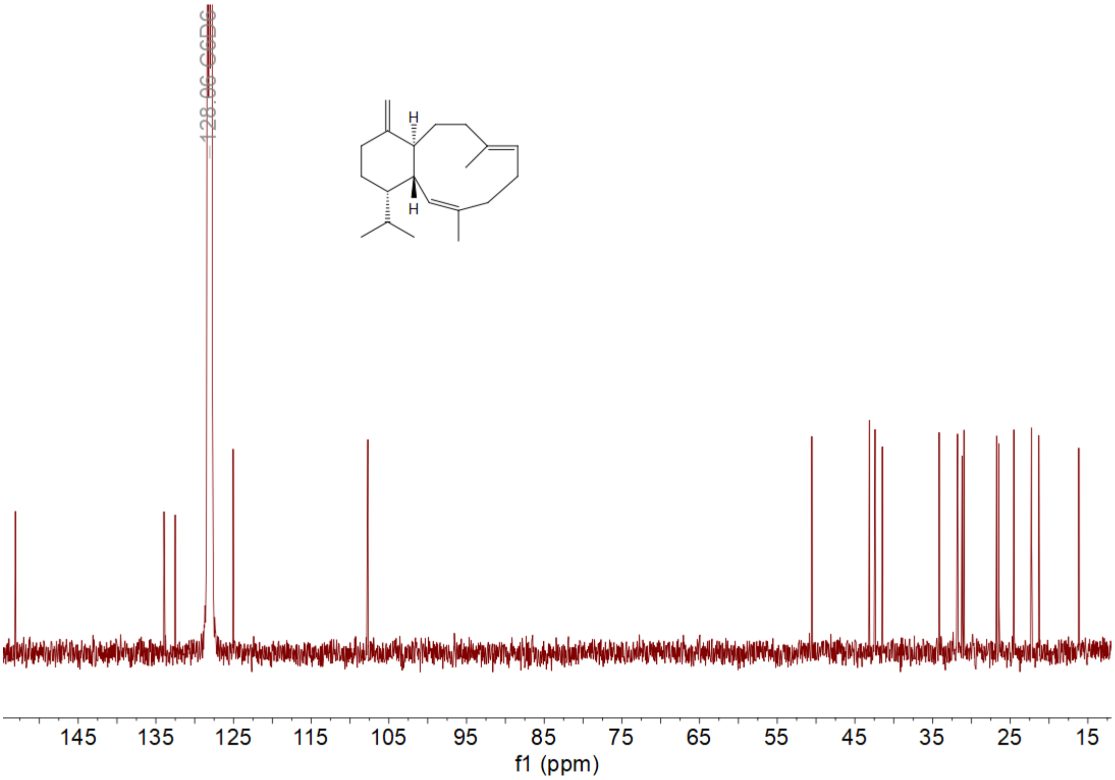

Supplementary Fig. 136. ^{13}C NMR spectrum of **1** in C_6D_6 at 308 K.

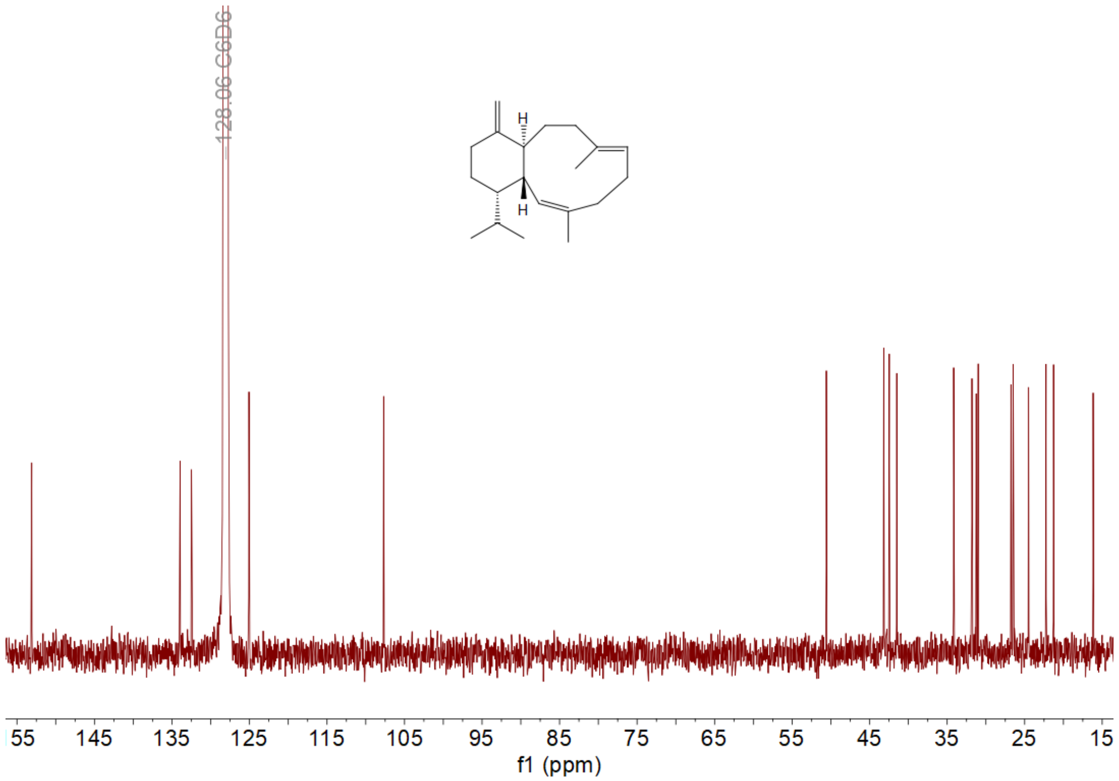

Supplementary Fig. 137. ^{13}C NMR spectrum of **1** in C_6D_6 at 318 K.

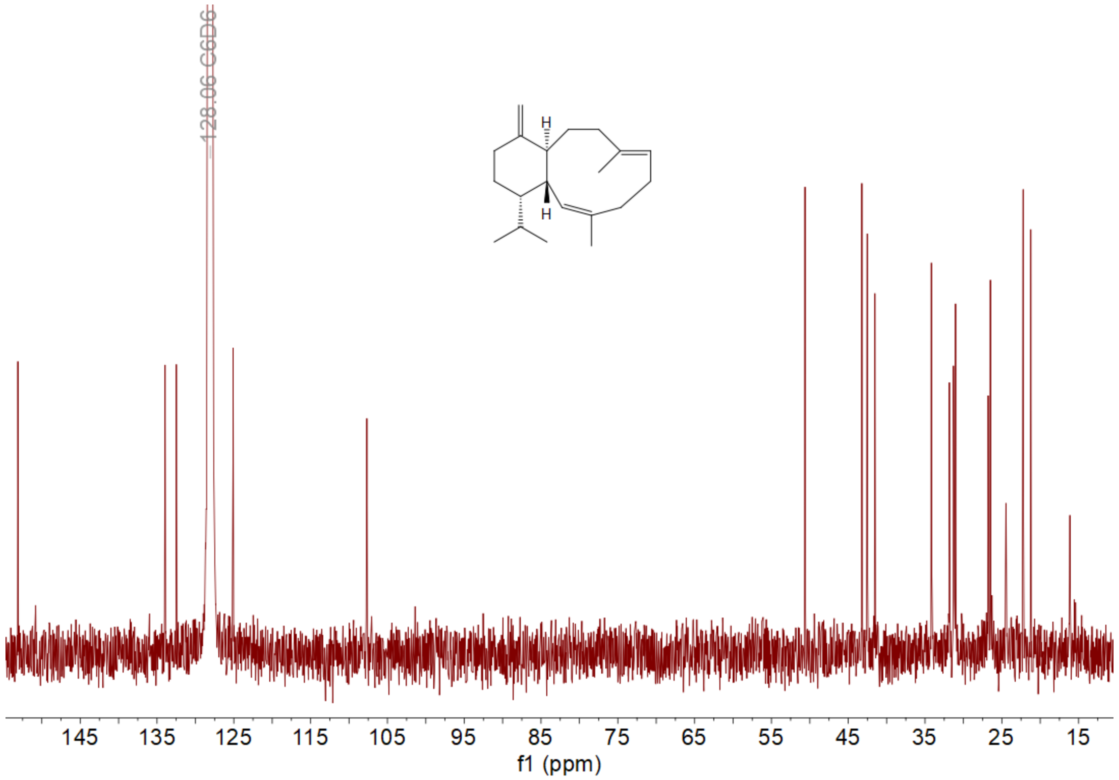

Supplementary Fig. 138. ^{13}C NMR spectrum of **1** in C_6D_6 at 328 K.

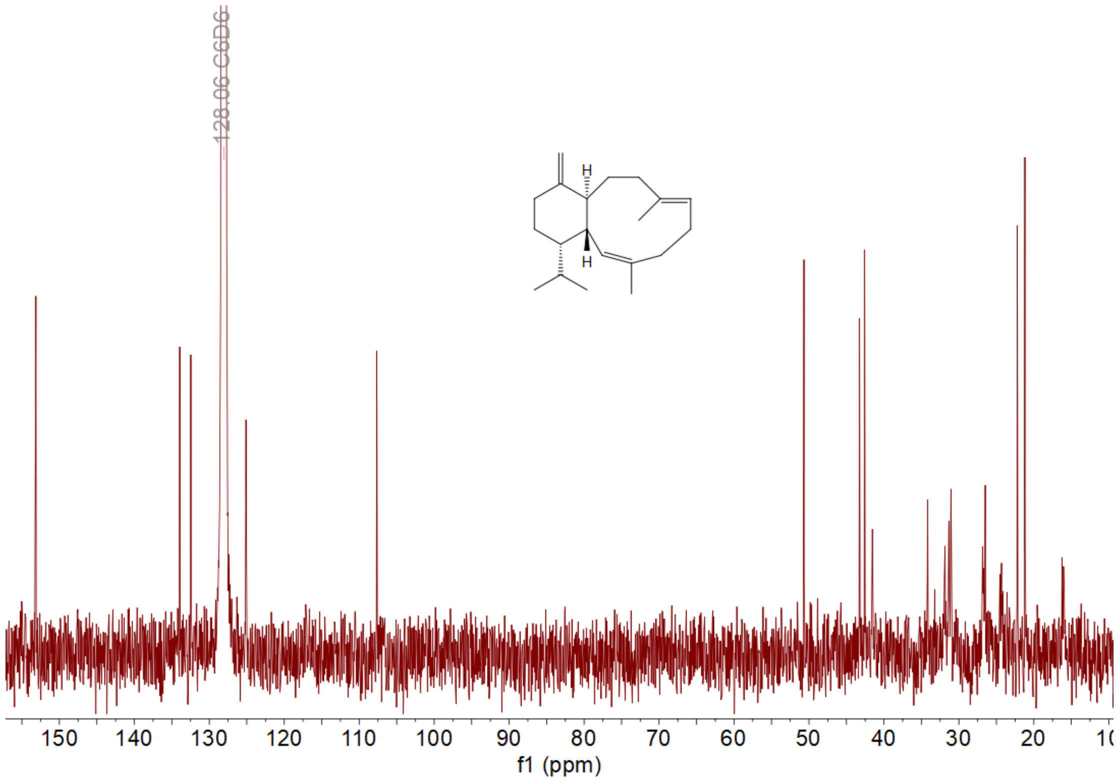

Supplementary Fig. 139. ^{13}C NMR spectrum of **1** in C_6D_6 at 338 K.

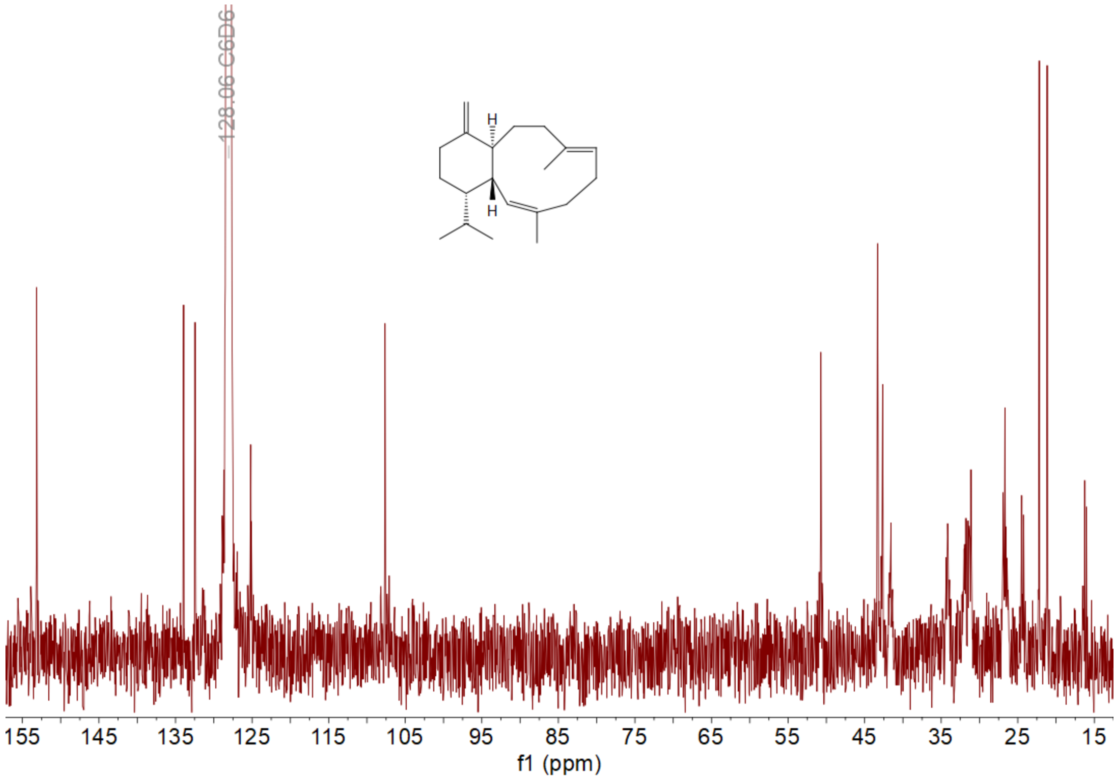

Supplementary Fig. 140. ^{13}C NMR spectrum of **1** in C_6D_6 at 348 K.

VT NMR of compound 6.

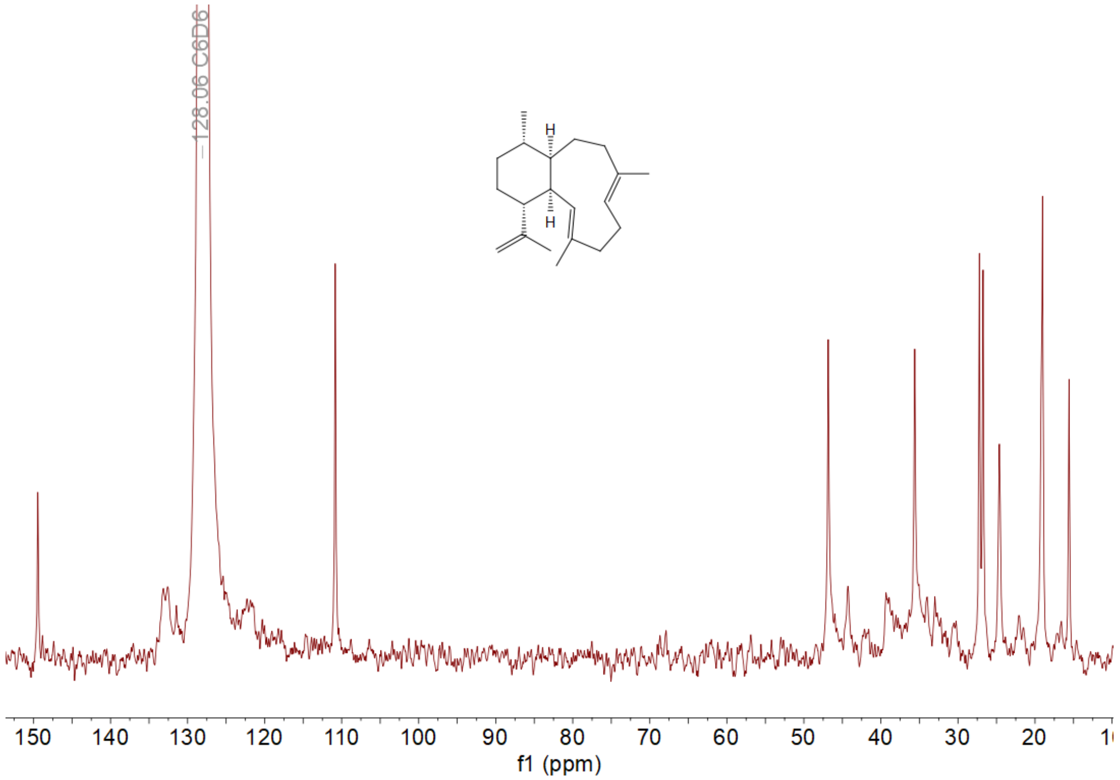

Supplementary Fig. 141. ^{13}C NMR spectrum of **6** in C_6D_6 at 298 K.

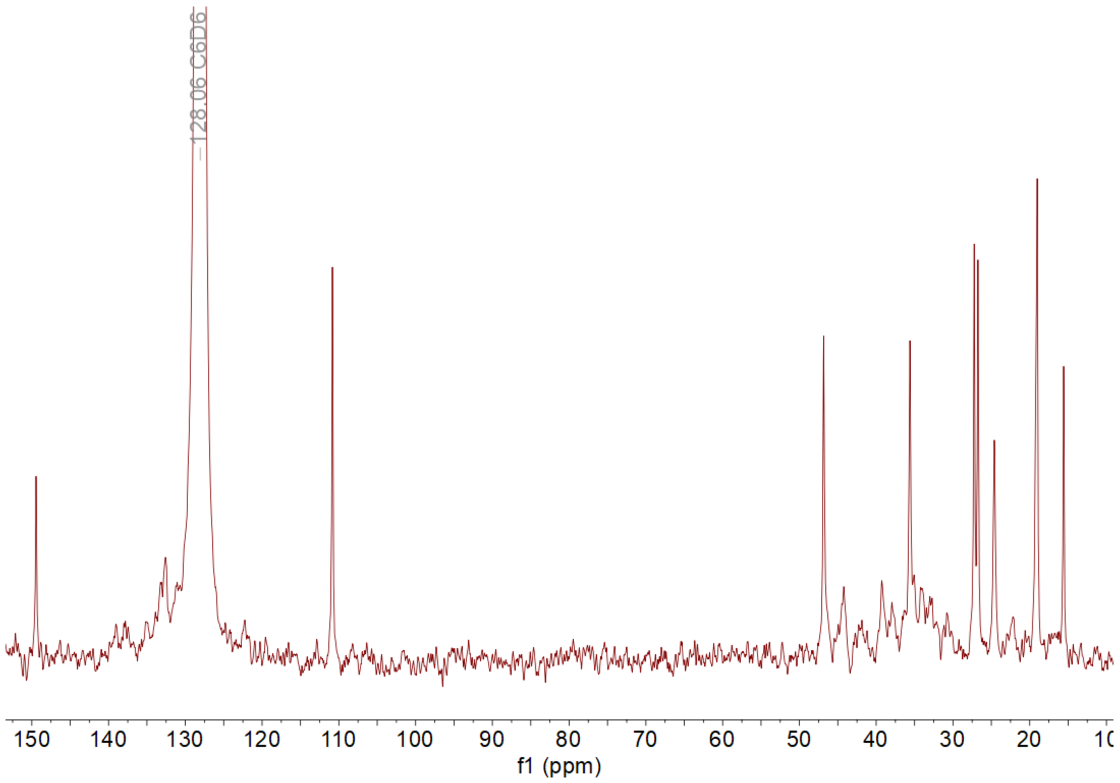

Supplementary Fig. 142. ^{13}C NMR spectrum of **6** in C_6D_6 at 308 K.

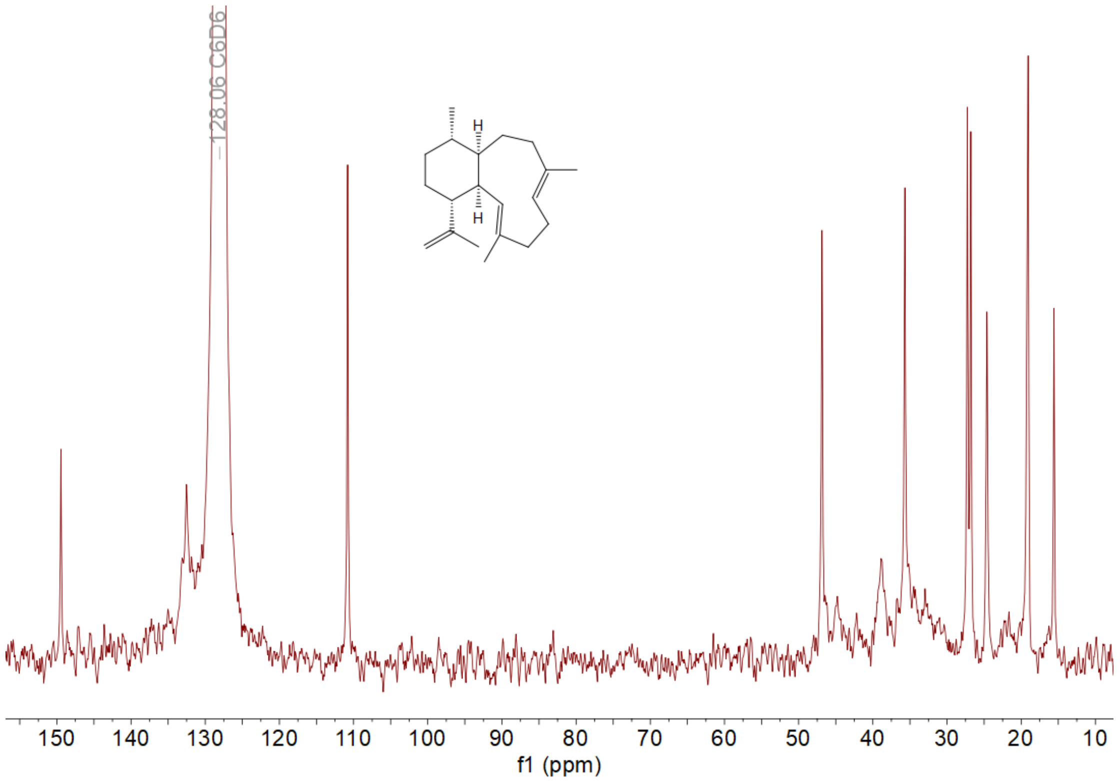

Supplementary Fig. 143. ^{13}C NMR spectrum of **6** in C_6D_6 at 318 K.

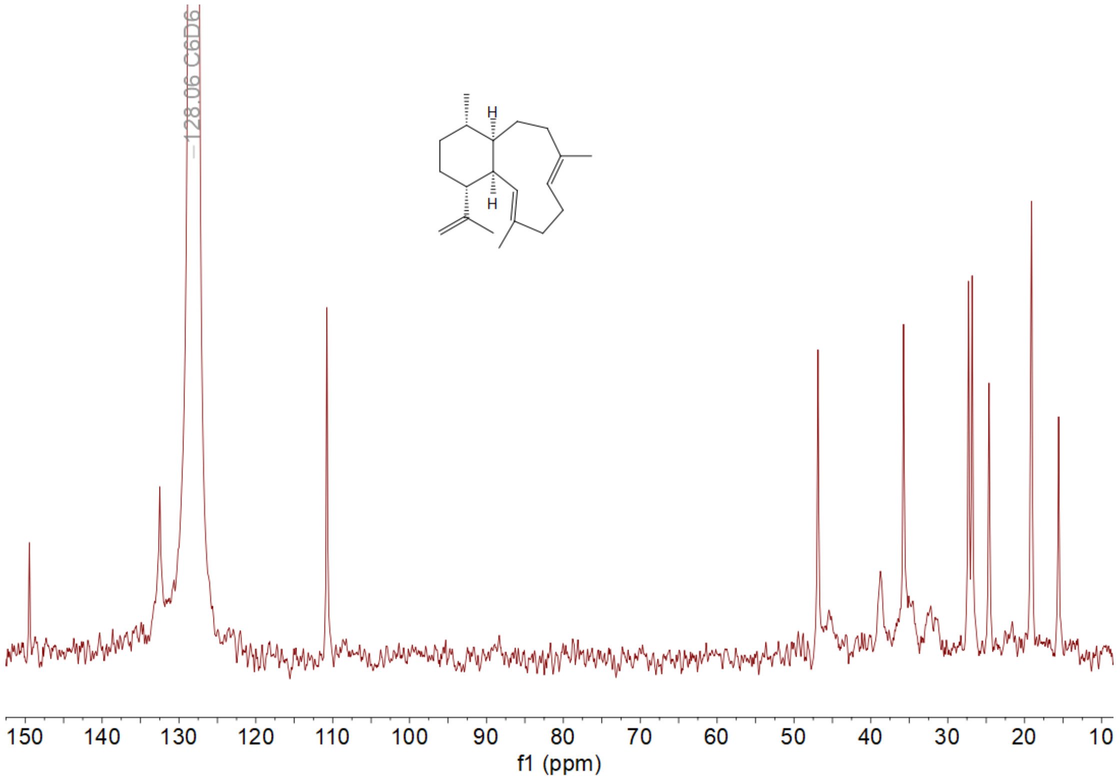

Supplementary Fig. 144. ^{13}C NMR spectrum of **6** in C_6D_6 at 328 K.

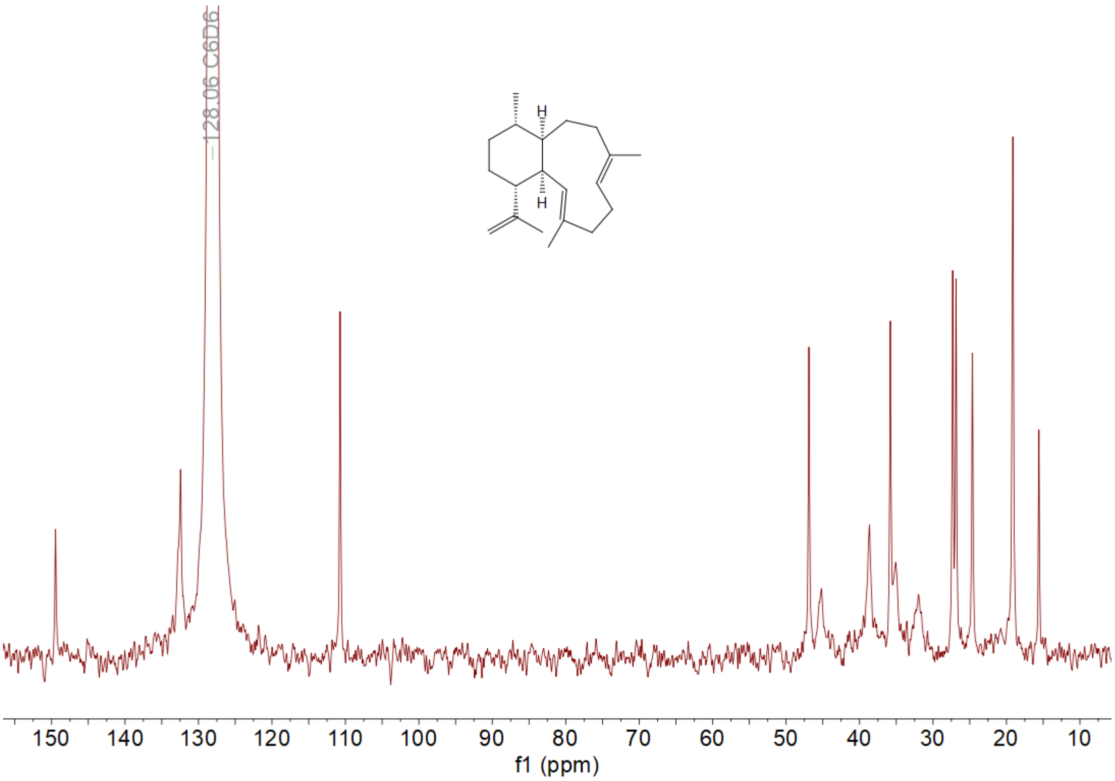

Supplementary Fig. 145. ^{13}C NMR spectrum of **6** in C_6D_6 at 338 K.

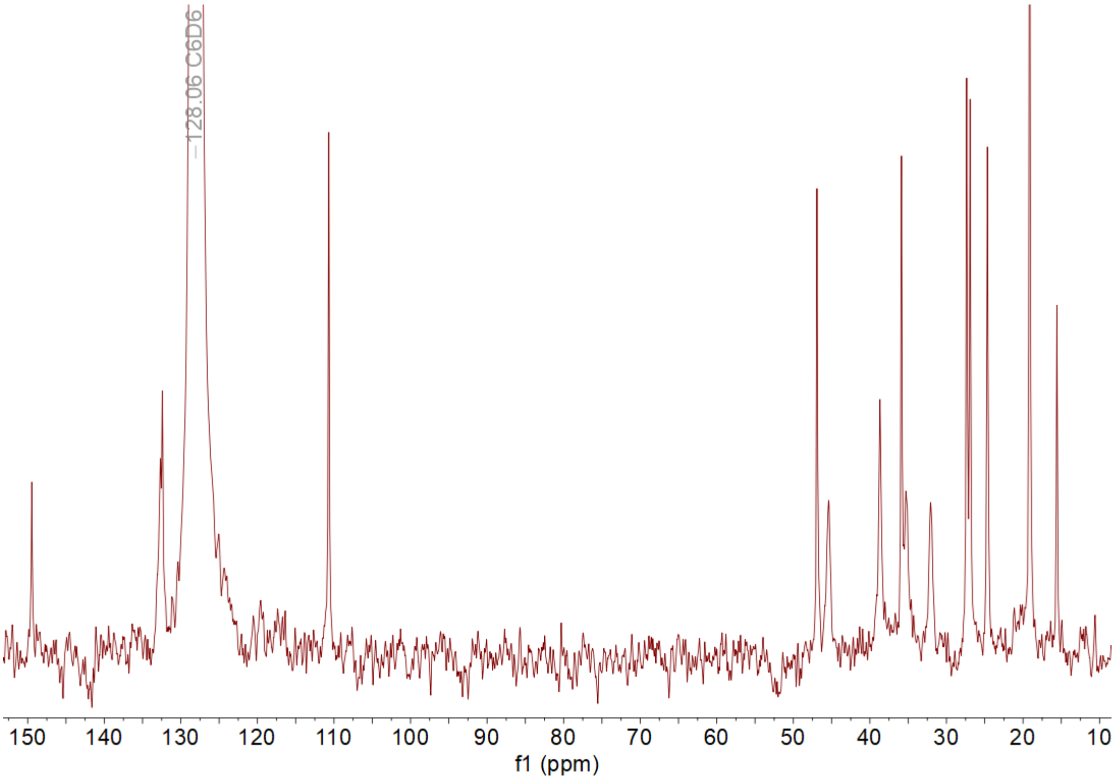

Supplementary Fig. 146. ^{13}C NMR spectrum of **6** in C_6D_6 at 348 K.

VT NMR of compound 7.

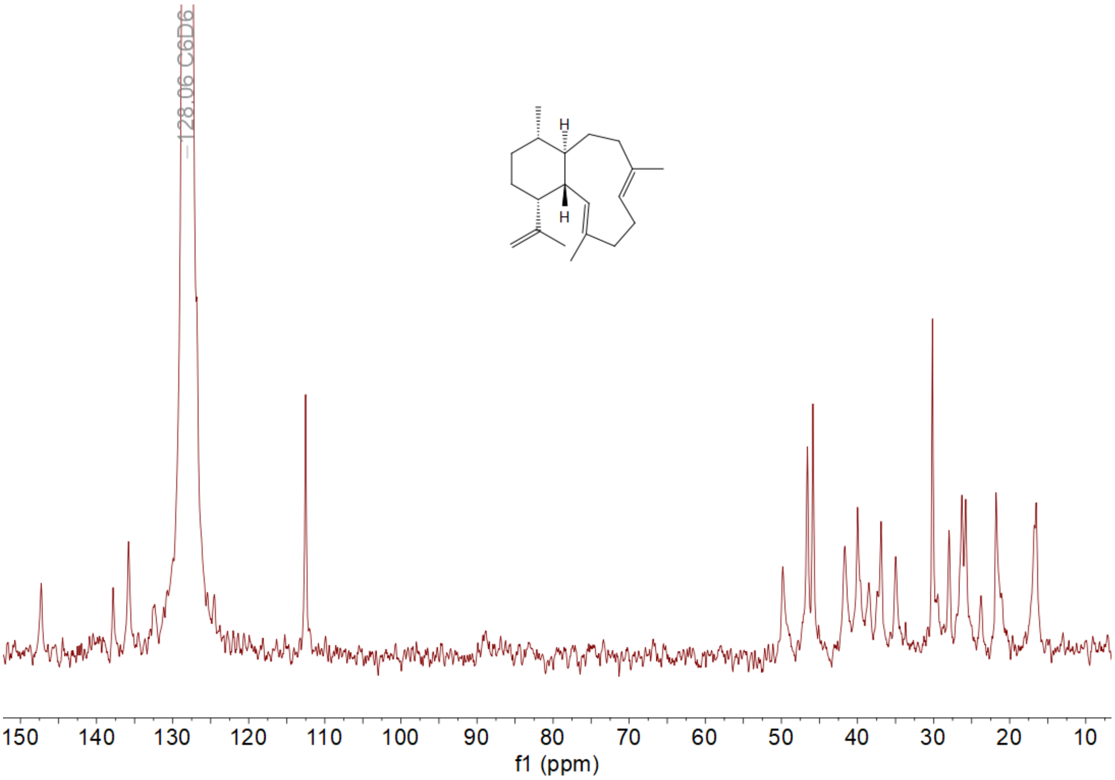

Supplementary Fig. 147. ^{13}C NMR spectrum of **7** in C_6D_6 at 298 K.

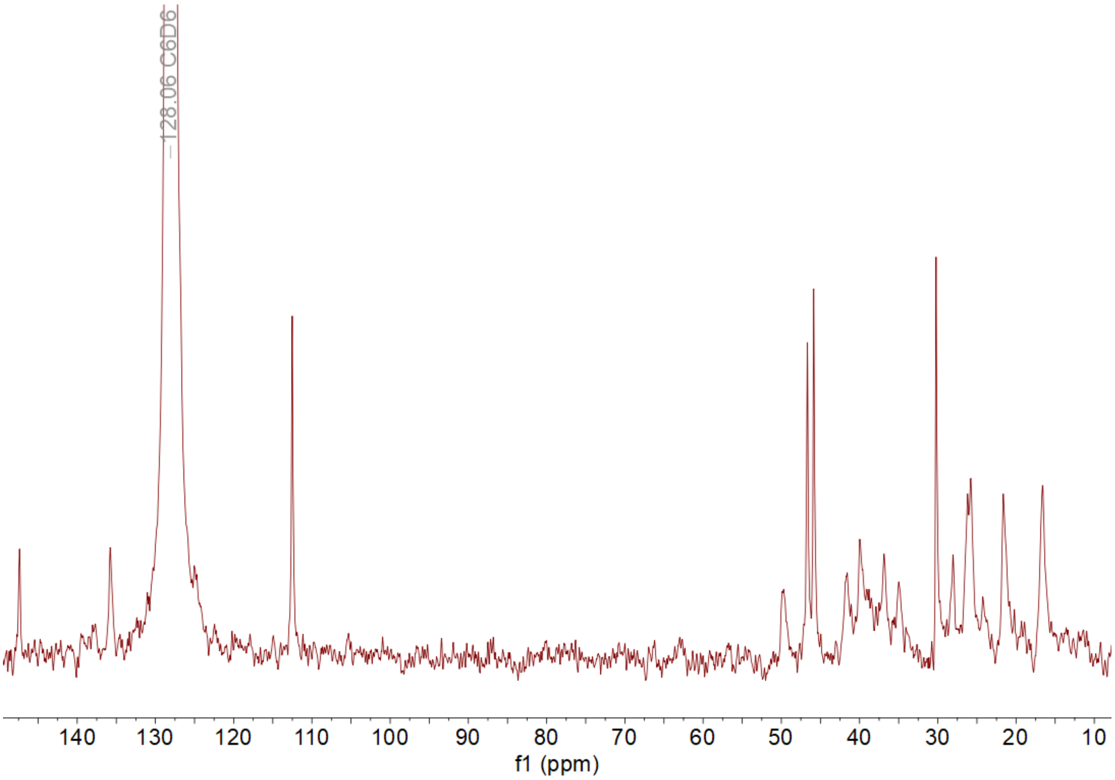

Supplementary Fig. 148. ^{13}C NMR spectrum of **7** in C_6D_6 at 308 K.

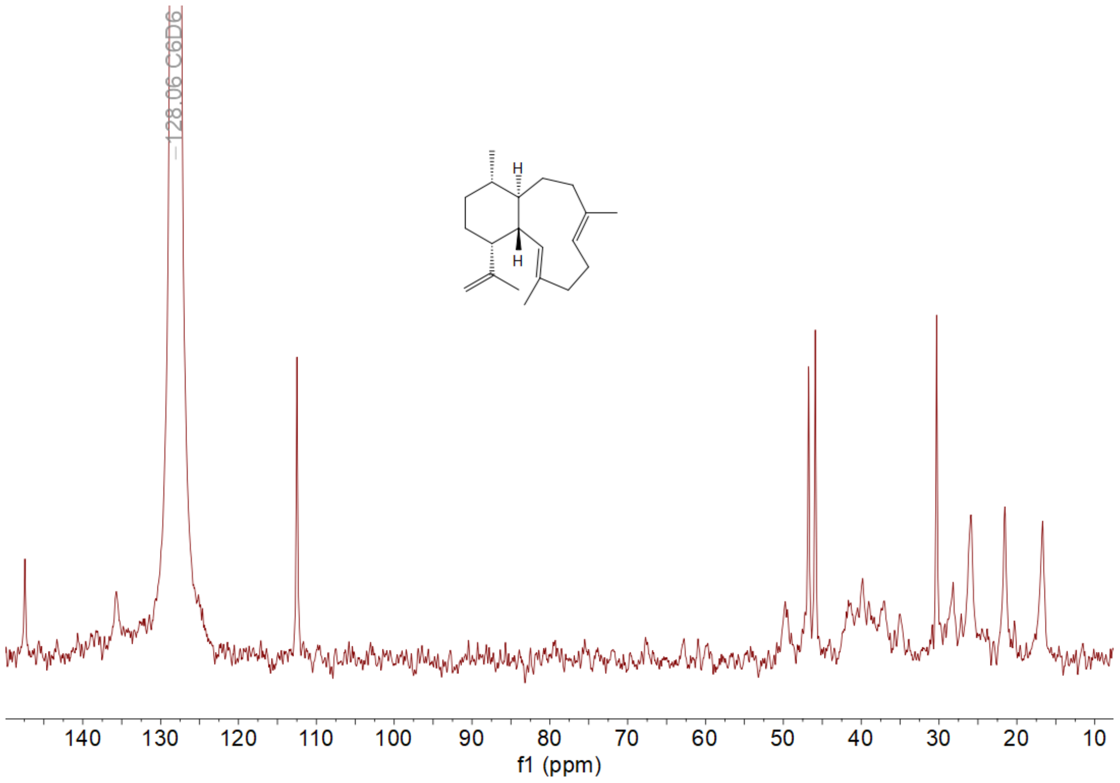

Supplementary Fig. 149. ^{13}C NMR spectrum of **7** in C_6D_6 at 318 K.

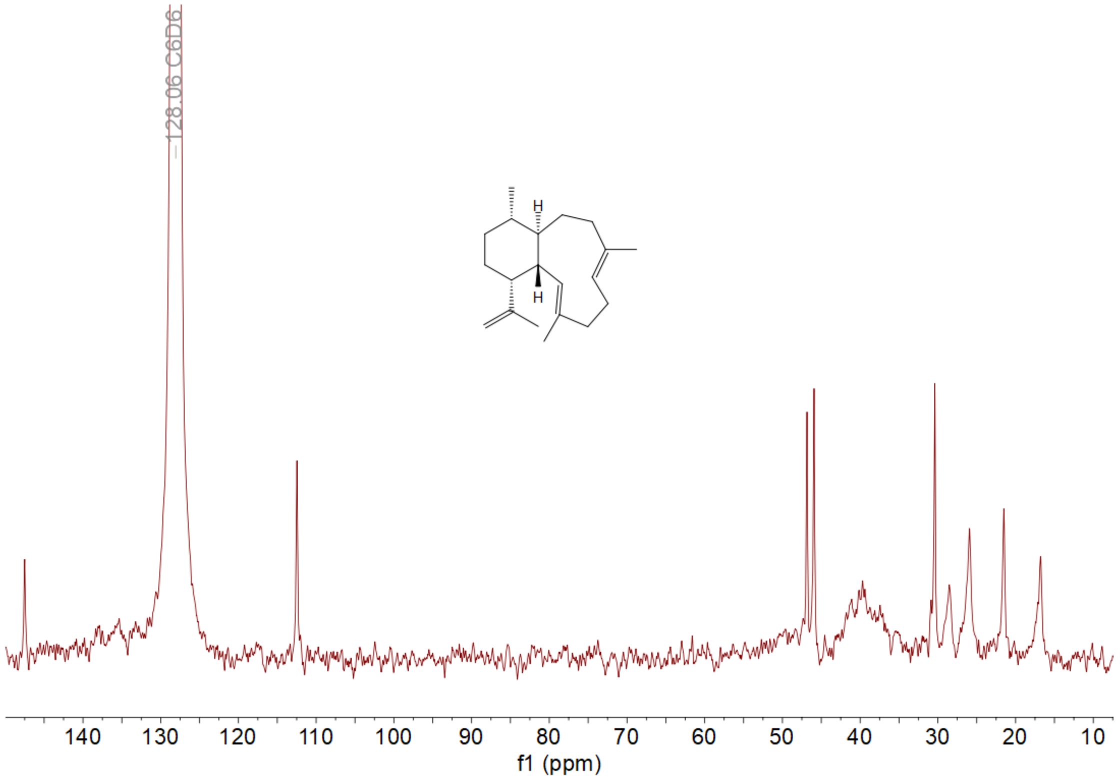

Supplementary Fig. 150. ^{13}C NMR spectrum of **7** in C_6D_6 at 328 K.

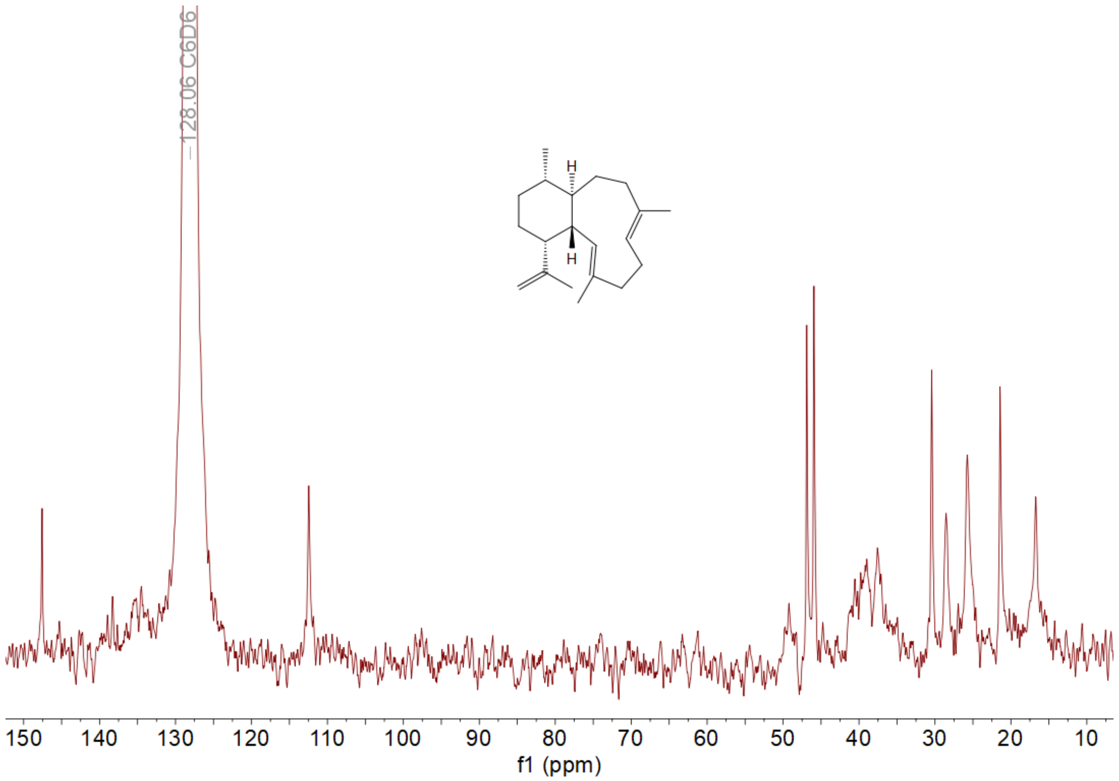

Supplementary Fig. 151. ^{13}C NMR spectrum of **7** in C_6D_6 at 338 K.

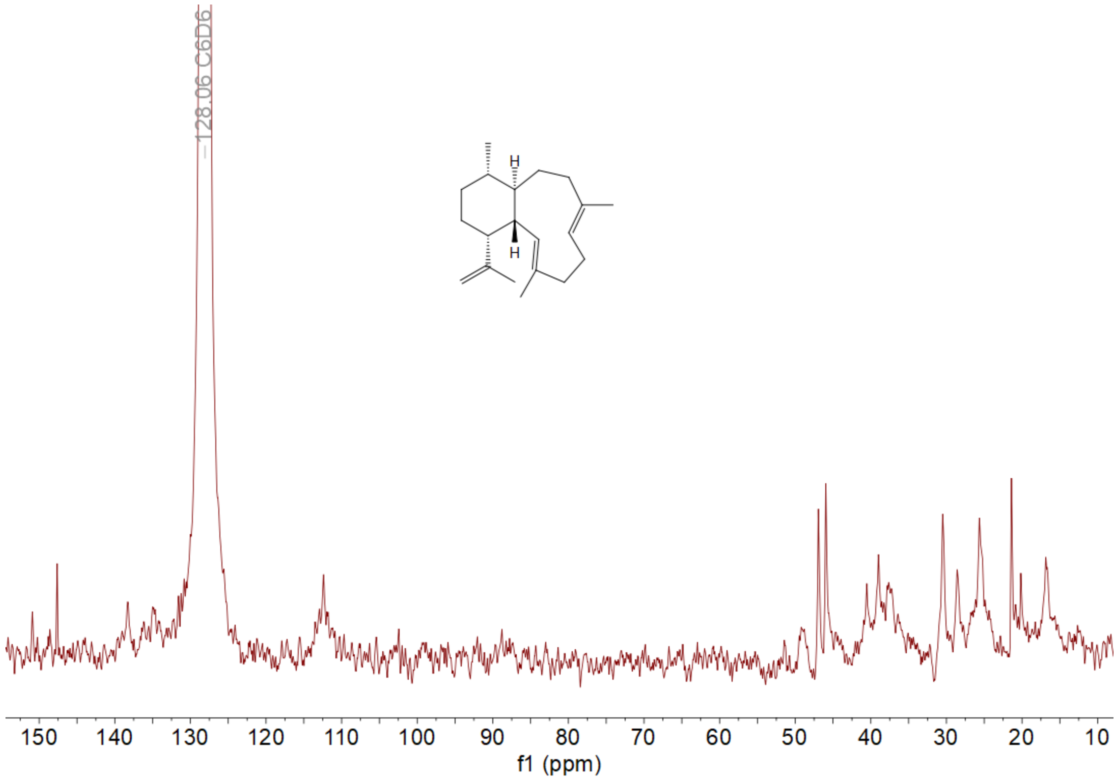

Supplementary Fig. 152. ^{13}C NMR spectrum of **7** in C_6D_6 at 348 K.

VT NMR of compound 8.

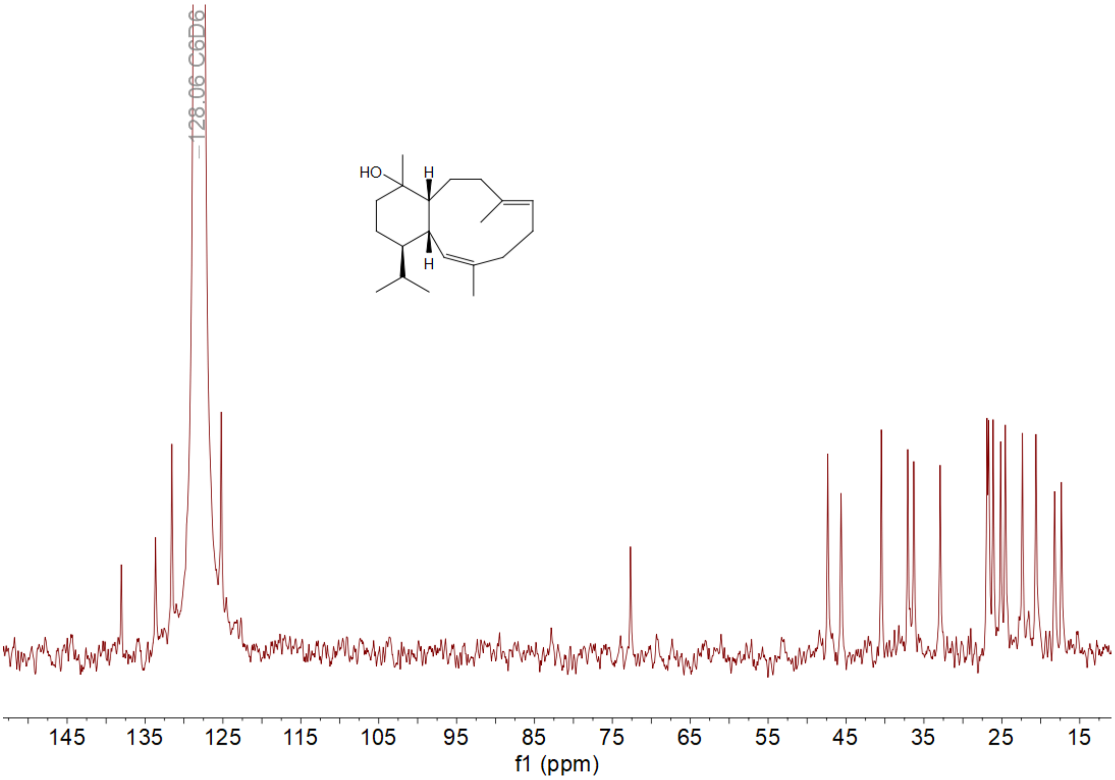

Supplementary Fig. 153. ^{13}C NMR spectrum of **8** in C_6D_6 at 298 K.

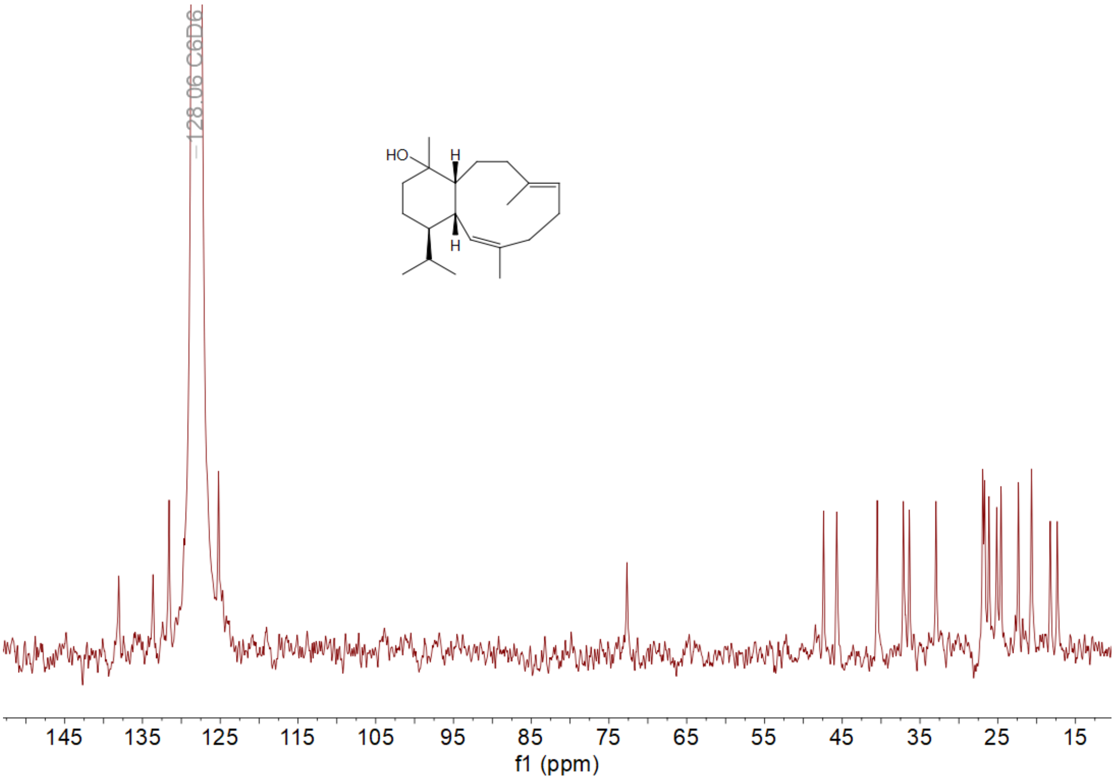

Supplementary Fig. 154. ^{13}C NMR spectrum of **8** in C_6D_6 at 308 K.

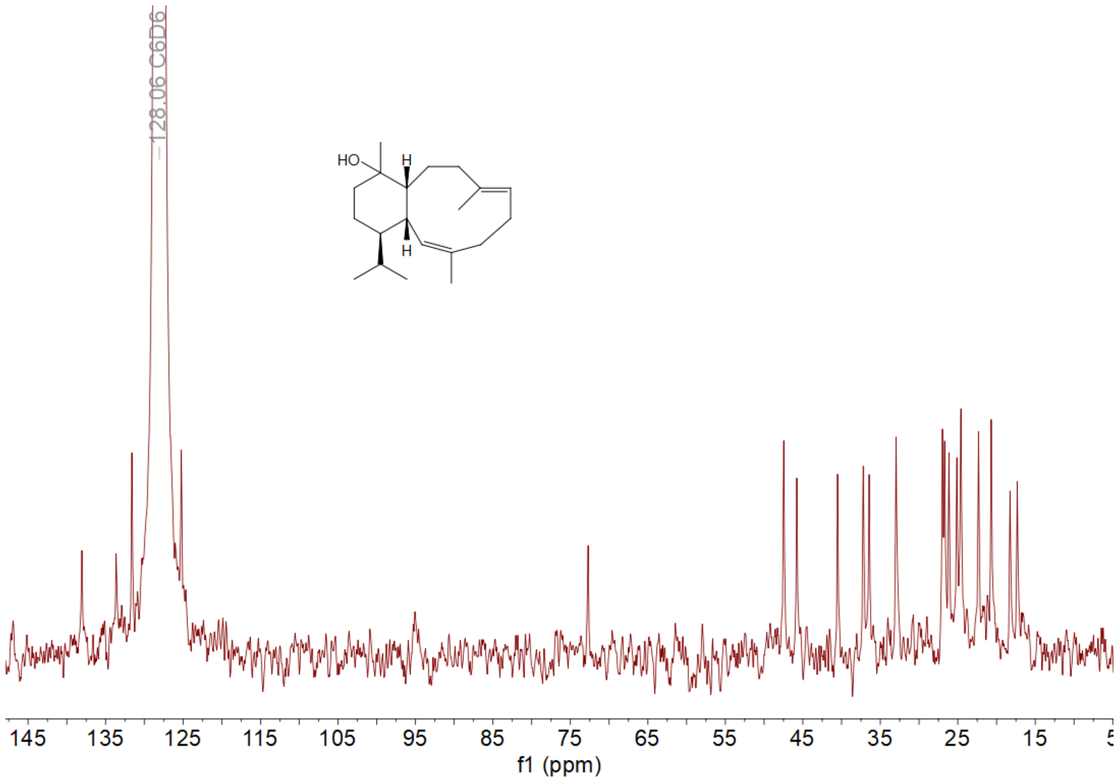

Supplementary Fig. 155. ^{13}C NMR spectrum of **8** in C_6D_6 at 318 K.

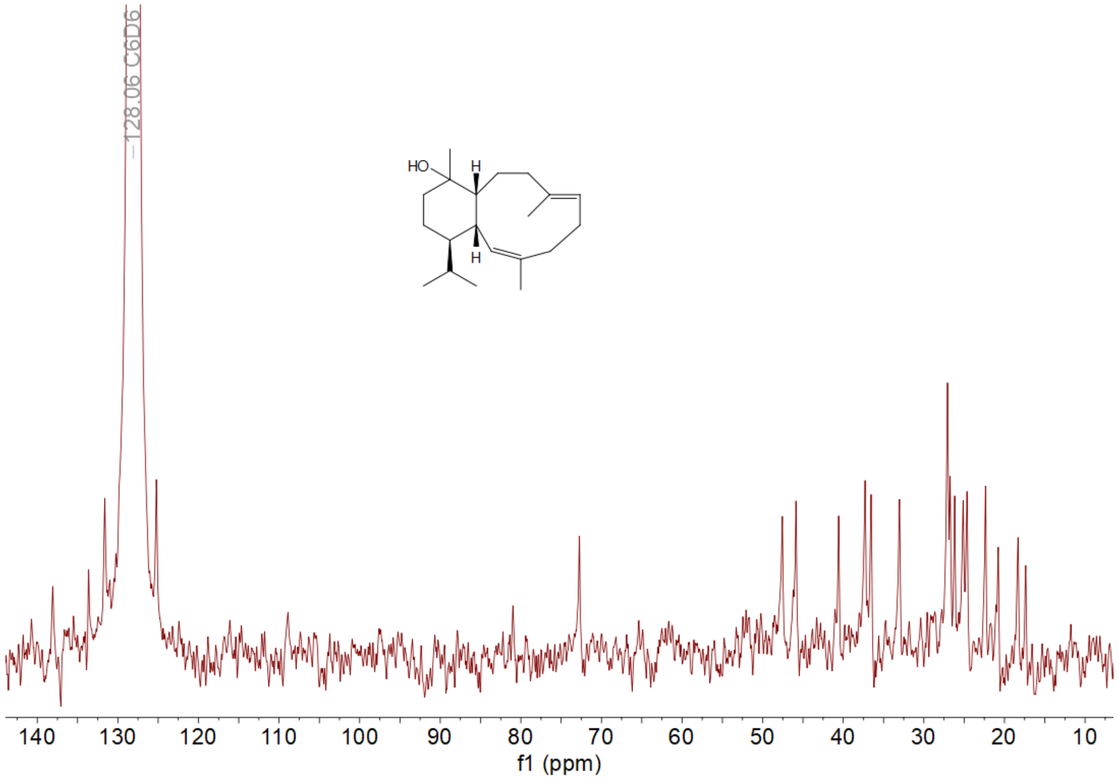

Supplementary Fig. 156. ^{13}C NMR spectrum of **8** in C_6D_6 at 328 K.

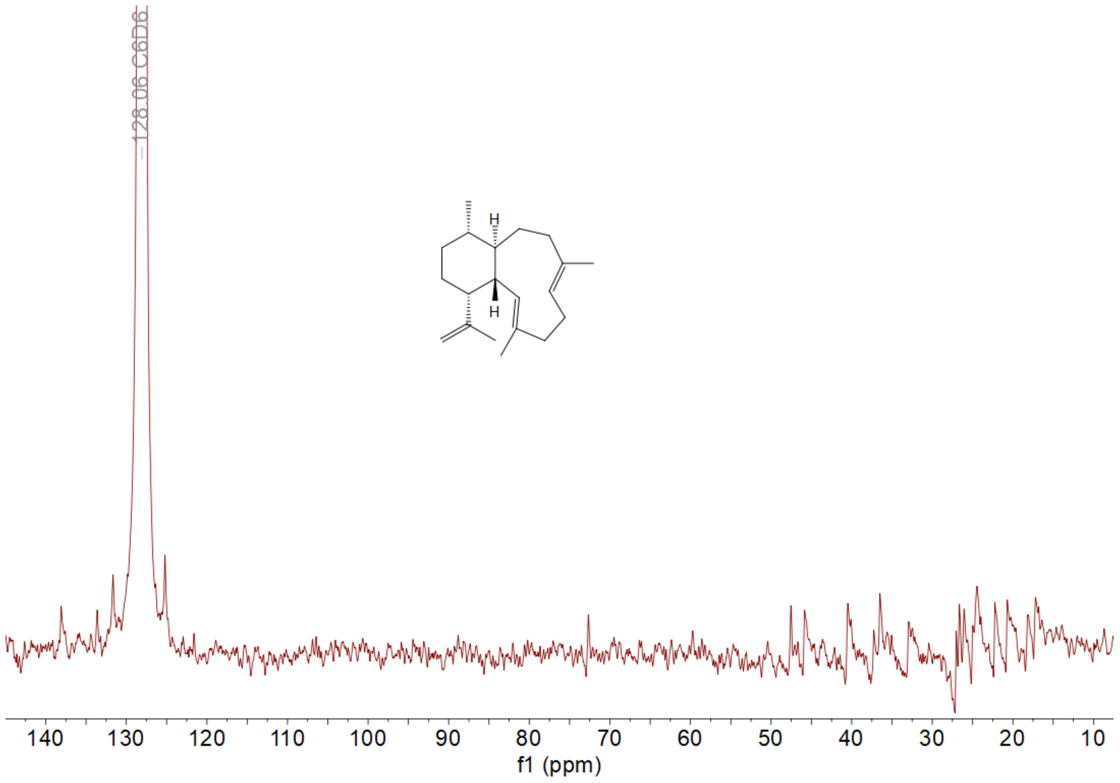

Supplementary Fig. 157. ^{13}C NMR spectrum of **8** in C_6D_6 at 338 K.

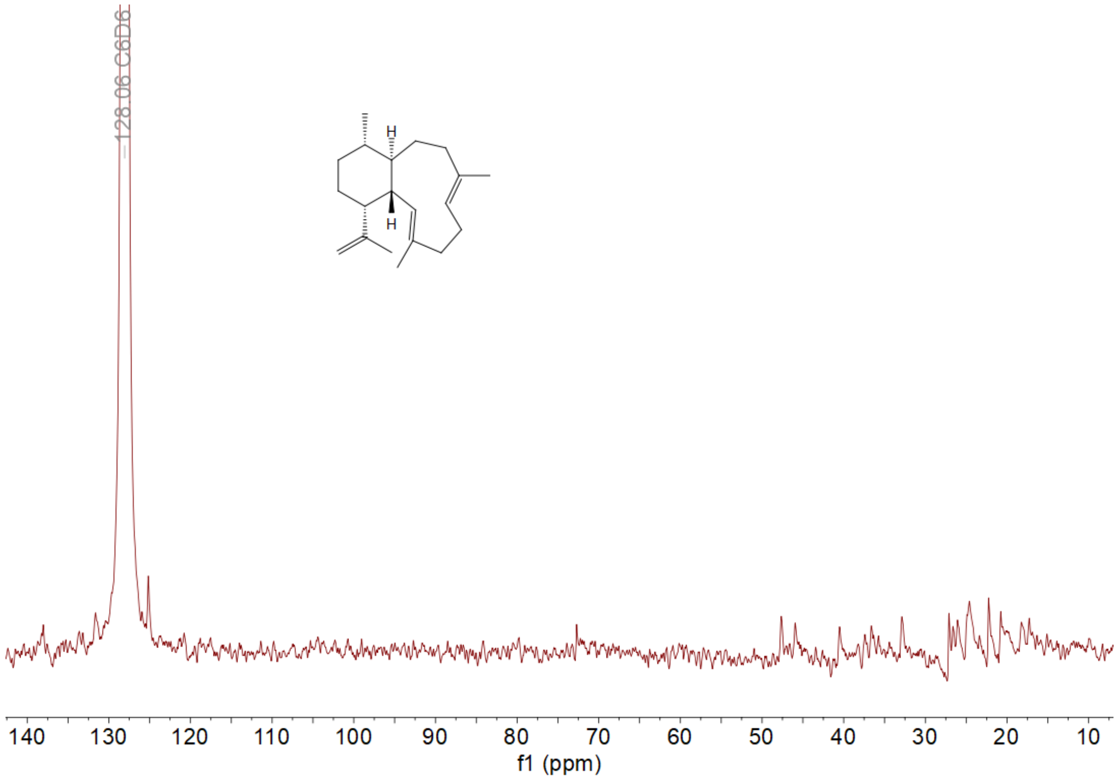

Supplementary Fig. 158. ^{13}C NMR spectrum of **8** in C_6D_6 at 348 K.

Supplementary Table

Supplementary Table 1. Strains used in this study.

Strain	Description	Source
E. coli Turbo	Host for general cloning	Shanghai Weidi Biotechnology
E. coli BL21 Gold (DE3)	Host for high-level protein production	Shanghai Weidi Biotechnology

Supplementary Table 2. Plasmids used in this study.

Plasmid	Description	Source [Reference]
CDF-MKI	Kinase-based system for DMAPP and IPP production (gift from *Prof.* Jeffrey D. Rudolf. Previously constructed by B.X.)	[13]
pET28a	General plasmid for cloning and protein production	Shanghai Weidi Biotechnology
CDF-MKI4	Kinase-based system for GGPP production (gift from *Prof.* Jeffrey D. Rudolf. Previously constructed by B.X.)	[13]
CDF-MKI-MicE	MicE replaced Bnd3 in MKI4 system	This study
pET28a-MicA-E159A	pET28a harboring MicA (E159A)	This study
pET28a-MicA-D81A	pET28a harboring MicA (D81A)	This study
pET28a-MicA-D82A	pET28a harboring MicA (D82A)	This study
pET28a-MicA-E85A	pET28a harboring MicA (E85A)	This study
pET28a-MicA-N224A	pET28a harboring MicA (N224A)	This study
pET28a-MicA-D225A	pET28a harboring MicA (D225A)	This study
pET28a-MicA-S228A	pET28a harboring MicA (S228A)	This study
pET28a-MicA-E232A	pET28a harboring MicA (E232A)	This study
pET28a-MicA-S187A	pET28a harboring MicA (S187A)	This study
pET28a-MicA-S187Y	pET28a harboring MicA (S187Y)	This study
pET28a-MicA-S187F	pET28a harboring MicA (S187F)	This study
pET28a-MicA-Y152A	pET28a harboring MicA (Y152A)	This study
pET28a-MicA-Y152C	pET28a harboring MicA (Y152C)	This study
pET28a-MicA-Y152S	pET28a harboring MicA (Y152S)	This study
pET28a-MicA-W313A	pET28a harboring MicA (W313A)	This study
pET28a-MicA-W306A	pET28a harboring MicA (W306A)	This study
pET28a-MicA-W306F	pET28a harboring MicA (W306F)	This study
pET28a-MicA-M74T	pET28a harboring MicA (M74T)	This study
pET28a-MicA-V220A	pET28a harboring MicA (V220A)	This study
pET28a-MicA-V220M	pET28a harboring MicA (V220M)	This study

pET28a-MicA-L221A	pET28a harboring MicA (L221A)	This study
pET28a-MicA-L221M	pET28a harboring MicA (L221M)	This study
pET28a-MicA-E53A	pET28a harboring MicA (E53A)	This study
pET28a-MicA-E53W	pET28a harboring MicA (E53W)	This study
pET28a-MicA-L54A	pET28a harboring MicA (L54A)	This study
pET28a-MicA-L54F	pET28a harboring MicA (L54F)	This study
pET28a-MicA-L54Y	pET28a harboring MicA (L54Y)	This study
pET28a-MicA-L54Y/E53W	pET28a harboring MicA (L54Y/E53W)	This study
pET28a-MicA-F51A	pET28a harboring MicA (F51A)	This study
pET28a-MicA-F51L	pET28a harboring MicA (F51L)	This study
pET28a-MicA-A78Y	pET28a harboring MicA (A78Y)	This study
pET28a-MicA-A78F	pET28a harboring MicA (A78F)	This study
pET28a-MicA-W77A	pET28a harboring MicA (W77A)	This study
pET28a-MicA-W77H	pET28a harboring MicA (W77H)	This study
pET28a-MicA-W77F	pET28a harboring MicA (W77F)	This study
pET28a-MicA-W77Y	pET28a harboring MicA (W77Y)	This study
pET28a-MicA-W77Q	pET28a harboring MicA (W77Q)	This study
pET28a-MicA-M184A	pET28a harboring MicA (M184A)	This study
pET28a-MicA-G183Y	pET28a harboring MicA (G183Y)	This study
pET28a-MicA-G182Y	pET28a harboring MicA (G182Y)	This study

Supplementary Table 3. Primer sequences used in this study.

Primer	Nucleotide Sequence (5´–3´)	Purpose
CDF-MKI-M134-PT-F	ATGACCGTGACCCAAGCCAC	MicE mutant amplification for expression in *E. coli*
CDF-MKI-M134-PT-R	TTAACGGGTACGCTGCAACA	
28a-MicA-F	ATGACCTTCACCGTCCCGGA	MicA mutant amplification for expression in *E. coli*
28a-MicA-R	TCACGGCTGCCCGGCGCGCT	
28a-MicA-E159A-F	TGTCATTGGGCATCCCATAACCGCCGGCGGGGGATCGCCC	MicA mutagenesis for E159A
28a-MicA-E159A-R	CCGGCGGTTATGGGATGCCCAATGACACCCTTCCAGGTAG	
28a-MicA-D81A-F	CTGGGCCATCTTCGCCGACTACCTGGAGCGCATTCCGGAC	MicA mutagenesis for D81A
28a-MicA-D81A-R	CTCCAGGTAGTCGGCGAAGATGGCCCAGACGGCCATCCAG	
28a-MicA-D82A-F	CTGGGCCATCTTCGACGCCTACCTGGAGCGCATTCCGGAC	MicA mutagenesis for D82A

28a-MicA-D82A-R	CGCTCCAGGTAGGCGTCGAAGATG GCCCAGACGGCCATCC	
28a-MicA-E85A-F	ATCTTCGACGACTACCTGGCGCGCA TTCCGGACGCCCAAG	
28a-MicA-E85A-R	GTCCGGAATGCGCGCCAGGTAGTC GTCGAAGATGGCCCAG	MicA mutagenesis for E85A
28a-MicA-N224A-F	CTGGTCCTGTGGGCCGCCGACGTGT TCTCCGTCGAGGCCG	
28a-MicA-N224A-R	GGAGAACACGTCGGCGGCCCACAG GACCAGGTCGGCGGTG	MicA mutagenesis for N224A
28a-MicA-D225A-F	GGTCCTGTGGGCCAACGCCGTGTTC TCCGTCGAGGCCGAG	
28a-MicA-D225A-R	GACGGAGAACACGGCGTTGGCCCA CAGGACCAGGTCGGCG	MicA mutagenesis for D225A
28a-MicA-S228A-F	GCCAACGACGTGTTCGCCGTCGAG GCCGAGAAGCGCGAGG	
28a-MicA-S228A-R	CTCGGCCTCGACGGCGAACACGTC GTTGGCCCACAGGACC	MicA mutagenesis for S228A
28a-MicA-E232A-F	GTTCTCCGTCGAGGCCGCTAAGCG CGAGGGCAACGTCAAT	
28a-MicA-E232A-R	GCCCTCGCGCTTAGCGGCCTCGACG GAGAACACGTCGTTG	MicA mutagenesis for E232A
28a-MicA-S187A-F	CTTTGGCGGCATGCGCCCGGCGAT GGACCTGTCCGAGATC	
28a-MicA-S187A-R	CAGGTCCATCGCCGGGCGCATGCC GCCAAAGCGGCGCCGG	MicA mutagenesis for S187A
28a-MicA-S187Y-F	CTTTGGCGGCATGCGCCCGTATATG GACCTGTCCGAGATC	
28a-MicA-S187Y-R	CAGGTCCATATACGGGCGCATGCC GCCAAAGCGGCGCCGG	MicA mutagenesis for S187Y
28a-MicA-S187F-F	CTTTGGCGGCATGCGCCCGTTCATG GACCTGTCCGAGATC	
28a-MicA-S187F-R	CAGGTCCATGAACGGGCGCATGCC GCCAAAGCGGCGCCGG	MicA mutagenesis for S187F
28a-MicA-Y152A-F	GTCCGCCACCTGACCGACGCCCTG GAAGGGTGTCATTGGG	
28a-MicA-Y152A-R	CCCTTCCAGGGCGTCGGTCAGGTG GCGGACAAACCGGCGG	MicA mutagenesis for Y152A
28a-MicA-Y152C-F	GTCCGCCACCTGACCGACTGCCTGG AAGGGTGTCATTGGG	
28a-MicA-Y152C-R	CCCTTCCAGGCAGTCGGTCAGGTG GCGGACAAACCGGCGG	MicA mutagenesis for Y152C
28a-MicA-Y152S-F	GTCCGCCACCTGACCGACTCCCTGG AAGGGTGTCATTGGG	MicA mutagenesis for Y152S

28a-MicA-Y152S-R	CCCTTCCAGGGAGTCGGTCAGGTG GCGGACAAACCGGCGG	
28a-MicA-W313A-F	CCGCGGCAACATCGACGCTTCCCG CGGCAACGAGCGCTAC	
28a-MicA-W313A-R	GCCGCGGGAAGCGTCGATGTTGCC GCGGACCCAGGACTCC	MicA mutagenesis for W313A
28a-MicA-W306A-F	CATCGCCGCGATGGAGTCCGCGGT CCGCGGCAACATCGAC	
28a-MicA-W306A-R	GCCGCGGACCGCGGACTCCATCGC GGCGATGTAGCGGGAG	MicA mutagenesis for W306A
28a-MicA-W306F-F	CATCGCCGCGATGGAGTCCTTTGTC CGCGGCAACATCGAC	
28a-MicA-W306F-R	GCCGCGGACAAAGGACTCCATCGC GGCGATGTAGCGGGAG	MicA mutagenesis for W306F
28a-MicA-M74T-F	GTCATCACCGACTGGACGGCCGTCT GGGCCATCTTCGACG	
28a-MicA-M74T-R	GGCCCAGACGGCCGTCCAGTCGGT GATGACGCACAGCTCC	MicA mutagenesis for M74T
28a-MicA-V220A-F	GACAACACCGCCGACCTGGCCCTG TGGGCCAACGACGTGT	
28a-MicA-V220A-R	GTTGGCCCACAGGGCCAGGTCGGC GGTGTTGTCCAGCAGC	MicA mutagenesis for V220A
28a-MicA-V220M-F	GACAACACCGCCGACCTGATGCTG TGGGCCAACGACGTGT	
28a-MicA-V220M-R	GTTGGCCCACAGCATCAGGTCGGC GGTGTTGTCCAGCAGC	MicA mutagenesis for V220M
28a-MicA-L221A-F	CAACACCGCCGACCTGGTCGCGTG GGCCAACGACGTGTTC	
28a-MicA-L221A-R	GTCGTTGGCCCACGCGACCAGGTC GGCGGTGTTGTCCAGC	MicA mutagenesis for L221A
28a-MicA-L221M-F	CAACACCGCCGACCTGGTCATGTG GGCCAACGACGTGTTC	
28a-MicA-L221M-R	GTCGTTGGCCCACATGACCAGGTC GGCGGTGTTGTCCAGC	MicA mutagenesis for L221M
28a-MicA-E53A-F	CCGGACGCGGTTCGGCGCACTCGT GGCCCGGGCCTATCCG	
28a-MicA-E53A-R	CGGGCCACGAGTGCGCCGAACCGC GTCCGGTCGTAGTGGA	MicA mutagenesis for E53A
28a-MicA-E53W-F	CCGGACGCGGTTCGGCTGGCTCGT GGCCCGGGCCTATCCG	
28a-MicA-E53W-R	CGGGCCACGAGCCAGCCGAACCGC GTCCGGTCGTAGTGGA	MicA mutagenesis for E53W
28a-MicA-L54A-F	CGGACGCGGTTCGGCGAAGCCGTG GCCCGGGCCTATCCGT	MicA mutagenesis for L54A

28a-MicA-L54A-R	GCCCGGGCCACGGCTTCGCCGAAC CGCGTCCGGTCGTAGT	
28a-MicA-L54F-F	CGGACGCGGTTCGGCGAATTCGTG GCCCGGGCCTATCCGT	
28a-MicA-L54F-R	GCCCGGGCCACGAATTCGCCGAAC CGCGTCCGGTCGTAGT	MicA mutagenesis for L54F
28a-MicA-L54Y-F	CGGACGCGGTTCGGCGAATACGTG GCCCGGGCCTATCCGT	
28a-MicA-L54Y-R	GCCCGGGCCACGTATTCGCCGAAC CGCGTCCGGTCGTAGT	MicA mutagenesis for L54Y
28a-MicA-F51A-F	TACGACCGGACGCGGGCCGGCGAA CTCGTGGCCCGGGCCT	
28a-MicA-F51A-R	CACGAGTTCGCCGGCCCGCGTCCG GTCGTAGTGGAACTTG	MicA mutagenesis for F51A
28a-MicA-F51L-F	TACGACCGGACGCGGCTCGGCGAA CTCGTGGCCCGGGCCT	
28a-MicA-F51L-R	CACGAGTTCGCCGAGCCGCGTCCG GTCGTAGTGGAACTTG	MicA mutagenesis for F51L
28a-MicA-A78Y-F	TGGATGGCCGTCTGGTACATCTTCG ACGACTACCTGGAGC	
28a-MicA-A78Y-R	GTCGTCGAAGATGTACCAGACGGC CATCCAGTCGGTGATG	MicA mutagenesis for A78Y
28a-MicA-A78F-F	TGGATGGCCGTCTGGTTCATCTTCG ACGACTACCTGGAGC	
28a-MicA-A78F-R	GTCGTCGAAGATGAACCAGACGGC CATCCAGTCGGTGATG	MicA mutagenesis for A78F
28a-MicA-M184A-F	CGCCGCTTTGGCGGCGCGCGCCCG TCGATGGACCTGTCCG	
28a-MicA-M184A-R	CCATCGACGGGCGCGCGCCGCCAA AGCGGCGCCGGGTCCG	MicA mutagenesis forM184A
28a-MicA-G183Y-F	CGGCGCCGCTTTGGCTACATGCGCC CGTCGATGGACCTGT	
28a-MicA-G183Y-R	CGACGGGCGCATGTAGCCAAAGCG GCGCCGGGTCCGAATG	MicA mutagenesis for G183Y
28a-MicA-G182Y-F	CGGACCCGGCGCCGCTTTTACGGC ATGCGCCCGTCGATGG	
28a-MicA-G182Y-R	CGGGCGCATGCCGTAAAAGCGGCG CCGGGTCCGAATGTAC	MicA mutagenesis for G182Y

Supplementary Table 4. DNA and protein sequence of MicA.

DNA sequence:

ATGACCTTCACCGTCCCGGACCTGTCCGTCCCGTTCCCGGAGCGGCAGAACCCGCATGT
GGACGAGGCCGAGGTCCACGTCCGCGAGTACCTGCGCGAGTTCGGCCTGCTGCGCTCCG
AGGAGGCCAAGTTCCACTACGACCGGACGCGGTTCGGCGAACTCGTGGCCCGGGCCTA
TCCGTTCGCCGCCCTGGAGGAGCTGTGCGTCATCACCGACTGGATGGCCGTCTGGGCCA
TCTTCGACGACTACCTGGAGCGCATTCCGGACGCCCAAGACGACGAGCGCTTCGTCGCC
CTGATTCACGAAACCGTCTCGTGGTTCCCCCTCACGCCGCCGGGCGCCGTGGCCCGCTC
CGGCAACCCGATCGAGCTGGCGATTCGCGACATCTGGGATCGCCTGACCGCCCGCTCCT
CCCTCACCTGGCGCCGCCGGTTTGTCCGCCACCTGACCGACTACCTGGAAGGGTGTCAT
TGGGAATCCCATAACCGCCGGCGGGGGATCGCCCCGGATCTGCCGACGTACATTCGGA
CCCGGCGCCGCTTTGGCGGCATGCGCCCGTCGATGGACCTGTCCGAGATCGGCCTGGGC
ATCGAGCTGACCGACGACGTCCACGCCCACCCGCGCATTCAGCAGCTGCTGGACAACA
CCGCCGACCTGGTCCTGTGGGCCAACGACGTGTTCTCCGTCGAGGCCGAGAAGCGCGA
GGGCAACGTCAATAACATTGTCCTGGTGGTGCAGCGGACCCGGGGCGGGTCCATGCGG
GAAGCCGCGGATGAAGTGGCCGCCATGCTCCGCGGCCGCTGCGCCGACTTCGTCGCCGC
GTCCCGGTCGGCGGTCGCGTTTTTCGCCTCCTCCGGGGGGTACACCGCCGAGCAAGCCC
ACCAAGTCTCCCGCTACATCGCCGCGATGGAGTCCTGGGTCCGCGGCAACATCGACTGG
TCCCGCGGCAACGAGCGCTACCGCTCCGAGCACCTGCGCACCGGCGAGGATCAGCCGA
ACTTCCTGGAGCGCGCCGGGCAGCCGTGA

Protein sequence:

MTFTVPDLSVPFPERQNPHVDEAEVHVREYLREFGLLRSEEAKFHYDRTRFGELVA
RAYPFAALEELCVITDWMAVWAIFDDYLERIPDAQDDERFVALIHETVSWFPLTPP
GAVARSGNPIELAIRDIWDRLTARSSLTWRRRFVRHLTDYLEGCHWESHNRRRGIA
PDLPTYIRTRRRFGGMRPSMDLSEIGLGIELTDDVHAHPRIQQLLDNTADLVLWAN
DVFSVEAEKREGNVNNIVLVVQRTRGGSMREAADEVAAMLRGRCADFVAASRSA
VAFFASSGGYTAEQAHQVSRYIAAMESWVRGNIDWSRGNERYRSEHLRTGEDQP
NFLERAGQP

Supplementary Table 5. ^{1}H (600 MHz) and ^{13}C NMR (150 MHz) data of 1 and ($^{2}H_2$)-1 in $CDCl_3$.

NO.	**1**		($^{2}H_2$)-**1**	
	δ_H, mult. (*J*, Hz)	δ_C (mult.)	δ_H, mult. (*J*, Hz)	δ_C (mult.)
1	2.12 (dd, 11.8, 4.7)	42.1, CH	-	41.6, C
2	5.23 (d, 11.4)	127.6, CH	5.22 (s)	127.6, CH
3	-	132.2, C	-	132.2, C
4a	2.60 (td, 13.1, 4.4)	31.5, CH_2	2.58 (td, 13.1, 4.4)	31.5, CH_2
4b	1.68 (overlapped)		1.68 (overlapped)	
5a	2.35 (ddt, 12.9, 8.3, 4.0)	26.2, CH_2	2.35 (m)	26.2, CH_2
5b	2.05 (tdd, 13.6, 7.5, 3.7)		2.05 (m)	
6	5.42 (t, 7.9)	124.8, CH	5.42 (t, 8.0)	124.8, CH
7	-	134.1, C	-	134.2, C
8a	2.16 (dt, 12.2, 3.4)	41.2, CH_2	2.15 (dt, 12.2, 3.4)	41.2, CH_2
8b	1.95 (overlapped)		1.94 (overlapped)	
9a	1.55 (tdd, 9.2, 6.5)	31.0, CH_2	1.54 (m)	31.0, CH_2
9b	1.22 (overlapped)		1.21 (m)	
10	1.98 (overlapped)	50.1, CH	1.97 (overlapped)	50.0, CH_2
11	-	153.5, C	-	153.5, C
12a	2.24 (dtd, 13,1, 3.4, 1.2)	33.9, CH_2	2.23 (dt, 13,1, 3.4)	33.9, CH_2
12b	1.94 (overlapped)		1.93 (overlapped)	
13a	1.66 (overlapped)	26.4, CH_2	1.65 (overlapped)	26.2, CH_2
13b	1.08 (qd 12.8, 3.6)		1.07 (td 13.2, 3.7)	
14	1.21 (overlapped)	42.9, CH	-	42.2, CD
15	1.27 (dp 9.2, 6.5)	30.6, CH	1.26 (overlapped)	30.5, CH
16	0.83 (dd, 8.1,6.6)	21.2, CH_3	0.81 (t, 6.7)	21.2, CH_3
17	0.83 (dd, 8.1,6.6)	22.2, CH_3	0.83 (t, 6.7)	22.1, CH_3
18	4.56 (d, 1.8)	107.1, CH_2	4.55 (d, 1.7)	107.1, CH_2
19	1.71(d, 1.6)	16.3, CH_3	1.71 (overlapped)	16.3, CH_3
20	1.71(d, 1.6)	24.5, CH_3	1.71 (overlapped)	24.5, CH_3

Supplementary Table 6. ^{1}H (600 MHz) and ^{13}C NMR (150 MHz) data of 2 inCDCl$_3$.

**No**.	δ_H (mult, J in Hz)	δ_C (mult)	**No**.	δ_H (mult, J in Hz)	δ_C (mult)
1	5.77 (d, 11.4)	119.7, CH	11	-	133.0, C
2	6.21 (d, 11.4)	122.2, CH	12	2.19 (2H, overlapped)	35.5, CH_2
3	-	134.8, C	13	2.19 (2H, overlapped)	26.1, CH_2
4	2.18 (2H, overlapped)	32.0, CH_2	14	-	142.3, C
5	2.16 (2H, overlapped)	25.4, CH_2	15	3.07 (hept, 7.0)	29.1, CH
6	4.99 (t, 7.4)	124.9, CH	16	1.01 (d, 6.9)	21.4, CH_3
7	-	132.8, C	17	1.02 (d, 6.9)	21.4, CH_3
8a	2.02 (m)	39.7, CH_2	18	1.80 (s)	24.0, CH_3
8b	2.10 (overlapped)		19	1.60 (s)	15.6, CH_3
9	2.16 (2H, overlapped)	25.0, CH_2	20	1.59 (s)	16.5, CH_3
10	4.92 (t, 7.7)	124.5, CH			

Supplementary Table 7. Crystal cultivation conditions of 1, 1a–1d.

Compound	Culture solvent	Culture temperature
**1**	None	-20°C
**1a**	MeOH	4°C
**1b**	PE/IPA (8:2)	4°C
**1c**	EA	rt
**1d**	PE/DCM (1:1)	rt

Supplementary Table 8. ^{1}H (600 MHz) and ^{13}C NMR (150 MHz) data of 1a in $CDCl_3$.

**No**.	δ_H (mult, *J* in Hz)	δ_C (mult)	**No**.	δ_H (mult, *J* in Hz)	δ_C (mult)
1	2.19 (dt, 13.3, 3.4)	42.5, CH	11	-	152.2, C
2	5.31 (d, 11.8)	128.0, CH	12a	2.23 (m)	33.2, CH_2
3	-	132.1, C	12b	1.96 (m)	
4a	2.70 (td, 13.7, 4.4)	28.7, CH_2	13a	1.68 (m)	26.1, CH_2
4b	1.79 (overlapped)		13b	1.12 (overlapped)	
5a	2.34 (tt, 14.0, 4.8)	28.2, CH_2	14	1.36 (m)	43.1, CH
5b	1.39 (t, 4.3)		15	1.35 (m)	30.5, CH
6	3.07 (dd, 9.5, 5.4)	61.9, CH	16	0.84 (d, 6.2)	20.9, CH_3
7	-	59.6, C	17	0.90 (d, 6.1)	22.5, CH_3
8a	2.62 (dd, 11.9, 3.9)	42.0, CH_2	18a	4.57 (d, 1.9)	107.9, CH_2
8b	1.11 (overlapped)		18b	4.53 (d, 1.9)	
9a	1.78 (overlapped)	32.3, CH_2	19	1.42 (s)	16.3, CH_3
9b	1.39 (overlapped)		20	1.76 (d, 1.5)	23.9, CH_3
10	2.05 (d, 8.2)	50.5, CH			

Supplementary Table 9. ^{1}H (600 MHz) and ^{13}C NMR (150 MHz) data of 1b in $CDCl_3$.

**No**.	δ_H (mult, *J* in Hz)	δ_C (mult)	**No**.	δ_H (mult, *J* in Hz)	δ_C (mult)
1	2.76 (dd, 12.2, 3.4)	42.5, CH	11	-	60.7, C
2	5.47 (d, 12.0)	127.0, CH	12a	1.77 (overlapped)	31.3, CH_2
3	-	133.3, C	12b	1.21 (overlapped)	
4a	2.69 (td, 13.7, 4.5)	28.7, CH_2	13a	1.77 (overlapped)	24.2, CH_2
4b	1.80 (overlapped)		13b	1.34 (overlapped)	
5a	2.30 (m)	28.1, CH_2	14	1.34 (overlapped)	42.6, CH
5b	1.39 (overlapped)		15	1.38 (overlapped)	30.3, CH
6	2.90 (dd, 9.5, 5.4)	61.7, CH	16	0.87 (d, 6.5)	21.0, CH_3
7	-	59.5, C	17	0.90 (d, 6.5)	22.5, CH_3
8a	2.21 (dt, 13.5, 3.5)	41.9, CH_2	18a	2.61 (d, 5.2)	57.5, CH_2
8b	0.99 (td, 13.5, 4.5)		18b	2.56 (dd, 5.2, 1.6)	
9a	1.76 (overlapped)	27.0, CH_2	19	1.41 (s)	16.3, CH_3
9b	1.56 (overlapped)		20	1.78 (d, 1.5)	24.0, CH_3
10	1.11 (d, 8.4)	48.6, CH			

Supplementary Table 10. ^{1}H (600 MHz) and ^{13}C NMR (150 MHz) data of 1c in $CDCl_3$.

**No.**	δ_H (mult, J in Hz)	δ_C (mult)	**No.**	δ_H (mult, J in Hz)	δ_C (mult)
1	2.70 (overlapped)	41.5, CH	11	-	61.4, C
2	5.65 (d, 12.4)	127.6, CH	12a	1.81 (overlapped)	31.2, CH_2
3	-	132.2, C	12b	1.21 (dtd, 13.7, 3.3, 1.8)	
4a	2.68 (overlapped)	28.6, CH_2	13a	1.64 (overlapped)	22.0, CH_2
4b	1.54 (overlapped)		13b	1.50 (overlapped)	
5a	2.30 (m)	28.1, CH_2	14	1.29 (m)	42.7, CH
5b	1.37 (overlapped)		15	1.39 (overlapped)	30.5, CH
6	2.96 (dd, 9.5, 5.4)	61.7, CH	16	0.89 (d, 6.6)	20.9, CH_3
7	-	59.2, C	17	0.90 (d, 6.6)	22.4, CH_3
8a	2.20 (dt, 13.3, 3.4)	42.1, CH_2	18a	2.52 (d, 4.5)	51.8, CH_2
8b	0.97 (td, 13.6, 4.6)		18b	2.46 (d, 4.5)	
9a	1.65 (overlapped)	29.0, CH_2	19	1.41 (s)	16.3, CH_3
9b	1.25 (overlapped)		20	1.79 (d, 1.5)	24.0, CH_3
10	1.07 (d, 8.0)	49.2, CH			

Supplementary Table 11. ^{1}H (600 MHz) and ^{13}C NMR (150 MHz) data of 1d in $CDCl_3$.

**No.**	δ_H (mult, J in Hz)	δ_C (mult)	**No.**	δ_H (mult, J in Hz)	δ_C (mult)
1	1.81 (overlapped)	39.0, CH	11	-	61.0, C
2	3.27 (d, 10.9)	64.1, CH	12a	1.84 (overlapped)	31.1, CH_2
3	-	58.2, C	12b	1.23 (overlapped)	
4a	1.58 (overlapped)	28.3, CH_2	13a	1.38 (overlapped)	22.5, CH_2
4b	1.52 (overlapped)		14	1.19 (m)	44.4, CH
5a	1.38 (overlapped)	25.4, CH_2	15	1.38 (overlapped)	29.0, CH
6	3.00 (dd, 9.8, 5.2)	61.2, CH	16	0.92 (d, 6.4)	21.8, CH_3
7	-	59.2, C	17	0.93 (d, 6.4)	22.5, CH_3
8a	2.19 (overlapped)	41.3, CH_2	18a	2.53 (d, 4.4)	51.2, CH_2
8b	0.97 (overlapped)		18b	2.50 (d, 4.4)	
9a	1.84 (overlapped)	29.9, CH_2	19	1.28 (s)	15.9, CH_3
10	1.19 (overlapped)	45.7, CH	20	1.38 (s)	21.9, CH_3

Supplementary Table 12. ^{1}H (600 MHz) and ^{13}C NMR (150 MHz) data of 3 in C_6D_6.

**No**.	δ_H (mult, *J* in Hz)	δ_C (mult)	**No**.	δ_H (mult, *J* in Hz)	δ_C (mult)
1	2.79 (m)	43.8, CH	10	-	134.6, C
2	4.76 (d, 10.1)	135.7, CH	11	-	128.4, C
3	-	129.1, C	12a	1.91 (overlapped)	29.2, CH_2
4a	2.10 (ddd, 11.9, 5.6, 2.4)	39.4, CH_2		1.87 (overlapped)	
4b	1.96 (overlapped)		13a	1.87 (overlapped)	21.0, CH_2
5a	2.26 (m)	26.0, CH_2	13b	1.56 (overlapped)	
5b	2.00 (overlapped)		14	1.27 (td, 5.1, 2.4)	47.0, CH
6	4.80 (overlapped)	127.2, CH	15	1.64 (overlapped)	27.8, CH
7	-	136.7, C	16	0.99 (d, 6.6)	22.0, CH_3
8a	2.31 (dd, 12.4, 8.4)	42.1, CH_2	17	0.92 (d, 6.6)	20.7, CH_3
8b	2.14 (m)		18	1.62 (s)	19.7, CH_3
9a	2.68 (m)	33.3, CH_2	19	1.54 (s)	16.3, CH_3
9b	1.59 (overlapped)		20	1.54 (s)	16.5, CH_3

Supplementary Table 13. ^{1}H (600 MHz) and ^{13}C NMR (150 MHz) data of 4 in $CDCl_3$.

**No**.	δ_H (mult, *J* in Hz)	δ_C (mult)	**No**.	δ_H (mult, *J* in Hz)	δ_C (mult)
1	2.17 (d, 11.5)	37.5, CH			
2	2.02 (m)	57.1, CH	11	-	123.1, C
3	-	149.2, C	12a	1.96 (m)	27.9, CH_2
4a	2.23 (m)	39.2, CH_2	12b	1.67 (overlapped)	
4b	1.87 (overlapped)		13a	1.56 (overlapped)	21.3, CH_2
5	1.61 (overlapped)	25.2, CH_2	13b	1.44 (ddd, 13.3, 5.7, 3.4)	
6a	1.40 (dtd, 13.1, 3.3, 1.8)	42.6, CH_2	14	2.17 (dt, 10.3, 3.5)	37.5, CH
6b	1.24 (m)		15	1.57 (overlapped)	27.9, CH
7	-	38.4, C	16	0.90 (d, 6.6)	21.5, CH_3
8a	1.57 (overlapped)	44.2, CH_2	17	0.99 (d, 6.6)	21.9, CH_3
8b	1.18 (m)		18	1.62 (d, 1.3)	19.2, CH_3
9a	2.48 (dt, 12.9, 3.5)	27.2, CH_2	19	0.91 (d, 0.8)	18.4, CH_3
9b	1.85 (overlapped)		20	4.91 (d, 1.8)	108.0, CH_2
10	-	133.5, C		4.48 (d, 1.8)	

Supplementary Table 14. ^{1}H (600 MHz) and ^{13}C NMR (150 MHz) data of 5 in $CDCl_3$.

**No**.	δ_H (mult, *J* in Hz)	δ_C (mult)	**No**.	δ_H (mult, *J* in Hz)	δ_C (mult)
1	2.70 (d, 6.9)	28.2, CH_2	10	4.85 (overlapped)	123.5, CH
2	4.95 (m)	124.3, CH	11	-	134.1, C
3	-	132.2, C	12	2.61 (d, 8.0)	37.2, CH_2
			13	5.27 (m)	119.6, CH
4	2.05 (overlapped)	38.4, CH_2	14	-	146.4, C
5	1.64 (overlapped)	24.8, CH_2	15	2.29 (m)	35.6, CH
6	4.85 (overlapped)	126.2, CH	16	1.03 (d, 6.9)	22.3, CH_3
7	-	133.4, C	17	1.03 (d, 6.9)	22.3, CH_3
8	2.12 (overlapped)	39.3, CH_2	18	1.58 (s)	16.2, CH_3
9a	2.13 (overlapped)	24.7, CH_2	19	1.51 (s)	15.3, CH_3
9b	1.64 (overlapped)		20	1.61 (s)	17.5, CH_3

Supplementary Table 15. ^{1}H (600 MHz) and ^{13}C NMR (150 MHz) data of 9 in C_6D_6.

**No**.	δ_H (mult, *J* in Hz)	δ_C (mult)	**No**.	δ_H (mult, *J* in Hz)	δ_C (mult)
1a	2.05 (m)	31.3, CH_2	10	5.34 (m)	124.0, CH
1b	1.96 (m)		11	-	134.9, C
2	5.43 (m)	121.4, CH	12a	2.09 (m)	40.2, CH_2
3	-	133.4, C	12b	2.09 (m)	
4a	1.95 (m)	31.0, CH_2	13a	2.18 (m)	27.2, CH_2
4b	1.86 (m)		13b	2.18 (m)	
5a	1.72 (m)	28.4, CH_2	14	5.23 (m)	124.9, CH
5b	1.49 (m)		15	-	131.2, C
6	2.12 (m)	43.3, CH	16	1.55 (s)	17.8, CH_3
7	-	139.4, C	17	1.67 (s)	25.9, CH_3
8	5.34 (m)	122.4, CH	18	1.62 (s)	16.2, CH_3
9a	2.82 (t, 7.1)	27.4, CH_2	19	1.60 (s)	14.4, CH_3
9b	2.82 (t, 7.1)		20	1.62 (s)	23.7, CH_3

Supplementary Table 16. ^{1}H (600 MHz) and ^{13}C NMR (150 MHz) data of 10 in C_6D_6.

No.	δ_H (mult, *J* in Hz)	δ_C (mult)	No.	δ_H (mult, *J* in Hz)	δ_C (mult)
1	2.68 (overlapped)	39.7, CH	11	1.97 (m)	31.9, CH
2	5.61 (br s)	125.0, CH	12a	1.38 (m)	36.2, CH_2
3	-	134.1, C	12b	1.38 (m)	
4a	1.92 (m)	32.4, CH_2	13a	2.06 (m)	26.7, CH_2
4b	1.92 (m)		13b	2.06 (m)	
5a	2.70 (overlapped)	27.3, CH_2	14	5.23 (m)	125.6, CH
5b	1.94 (m)		15	-	131.0, C
6	-	130.5, C	16	1.57 (s)	17.8, CH_3
7	-	124.2, C	17	1.70 (s)	25.9, CH_3
8	2.02 (m)	32.8, CH_2	18	0.83 (d, 7.0)	14.2, CH_3
9a	1.55 (m)	21.9, CH_2	19	1.65 (s)	18.7, CH_3
9b	1.22 (m)		20	1.67 (s)	23.8, CH_3
10	1.36 (m)	43.6, CH			

Supplementary Table 17. ^{1}H (600 MHz) and ^{13}C NMR (150 MHz) data of 11 in C_6D_6.

No.	δ_H (mult, *J* in Hz)	δ_C (mult)	No.	δ_H (mult, *J* in Hz)	δ_C (mult)
1	2.41 (m)	36.2, CH	11	-	153.0, C
2	5.56 (br s)	126.5, CH	12a	2.15 (t, 8.1)	35.8, CH_2
3	-	133.3, C	12b	2.15 (t, 8.1)	
4a	1.93 (m)	30.8, CH_2	13a	2.25 (m)	27.1, CH_2
4b	1.87 (overlapped)		13b	2.25 (m)	
5a	1.86 (overlapped)	26.1, CH_2	14	5.27 (m)	125.1, CH
5b	1.30 (overlapped)		15	-	131.2, C
6	1.56 (m)	39.7, CH	16	1.57 (s)	17.8, CH_3
7	1.64 (overlapped)	33.3, CH	17	1.68 (s)	25.9, CH_3
8a	1.61 (overlapped)	28.4, CH_2	18a	5.01 (s)	109.1, CH_2
8b	1.30 (overlapped)		18b	4.99 (s)	
9a	1.61 (overlapped)	27.5, CH_2	19	1.02 (d, 7.0)	19.8, CH_3
9b	1.49 (m)		20	1.64 (s)	23.9, CH_3
10	2.06 (m)	47.6, CH			

Supplementary Table 18. ^{1}H (600 MHz) and ^{13}C NMR (150 MHz) data of 12 in C_6D_6.

**No.**	δ_H (mult, J in Hz)	δ_C (mult)	**No.**	δ_H (mult, J in Hz)	δ_C (mult)
1a	2.15 (m)	31.9, CH_2	10	5.29 (m)	124.8, CH
1b	1.98 (m)		11	-	135.1, C
2	5.43 (m)	121.3, CH	12a	2.10 (overlapped)	40.2, CH_2
3	-	133.5, C	12b	2.10 (overlapped)	
4a	1.94 (m)	31.0, CH_2	13a	2.19 (overlapped)	27.2, CH_2
4b	1.86 (m)		13b	2.19 (overlapped)	
5a	1.79 (m)	28.7, CH_2	14	5.24 (m)	125.0, CH
5b	1.49 (m)		15	-	131.2, C
6	2.14 (overlapped)	40.2, CH	16	1.57 (s)	17.8, CH_3
7	-	154.1, C	17	1.67 (s)	25.9, CH_3
8a	2.13 (overlapped)	35.4, CH_2	18	1.60 (s)	16.2, CH_3
8b	2.13 (overlapped)		19a	4.90 (s)	107.8, CH_2
9a	2.23 (m)	27.2, CH_2	19b	4.90 (s)	
9b	2.11 (overlapped)		20	1.62 (s)	23.7, CH_3

Supplementary Table 19. Energies, enthalpies, and free energies of the structures calculated at the mPW1PW91/6-31+G(d,p)//B3LYP/6-31+G(d,p).

Structures	ZPE	DH	DG	E	H	G	Imaginary	ΔG
A^+	0.480259	0.505983	0.425503	-781.55874	-781.052757	-781.133237		0
TS^{A_B}	0.480943	0.505564	0.42837	-781.560891	-781.055327	-781.132521	-140.5	0.449294224
B^+	0.481443	0.506486	0.426924	-781.571212	-781.064726	-781.144288		-6.934567701
TS^{B_C}	0.479088	0.503579	0.425888	-781.557516	-781.053937	-781.131628	-476.11	1.009656993
C^+	0.482002	0.506588	0.429019	-781.579757	-781.073169	-781.150738		-10.98198076
TS^{C_D}	0.480457	0.504578	0.42851	-781.573167	-781.068589	-781.144657	-602.45	-7.166117378
D^+	0.483399	0.507992	0.4301	-781.59493	-781.086938	-781.16483		-19.8247939
TS^{D_E}	0.484817	0.507718	0.436178	-781.567483	-781.059765	-781.131305	-153.97	1.212341399
E^+	0.485012	0.508115	0.435678	-781.590894	-781.082779	-781.155216		-13.79195218
TS^{A_F}	0.480457	0.505265	0.426207	-781.555534	-781.050269	-781.129327	-111.67	2.453548069
F^+	0.482055	0.506614	0.429102	-781.566603	-781.059989	-781.137501		-2.675685158
TS^{F_M}	0.480459	0.504285	0.428949	-781.558407	-781.054122	-781.129458	-461.9	2.371344796
M^+	0.484196	0.508339	0.430822	-781.596766	-781.088427	-781.165944		-20.52383547
TS^{M_N}	0.484643	0.507498	0.436212	-781.586606	-781.079108	-781.150394	-214.94	-10.76611873
N^+	0.485482	0.508343	0.437351	-781.59488	-781.086537	-781.157529		-15.24337332
TS^{N_O}	0.482726	0.50557	0.433958	-781.572146	-781.066576	-781.138188	-533.12	-3.106781711
O^+	0.486081	0.509039	0.43736	-781.588054	-781.079015	-781.150694		-10.9543705
TS^{O_P}	0.483925	0.506302	0.435509	-781.583923	-781.077621	-781.148414	-283.22	-9.523657044
P^+	0.487235	0.509912	0.438948	-781.613838	-781.103926	-781.17489		-26.13750325
TS^{P_E}	0.485088	0.507267	0.438131	-781.565312	-781.058045	-781.127181	-343.57	3.80017573
TS^{F_G}	0.479831	0.504016	0.426138	-781.556368	-781.052352	-781.13023	-487.11	1.886910241
G^+	0.481696	0.506265	0.428632	-781.574316	-781.068051	-781.145684		-7.810565937
TS^{G_Q}	0.480058	0.503826	0.427698	-781.552854	-781.049028	-781.125156	-705.25	5.070875178
Q^+	0.48405	0.508091	0.430571	-781.594676	-781.086585	-781.164105		-19.36985212
TS^{Q_R}	0.48506	0.507817	0.437242	-781.564011	-781.056194	-781.126769	-173.58	4.058708161

R^+	0.485768	0.508633	0.438109	-781.570084	-781.061451	-781.131975		0.791912446
TS^{R_S}	0.483544	0.506115	0.435586	-781.558736	-781.052621	-781.12315	-502.73	6.329652013
S^+	0.487632	0.510247	0.440253	-781.592348	-781.082101	-781.152095		-11.83350626
TS^{S_T}	0.484181	0.506392	0.436684	-781.571359	-781.064967	-781.134675	-331.49	-0.902353484
T^+	0.487917	0.510537	0.439442	-781.59831	-781.087773	-781.158868		-16.08360372
TS^{T_L}	0.484443	0.506686	0.436821	-781.575221	-781.068535	-781.1384	-107.42	-3.239812962
L^+	0.486387	0.509412	0.437338	-781.598595	-781.089183	-781.161257		-17.58271532
TS^{L_E}	0.483197	0.50582	0.434358	-781.581795	-781.075975	-781.147437	-437.3	-8.91058378
TS^{G_H}	0.479814	0.504007	0.426096	-781.538947	-781.03494	-781.112851	-804.11	12.79861
H^+	0.483652	0.508186	0.430521	-781.573412	-781.065226	-781.142891		-6.05167
TS^{H_I}	0.484085	0.507147	0.434634	-781.561459	-781.054312	-781.126825	-220.89	4.029843
I^+	0.484876	0.507949	0.436417	-781.587366	-781.079417	-781.150949		-11.1081
TS^{I_J}	0.482706	0.505615	0.433497	-781.560282	-781.054667	-781.126785	-522.96	4.054943
J^+	0.484597	0.507869	0.434857	-781.580626	-781.072757	-781.145769		-7.85763
TS^{J_K}	0.483985	0.506588	0.435472	-781.574822	-781.068234	-781.13935	-588.34	-3.82967
K^+	0.488049	0.510638	0.440152	-781.612589	-781.101951	-781.172437		-24.592
TS^{K_L}	0.480286	0.503051	0.43174	-781.505076	-781.002025	-781.073336	-1216.98	37.59451

Supplementary References

1. Breitmaier E. & Voelter W. Carbon-^{13}NMR spectroscopy. 3rd ed. New York: VCH; **1987**.
2. Bloor, S. J. et al. Diterpenoids from the gorgonian *Solenopodium stechei*. *J. Org. Chem.* **57**, 1205–1216 (1992).
3. Chen, B. et al. Klysimplexins I-T, eunicellin-based diterpenoids from the cultured soft coral *Klyxum simplex*. *Org. Biomol. Chem.* **9**, 834–44 (2011).
4. Chen, Y. et al. Discovery of new eunicellins from an Indonesian octocoral *Cladiella* sp. *Mar. Drugs* **9**, 934–943 (2011).
5. Zhu, C. et al. Discovery and biosynthesis of a structurally dynamic antibacterial diterpenoid. *Angew. Chem. Int. Ed.* **60**, 14163–14170 (2021).
6. Xu, B., Tantillo, D. J. & Rudolf, J. D. Mechanistic insights into the formation of the 6,10-bicyclic eunicellane skeleton by the bacterial diterpene synthase Bnd4. *Angew. Chem. Int. Ed.* **60**, 23159–23163 (2021).
7. Li, Z. et al. First trans-eunicellane terpene synthase in bacteria. *Chem* **9**, 698–708 (2023).
8. Li, Z. et al. Cryptic isomerization in diterpene biosynthesis and the restoration of an evolutionarily defunct P450. *J. Am. Chem. Soc.* **145**, 22361–22365 (2023).
9. Kodama, K. et al. (-)-Axinyssene: a novel cytotoxic diterpene from a Japanese marine sponge *Axinyssa* sp. *Org. Lett.* **5**, 169–171 (2003).
10. Yamada, Y. et al. Novel terpenes generated by heterologous expression of bacterial terpene synthase genes in an engineered *Streptomyces* host. *J. Antibiot.* **68**, 385–394 (2015).
11. Wiemer, D. et al. Biflora-4,10(19),15-triene: a new diterpene from a termite soldier (*Isoptera Termitidae Termitinae*). *J. Org. Chem.* **45**, 191–192 (1980).
12. Jin, Y. et al. Taxadiene synthase-catalyzed cyclization of 6-fluorogeranylgeranyl disphosphate to 7-fluoroverticillenes. *J. Am. Chem. Soc.* **127**, 7834–7842 (2005).
13. Xu, B. et al. Mutation of the eunicellane synthase Bnd4 alters its product profile and expands its prenylation ability. *Org. Biomol. Chem.* **38**, 8833–8837 (2022).
14. Jo, S. et al. CHARMM-GUI: a web-based graphical user interface for CHARMM. *J. Comput. Chem.* **29**, 1859–1865 (2008).
15. S. Kim, S. et al. CHARMM-GUI ligand reader and modeler for CHARMM Force Field Generation of small molecules. *J. Comput. Chem.* 38, 1879–1886 (2017).
16. Huang, J. & MacKerell Jr, A. D. CHARMM36 all-atom additive protein force field: validation based on comparison to NMR data. *J. Comput. Chem.* **34**, 2135–2145 (2013).
17. Abraham, M. et al. GROMACS: High performance molecular simulations through multi-level parallelism from laptops to supercomputers. *SoftwareX* **1**, 19–25 (2015).
18. Essmann, U. et al. A smooth particle mesh Ewald potential. *J. Chem. Phys.* **103**, 577–8593 (1995).
19. Hess, B. et al. LINCS: A Linear Constraint Solver for molecular simulations. *J. Comput. Chem.* 18, 1463–1472 (1998).
20. Tribello, G. et al. Plumed 2: New feathers for an old bird. *Comput. Phys. Commun.* 185, 604–

613 (2014).

21. Valsson, O. and Parrinello, M. Well-Tempered Variational Approach to Enhanced Sampling. *J. Chem. Theory Comput.* **11**, 1996–2002 (2015).
22. Pinto, A. et al. The isolation of novel diterpenoids, including a C40 *bis*-diterpenoid, from the Brazilian plant *Vellozia magdalenae* (Velloziaceae). *Tetrahedron* **53**, 2005–2012 (1997).
23. Zhang, B. et al. Bisyinshanic acids A and B, two novel diterpene dimers from the roots of *Euphorbia yinshanica*. *Helv. Chim. Acta*. **95** (2012).
24. Li, G., Dickschat, J. S. & Guo, Y. W. Diving into the world of marine 2,11-cyclized cembranoids: a summary of new compounds and their biological activities. *Nat. Prod. Rep.* **37**, 1367–1383 (2020).
25. Welford, A. J. & Collins, I. The 2,11-cyclized cembranoids: cladiellins, asbestinins, and briarellins (period 1998-2010). *J. Nat. Prod.* **74**, 2318–2328 (2011).
26. Cóbar, O. M. Survey of 2,11-cyclized cembranoids from Caribbean sources. *Nat. Prod. Res.* **23**, 26–43 (2009).
27. Ma, L. F. et al. *Streptomyces albogriseolus* SY67903 produces eunicellin diterpenoids structurally similar to terpenes of the gorgonian *Muricella sibogae*, the bacterial source. *J. Nat. Prod.* **83**, 1641–1645 (2020).
28. Li, Z. & Rudolf, J. D. Biosynthesis, enzymology, and future of eunicellane diterpenoids. *J. Ind. Microbiol. Biotechnol.* **50**, kuad027 (2023).
29. Scesa, P. D., Lin, Z. & Schmidt, E. W. Ancient defensive terpene biosynthetic gene clusters in the soft corals. *Nat. Chem. Biol.* **18**, 659–663 (2022).
30. Burkhardt, I. et al. Ancient plant-like terpene biosynthesis in corals. *Nat. Chem. Biol.* **18**, 664–669 (2022).
31. Shen, T. et al. When homologous sequences meet structural decoys: accurate contact prediction by tFold in CASP14 (tFold for CASP14 contact prediction). *Proteins: Struct., Funct., Bioinf.* **89**, 1901–1910 (2021).
32. Baek, M. et al. Accurate prediction of protein structures and interactions using a three-track neural network. *Science* **373**, 871–876 (2021).
33. Jumper, J. et al. Highly accurate protein structure prediction with AlphaFold. *Nature* **596**, 583–589 (2021).
34. Schotte, C. et al. Understanding and engineering the stereoselectivity of humulene synthase. *Angew. Chem. Int. Ed.* **60**, 20308–20312 (2021)
35. Schotte, C. et al. Understanding and engineering the stereoselectivity of humulene synthase. *Angew. Chem. Int. Ed.* **60**, 20308–20312 (2021).
36. Chen, Y. et al. A cryptic plant terpene cyclase producing unconventional 18- and 14-membered macrocyclic C25 and C20 terpenoids with immunosuppressive activity. *Angew. Chem. Int. Ed.* **60**, 25468–25476 (2021).
37. Tao, H. et al. Discovery of non-squalene triterpenes. *Nature* **606**, 414–419 (2022).
38. Xu, B. et al. Bacterial diterpene synthases prenylate small molecules. *ACS Catal.* **11**, 5906–5915 (2021).
