# Supplementary material for: Discovery of a terpene synthase synthesizing a nearly non-flexible eunicellane reveals the basis of flexibility
Source: Nat Commun. 2024 Jul 15;15:5940. doi: 10.1038/s41467-024-50209-z (PMC11250809; doi:10.1038/s41467-024-50209-z)
Supplement: Supplementary file 3 — Description of Additional Supplementary Files [file 41467_2024_50209_MOESM3_ESM.pdf]

Description of Additional Supplementary Files

Supplementary Data 1. Cartesian coordinates for conformations of 1, 6–8. This document provides supplementary data corresponding to Figure 3b in the main text.

Supplementary Data 2. Cartesian coordinates for the optimized structures of intermediates and transition. This document provides supplementary data corresponding to Figure 4b in the main text and Supplementary Fig. 25.

Supplementary Data 3. The global score and each residue score of MicA predicted models. The term "Af_MicA.pdb" represents model of MicA, which was predicted using AlphaFold computational methods. Similarly, "Tf_MicA.pdb" represents the MicA model predicted using tFold, while "Rf_MicAl.pdb" represents the MicA model predicted using RoseTTAFold. This document provides supplementary data corresponding to Figure 5a in the main text, Supplementary Fig.s 31 and 32.
