# Supplementary material for: Discovery of a terpene synthase synthesizing a nearly non-flexible eunicellane reveals the basis of flexibility
Source: Nat Commun. 2024 Jul 15;15:5940. doi: 10.1038/s41467-024-50209-z (PMC11250809; doi:10.1038/s41467-024-50209-z)
Supplement: Supplementary file 4 — Supplementary Data 1 [file 41467_2024_50209_MOESM4_ESM.docx]

**Supplementary data 1. Cartesian coordinates for conformations of 1, 6–8.** This document provides supplementary data corresponding to Figure 3b in the main text.

**Conformation of 1_1 (99.3%)**

C 2.62719400 1.99407100 0.34110300

C 2.92150000 0.48356100 0.32802100

C 1.87182500 -0.28356000 -0.49892300

C 0.41935600 -0.02473700 0.02877700

C 0.11327700 1.51218300 0.21170200

C 1.24908400 2.25789800 0.88773600

C 1.03702500 3.06926300 1.93346800

C 2.19530200 -1.79758300 -0.65775600

C 3.66556700 -2.06334700 -1.03243800

C 1.29093600 -2.47034700 -1.70593700

C -0.35012900 2.17920700 -1.11897500

C -1.90241300 2.23458000 -1.24259000

C -2.55434100 0.86927200 -1.14158800

C -2.95395100 0.40176100 0.05522100

C -3.30457500 -1.02391900 0.40906700

C 0.07204200 -0.75175400 1.30929500

C -1.00425900 -1.52775300 1.54801000

C -2.03931300 -1.93073700 0.50649000

C -2.59307400 0.06538300 -2.41993900

C -1.25844000 -2.08087500 2.93361800

H -0.26525900 -0.37201000 -0.74586500

H -0.73688600 1.57202800 0.89596700

H 2.69567300 2.36798600 -0.69256700

H 3.38199000 2.53179700 0.92549700

H 2.94998700 0.10584500 1.35961400

H 3.91944100 0.32649500 -0.09138900

H 1.91055400 0.14808500 -1.51391900

H 0.04252400 3.22192500 2.34571600

H 1.85241000 3.60992700 2.40813400

H 2.00514000 -2.28223000 0.31027300

H 3.94873800 -1.51858000 -1.94295300

H 4.36187500 -1.77745700 -0.23930100

H 3.81788300 -3.13062300 -1.22843900

H 1.43517900 -2.01303300 -2.69384200

H 1.53266300 -3.53509800 -1.79740000

H 0.23050600 -2.39978500 -1.45496300

H 0.07518300 1.65353600 -1.98344500

H 0.02250700 3.20905800 -1.17083600

H -2.16073900 2.71769600 -2.19445900

H -2.28042400 2.88324900 -0.44309800

H -2.89788500 1.08801200 0.89911000

H -3.97655900 -1.47081200 -0.33439800

H -3.84626500 -1.04372700 1.36183900

H 0.73119600 -0.55220000 2.15557400

H -1.58373800 -1.96750100 -0.48661300

H -2.37334500 -2.95358800 0.72913000

H -3.16150100 0.60598200 -3.18812000

H -1.58611100 -0.08749300 -2.83128800

H -3.05382600 -0.91682000 -2.29262500

H -1.24496000 -3.17933800 2.92941100

H -0.51285900 -1.73309700 3.65458800

H -2.24876400 -1.78362600 3.30448400

**Conformation of 1*R*_10*R*_1_1 (97.5%)**

C 2.86629500 -1.72388500 0.20403800

C 2.68131100 -0.49707100 -0.71979400

C 1.84133800 0.58942500 -0.02522200

C 0.38561500 0.07894500 0.22991300

C 0.34656400 -1.46460700 0.60006700

C 1.69128700 -1.90057200 1.14801300

C 1.83515000 -2.38274200 2.38971200

C 1.86259700 1.96593800 -0.74652400

C 3.19431300 2.70372200 -0.52432800

C 1.54155300 1.88928600 -2.24982200

C -0.10645100 -2.39008900 -0.57049400

C -1.64249200 -2.58545400 -0.68326200

C -2.39680100 -1.30750900 -0.98419300

C -2.94643500 -0.60293900 0.01931400

C -3.44378400 0.81936500 -0.03274600

C -0.26615200 0.88905400 1.32808500

C -1.38484700 1.63688500 1.29100600

C -2.25430500 1.82381400 0.05974700

C -2.37956800 -0.85981100 -2.42675400

C -1.88248800 2.33922600 2.53536800

H 3.78568700 -1.62293900 0.79203800

H 2.99013300 -2.62862400 -0.40724300

H 2.19198000 -0.79885200 -1.65327500

H 3.66386500 -0.10589000 -1.00574000

H 2.29390000 0.75465500 0.96497500

H -0.17960900 0.20203800 -0.69674800

H -0.38992000 -1.59009000 1.40153300

H 2.80821900 -2.67206200 2.78024600

H 0.98380900 -2.50867900 3.05388200

H 1.07906500 2.57253600 -0.27244400

H 3.40558600 2.82925100 0.54370000

H 4.03832900 2.16475900 -0.97157700

H 3.16817000 3.70082100 -0.97882700

H 1.44453100 2.89658700 -2.67031200

H 0.60288300 1.36083500 -2.44862700

H 2.33591800 1.37865000 -2.80656000

H 0.33229900 -3.38397200 -0.41726100

H 0.29162000 -2.02493600 -1.52485800

H -1.83260600 -3.33431600 -1.46483700

H -2.00348700 -3.01319200 0.26010000

H -2.89572900 -1.04037900 1.01562800

H -3.99687900 1.02701200 -0.95630500

H -4.14248600 0.99950300 0.79267300

H 0.25671300 0.82687500 2.28565800

H -1.65450500 1.72222600 -0.84987100

H -2.65861700 2.84533500 0.05609400

H -2.82784500 -1.63019900 -3.06801500

H -1.35397500 -0.71369300 -2.79133400

H -2.92597500 0.07153800 -2.59093400

H -1.24838100 2.13035500 3.40193000

H -1.91202900 3.42738900 2.38805200

H -2.90687100 2.03156100 2.78551400

**Conformation of 1*R*_10*S*_1_1 (75.4%)**

C -2.86892800 1.52640600 0.64458800

C -2.62852900 0.15819000 1.30764700

C -1.80039500 -0.78187700 0.40882400

C -0.43464600 -0.09410800 0.05384000

C -0.66038400 1.31131800 -0.61912900

C -1.55928200 2.18066400 0.25759100

C -1.25557100 3.41306200 0.69041900

C -2.57532200 -1.30934400 -0.83931000

C -3.95805300 -1.87844200 -0.46891700

C -1.78166300 -2.39114400 -1.59604700

C 0.61023900 2.05673500 -1.09066400

C 1.59352800 1.31001200 -2.03806200

C 2.58021100 0.44372600 -1.28057900

C 2.45149300 -0.89353700 -1.28268800

C 2.97389600 -1.85293900 -0.25360300

C 0.47677000 -0.02257900 1.26197900

C 1.44994900 -0.88856800 1.60794800

C 1.84444000 -2.11555400 0.80087900

C 3.61610200 1.18980200 -0.47031600

C 2.26403800 -0.66868400 2.86428700

H -1.24600700 1.10123800 -1.52863300

H 0.05316200 -0.71583300 -0.69227100

H -3.48797400 1.39120900 -0.25490800

H -3.43199300 2.18269800 1.31724700

H -2.08246700 0.31530400 2.24583200

H -3.58211600 -0.30082200 1.58570600

H -1.55155100 -1.67145300 1.00636700

H -1.95286600 3.96710700 1.31397200

H -0.32730100 3.91714200 0.44599200

H -2.74340700 -0.47728200 -1.53687700

H -3.86952500 -2.65813800 0.29885700

H -4.43132700 -2.33223700 -1.34673400

H -4.64172500 -1.11308100 -0.09128600

H -1.56685100 -3.24560300 -0.94124100

H -0.82965700 -2.02415700 -1.98958200

H -2.35961500 -2.76599200 -2.44800700

H 0.26421200 2.95737100 -1.61211700

H 1.16817000 2.41368700 -0.21876100

H 1.03067600 0.70426100 -2.75968400

H 2.13211900 2.07206400 -2.61879100

H 1.70697100 -1.32159100 -1.95612900

H 3.24792800 -2.81523300 -0.70410700

H 3.86554000 -1.47117400 0.25372200

H 0.30871700 0.82350000 1.92928800

H 0.97764000 -2.53002300 0.27505800

H 2.19177300 -2.89845300 1.48781100

H 4.13357500 1.91798200 -1.10873100

H 4.37255500 0.52648100 -0.04624000

H 3.16969000 1.75393600 0.35690500

H 3.33854200 -0.62239700 2.64054300

H 2.12956200 -1.49835400 3.57149000

H 1.98618700 0.25900600 3.37319900

**Conformation of 1*R*_10*S*_1_2 (10.4%)**

C -2.84864200 2.08255600 -0.01389200

C -2.95424200 0.66663400 -0.61963600

C -2.00941100 -0.29522100 0.12122100

C -0.50312100 0.10676400 -0.10187400

C -0.37943500 1.66401600 -0.39335200

C -1.42994400 2.39073500 0.43309900

C -1.17543200 3.19661000 1.47251900

C -2.27243600 -1.79624100 -0.17705800

C -2.31135400 -2.13413400 -1.67790600

C -3.55137500 -2.29900200 0.51556700

C 1.01699600 2.31671200 -0.30328500

C 2.08655300 1.86301600 -1.33727100

C 2.83147300 0.62464300 -0.88979400

C 2.51776700 -0.57739000 -1.39952000

C 2.77971400 -1.91467700 -0.77204900

C 0.31381100 -0.35443800 1.08264000

C 1.14914800 -1.40831900 1.17557600

C 1.50540400 -2.34216900 0.02959400

C 3.83691600 0.84280300 0.21724700

C 1.83947500 -1.72468500 2.48484200

H -0.69453300 1.77847300 -1.44216500

H -0.14902800 -0.40093400 -1.00194400

H -3.16472100 2.82683100 -0.75903000

H -3.53173100 2.18387300 0.83766700

H -3.99138100 0.31952100 -0.55863300

H -2.70399100 0.68971100 -1.68733200

H -2.20790000 -0.15848700 1.19510900

H -1.98581000 3.64780800 2.04032600

H -0.16814400 3.44411300 1.79069400

H -1.43671200 -2.35046100 0.26940400

H -3.18724100 -1.69209000 -2.16674200

H -2.36877900 -3.21860000 -1.82474700

H -1.42139700 -1.77835700 -2.20875100

H -4.44896000 -1.80446700 0.12537400

H -3.51353500 -2.12078100 1.59625000

H -3.68053400 -3.37619000 0.35928300

H 0.85612100 3.39244500 -0.44324800

H 1.42254400 2.20378200 0.70782500

H 1.60644000 1.69028200 -2.30902300

H 2.79052800 2.69526100 -1.47617500

H 1.80847600 -0.59740300 -2.22887800

H 2.97803600 -2.68323500 -1.52968400

H 3.64609500 -1.89471900 -0.10367300

H 0.15081300 0.23001800 1.99077200

H 0.67567300 -2.42215000 -0.67929100

H 1.66935500 -3.35225500 0.42805500

H 4.56229300 1.60941500 -0.08591600

H 4.39596300 -0.06088800 0.46687500

H 3.36189500 1.20413000 1.13725700

H 2.93118200 -1.74674400 2.36589200

H 1.54723000 -2.71680300 2.85470000

H 1.60057100 -0.99036700 3.25974500

**Conformation of 1*R*_10*S*_1_3 (9.7%)**

C 3.00234200 -1.37711700 -0.10135200

C 2.71360000 -0.46083700 1.10217500

C 1.68316000 0.64130100 0.77797700

C 0.36621500 -0.00805000 0.21396000

C 0.67896100 -0.93616400 -1.02532500

C 1.72047800 -1.97850400 -0.63439300

C 1.54999000 -3.30630400 -0.70201100

C 2.22242100 1.79414900 -0.12726100

C 3.66058600 2.22035300 0.21943800

C 1.31039700 3.03309800 -0.04653200

C -0.54673300 -1.53478300 -1.75248000

C -1.46999900 -0.54458100 -2.50211600

C -2.31574200 0.37171200 -1.62223900

C -2.94901900 -0.12461900 -0.54838300

C -3.48047500 0.63292600 0.63923500

C -0.37932400 -0.73544000 1.31426900

C -1.50054500 -0.35686700 1.95840600

C -2.30408800 0.89372200 1.63627100

C -2.32224100 1.82894700 -2.01501500

C -2.08546700 -1.22104000 3.05376200

H 1.17886000 -0.28262700 -1.75819800

H -0.26664100 0.79879700 -0.15424900

H 3.48761600 -0.79233100 -0.89727000

H 3.70415000 -2.16880700 0.18351300

H 2.31932000 -1.07751200 1.91881100

H 3.64397800 -0.02449800 1.47822600

H 1.41093500 1.11264400 1.73365400

H 2.34336800 -3.98375600 -0.39592600

H 0.63468900 -3.76716300 -1.05738800

H 2.22927600 1.45364200 -1.17199900

H 3.74628200 2.49918500 1.27761600

H 3.94977000 3.09446200 -0.37486100

H 4.39360000 1.43367000 0.01916000

H 1.32742900 3.45674600 0.96577800

H 0.26888000 2.80951100 -0.29320500

H 1.65010000 3.81273600 -0.73757900

H -0.16019200 -2.23565100 -2.50231300

H -1.13734200 -2.13662900 -1.05336900

H -0.85429100 0.07357300 -3.16979500

H -2.12208600 -1.12873300 -3.16741500

H -2.88820100 -1.19917400 -0.39103900

H -3.92550800 1.59615300 0.36704600

H -4.26656700 0.05333300 1.13760000

H 0.06860600 -1.67796700 1.62953100

H -1.65742700 1.66111000 1.20048400

H -2.71061100 1.31634000 2.56516900

H -2.67956300 1.93928500 -3.04790400

H -1.30940900 2.25380600 -1.99577200

H -2.96080400 2.44285800 -1.37548000

H -3.11943900 -1.51261700 2.82381600

H -2.12153500 -0.67653000 4.00711800

H -1.50434500 -2.13527400 3.20593000

**Conformation of 1*S*_10*R*_1_1 (76.1%)**

C 2.86970400 1.84316600 -0.01942700

C 2.95790200 0.32074000 0.20280100

C 1.85965400 -0.42607500 -0.57691400

C 0.43955100 0.12513000 -0.18893900

C 0.38755800 1.66652400 -0.52916900

C 1.48401600 2.38520400 0.24951800

C 1.28524300 3.38738000 1.11697400

C 1.94479300 -1.97753200 -0.48915300

C 3.38541900 -2.52019600 -0.48526200

C 1.17270200 -2.64176800 -1.64513500

C -0.99011100 2.35617300 -0.42305000

C -2.08676900 1.88025100 -1.40471400

C -2.66162000 0.49520700 -1.12303500

C -2.97935600 0.14230800 0.13204700

C -3.16932000 -1.25135500 0.66942100

C 0.07620900 -0.15361900 1.25543600

C -0.84222200 -1.00269800 1.75822600

C -1.76504400 -1.88436000 0.93125900

C -2.76477800 -0.42514200 -2.31490500

C -1.05195300 -1.10162600 3.25387600

H 0.68425500 1.72699300 -1.59015200

H -0.28712900 -0.36357000 -0.83858600

H 3.60887200 2.36239000 0.60068100

H 3.13217400 2.05066400 -1.06878900

H 3.94805300 -0.01846000 -0.11660100

H 2.88136800 0.09782100 1.27510000

H 2.00416700 -0.16758900 -1.63994900

H 2.12261700 3.84086000 1.64163900

H 0.30353500 3.79257000 1.33716500

H 1.47754100 -2.28290100 0.45674800

H 3.94129800 -2.18810900 -1.37190200

H 3.37409300 -3.61604800 -0.49807500

H 3.94718800 -2.21028000 0.40055600

H 1.66279700 -2.43159900 -2.60477900

H 0.13901700 -2.29242300 -1.72088100

H 1.14251200 -3.73009400 -1.52092500

H -1.36163700 2.29056200 0.60496100

H -0.82586000 3.42328900 -0.61714300

H -2.89336900 2.62736000 -1.40030700

H -1.68031300 1.89720600 -2.42520100

H -2.87918800 0.90350000 0.90270700

H -3.74558800 -1.22271600 1.60152300

H -3.71951100 -1.90250500 -0.01898600

H 0.63804500 0.42998900 1.98451000

H -1.89761200 -2.84895600 1.44040600

H -1.31258000 -2.10315900 -0.03896900

H -1.77710800 -0.62045800 -2.75601900

H -3.36477100 0.04518100 -3.10547000

H -3.22507500 -1.38662200 -2.07530200

H -0.85193200 -2.12035700 3.61274200

H -2.09181800 -0.87563500 3.52667000

H -0.40351500 -0.41317700 3.80349300

**Conformation of 1*S*_10*R*_1_2 (23.9%)**

C 2.61453500 2.20636400 0.24607500

C 2.85577400 0.71320500 0.53043800

C 2.03607200 -0.16159200 -0.43289700

C 0.50366300 0.16838000 -0.30437100

C 0.28364200 1.69087500 -0.65725000

C 1.14512700 2.56314100 0.25450100

C 0.69051100 3.56420700 1.02235200

C 2.33253800 -1.68722900 -0.35221000

C 3.80202600 -2.02326600 -0.04044000

C 1.93531700 -2.39154300 -1.66416500

C -1.17421500 2.19835700 -0.74138000

C -2.17824900 1.42696400 -1.64487200

C -2.83704700 0.28145700 -0.90406300

C -2.49078500 -0.98823800 -1.16966200

C -2.62993300 -2.16895200 -0.25492200

C -0.08889800 -0.18812100 1.04412800

C -0.84204700 -1.25126300 1.39457600

C -1.26091400 -2.38604300 0.47167300

C -3.79320800 0.67883000 0.19883100

C -1.35961000 -1.37976500 2.81190300

H 0.71010700 1.79986200 -1.66876600

H -0.00910700 -0.40549500 -1.07624800

H 3.15844000 2.82814300 0.96569000

H 3.02932200 2.43448400 -0.74852200

H 3.92477100 0.50716700 0.42065800

H 2.59489200 0.48377700 1.57210900

H 2.32457600 0.15224700 -1.45078000

H 1.37429200 4.13487000 1.64588300

H -0.35164900 3.86032400 1.06081100

H 1.72721300 -2.10226500 0.46423800

H 4.48164700 -1.57754600 -0.77835100

H 3.95292900 -3.10841600 -0.07097400

H 4.10779500 -1.67864000 0.95161300

H 2.60267200 -2.08405100 -2.47958000

H 0.91207300 -2.16134100 -1.97657700

H 2.01334300 -3.48023100 -1.56607300

H -1.59483800 2.26297300 0.26688400

H -1.11649200 3.23116700 -1.10573800

H -2.93918100 2.14472200 -1.98197000

H -1.67124300 1.05867900 -2.54568300

H -1.83547700 -1.15910500 -2.02548300

H -3.42366000 -2.02782800 0.48524300

H -2.86621500 -3.08573200 -0.81007200

H 0.11542600 0.53599100 1.83356400

H -1.32663300 -3.31167700 1.05883300

H -0.50123300 -2.56281700 -0.29385000

H -4.50954000 1.42057400 -0.17740800

H -3.27869800 1.13867900 1.05104500

H -4.36622700 -0.16936500 0.57899100

H -0.96442000 -2.28317600 3.29581500

H -2.45403200 -1.47303700 2.82804400

H -1.08664100 -0.51755400 3.42718500

**Conformation of 2*E*_1_1 (80.3%)**

C 1.56144500 -1.60649600 2.20463000

C 3.36660200 1.44887400 0.76677300

C -1.84567500 3.37821700 0.72504900

C -2.56677900 -1.27379100 -1.93631100

C -3.21987000 -2.86835100 -0.10545600

C -2.18077100 -1.82122100 -0.55062700

C 1.45490600 -1.56444800 0.69934900

C 2.54812900 -2.28590900 -0.07115000

C 3.74204700 -1.33905600 -0.47236500

C 0.54006300 -0.85993900 0.00844500

C 3.18217400 -0.13860100 -1.17611700

C 2.85060100 1.02512500 -0.59139200

C 1.84929100 1.96463300 -1.22708300

C 0.59896300 2.29682100 -0.35739600

C -1.91774200 -0.78027400 0.58529700

C -0.56777500 0.04061700 0.49171400

C -0.65821000 1.38148500 -0.34232800

C -1.87743600 2.16205400 0.15916100

C -3.17995200 1.40791300 0.05920800

C -3.11608000 0.13184600 0.91754800

H -0.86939100 1.10469800 -1.38672100

H -0.35486900 0.35970300 1.52131000

H 2.56324400 -1.30510100 2.53903500

H 1.40650600 -2.62922600 2.57387400

H 0.83268600 -0.96170400 2.70238300

H 3.55985400 2.52880600 0.78055200

H 4.29786300 0.94406900 1.03303200

H 2.64072700 1.24700800 1.56430500

H -0.93859500 3.95406600 0.86601400

H -2.76400900 3.84229100 1.07578800

H -3.55148300 -0.79354300 -1.92645400

H -2.61932200 -2.09733600 -2.65826000

H -1.84259700 -0.54996400 -2.32116900

H -4.22320700 -2.43863800 -0.00245500

H -2.94868600 -3.31892200 0.85618900

H -3.28929900 -3.67579700 -0.84332900

H -1.23673400 -2.36516800 -0.67366200

H 2.94858000 -3.12769600 0.50781100

H 2.13220900 -2.70583000 -0.99463800

H 4.29977100 -1.05961900 0.42695000

H 4.42611800 -1.91203300 -1.11051600

H 0.58979500 -0.94628400 -1.07641200

H 2.76886000 -0.33847800 -2.16643900

H 2.33941700 2.93043500 -1.42244500

H 1.53276800 1.57535600 -2.20274000

H 0.91637400 2.48826900 0.67464500

H 0.24191200 3.25961600 -0.73566700

H -1.75888800 -1.41484400 1.46857100

H -4.00903600 2.04017700 0.39570100

H -3.37901800 1.15039000 -0.98771500

H -4.05592000 -0.42574300 0.83312700

H -3.03923400 0.44402800 1.96762500

**Conformation of 2*E*_1_2 (11.2%)**

C -1.54650200 -1.36625900 -2.59917000

C -2.73704300 0.31290100 2.37599500

C 1.97430200 3.22039300 -1.07086900

C 2.21324100 -1.17932200 2.12298900

C 3.11444600 -2.92458200 0.55338100

C 2.03624000 -1.84043400 0.74458800

C -1.52549600 -1.40699800 -1.09136400

C -2.77808700 -1.93821000 -0.41904000

C -3.90371200 -0.83918900 -0.28160300

C -0.56614800 -0.87360800 -0.31402000

C -3.29630100 0.52453200 -0.06840000

C -2.66367400 0.97842400 1.02441300

C -1.75124200 2.19717000 0.92412000

C -0.66556000 2.18137200 -0.19020000

C 1.97193000 -0.89416200 -0.49671100

C 0.64622900 -0.06155300 -0.68250300

C 0.62919400 1.34757100 0.04515000

C 1.90585800 2.08379200 -0.36213000

C 3.16748300 1.34577500 0.01591900

C 3.22271600 -0.01303500 -0.70882600

H 0.71287100 1.16023000 1.12572200

H 0.60573100 0.18162900 -1.75235500

H -2.40470900 -0.78560100 -2.96604900

H -1.65867500 -2.37816900 -3.00984500

H -0.64099500 -0.93102700 -3.02816400

H -3.05481700 1.03497700 3.14012400

H -1.75148500 -0.05582500 2.69353700

H -3.43619500 -0.52686000 2.39921200

H 1.10615300 3.76892100 -1.41821600

H 2.94025900 3.64291100 -1.33558700

H 3.18988100 -0.69185200 2.21817700

H 2.15574900 -1.93983000 2.91069200

H 1.44283400 -0.43230400 2.33498900

H 4.12827200 -2.50789000 0.57248500

H 2.98758400 -3.45225900 -0.39880400

H 3.05608200 -3.66824000 1.35636600

H 1.07595100 -2.37010400 0.76226100

H -3.18982400 -2.80139200 -0.95758500

H -2.51972900 -2.28221000 0.58797200

H -4.51448500 -0.82583800 -1.19087200

H -4.57199500 -1.13842100 0.53413900

H -0.71007100 -0.98421800 0.75997400

H -3.16531600 1.10287000 -0.98322300

H -2.36818700 3.09110800 0.75603000

H -1.25693100 2.36354900 1.89031900

H -1.12225000 1.89666700 -1.14494800

H -0.36812300 3.22647000 -0.30173400

H 1.94604300 -1.59725100 -1.34133200

H 4.04913000 1.93993600 -0.24859500

H 3.19591900 1.19646100 1.10210600

H 4.12789900 -0.55656000 -0.41463300

H 3.32618100 0.19263900 -1.78247000

**Conformation of 6_1 (37.5%)**

C 0.83384900 1.63253600 2.59760800

C 2.71151900 -1.00925000 -2.23276200

C -2.49486600 -1.62378200 2.20950200

C -2.30295800 2.85601300 -0.09565300

C -1.61672800 2.40038400 -2.43907400

C -1.84820900 1.93385100 -1.20346500

C 1.32844300 1.44789900 1.18060400

C 2.79682000 1.82550500 0.98871300

C 3.63342600 0.88294700 0.06109800

C 0.60442500 0.89519900 0.19130900

C 3.15966500 -0.53982500 0.20359600

C 2.62059900 -1.31255000 -0.75416700

C 1.72525200 -2.47603300 -0.38863700

C 0.20654100 -2.16646100 -0.57889700

C -1.67820100 0.45389000 -0.88617500

C -0.71973900 0.18423100 0.30923900

C -0.52626500 -1.36392400 0.54606200

C -1.91195600 -2.01470500 0.83976200

C -2.89215000 -1.73107200 -0.31897900

C -3.04158600 -0.23332600 -0.62887600

H -1.22659900 0.54897400 1.20887500

H 0.08004700 -1.45775800 1.45769700

H -0.24300000 1.48550600 2.70558700

H 1.33704600 0.93691200 3.28466800

H 1.06970900 2.64351800 2.95548000

H 3.01225400 -1.91166400 -2.78055900

H 1.74566400 -0.69861900 -2.65438600

H 3.44010400 -0.22930100 -2.46184500

H -2.75334800 -0.56278500 2.28141400

H -3.40959700 -2.19367900 2.41022200

H -1.78281300 -1.84496400 3.01311300

H -3.24768300 2.52260300 0.35042600

H -1.56141700 2.89561100 0.71078900

H -2.44848300 3.87396400 -0.46768300

H -1.28067200 1.74801800 -3.24139700

H -1.75341500 3.44963900 -2.68838100

H 3.26305900 1.81992400 1.98268500

H 2.89469000 2.85917300 0.62820100

H 4.69024600 0.99980800 0.33686000

H 3.54688100 1.21295600 -0.97794900

H 1.06043400 0.83953900 -0.79384800

H 3.05323600 -0.88232400 1.23447500

H 1.90121200 -2.77948800 0.65092000

H 1.96068700 -3.34586500 -1.01834000

H -0.29408000 -3.13907200 -0.66254900

H 0.06159700 -1.69140300 -1.55510200

H -1.24254900 -0.00735800 -1.78311900

H -1.75129300 -3.10145800 0.87787900

H -3.87524300 -2.16134100 -0.08712100

H -2.53822300 -2.24664500 -1.22184700

H -3.68601000 -0.09767600 -1.50578300

H -3.54973000 0.26608700 0.20577900

**Conformation of 6_2 (27.9%)**

C -1.61385900 -2.79722100 0.23716600

C -3.01394500 0.25021500 1.93921700

C 1.50984900 3.32041300 -0.35227900

C 2.63739200 -2.15032800 1.77694000

C 4.30187800 -0.94986600 0.40280700

C 3.02772700 -1.33792700 0.55921900

C -1.54273000 -1.64783300 -0.73852100

C -2.75884700 -1.44402900 -1.62257300

C -3.81670200 -0.48627800 -0.95188100

C -0.56840800 -0.72064600 -0.78046400

C -3.14162700 0.78368500 -0.51901300

C -2.65175200 1.04756100 0.70521000

C -1.61879100 2.13421800 0.91894000

C -0.24255300 1.63772700 1.46036800

C 1.92128200 -1.09989700 -0.46965900

C 0.60325500 -0.50830900 0.14865500

C 0.82778600 0.99637400 0.52783300

C 1.21144200 1.85403100 -0.70677300

C 2.44222800 1.24230900 -1.41138400

C 2.31996800 -0.26247900 -1.70128700

H 0.39898900 -1.04382200 1.08134800

H 1.73175800 0.98023100 1.15810400

H -1.60711300 -3.75508300 -0.30018000

H -0.78037900 -2.81062200 0.94377700

H -2.54726300 -2.77321100 0.81519900

H -2.23524100 -0.47662900 2.20177300

H -3.12680600 0.91752800 2.80288200

H -3.95033000 -0.29952800 1.82198900

H 2.29384200 3.38642500 0.41304800

H 1.86731900 3.86064600 -1.23664200

H 0.63057700 3.85195500 0.02286800

H 2.00022200 -1.57972900 2.46352900

H 2.07231800 -3.04697000 1.49127700

H 3.52113000 -2.46909200 2.33636800

H 4.64424500 -0.37544200 -0.45027900

H 5.05779300 -1.20888000 1.13969500

H -3.25082000 -2.39582800 -1.86003500

H -2.45395500 -0.99497300 -2.57507600

H -4.28314100 -1.00303000 -0.10722100

H -4.60931800 -0.29481500 -1.68605500

H -0.66985000 0.02504400 -1.56531300

H -2.82354800 1.43513900 -1.33509800

H -1.99828700 2.85017300 1.66363400

H -1.47806400 2.69861600 -0.00956500

H -0.41937700 0.94357500 2.29170700

H 0.23756600 2.50887900 1.92120000

H 1.65270300 -2.10529700 -0.82880400

H 0.37294800 1.85250200 -1.41768800

H 3.31808400 1.42892500 -0.77733600

H 2.62343600 1.77938900 -2.35182200

H 1.55417100 -0.41603100 -2.47142300

H 3.25482000 -0.64036800  **-2.13098600**

**Conformation of 6_3 (18.2%)**

C -1.24400600 -1.72164200 2.07714300

C -3.24993800 1.32176600 1.00904200

C 1.11846000 3.73814300 0.53170800

C 2.66330800 -2.62278700 0.76117800

C 2.92645000 -2.39811800 -1.69973600

C 2.60038500 -1.83473200 -0.52725900

C -1.16730100 -1.76444200 0.56966300

C -2.32055600 -2.46162800 -0.12769700

C -3.55198000 -1.50342900 -0.31320300

C -0.25109300 -1.12550300 -0.17950200

C -3.10920800 -0.22992900 -0.97935600

C -2.83906000 0.95029900 -0.39634200

C -2.00028400 1.98837200 -1.13176100

C -0.50015000 1.61665900 -1.30344400

C 2.17469700 -0.37508200 -0.45768400

C 0.80229900 -0.13346800 0.23743800

C 0.33502500 1.36414900 -0.00477300

C 1.54745800 2.34707600 0.04536200

C 2.70052800 1.77451300 0.87773900

C 3.26981300 0.49825400 0.22766800

H 0.96342500 -0.22898300 1.31720300

H -0.30235400 1.60715300 0.85512500

H -2.18205900 -1.26308200 2.41581900

H -0.42163200 -1.16612900 2.53393500

H -1.23098000 -2.73988000 2.48857000

H -3.88640800 0.57254300 1.48427700

H -2.37820200 1.48564500 1.65568800

H -3.80583800 2.26874600 0.99583000

H 0.78277900 3.69864300 1.57582600

H 1.94898500 4.45118000 0.47457200

H 0.29369900 4.14134200 -0.06671900

H 3.30513900 -2.13933000 1.50735300

H 1.66684200 -2.71804700 1.20706200

H 3.05261000 -3.62971700 0.58684300

H 2.87317800 -1.84287300 -2.63307000

H 3.25466000 -3.43240600 -1.76479400

H -2.64886900 -3.35017200 0.42756000

H -1.99873800 -2.80227100 -1.11878900

H -4.01131900 -1.30962800 0.66097500

H -4.30257000 -2.03023200 -0.91639700

H -0.32383400 -1.27850900 -1.25565000

H -2.78005000 -0.35605000 -2.01359700

H -2.06811100 2.95183700 -0.61022100

H -2.41145100 2.15508400 -2.13625200

H -0.03967100 2.44338300 -1.85969600

H -0.43571700 0.75051500 -1.96949200

H 2.06967600 -0.03355000 -1.49610700

H 1.92691100 2.45930400 -0.98227200

H 2.34478400 1.56429300 1.89625600

H 3.49646500 2.52205400 0.98497000

H 4.01868900 0.76684900 -0.52718600

H 3.80087600 -0.08853900 0.98527600

**Conformation of 6_4 (10.2%)**

C 1.06244900 -1.59522500 -2.39822300

C 3.13467200 0.53177000 2.04135900

C -1.72923400 3.50099200 -1.01425500

C -2.32531500 -2.94439100 -0.31243400

C -2.23876200 -2.51735400 2.13436200

C -2.18732900 -2.03244000 0.88507700

C 1.28344600 -1.53299900 -0.90572600

C 2.65677500 -1.96315600 -0.43213700

C 3.73456300 -0.83933700 -0.67934800

C 0.44047600 -0.97110500 -0.01985200

C 3.14098100 0.53362300 -0.47554300

C 2.76082300 1.08663800 0.68762800

C 1.81126400 2.26532200 0.70175700

C 0.31977400 1.89750300 0.97415700

C -2.00606400 -0.54216100 0.63633700

C -0.80888300 -0.17980600 -0.29043300

C -0.53925000 1.37681400 -0.22529700

C -1.88866800 2.17166800 -0.26389100

C -3.04118100 1.32839800 -0.82197100

C -3.31155900 0.10027800 0.06885300

H -1.12410000 -0.38298000 -1.31963000

H 0.00895900 1.61614200 -1.14748400

H 0.05188100 -1.30807300 -2.69752100

H 1.76355500 -0.93675500 -2.93017900

H 1.24771300 -2.61088000 -2.77124400

H 3.83189700 -0.30669200 1.97603000

H 3.60367600 1.31241100 2.65492700

H 2.25221700 0.18884400 2.59803100

H -1.52188400 3.32419600 -2.07726000

H -2.63987600 4.10744800 -0.94731600

H -0.90273300 4.09718300 -0.61065800

H -3.13501800 -2.62581700 -0.97980900

H -1.40307500 -2.94904900 -0.90423100

H -2.53223700 -3.97229500 -0.00186300

H -2.12892600 -1.87073200 3.00158400

H -2.38995400 -3.57581400 2.33054000

H 2.98775000 -2.88569700 -0.92702900

H 2.61586000 -2.16910300 0.64247100

H 4.11884600 -0.92040300 -1.70200100

H 4.58609900 -1.02966600 -0.01548400

H 0.74171300 -1.00581600 1.02639200

H 2.80790800 1.03387900 -1.38533300

H 1.86867400 2.80364700 -0.25246000

H 2.11983400 2.97531400 1.48223900

H -0.15258500 2.81780100 1.33764900

H 0.26053400 1.19820600 1.81754300

H -1.80306500 -0.08810000 1.61507100

H -2.15900500 2.40976700 0.77662100

H -2.80705200 1.01768000 -1.84965600

H -3.94992200 1.93923600 -0.89302100

H -3.95087700 0.38776600 0.91230400

H -3.88067300 -0.64183800 -0.50173300

**Conformation of 7_1 (31.9%)**

C 1.90588900 -1.53838400 2.05301100

C 3.71786800 1.67151400 0.57940100

C -2.20206800 3.24226700 -0.46396900

C -2.63025700 -1.29662800 -1.87061300

C -2.36552500 -3.25747900 -0.39624200

C -2.33394000 -1.92217100 -0.52418600

C 1.52731400 -1.60442700 0.59405700

C 2.56932300 -2.20441400 -0.33725400

C 3.64055600 -1.14902900 -0.79779100

C 0.41637900 -1.07298500 0.05633200

C 2.92213500 0.09783000 -1.23028500

C 2.80946300 1.25313900 -0.55397800

C 1.63280800 2.17513800 -0.80556500

C 0.53130500 2.02210600 0.29979800

C -2.02641600 -1.06025700 0.70187400

C -0.68308600 -0.24128700 0.66687100

C -0.74624200 1.17333600 -0.01913300

C -2.01076300 1.98184000 0.39836600

C -3.30448200 1.15228500 0.36129800

C -3.20047600 -0.15740300 1.14418600

H -0.80152100 1.02443700 -1.10751300

H -0.45566100 -0.04388300 1.72341000

H 2.85802800 -1.00908700 2.19190000

H 1.15277200 -1.03905600 2.66740400

H 2.05018300 -2.54932200 2.45700300

H 4.53123000 0.96533100 0.75797900

H 4.17151500 2.64474100 0.34767300

H 3.17104900 1.80246800 1.52182900

H -2.37090100 2.96743600 -1.51293300

H -3.07708500 3.81003600 -0.12724600

H -1.34275100 3.91825700 -0.43395300

H -1.86312300 -0.57432400 -2.16938600

H -2.69086000 -2.06521000 -2.64671500

H -3.58474000 -0.75823300 -1.86513500

H -2.16303100 -3.74647000 0.55284400

H -2.58935100 -3.90563700 -1.24000100

H 2.07036100 -2.59435700 -1.23183500

H 3.08683100 -3.05018600 0.13394900

H 4.22769300 -1.59500600 -1.61106500

H 4.33586600 -0.94823500 0.02272500

H 0.29128500 -1.22059600 -1.01665700

H 2.22622800 -0.05431500 -2.05691400

H 1.19840000 1.97550900 -1.79199700

H 1.96827000 3.22131100 -0.81553100

H 0.20730800 3.02839700 0.58058000

H 0.99427300 1.61259700 1.20462000

H -1.87351400 -1.78748800 1.50811000

H -1.85589800 2.30053200 1.44263200

H -3.57235100 0.95638200 -0.68342300

H -4.12365800 1.75798600 0.77124000

H -3.05502400 0.08528000 2.20695000

H -4.14053400 -0.71924800 1.08237900

**Conformation of 7_2 (31.2%)**

C -1.90385100 -1.58242600 -2.28363600

C -2.27562100 1.09315700 2.23789400

C 2.33603500 3.11365100 -0.01054800

C 2.23501100 -1.20606900 2.11736900

C 1.87258400 -3.33316100 0.92026000

C 1.99056500 -1.99836500 0.85104200

C -1.70725900 -1.47019500 -0.79207300

C -2.92778100 -1.74595000 0.06631500

C -3.93047700 -0.52316500 0.11870100

C -0.59643700 -1.01510800 -0.18825000

C -3.21765400 0.79190600 -0.07871000

C -2.39877100 1.42906900 0.77327600

C -1.45233200 2.49608700 0.22963000

C -0.41284600 1.96296000 -0.79555300

C 1.90612400 -1.30890800 -0.51167000

C 0.65239700 -0.39414700 -0.75880000

C 0.77375600 1.09277800 -0.26129300

C 2.12938800 1.73718600 -0.66592200

C 3.33520100 0.83836300 -0.34762600

C 3.20183200 -0.57268200 -0.92636700

H 0.73448300 1.08855200 0.83784900

H 0.56716000 -0.33012200 -1.85247100

H -1.00284000 -1.33970900 -2.85219400

H -2.20479300 -2.60323300 -2.55354400

H -2.70889100 -0.91964100 -2.63062200

H -1.26185100 0.75057400 2.48570600

H -2.45762800 1.98610100 2.85094000

H -2.97982600 0.31982700 2.55542200

H 2.37588300 3.01796800 1.08203500

H 3.28328100 3.55815400 -0.33702200

H 1.54035200 3.82435200 -0.25224600

H 1.52262400 -0.38239700 2.23425700

H 2.15235700 -1.85110300 2.99701100

H 3.23609500 -0.76021000 2.12768400

H 1.70310400 -3.93943100 0.03451300

H 1.93518300 -3.86236200 1.86796500

H -2.59485800 -1.95794400 1.08755700

H -3.47434200 -2.63178200 -0.28267300

H -4.46832300 -0.56810100 1.07281200

H -4.68725600 -0.64031000 -0.66450300

H -0.61561800 -1.01329500 0.90153300

H -3.21007500 1.14619000 -1.11082400

H -0.92832200 2.99366700 1.05568900

H -2.03726200 3.27773100 -0.27350800

H 0.00092200 2.83464900 -1.31578500

H -0.95415400 1.39756500 -1.56145000

H 1.77878200 -2.13478100 -1.22163200

H 2.10229100 1.87940000 -1.75922700

H 3.47108500 0.79901800 0.73970200

H 4.24399600 1.30924700 -0.74567000

H 3.20570700 -0.49621900 -2.02335800

H 4.07354100 -1.18223500 -0.65817500

**Conformation of 7_3 (20.0%)**

C 1.35897700 -2.25001800 1.29324500

C 3.94950000 0.48657500 1.08570500

C -1.05286500 3.86230100 -0.03002600

C -2.67710100 -2.42441600 1.57532600

C -3.35725700 -2.34243300 -0.80919800

C -2.84025100 -1.71009900 0.25337500

C 0.96423000 -1.86172100 -0.11049000

C 1.89711900 -2.34018400 -1.20891100

C 3.20396800 -1.46639100 -1.27104500

C -0.03837500 -1.02679500 -0.43298600

C 2.84694300 -0.00921500 -1.13895300

C 3.03792100 0.79620200 -0.07950900

C 2.20822500 2.05634100 0.09434500

C 0.91771000 1.76016500 0.92692200

C -2.40115100 -0.25355300 0.23449600

C -0.84890300 -0.09313400 0.43307500

C -0.35174500 1.37482000 0.10443600

C -1.49623500 2.41285500 0.23054600

C -2.65596100 2.06681100 -0.73962700

C -2.85312800 0.55668900 -0.98610800

H -0.06551600 1.37655000 -0.95747000

H -0.63706300 -0.28392200 1.49037200

H 2.39128700 -1.95450800 1.51601900

H 0.71440400 -1.80954000 2.05732600

H 1.31547500 -3.34153700 1.40801400

H 4.71383300 1.27044200 1.17429200

H 3.40275600 0.48018000 2.03776600

H 4.46930300 -0.46869300 0.98825100

H -0.58684400 3.95511500 -1.01920600

H -1.92108000 4.53154300 -0.01258200

H -0.34116600 4.23463000 0.71222100

H -3.18487100 -1.87961900 2.38236700

H -3.08817800 -3.43706700 1.53597600

H -1.62122600 -2.50317300 1.85900900

H -3.48638400 -1.85931000 -1.77198500

H -3.66347100 -3.38338300 -0.74681700

H 1.38439800 -2.26835600 -2.17488900

H 2.18438600 -3.39145000 -1.07340600

H 3.70819500 -1.67667200 -2.22334900

H 3.89042700 -1.77923700 -0.47896000

H -0.20438500 -0.87155600 -1.50051500

H 2.18553000 0.36196400 -1.92344000

H 1.92895900 2.47564600 -0.87908100

H 2.79828100 2.82483400 0.61077800

H 0.69031900 2.63311100 1.54809800

H 1.14796000 0.95755100 1.63597000

H -2.87145700 0.20983900 1.11632700

H -1.87622900 2.36473400 1.26279400

H -2.47549100 2.56825700 -1.69973800

H -3.58130800 2.50253300 -0.34124300

H -3.90525600 0.35246400 -1.21076900

H -2.28532200 0.23983700 -1.86984000

**Conformation of 7_4 (13.8%)**

C 1.27690200 -2.37952900 1.20355900

C 3.76638800 0.48387000 1.31612000

C -1.21366600 3.84963800 -0.21802400

C -2.78293600 -2.63064800 -0.75793500

C -3.08134800 -2.22741100 1.67436900

C -2.74174600 -1.74836200 0.46813700

C 1.00564800 -1.85488000 -0.18439400

C 2.00502700 -2.25817300 -1.25283500

C 3.30538000 -1.37682700 -1.17593200

C 0.05421600 -0.95889100 -0.50048600

C 2.93291500 0.07548000 -1.03761300

C 3.00129500 0.84441800 0.06309800

C 2.19441200 2.12561500 0.16875700

C 0.84475700 1.90949800 0.92443400

C -2.32673900 -0.28947100 0.31491800

C -0.77613300 -0.07160500 0.39713500

C -0.36401400 1.42436100 0.06193700

C -1.57501600 2.39317700 0.12135300

C -2.70071300 1.93457900 -0.84156200

C -2.88981800 0.40325500 -0.93590100

H -0.03858300 1.43441000 -0.98890700

H -0.50676500 -0.26835200 1.43985800

H 1.21497000 -3.47599000 1.20643200

H 2.28904200 -2.12372000 1.54151800

H 0.56466400 -2.01002100 1.94389900

H 4.51216400 1.26124300 1.53037000

H 3.10955700 0.43535800 2.19404200

H 4.29611100 -0.46763900 1.24078500

H -0.73323100 3.91187100 -1.20281500

H -2.12012100 4.46509900 -0.25566700

H -0.54128500 4.30852200 0.51212300

H -1.81293800 -2.65550000 -1.26481200

H -3.05834600 -3.65558900 -0.49298500

H -3.51727000 -2.26254200 -1.48505700

H -3.08084000 -1.59585000 2.55987100

H -3.36981300 -3.26571900 1.81660600

H 1.55515400 -2.12302100 -2.24353300

H 2.29210500 -3.31464000 -1.16892000

H 3.89153400 -1.55753800 -2.08648200

H 3.92140200 -1.71119200 -0.33621100

H -0.02624700 -0.70477500 -1.55936800

H 2.37211700 0.47747900 -1.88325100

H 1.99035700 2.53540900 -0.82753500

H 2.77586700 2.88523800 0.70893100

H 0.57895600 2.85180800 1.41405900

H 1.01384100 1.20222700 1.74430300

H -2.74629600 0.23080700 1.18542800

H -1.96773700 2.38169100 1.14941600

H -2.49001100 2.33708000 -1.84139200

H -3.63719000 2.40697000 -0.51912900

H -3.95297700 0.16544800 -1.05726000

H -2.38705500 0.01286800 -1.82912200

**Conformation of 8_1 (74.8%)**

C -3.01674800 -0.78372600 0.58878100

C -2.49734900 0.47198000 1.29728800

C -1.49098300 1.23871400 0.41842400

C -0.27657500 0.30110700 0.08730600

C -0.74121400 -1.06507600 -0.53504600

C -1.89975500 -1.78518200 0.23244500

C -1.47354100 -2.57921600 1.48038200

C -2.12743200 1.89249200 -0.84873000

C -1.15414100 2.86568800 -1.54011900

C -3.43272000 2.64558500 -0.53054700

C 0.39041600 -2.06642700 -0.88144300

C 1.46730300 -1.61286100 -1.91074400

C 2.59656300 -0.83905900 -1.26102000

C 2.66723300 0.49660900 -1.38703400

C 3.36370100 1.45591400 -0.46636300

C 0.66454500 0.18663200 1.27304000

C 1.77492600 0.91413100 1.50933900

C 2.32767500 1.98562100 0.58278500

C 3.53555700 -1.65765800 -0.40462900

C 2.59139100 0.68316200 2.76239400

H 0.28670600 0.78687200 -0.70633300

H -1.21030900 -0.80051100 -1.49290000

O -2.42424100 -2.72888900 -0.73515200

H -3.52219900 -0.50689800 -0.34375200

H -3.76629300 -1.29225500 1.21197300

H -2.00310300 0.19311100 2.23641600

H -3.33515900 1.11400200 1.58609600

H -1.09347400 2.06880100 1.02009500

H -1.10706900 -1.93417400 2.28280100

H -2.34089300 -3.12275600 1.87671000

H -0.70053600 -3.31359800 1.24465500

H -2.37254400 1.10376900 -1.57260500

H -0.87850100 3.68550100 -0.86420500

H -0.23149300 2.38106800 -1.87139700

H -1.62040400 3.31085400 -2.42604000

H -3.27318300 3.39469700 0.25606100

H -3.79407000 3.17377100 -1.41986300

H -4.23491600 1.97934700 -0.20089700

H -0.10345800 -2.95714700 -1.28110500

H 0.89955200 -2.38228400 0.03505500

H 0.99666400 -1.01209200 -2.69934800

H 1.86264900 -2.51608400 -2.39612300

H 1.97225400 0.96795400 -2.08415100

H 3.76290100 2.31932000 -1.01345700

H 4.20626500 0.99100700 0.05522300

H 0.40427700 -0.54709700 2.03394600

H 1.51868600 2.48569300 0.04059700

H 2.81737800 2.76005500 1.18776100

H 3.94850000 -2.48579900 -0.99580600

H 4.37516000 -1.07387500 -0.02222700

H 3.02496800 -2.10752600 0.45502600

H 3.63610000 0.44661900 2.51913300

H 2.61479300 1.58463400 3.38954500

H 2.19277500 -0.13855400 3.36488100

H -3.15121400 -3.21588700 -0.32359800

**Conformation of 8_2 (25.2%)**

C -3.07197100 -0.54911000 0.40822300

C -2.50699400 0.59124800 1.26172200

C -1.33074400 1.30318100 0.56493300

C -0.19549800 0.26035000 0.25981200

C -0.74515100 -0.98843700 -0.52702800

C -2.03054100 -1.63627900 0.07906400

C -1.79504200 -2.55660400 1.28936800

C -1.73618000 2.14301300 -0.68767500

C -0.61836400 3.12653500 -1.08276200

C -3.03867200 2.93921200 -0.48666800

C 0.31062500 -2.06874700 -0.87779900

C 1.30738100 -1.72587400 -2.01215200

C 2.31424000 -0.63010600 -1.67657300

C 3.02299800 -0.70784400 -0.53955900

C 3.74622300 0.40503200 0.17160400

C 0.58112800 -0.04951500 1.52665300

C 1.80890100 0.38259600 1.87722200

C 2.71436900 1.23513900 1.00188000

C 2.38367000 0.52416200 -2.64599900

C 2.40584100 -0.00504600 3.21240300

H 0.50210600 0.73692300 -0.42856200

H -1.10555100 -0.59062500 -1.48581900

O -2.55047200 -2.45055300 -1.00233200

H -3.44615200 -0.15552000 -0.54434400

H -3.92995300 -1.01536200 0.91376200

H -2.16019600 0.19712400 2.22514600

H -3.30073000 1.30325800 1.50805200

H -0.91403200 2.02065800 1.28626700

H -1.47819300 -2.00728300 2.17887500

H -2.73211600 -3.06817500 1.54463000

H -1.04568600 -3.32052200 1.07029100

H -1.89652700 1.46261300 -1.53521000

H -0.46110100 3.86894800 -0.28984900

H 0.33849100 2.63032100 -1.26593200

H -0.88403400 3.66928100 -1.99684900

H -2.97340300 3.58518700 0.39853100

H -3.22493800 3.58551500 -1.35170700

H -3.91431900 2.29471600 -0.36814100

H -0.23817000 -2.96088600 -1.19539700

H 0.87205600 -2.34576500 0.02124600

H 0.73947700 -1.43877400 -2.90673500

H 1.83811300 -2.64962200 -2.28241100

H 2.89575100 -1.60768800 0.05981300

H 4.26331800 1.08385300 -0.51572200

H 4.51234300 -0.00778600 0.83850700

H 0.06727200 -0.66108900 2.26616600

H 2.11882000 1.82777200 0.30175000

H 3.25822000 1.95100400 1.63336200

H 1.40885500 1.02011500 -2.74795500

H 3.12198700 1.27883400 -2.36399100

H 2.64502200 0.15870100 -3.64831600

H 3.36383000 -0.52719300 3.08467000

H 2.61577300 0.88395200 3.82249400

H 1.74049400 -0.65956200 3.78336400

H -3.35036800 -2.89593700 -0.69134100
