# Supplementary material for: Discovery of a terpene synthase synthesizing a nearly non-flexible eunicellane reveals the basis of flexibility
Source: Nat Commun. 2024 Jul 15;15:5940. doi: 10.1038/s41467-024-50209-z (PMC11250809; doi:10.1038/s41467-024-50209-z)
Supplement: Supplementary file 5 — Supplementary Data 2 [file 41467_2024_50209_MOESM5_ESM.docx]

**Supplementary data 2. Cartesian coordinates for the optimized structures of intermediates and transition.** This document provides supplementary data corresponding to Figure 4b in the main text and Supplementary Fig. 25.

**Structure A^+^**

C 1.11670600 -2.94198100 -0.57843600

C -0.11489100 -2.94227900 0.38395700

C -1.41660800 -2.59177400 -0.28511000

C -1.03886000 -0.05720400 -0.99868500

C 2.51126200 -0.91910500 -0.96038000

C 2.22175600 -1.97773300 -0.18171300

C 2.97293300 -2.35546300 1.07350200

C -2.52437900 -2.01693000 0.27151500

C -3.81329300 -1.93329700 -0.49850700

C -2.60020500 -1.53290900 1.69227800

C 3.59088600 0.11628800 -0.78831400

C 3.08265300 1.57033700 -0.95771500

C 2.08889700 2.04316600 0.08480300

C 0.89743000 2.55820100 -0.29575500

C -0.14890700 3.16357400 0.59263800

C -2.06344900 0.88039100 -1.09049800

C -2.24685300 1.93760700 -0.20175600

C -1.35590400 2.20390300 0.96189300

C 2.55466900 1.95002000 1.51600400

C -3.32339000 2.93918000 -0.46576000

H 1.53373900 -3.95594200 -0.61529200

H 0.78960200 -2.71747400 -1.59997100

H -0.24096200 -3.94748700 0.81183100

H 0.07073400 -2.28600500 1.23989000

H -1.52833600 -2.97186400 -1.30158700

H -0.79432600 -0.66194500 -1.86265800

H -0.31081600 -0.05285300 -0.19849800

H 1.92258300 -0.79648000 -1.87286600

H 3.52715300 -3.28938400 0.91258300

H 3.69229300 -1.59893000 1.39083300

H 2.29158800 -2.54242100 1.91266000

H -3.71029900 -2.28115200 -1.52945400

H -4.58142800 -2.54465900 -0.00833500

H -4.20096300 -0.90579600 -0.50757900

H -1.63751500 -1.51966200 2.20555700

H -3.03843900 -0.52714700 1.73177700

H -3.27580100 -2.17991200 2.26654800

H 4.10327000 0.00689900 0.17114100

H 4.35910500 -0.04594900 -1.55717300

H 2.64880800 1.68762900 -1.95770200

H 3.95691600 2.23561800 -0.91892300

H 0.68755300 2.58763200 -1.36435000

H 0.27491400 3.47442700 1.55035200

H -0.55826200 4.06672300 0.12661000

H -2.72119600 0.83041200 -1.95445800

H -0.94100900 1.28632100 1.38316100

H -1.93500400 2.70484100 1.74448200

H 2.66059800 0.90233200 1.82186900

H 3.54591400 2.40677000 1.62468500

H 1.88395300 2.43483200 2.22723100

H -3.83807100 2.77452400 -1.41375200

H -4.06222400 2.90373700 0.34720500

H -2.91679300 3.95841700 -0.44958100 **Structure TS^A_B^**

C 1.10702200 -2.89178900 -0.81324900

C -0.14331400 -2.99315800 0.11285200

C -1.41622600 -2.34756900 -0.39152800

C -0.93237000 -0.27721500 -0.74133600

C 2.49571700 -0.82644700 -1.00570200

C 2.18713900 -1.93991000 -0.31718700

C 2.91223000 -2.40441200 0.92549500

C -2.47042500 -1.92682800 0.40361700

C -3.80877200 -1.64461300 -0.19114400

C -2.36433700 -1.74228700 1.88721500

C 3.55413400 0.20395500 -0.71393100

C 3.03883400 1.66261100 -0.80854300

C 2.01135400 2.07711100 0.22929800

C 0.81496500 2.56269900 -0.16260800

C -0.28458600 3.12666600 0.69662100

C -1.92857500 0.68178700 -1.09861300

C -2.21032500 1.81638200 -0.38094400

C -1.52109200 2.17982700 0.90388400

C 2.45802900 1.97077600 1.66693600

C -3.16686300 2.83233100 -0.93334300

H 1.54591300 -3.89388700 -0.89294100

H 0.80426600 -2.62297800 -1.83255000

H -0.40344700 -4.05319900 0.24272200

H 0.10286600 -2.62960500 1.11372400

H -1.67233600 -2.58714400 -1.42375100

H -0.38410200 -0.72439100 -1.56110800

H -0.33964000 -0.13759900 0.15327000

H 1.94036000 -0.63727200 -1.92761600

H 3.49409100 -3.30896000 0.70484900

H 3.60373300 -1.65971100 1.32166300

H 2.21885200 -2.67403900 1.73158400

H -3.88123700 -1.93310900 -1.24151100

H -4.58629300 -2.16885500 0.37723900

H -4.03502600 -0.57090300 -0.10304900

H -1.34026900 -1.63591700 2.24679500

H -2.95772700 -0.88471300 2.21985500

H -2.79383000 -2.62631200 2.37991900

H 4.02374400 0.03304200 0.25833400

H 4.35767800 0.09866800 -1.45640600

H 2.63256700 1.83421500 -1.81239900

H 3.90904400 2.32682500 -0.71026900

H 0.62703700 2.59972700 -1.23563200

H 0.07848900 3.38036400 1.69589800

H -0.64077400 4.06430900 0.25324100

H -2.41810000 0.55231300 -2.06135500

H -1.18385200 1.29176100 1.44453000

H -2.23275500 2.70467200 1.55129600

H 2.63293500 0.92508200 1.94768200

H 3.41156100 2.49386900 1.81072700

H 1.73994900 2.38646000 2.37607800

H -3.57766000 2.54053600 -1.90202600

H -3.99594700 2.99033200 -0.23161000

H -2.67532000 3.80691400 -1.04391700 **Structure B^+^**

C -1.41345200 -2.76034200 0.25126200

C -2.29141300 -1.60733600 0.78348400

C -1.98798100 -0.19943400 0.01419000

C -1.37918700 0.86942700 0.93985400

C 0.85966300 -2.52768100 -0.69077500

C 0.08420800 -2.60227200 0.40863300

C 0.59977400 -2.64185000 1.82838500

C -3.34411800 -0.04210200 -0.46331200

C -3.71413400 -0.59198100 -1.78864800

C -4.39311700 0.65677400 0.31487300

C 2.35902500 -2.51544300 -0.79918300

C 2.94408400 -1.22348400 -1.43856500

C 3.11511300 -0.05007400 -0.49143500

C 2.36050900 1.05682500 -0.62519500

C 2.45088800 2.33813100 0.16150700

C -1.16372700 2.17225300 0.20935800

C -0.06587900 2.95132500 0.21534500

C 1.18939300 2.65796900 1.00986800

C 4.22937600 -0.20957100 0.51789300

C -0.05316400 4.24277100 -0.56883000

H -1.74545800 -3.65340400 0.80134800

H -1.66272600 -2.94401700 -0.80015100

H -3.33949900 -1.92928300 0.71000900

H -2.13371700 -1.37925400 1.84149500

H -1.29637200 -0.43592900 -0.79990600

H -0.43696600 0.46473300 1.31466300

H -2.02785900 1.01131000 1.81580400

H 0.35006900 -2.53393500 -1.65739400

H 0.14055700 -1.86379300 2.45106000

H 0.36060200 -3.60311600 2.30156600

H 1.67940200 -2.50178200 1.88525900

H -3.05781100 -1.40543200 -2.10655800

H -4.76548100 -0.87595000 -1.86824100

H -3.54149700 0.23290500 -2.50259900

H -5.34880700 0.12527400 0.27583700

H -4.11069900 0.87757700 1.34322000

H -4.56620400 1.61916600 -0.19592100

H 2.83210900 -2.70327000 0.16801400

H 2.64040400 -3.36082300 -1.44154500

H 2.31917500 -0.92974900 -2.29043000

H 3.92952600 -1.47747700 -1.85124400

H 1.61240900 1.05604300 -1.41970400

H 3.30707800 2.32125800 0.84030600

H 2.63615300 3.16982400 -0.53104900

H -2.01961700 2.52824000 -0.36847700

H 1.02978500 1.84017700 1.71823400

H 1.41064900 3.54858400 1.61382400

H 5.19988000 -0.25821500 0.00699200

H 4.27710600 0.60274700 1.24437300

H 4.13050700 -1.14581300 1.07970400

H -0.99698300 4.41308000 -1.09378000

H 0.12732500 5.09651300 0.09573500

H 0.75179300 4.25038900 -1.31270200 **Structure TS^B_C^**

C -1.16994600 -2.41520800 -0.88754900

C -2.21734300 -1.88262100 0.14176800

C -2.40444900 -0.37687900 0.18788800

C -1.14737800 0.45778100 0.43376500

C 1.28813800 -2.04312600 -0.89144100

C 0.22944400 -2.67431200 -0.35176700

C 0.32788000 -3.75005100 0.70493100

C -3.70235400 0.20446700 0.25863800

C -4.96784600 -0.60742600 0.13677900

C -3.90748200 1.66089900 0.55906600

C 2.75037400 -2.23742000 -0.58205500

C 3.58879100 -0.94784700 -0.75502800

C 3.26322800 0.17683300 0.21080100

C 2.73090300 1.32471200 -0.24785500

C 2.38520000 2.57281600 0.52477100

C -0.92266100 1.61684700 -0.52684400

C -0.03463300 2.61296300 -0.36152400

C 0.86939600 2.72711900 0.84655200

C 3.61996100 -0.08201600 1.65538000

C 0.11291900 3.68757900 -1.41098100

H -1.57233200 -3.36250100 -1.26966200

H -1.11945900 -1.74053200 -1.75015300

H -3.17201800 -2.38679200 -0.00715100

H -1.90210200 -2.14614000 1.16077800

H -2.93541400 0.02874000 -0.89683200

H -0.29695600 -0.22815700 0.39564100

H -1.20896600 0.81438400 1.47104300

H 1.08807000 -1.32357200 -1.68781600

H -0.32868500 -3.55687900 1.56326700

H 0.02125600 -4.72031100 0.29203100

H 1.34061000 -3.86529000 1.09272000

H -4.88570900 -1.46141800 -0.53566600

H -5.21023000 -0.99753900 1.13430300

H -5.79978800 0.02051000 -0.18616900

H -4.37207700 1.69675800 1.55495800

H -2.99164000 2.24767200 0.57670700

H -4.62541000 2.10983500 -0.13320300

H 2.89351200 -2.64889700 0.42192500

H 3.15741400 -2.98995100 -1.27333400

H 3.47271600 -0.58903100 -1.78460500

H 4.64664800 -1.21986400 -0.63896200

H 2.53362600 1.38127800 -1.31894900

H 2.92687600 2.61839900 1.47394000

H 2.71488400 3.44577200 -0.05161000

H -1.50257700 1.59946300 -1.45173100

H 0.60375600 1.98079000 1.60124700

H 0.72025700 3.71361800 1.30743400

H 4.68687600 -0.32063400 1.75048700

H 3.40832000 0.76650100 2.30877400

H 3.07270900 -0.94700200 2.05086400

H -0.59970500 3.56190500 -2.23054800

H -0.03892400 4.68119600 -0.97174600

H 1.12162300 3.68704200 -1.83975200 **Structure C^+^**

C -1.51849600 -2.58486900 0.15654700

C -2.27003000 -1.43941500 1.00198400

C -2.21430700 -0.15554900 0.28968800

C -0.99594600 0.64117100 0.42809300

C 0.73793500 -2.34907200 -0.81930400

C -0.01943900 -2.59836700 0.27521700

C 0.52776100 -3.00398900 1.62257200

C -3.33714100 0.28180000 -0.57991800

C -4.22639000 -0.80335900 -1.20374700

C -4.18383000 1.22685700 0.35229100

C 2.22226300 -2.49788800 -0.98805500

C 2.94943000 -1.21061800 -1.46880800

C 3.11996300 -0.12989900 -0.41878800

C 2.50471300 1.05909500 -0.56851400

C 2.59473900 2.27383900 0.31694300

C -0.87758900 2.02664300 -0.13502100

C 0.10900200 2.90034000 0.15291500

C 1.27218700 2.60168000 1.07155500

C 4.05881300 -0.47193000 0.71433000

C 0.10652900 4.27352500 -0.47090200

H -1.93109700 -3.51487100 0.56312900

H -1.83162200 -2.52575000 -0.88787700

H -3.30235700 -1.76837900 1.12927000

H -1.78840700 -1.38127100 1.98091700

H -2.93683300 0.91310500 -1.37964100

H -0.27226100 -0.06696100 -0.08645200

H -0.62155600 0.56812600 1.45653100

H 0.21003800 -2.08272400 -1.73800000

H 0.14017000 -2.36484400 2.42625600

H 0.22664300 -4.02977600 1.86898100

H 1.61542400 -2.95530000 1.66904600

H -3.66103300 -1.46867300 -1.86164400

H -4.74337300 -1.41018400 -0.45493400

H -4.99481700 -0.31829700 -1.81180400

H -4.64984700 0.65798700 1.16129600

H -3.58984500 2.03757400 0.78038200

H -4.97502500 1.66614600 -0.26186800

H 2.69394900 -2.88211700 -0.08078600

H 2.37904700 -3.26552500 -1.75876400

H 2.42156200 -0.80515100 -2.33995900

H 3.94320400 -1.51606000 -1.82207600

H 1.88350200 1.18647300 -1.45608800

H 3.38253600 2.16518100 1.06645400

H 2.87576900 3.14016000 -0.29553800

H -1.63732900 2.34815900 -0.84075400

H 1.04775800 1.76978100 1.74680200

H 1.45239300 3.47975400 1.70411800

H 5.05607300 -0.70924200 0.32253600

H 4.17084000 0.33446400 1.44023000

H 3.72328400 -1.36053200 1.26239800

H -0.73056800 4.41426200 -1.15926100

H 0.04746400 5.04573300 0.30595100

H 1.03530000 4.45876500 -1.02332000

**Structure TS^C_D^**

C -1.00043500 -3.00088700 0.55190800

C -1.79905400 -1.87339800 1.28316100

C -2.00781800 -0.64395100 0.43969400

C -1.24826200 0.51883400 0.72765200

C 0.98693900 -2.29583500 -0.76358400

C 0.47965300 -2.70319500 0.41852300

C 1.29620900 -2.96028400 1.66365600

C -3.05897500 -0.74794400 -0.65529900

C -4.36715600 -0.08269200 -0.15512600

C -2.63980800 -0.24430800 -2.05044900

C 2.42654000 -2.06721500 -1.14024800

C 2.74862000 -0.63371600 -1.64174500

C 2.81978900 0.44230700 -0.57445600

C 1.97972900 1.49540400 -0.61772100

C 1.96024300 2.70412300 0.28011700

C -1.42227900 1.86854400 0.20413400

C -0.58522700 2.90590000 0.47106400

C 0.71609100 2.78945000 1.22111000

C 3.92731700 0.27546800 0.43899900

C -0.92936700 4.28126600 -0.02916400

H -1.14128300 -3.91661400 1.13692600

H -1.44710100 -3.18521500 -0.43054600

H -2.79912700 -2.25522600 1.52194800

H -1.31242300 -1.62309900 2.22977900

H -0.70661800 -0.30801400 -0.07024500

H -0.58399700 0.41725300 1.58403700

H 0.28831500 -2.19893800 -1.59874100

H 2.32153000 -2.59796000 1.58600800

H 0.84558700 -2.49034200 2.54661900

H 1.33723200 -4.03658400 1.87452200

H -3.26938800 -1.81796300 -0.75918900

H -5.16222900 -0.28055100 -0.87941700

H -4.68796100 -0.48823000 0.80924100

H -4.26535000 1.00079500 -0.05236600

H -3.42479200 -0.50396100 -2.76581000

H -1.71541700 -0.72273200 -2.38979800

H -2.50062600 0.83719300 -2.09536600

H 3.09955200 -2.34055700 -0.32403600

H 2.65911700 -2.75738600 -1.96242800

H 2.01895100 -0.34805600 -2.40891900

H 3.72388200 -0.67766100 -2.14544200

H 1.25097100 1.51341200 -1.42917000

H 2.84513300 2.74114700 0.91960800

H 1.99378300 3.61041000 -0.33783500

H -2.30415800 2.06463200 -0.39312600

H 0.73013700 1.91956000 1.88416400

H 0.83208400 3.67614700 1.85506600

H 4.89690900 0.19759900 -0.06862900

H 3.99086000 1.09891400 1.15154600

H 3.80641900 -0.64785200 1.01720400

H -1.83219100 4.29129600 -0.64361200

H -1.08011700 4.95928800 0.82029200

H -0.10417200 4.70208200 -0.61563800 **Structure D^+^**

C -0.81736800 -2.77521700 -0.85778900

C -2.06085800 -2.21355600 -0.13744500

C -2.40598900 -0.74240500 -0.45865600

C -1.40468700 0.25332000 -0.03885700

C 1.55094500 -2.03022900 -0.98461300

C 0.53714000 -2.50505500 -0.22776600

C 0.69629100 -2.97811400 1.19922500

C -3.81656100 -0.36019300 0.22058800

C -3.75989100 -0.21253100 1.74789600

C -4.47321200 0.85834500 -0.43519500

C 3.01456000 -1.95122500 -0.64932200

C 3.67439800 -0.56515600 -0.89295500

C 3.29791200 0.49244700 0.12217000

C 2.46404400 1.48784600 -0.23303700

C 1.96698000 2.62359000 0.60806700

C -1.18966000 1.44025800 -0.72554100

C -0.42262700 2.52168500 -0.27604100

C 0.42838100 2.50530400 0.94122000

C 3.93160000 0.34382200 1.48347700

C -0.41431900 3.76898200 -1.09704600

H -0.94148400 -3.86762700 -0.88819400

H -0.81659800 -2.44527600 -1.90362800

H -2.93170400 -2.80415500 -0.44157300

H -1.96775900 -2.35165600 0.94452000

H -2.57517000 -0.64186900 -1.53846600

H -0.91112500 0.09473800 0.91727100

H 1.31316000 -1.75623700 -2.01463500

H 1.71420700 -2.86897900 1.57389800

H 0.03748200 -2.43365500 1.88705900

H 0.42149200 -4.03736200 1.28526100

H -4.43048000 -1.23761900 -0.01630100

H -4.77713300 -0.14038200 2.14455800

H -3.28315600 -1.06603900 2.23838500

H -3.23280500 0.69962200 2.05143800

H -5.49261700 0.97265000 -0.05272200

H -4.54129800 0.75075200 -1.52287900

H -3.94104900 1.78928400 -0.21056300

H 3.21483900 -2.28417500 0.37199700

H 3.53323100 -2.66561000 -1.30477400

H 3.41999600 -0.22295400 -1.90253200

H 4.76228800 -0.70757400 -0.87333900

H 2.09618000 1.47775300 -1.26053400

H 2.48213800 2.68107700 1.56897900

H 2.13787200 3.58173500 0.10341200

H -1.71992400 1.57091200 -1.66631300

H 0.27553100 1.61014900 1.54475900

H 0.16442100 3.37903000 1.55146900

H 5.02078300 0.44989200 1.40371600

H 3.58001300 1.07403800 2.21446300

H 3.74815300 -0.65412500 1.89758100

H -1.24718600 3.81297900 -1.80106000

H -0.41146500 4.66466000 -0.46822700

H 0.51668900 3.80450500 -1.68330200 **Structure TS^D_E^**

C 1.80178100 2.08889900 1.22182200

C 2.08641800 0.57277300 1.22355400

C 2.15512000 0.09399100 -0.24273900

C 0.80845600 0.14971100 -0.97496800

C -0.30950100 1.67384600 -0.02712000

C 0.78394400 2.50132200 0.19293500

C 1.06844400 3.75803000 -0.56003900

C 2.94730200 -1.24295100 -0.47878900

C 4.45625500 -0.93491500 -0.51945600

C 2.66283400 -2.36356800 0.52961600

C -1.54771300 2.04576900 -0.82369200

C -2.84856600 1.87779300 0.02387400

C -3.10812000 0.40859100 0.28290100

C -2.54096400 -0.17962300 1.35182000

C -2.28360200 -1.64464300 1.56971900

C -0.12787500 -0.95287000 -1.20288200

C -0.72512900 -1.92536600 -0.44689800

C -0.84465000 -2.00323500 1.05716200

C -3.90305300 -0.30461300 -0.78268900

C -1.38987500 -3.06430200 -1.17845100

H -0.49812700 0.95874900 0.76888900

H 0.91082600 0.70338600 -1.90644500

H 2.73220300 2.64225200 1.05674600

H 1.42938600 2.40452800 2.20883600

H 1.33096800 0.02119400 1.78916900

H 3.03884500 0.39573000 1.73009700

H 2.75406600 0.85869300 -0.75531100

H 2.01345500 3.65737000 -1.11160400

H 0.28516900 4.05342700 -1.25639300

H 1.23502600 4.57618100 0.15381200

H 2.66548600 -1.60921400 -1.47457600

H 5.02259400 -1.84633800 -0.73320900

H 4.70077200 -0.20338200 -1.29694400

H 4.81751900 -0.54565800 0.43959300

H 3.26444900 -3.24253400 0.27859000

H 1.61815900 -2.67700600 0.52612400

H 2.92926800 -2.07051000 1.55103300

H -1.63317600 1.42191800 -1.72092900

H -1.48345900 3.07876200 -1.17517600

H -2.74095100 2.43153400 0.96342700

H -3.67617600 2.33701500 -0.52736800

H -2.07352900 0.47096000 2.09299500

H -3.02526000 -2.27826400 1.07733500

H -2.31862000 -1.89273000 2.63566200

H -0.36350200 -1.02511300 -2.26478000

H -0.12652700 -1.34794900 1.55394200

H -0.61621600 -3.02930800 1.37216400

H -4.91126900 0.12500500 -0.83328700

H -3.46165100 -0.17614500 -1.77898200

H -4.01321000 -1.37384200 -0.59677800

H -1.43872700 -2.90387500 -2.25699300

H -0.81660100 -3.98370000 -0.99773300

H -2.40023300 -3.25897300 -0.80598500 **Structure E^+^**

C 1.89063200 2.52229200 0.60573900

C 2.62482900 1.16656700 0.57413700

C 1.99148700 0.23695500 -0.46815500

C 0.49290500 -0.04286800 -0.14770400

C -0.29620100 1.28959200 0.12243100

C 0.42443600 2.39797500 0.72142400

C -0.31509400 3.43167400 1.47559800

C 2.81862000 -1.05148200 -0.76244200

C 3.93939000 -0.75806700 -1.77587600

C 3.39905500 -1.73910300 0.48571600

C -0.94877100 1.91508800 -1.22453200

C -2.48351000 1.69952000 -1.28034300

C -2.95357500 0.35880000 -0.72903100

C -2.48072900 -0.77864600 -1.26939700

C -2.55661400 -2.18296200 -0.74624600

C 0.18513400 -0.99019800 0.98629900

C -0.55050300 -2.12064500 0.91711000

C -1.13495100 -2.70779600 -0.35852500

C -3.90598800 0.43019600 0.44021500

C -0.83772300 -2.91851300 2.16662500

H 0.06759200 -0.46954200 -1.05523300

H 2.05073000 3.03210300 -0.36545800

H 2.28001000 3.21573600 1.35960100

H 2.59794900 0.72086300 1.57447700

H 3.67685300 1.35657000 0.34428300

H 1.97988000 0.80124500 -1.41513100

H -1.16633700 1.06591600 0.75322200

H 0.12098700 4.42813100 1.34930500

H -0.18145800 3.18114800 2.54329600

H -1.38783800 3.43823700 1.27368600

H 2.12724600 -1.76056400 -1.23869200

H 4.49294500 -1.67156700 -2.01394700

H 3.54206500 -0.35677200 -2.71489900

H 4.66442300 -0.03640000 -1.38018300

H 3.87305400 -2.68360800 0.20089300

H 2.63365200 -1.96808100 1.23135600

H 4.17178700 -1.12469900 0.96190700

H -0.42978500 1.41026400 -2.04361300

H -0.73581500 2.98361900 -1.34144800

H -2.77517900 1.82239600 -2.33235600

H -2.97885200 2.51178000 -0.73688600

H -1.86893400 -0.68141100 -2.16943900

H -3.22904000 -2.26697300 0.11182900

H -2.94846400 -2.84998200 -1.52435100

H 0.54219700 -0.68896000 1.97296900

H -0.46347000 -2.54717000 -1.20940500

H -1.20566400 -3.79349900 -0.23099500

H -4.82118600 0.95792600 0.14210500

H -4.19969300 -0.55196500 0.81249400

H -3.48558000 0.99779400 1.28193400

H -0.46334600 -2.42800100 3.06901800

H -1.91631000 -3.08045800 2.28849500

H -0.38139800 -3.91435200 2.10292700 **Structure TS^A_F^**

C -2.10268100 1.26179500 -0.07254400

C -3.27819700 0.56732900 -0.80684000

C -4.45798000 0.38395700 0.11174400

C 0.51698900 -0.48784900 -1.33467500

C 0.39881300 1.49650300 -0.31424000

C -0.86746900 1.55202700 -0.88016800

C -1.03681400 1.96945000 -2.30885400

C -5.05911100 -0.76221000 0.47837700

C -6.25596300 -0.73927300 1.39913000

C -4.65037900 -2.14281600 0.02520800

C 0.66505900 1.46495300 1.17868700

C 2.07756900 1.99188400 1.55212000

C 3.13620200 0.94989500 1.25912800

C 3.70706400 0.89939600 0.04312900

C 4.48869500 -0.23918700 -0.54807100

C 1.16971000 -1.44003500 -0.50008000

C 2.48839000 -1.82645500 -0.58720900

C 3.54425900 -1.14525900 -1.41450400

C 3.38871400 -0.02880100 2.37990000

C 2.93209500 -3.05232600 0.15514200

H 1.20986800 1.88960600 -0.92274600

H -1.84712200 0.69478200 0.82725300

H -2.45780700 2.24166600 0.29069700

H -3.58285900 1.18043400 -1.66465200

H -2.94366700 -0.39316100 -1.21291500

H -4.86751200 1.31448300 0.50760600

H 1.04498200 -0.04667100 -2.17164300

H -0.53268200 -0.67757600 -1.52215100

H -0.08991300 2.24115700 -2.77996800

H -1.52404400 1.18834100 -2.90579000

H -1.70454200 2.83917200 -2.35483600

H -6.52046700 0.27640700 1.70453400

H -7.13028800 -1.18751800 0.91055000

H -6.06524400 -1.33157700 2.30305100

H -3.76794800 -2.15490500 -0.61852600

H -4.44754700 -2.78452900 0.89178200

H -5.46999200 -2.61957700 -0.52688000

H -0.08903000 2.09070600 1.67234900

H 0.52825100 0.45560700 1.58664300

H 2.27306100 2.91885600 1.00089700

H 2.07518800 2.25171100 2.61619700

H 3.48861700 1.70984400 -0.65397000

H 4.98165100 -0.84552700 0.21605100

H 5.27661900 0.12843100 -1.21367400

H 0.52839700 -2.01311300 0.16574300

H 4.15567000 -1.91171000 -1.90573000

H 3.10566700 -0.52777600 -2.20319700

H 4.10766300 -0.80868000 2.12568300

H 3.78176300 0.50754600 3.25252100

H 2.46215900 -0.51613800 2.70961300

H 3.89043300 -2.88837200 0.65941100

H 2.19690600 -3.39357800 0.88657900

H 3.10398000 -3.86619300 -0.56275800

**Structure F^+^**

C 2.30978600 -2.07535000 -0.00714400

C 3.38364400 -1.27570400 -0.91208400

C 4.40184000 -0.51191900 -0.13545100

C -0.39055800 0.59513200 -0.39853900

C 0.06730800 -0.90052700 -0.23004100

C 1.33382400 -1.10647600 0.49654000

C 1.70430500 -0.32076500 1.67681100

C 4.69817800 0.80656000 -0.22753100

C 5.84443600 1.38525300 0.56253800

C 3.99153300 1.78826000 -1.12674400

C -0.89750400 -1.90258400 0.54791300

C -2.13757000 -2.36100100 -0.28097700

C -3.23979600 -1.32893000 -0.27208100

C -3.37219700 -0.48325000 -1.30836400

C -4.11213300 0.82118800 -1.31556500

C -1.30775200 1.22674400 0.62029300

C -2.46401500 1.87332000 0.36877600

C -3.11214100 1.98513400 -0.99989900

C -4.07140800 -1.29080800 0.98784700

C -3.22885900 2.53295200 1.49187900

H 0.18426000 -1.33417600 -1.23087100

H 1.84004300 -2.81936500 -0.65401400

H 2.86936000 -2.56918300 0.79153100

H 3.87158100 -2.08829300 -1.46234600

H 2.85638800 -0.66672000 -1.64939600

H 5.01399400 -1.11725000 0.53352000

H -0.85086200 0.65351900 -1.38430900

H 0.52730400 1.19777000 -0.47220800

H 2.63091100 0.22279400 1.38023300

H 0.95212700 0.38781600 2.01465200

H 2.03869700 -0.96429900 2.49928800

H 6.32697800 0.64084000 1.20028900

H 6.60242700 1.79847500 -0.11440700

H 5.50552800 2.21783300 1.19127000

H 3.10418000 1.38542100 -1.61933200

H 3.69769500 2.68139100 -0.56278700

H 4.67593200 2.13368100 -1.91180600

H -1.20831800 -1.42659500 1.48180800

H -0.33192600 -2.80205400 0.82135800

H -1.82412000 -2.60057400 -1.30332400

H -2.48004100 -3.29770900 0.17736800

H -2.74445200 -0.65973500 -2.18424400

H -4.92812900 0.83397700 -0.58791500

H -4.55760600 1.01781100 -2.29700900

H -0.96363400 1.22780400 1.65470600

H -2.35977000 2.02110300 -1.79484800

H -3.66132500 2.93174900 -1.05892800

H -4.85483000 -0.53314600 0.95673800

H -4.55746100 -2.26254600 1.14209100

H -3.46229500 -1.09721400 1.87953500

H -2.77195100 2.35056900 2.46844900

H -3.28095500 3.61786100 1.33729000

H -4.26525000 2.17507100 1.52854200 **Structure TS^F_M^**

C 2.24129400 -2.35920800 0.10594200

C 2.97688900 -1.32350500 -0.76950600

C 3.48461400 -0.14087300 0.02261100

C 0.07865200 -0.32376300 0.34996200

C -0.22436100 -1.64597900 -0.37261700

C 0.83644800 -2.03831000 0.61383100

C 0.43854500 -2.72092300 1.90352700

C 3.54944000 1.14919000 -0.35806300

C 4.17184900 2.18821100 0.54346300

C 3.07671600 1.67704700 -1.69112700

C -1.61758500 -2.27344100 -0.38271700

C -2.52809900 -1.64918100 -1.47502600

C -2.92778500 -0.23035500 -1.11858400

C -2.17733700 0.80564000 -1.53578000

C -2.21184400 2.21953000 -1.02887900

C -0.80168700 0.31840900 1.36516500

C -1.27160800 1.58034300 1.26419600

C -1.09039400 2.45457700 0.04427700

C -4.13685300 -0.11862000 -0.22248300

C -2.12704300 2.15554300 2.36365700

H 0.17527700 -1.61602200 -1.38789400

H 2.09912100 -3.28732500 -0.46654000

H 2.87138200 -2.61092000 0.96532300

H 3.82820300 -1.85625500 -1.21412500

H 2.35478400 -1.01667800 -1.61693000

H 3.91688400 -0.39754900 0.99153900

H 0.66368300 0.34765400 -0.27511900

H 1.04437700 -0.71128300 1.03762600

H 1.18095000 -2.56701700 2.68999100

H -0.54716900 -2.43372700 2.26744100

H 0.40467600 -3.79538900 1.68311500

H 4.50796700 1.76271100 1.49252500

H 5.03639800 2.65412100 0.05451400

H 3.46337900 2.99803800 0.75898400

H 2.55173400 0.93834400 -2.30152100

H 2.42154100 2.54687800 -1.55595800

H 3.93328600 2.03122300 -2.27847000

H -2.10029100 -2.18869700 0.59551600

H -1.50168600 -3.34466600 -0.58847600

H -2.00070600 -1.67462400 -2.43630300

H -3.41370400 -2.28532600 -1.58300000

H -1.36497700 0.58893300 -2.23314600

H -3.18236400 2.47396200 -0.59470600

H -2.03681200 2.92977500 -1.84451600

H -1.06825500 -0.27196600 2.23699100

H -0.11799200 2.28383000 -0.43220600

H -1.11098800 3.50797700 0.34316800

H -4.35241300 0.90329300 0.09139600

H -5.02203900 -0.49690200 -0.74932500

H -4.02919500 -0.73094700 0.68162400

H -2.31734400 1.43750300 3.16507100

H -1.63532100 3.03417900 2.79918000

H -3.09149400 2.50088500 1.97314000 **Structure M^+^**

C 2.44761500 -2.05706200 0.47325400

C 3.31476300 -1.24274300 -0.51059500

C 3.89615800 0.00587900 0.10325900

C -0.61705500 0.16291800 0.08938900

C 0.12001100 -1.12961900 -0.09993400

C 1.20381400 -1.33472300 1.04192400

C 0.67422200 -2.07308700 2.28103500

C 3.94044400 1.25219800 -0.40166500

C 4.62251200 2.36927500 0.35366600

C 3.38151300 1.66124100 -1.74428800

C -0.81585000 -2.34128500 -0.31462200

C -1.75200300 -2.08960600 -1.52864300

C -2.64845600 -0.92525400 -1.21647800

C -2.36778600 0.33185500 -1.68548200

C -3.08844400 1.58212300 -1.29270600

C -1.39466900 0.58814400 1.18624000

C -2.17529300 1.72457000 1.10467600

C -2.32864000 2.45036100 -0.19365700

C -3.81154300 -1.19008300 -0.29788400

C -3.00506100 2.18097100 2.26219200

H 0.68224100 -1.00887900 -1.03109300

H 2.14487600 -3.00105000 0.00115000

H 3.07437600 -2.33848000 1.32910100

H 4.13517800 -1.89960600 -0.83329400

H 2.75481200 -1.01268800 -1.42377600

H 4.37265800 -0.15219700 1.07285400

H -0.29719200 0.95556100 -0.58041000

H 1.53440900 -0.33654900 1.35525500

H 1.43595900 -2.05668000 3.06624800

H -0.22830000 -1.62161400 2.70399600

H 0.45261700 -3.12332100 2.06465900

H 5.01499200 2.03239500 1.31687300

H 5.45788300 2.77996200 -0.22790900

H 3.93219800 3.20344500 0.53677600

H 2.84138300 0.86151700 -2.25653400

H 2.70936200 2.52357600 -1.63870200

H 4.19060400 1.98690800 -2.41061600

H -1.41334000 -2.53762600 0.58169100

H -0.21506700 -3.23762500 -0.49650100

H -1.15103800 -1.89869400 -2.42405100

H -2.34837300 -2.99058700 -1.71162600

H -1.58912600 0.42957700 -2.44048000

H -4.09457900 1.36844900 -0.92457900

H -3.19494000 2.23600600 -2.16350300

H -1.46278900 -0.03293800 2.07430300

H -1.35476500 2.72914600 -0.61151700

H -2.89519700 3.37355200 -0.05189300

H -3.85909200 -0.47502000 0.53074400

H -4.75322400 -1.09413000 -0.85286900

H -3.77728000 -2.19769100 0.12133100

H -2.90916000 1.52985200 3.13335700

H -2.70317900 3.19693700 2.55025700

H -4.06440500 2.25205000 1.98266300 **Structure TS^M_N^**

C -3.13599500 -1.38497300 -0.01382700

C -3.11543400 0.09465000 -0.41302500

C -1.85676100 0.44518800 -1.17937000

C -0.22119600 -0.02057300 0.08039900

C -0.59529400 -1.45752300 0.49312600

C -2.04464700 -1.75180300 1.00236200

C -2.34208900 -1.17000400 2.39725600

C -1.45831800 1.73728700 -1.49314200

C -0.47476700 1.98957200 -2.59020700

C -1.91971400 2.92886500 -0.73090000

C -0.08422200 -2.48314600 -0.55231100

C 1.42609300 -2.83184300 -0.38522900

C 2.33729000 -1.63264800 -0.55882800

C 2.70080000 -0.90185800 0.51413000

C 3.35424200 0.45494400 0.51580400

C 0.04053700 0.96516000 1.11241500

C 1.19237700 1.69260600 1.21130300

C 2.33279100 1.61535800 0.22388900

C 2.70018500 -1.28780400 -1.98270200

C 1.44016800 2.54374200 2.42698400

H 0.47591800 0.00692000 -0.74513500

H 0.02693600 -1.59896600 1.38813300

H -4.11443100 -1.62973700 0.41339000

H -3.03452600 -2.00103900 -0.91680500

H -3.23932800 0.73438200 0.46576000

H -3.97232700 0.30189500 -1.06797700

H -1.55998900 -0.30993500 -1.90898100

H -2.06798700 -2.84169900 1.12101600

H -3.28255000 -1.58215000 2.77656800

H -1.55685200 -1.43217300 3.11399500

H -2.44790300 -0.08090700 2.40206200

H -0.01692700 1.07645300 -2.97680100

H -1.00438800 2.47589100 -3.42221000

H 0.30292600 2.69603900 -2.27778200

H -2.92204300 2.82033300 -0.31393300

H -1.22306700 3.06773300 0.11591000

H -1.86865500 3.83872400 -1.33454900

H -0.65201000 -3.41383300 -0.44922900

H -0.27116800 -2.13147600 -1.57481200

H 1.67743800 -3.61218200 -1.11329400

H 1.56872900 -3.26948600 0.60935600

H 2.40276200 -1.27591200 1.49297600

H 4.14129900 0.53122400 -0.24119300

H 3.83546200 0.63687700 1.48189500

H -0.65827800 1.01806600 1.94256800

H 2.88474800 2.56128700 0.24237700

H 1.96227300 1.48152200 -0.79624300

H 3.29347700 -2.09937400 -2.42197300

H 1.80775600 -1.19209200 -2.61677200

H 3.28153000 -0.36805300 -2.07319500

H 0.61539900 2.50594400 3.14211200

H 2.35660800 2.22587200 2.93998300

H 1.60289500 3.58866600 2.13401600 **Structure N^+^**

C -3.12330400 -1.33383100 0.01411300

C -3.00278100 0.17014500 -0.25296800

C -1.69219000 0.43410000 -1.01154900

C -0.36663200 0.04160800 -0.04666400

C -0.56697200 -1.47029900 0.37023200

C -1.98031700 -1.86333900 0.90035000

C -2.20803900 -1.50214200 2.38167300

C -1.41807800 1.75714200 -1.50980900

C -0.64174300 1.93591200 -2.75756900

C -1.79115700 2.94865900 -0.74286200

C -0.01940800 -2.41831200 -0.73081800

C 1.49576100 -2.74473300 -0.56175400

C 2.38082300 -1.51484300 -0.62195200

C 2.69916800 -0.85887600 0.50950200

C 3.29886000 0.51987200 0.62921300

C -0.10292800 0.94523800 1.11197700

C 1.05420800 1.61556000 1.34699600

C 2.23753600 1.64885100 0.39830300

C 2.76647300 -1.05477000 -2.00768200

C 1.25594000 2.34507700 2.65181100

H 0.47651600 0.07566100 -0.73106800

H 0.10495100 -1.57831600 1.22814900

H -4.08532800 -1.54563300 0.49310800

H -3.13672500 -1.86306800 -0.94807500

H -3.03827900 0.73665500 0.68300200

H -3.84612300 0.50996500 -0.86498400

H -1.58154900 -0.28889800 -1.82806100

H -2.01377600 -2.95835300 0.85271000

H -3.12024800 -1.98502100 2.74668900

H -1.37891100 -1.84970500 3.00643800

H -2.32419900 -0.42776200 2.55501500

H -0.13592400 1.02744400 -3.08950800

H -1.36920900 2.21540700 -3.53991900

H 0.05699900 2.77677700 -2.69333900

H -2.78424500 2.88113900 -0.29097900

H -1.07830500 2.94199600 0.11520800

H -1.66379000 3.88385300 -1.28984300

H -0.56676200 -3.36676000 -0.70011700

H -0.19625900 -2.00941500 -1.73496200

H 1.78010500 -3.46257400 -1.34064700

H 1.62832800 -3.25253600 0.40038900

H 2.39440000 -1.31461900 1.45094700

H 4.10448100 0.68050300 -0.09499400

H 3.74763900 0.64558200 1.61990400

H -0.85549300 0.96794600 1.89630400

H 2.74385300 2.61552100 0.50520400

H 1.90321500 1.58365000 -0.64278000

H 3.38584000 -1.81754400 -2.49580200

H 1.88557800 -0.92684000 -2.65297200

H 3.33010200 -0.11946200 -2.01291700

H 0.39304900 2.25708500 3.31651800

H 2.13297100 1.95160600 3.18074700

H 1.45576500 3.40960200 2.47737700 **Structure TS^N_O^**

C 2.89509600 -1.27125400 -0.73543400

C 2.87625900 0.10357900 -0.04798700

C 1.57858500 0.38274100 0.69075900

C 0.27906100 0.08231200 -0.07661800

C 0.36006600 -1.27702700 -0.87430200

C 1.68382500 -1.47905200 -1.66053200

C 1.79046100 -0.65795500 -2.95790700

C 1.57575200 1.12251000 1.90669900

C 2.85014200 1.57572300 2.57455200

C 0.31694700 1.59679800 2.57027200

C 0.05648400 -2.49290000 0.05079300

C -1.45455500 -2.84494000 0.17780700

C -2.23436100 -1.67866600 0.74340500

C -2.73312500 -0.75499800 -0.09562200

C -3.14015700 0.65765300 0.21922100

C -0.03259600 1.31854800 -0.91484900

C -1.19490900 1.94736600 -1.19815700

C -2.65571300 1.66879600 -0.84333800

C -2.23945200 -1.56313500 2.24882400

C -1.11388900 3.15850000 -2.11445800

H -0.52171100 -0.04682100 0.64350500

H -0.44727000 -1.22032300 -1.61119900

H 3.82838200 -1.36321200 -1.30064700

H 2.92190900 -2.05434900 0.03322700

H 2.95121900 0.89966400 -0.80183700

H 3.74899900 0.22102300 0.59527400

H 1.56448000 -0.27774200 1.77556400

H 1.69458400 -2.53122600 -1.97010200

H 2.67117700 -0.97067100 -3.52876500

H 0.91213100 -0.81771200 -3.59115800

H 1.87449200 0.41943300 -2.79058300

H 3.66997700 0.86132100 2.49733000

H 3.17591700 2.49830500 2.07546300

H 2.67797600 1.80511700 3.62703600

H -0.59978700 1.21237500 2.13281100

H 0.33920200 1.39305400 3.64432900

H 0.31172500 2.68970200 2.45496300

H 0.57165200 -3.37861400 -0.33775100

H 0.47512800 -2.33309900 1.05669200

H -1.55399800 -3.73868900 0.80529500

H -1.83105600 -3.10782700 -0.81739800

H -2.73179400 -0.99558300 -1.15968000

H -2.79849200 0.95846300 1.21654300

H -4.23463000 0.75556200 0.24327200

H 0.84219500 1.75794500 -1.39037700

H -3.13629600 1.40241000 -1.79725000

H -3.09468900 2.64226400 -0.58770100

H -2.82754800 -2.38476400 2.67666600

H -1.23105800 -1.66416000 2.67536100

H -2.68019800 -0.63210600 2.61248700

H -0.09580100 3.35444300 -2.45887600

H -1.74936600 3.02114100 -2.99766200

H -1.48099100 4.05706600 -1.60360000 **Structure O^+^**

C -2.85594900 -1.56054400 -0.34351800

C -2.78809600 -0.04148300 -0.62785300

C -1.39687400 0.42662600 -0.86848100

C -0.31997300 -0.11909300 -0.02837700

C -0.47930900 -1.56495300 0.56579500

C -1.95787800 -1.96612000 0.83586700

C -2.50838900 -1.47857100 2.18845700

C -1.18145100 1.42517300 -1.95756600

C -2.05121800 2.70127800 -1.75343900

C 0.25981500 1.79730500 -2.30920700

C 0.25245800 -2.62124800 -0.31707200

C 1.78059200 -2.74538700 -0.05710900

C 2.50508500 -1.46590300 -0.41404200

C 2.66482200 -0.51339700 0.52212300

C 2.94113200 0.94921000 0.29168500

C -0.35467400 1.02701500 1.01016900

C 0.65075800 1.80222300 1.50391400

C 2.15257300 1.86334700 1.25398500

C 2.86016000 -1.30304600 -1.87165700

C 0.26267200 2.75588700 2.61948600

H 0.63186300 -0.03657100 -0.54475100

H 0.03723800 -1.54534400 1.53176800

H -3.89992900 -1.82227100 -0.14415300

H -2.56585500 -2.10535800 -1.24960200

H -3.16127800 0.49879700 0.25738900

H -3.44553000 0.25152700 -1.45066300

H -1.62635200 0.89707900 -2.82416500

H -1.97696900 -3.06121800 0.88381200

H -3.50566800 -1.89517500 2.36431300

H -1.86396700 -1.80784300 3.00956100

H -2.59289500 -0.38979600 2.26395100

H -3.10402900 2.47942200 -1.56926900

H -1.66074500 3.28030800 -0.91169200

H -1.98960000 3.31577400 -2.65436400

H 0.87355400 0.92273300 -2.53821800

H 0.25650700 2.43630200 -3.19612200

H 0.73340500 2.35960800 -1.49983900

H -0.19216700 -3.60219600 -0.11633200

H 0.07142200 -2.42509700 -1.38283800

H 2.16128100 -3.59178500 -0.64100400

H 1.93453700 -2.99058600 1.00029200

H 2.43952800 -0.78224500 1.55533900

H 2.74101900 1.22348400 -0.74820900

H 3.99976500 1.19033900 0.46090900

H -1.32015400 1.16981900 1.48383300

H 2.59254200 1.74110700 2.25476900

H 2.37878800 2.90825600 0.99729300

H 3.63820200 -2.02972900 -2.13753100

H 2.00800200 -1.51924000 -2.53018100

H 3.24868700 -0.31284000 -2.11843600

H -0.81354300 2.77756000 2.80135100

H 0.76381200 2.48089900 3.55497600

H 0.59098500 3.77436200 2.37885200 **Structure TS^O_P^**

C -2.12244400 -2.42677500 -0.33748500

C -2.90086900 -1.14598700 -0.02884400

C -2.10656000 0.11976800 -0.14725600

C -0.67901900 0.06071300 -0.32057700

C 0.10237300 -1.25160900 -0.11692400

C -0.76524800 -2.43326500 0.38410900

C -0.91353900 -2.44090900 1.91609400

C -2.84686700 1.44012900 -0.16985100

C -3.53727800 1.68181800 -1.53432600

C -3.87092500 1.51129100 0.98778500

C 0.97590600 -1.61742600 -1.35619500

C 2.45191400 -1.95528400 -0.98052400

C 3.11034700 -0.70418500 -0.44001500

C 3.00677900 -0.43597900 0.87517600

C 3.04047300 0.90810000 1.54497100

C 0.08878100 1.35042200 -0.48687400

C 1.02184300 1.94120100 0.29415900

C 1.57461900 1.46483300 1.62381100

C 3.67673100 0.24010100 -1.47115000

C 1.57138800 3.27862900 -0.15278400

H 0.79693600 -0.98301100 0.68241800

H -2.72584700 -3.29048400 -0.04292900

H -1.97032300 -2.51109700 -1.42215600

H -3.81550400 -1.06760400 -0.62742100

H -3.24862700 -1.15579900 1.01492500

H -1.26995400 -0.06245800 -1.37283900

H -0.24292800 -3.35531400 0.10462600

H -1.56985100 -3.25430000 2.24278900

H 0.05849900 -2.58978500 2.39612900

H -1.32501800 -1.50402500 2.31016100

H -2.11078300 2.23301900 -0.01544200

H -4.03297600 2.65599600 -1.51381000

H -2.81823300 1.69327100 -2.36040400

H -4.29871500 0.92529800 -1.74671700

H -4.28514800 2.52257700 1.01620400

H -3.40775400 1.31669300 1.95987000

H -4.70266300 0.81548500 0.84790300

H 0.99037900 -0.79226900 -2.07696000

H 0.52472100 -2.47188200 -1.87530400

H 2.46266600 -2.75948300 -0.23503900

H 2.96104500 -2.33417500 -1.87346400

H 2.66525700 -1.24493100 1.52357600

H 3.68251600 1.62423900 1.02545300

H 3.42022500 0.82842300 2.56884400

H -0.18602500 1.90051300 -1.38473800

H 0.93311800 0.70291000 2.07880000

H 1.56934900 2.32279100 2.30817000

H 4.49975100 -0.25215700 -2.00413300

H 2.93525900 0.51727500 -2.23096100

H 4.07313800 1.15911200 -1.03612900

H 1.20866300 3.57222500 -1.14049600

H 1.29238800 4.06091600 0.56368600

H 2.66601200 3.26452800 -0.18442300 **Structure P^+^**

C -2.97657800 0.52677600 1.05471800

C -2.32598200 -0.83401300 1.32100200

C -1.11769400 -1.21845000 0.36387900

C -0.26461600 0.01451900 0.41147300

C -0.86855800 1.26207300 -0.14713000

C -1.97249500 1.68524900 0.92955900

C -2.67919600 2.99532100 0.56061100

C -1.52599600 -1.84831600 -1.01949900

C -1.70391600 -3.36690700 -0.82001000

C -2.76772100 -1.28852400 -1.73400900

C 0.08565800 2.39013600 -0.57539700

C 1.01563700 1.99930300 -1.76518300

C 2.11537000 1.06713400 -1.32505000

C 2.00033200 -0.27217800 -1.49368100

C 2.82888500 -1.32258300 -0.82835100

C 0.83988600 0.06886400 1.30576700

C 1.83321300 -0.87396900 1.47682400

C 2.10477200 -1.93395400 0.45902700

C 3.27327500 1.73851800 -0.63112500

C 2.86062100 -0.69442600 2.55269000

H -1.44778100 0.97668500 -1.03133500

H -3.58831200 0.49456100 0.14766000

H -3.66571100 0.74521200 1.87928800

H -1.94217800 -0.85755100 2.34696400

H -3.06186300 -1.64121900 1.24521700

H -0.59296300 -2.00341800 0.91104400

H -1.46020300 1.82722100 1.89130500

H -3.49701200 3.17439000 1.26638600

H -2.01415300 3.86034600 0.61094100

H -3.11256200 2.94821900 -0.44481000

H -0.66997500 -1.70724500 -1.69104600

H -2.53095900 -3.58587100 -0.13501900

H -1.93228000 -3.85219600 -1.77338700

H -0.80144400 -3.83890700 -0.41679900

H -2.90453600 -1.82400800 -2.67847800

H -2.69527300 -0.22670300 -1.98206100

H -3.67764900 -1.44105000 -1.14509600

H 0.68361200 2.75289300 0.26865400

H -0.52575800 3.23603200 -0.89836400

H 0.41089100 1.54499800 -2.55828800

H 1.44028800 2.92386500 -2.17339200

H 1.16909500 -0.63352200 -2.09876400

H 3.81225500 -0.95267100 -0.53138900

H 2.99355400 -2.17382300 -1.49622200

H 0.97662800 1.01457900 1.83149400

H 1.19368700 -2.41401200 0.10113900

H 2.75743000 -2.70867900 0.87031500

H 3.96386300 1.04720800 -0.14785400

H 3.84790600 2.31196500 -1.37021700

H 2.93359100 2.46260500 0.11793300

H 2.69519600 0.20206700 3.15358800

H 2.83866700 -1.56689000 3.21960700

H 3.87457600 -0.66253100 2.13430200 **Structure TS^P_E^**

C 3.08308700 -1.25111100 0.32134400

C 3.02724800 0.18573000 0.91344400

C 1.66375700 0.84952200 0.57767600

C 0.61257600 -0.31322900 0.65883300

C 0.68680500 -1.30416200 -0.56575700

C 1.68865100 -1.85696500 0.38869600

C 1.47624100 -3.17146000 1.09180800

C 1.68255300 1.75749800 -0.70149400

C 2.03623400 3.19614000 -0.27399900

C 2.62677000 1.34236800 -1.84932600

C -0.48572900 -2.18209100 -1.01948400

C -1.42287000 -1.51880100 -2.06355600

C -2.35217000 -0.51754000 -1.41098200

C -2.01222700 0.78178200 -1.37795100

C -2.56481800 1.84466000 -0.47326500

C -0.66091600 -0.25488200 1.48712100

C -1.57474200 0.72932000 1.60517000

C -1.64302300 1.99823600 0.78683900

C -3.57781600 -1.11072800 -0.75998900

C -2.70287800 0.55487700 2.59641400

H 1.14878700 -0.81430700 -1.41750100

H 3.40039900 -1.23089000 -0.72521300

H 3.79052900 -1.86905700 0.87992300

H 3.11762100 0.10070000 2.00334900

H 3.87626100 0.78150300 0.57507900

H 1.40534200 1.50030700 1.41831900

H 1.21399900 -0.97165800 1.45878800

H 2.07145000 -3.24781800 2.00457400

H 0.42874700 -3.38216500 1.31027800

H 1.82708900 -3.94760000 0.39784500

H 0.66776000 1.77298800 -1.11001000

H 3.03882800 3.24305500 0.16754900

H 2.02728700 3.86860200 -1.13680900

H 1.32988300 3.59025900 0.46405200

H 2.52907600 2.06306600 -2.66657700

H 2.41394000 0.35886800 -2.27693800

H 3.67794800 1.35593200 -1.54312500

H -1.07728400 -2.52566300 -0.16585300

H -0.04325500 -3.07804100 -1.47382000

H -0.81740400 -1.03996900 -2.84259800

H -1.99329800 -2.31718100 -2.55243400

H -1.13804700 1.08044300 -1.95755400

H -3.58641200 1.62286200 -0.15344100

H -2.59736400 2.81653400 -0.97784500

H -0.81587900 -1.13960000 2.10210200

H -0.65418600 2.31444200 0.45292600

H -2.03996500 2.80781400 1.40994400

H -4.20039600 -0.36903700 -0.25827900

H -4.19762200 -1.60039700 -1.52170400

H -3.32090300 -1.88348900 -0.02464600

H -2.69213700 -0.42558200 3.07874500

H -2.63430200 1.32066600 3.37875100

H -3.67690100 0.68943300 2.11286900

**Structure TS^F_G^**

C 2.00358300 2.00147500 0.70303200

C 2.90860300 1.71063600 -0.53719300

C 3.95395200 0.65532500 -0.29122400

C -0.45373800 0.67164200 -0.36352600

C 0.27789600 0.08332900 0.84201500

C 1.41631200 0.76211400 1.36370400

C 2.10353700 0.36532800 2.64372000

C 4.16861700 -0.47937100 -0.98849600

C 5.31558200 -1.39340000 -0.62812300

C 3.37563800 -0.92543500 -2.19426600

C -0.34014100 -1.08475500 1.61128600

C -1.07618800 -2.20959900 0.82595500

C -2.45291500 -1.79857100 0.34822900

C -2.66891100 -1.60084800 -0.96263200

C -3.76345600 -0.78840600 -1.58557300

C -1.71072100 1.40751000 0.07101900

C -2.89577500 1.42799400 -0.57091300

C -3.21313300 0.65658500 -1.83996300

C -3.48143800 -1.57118700 1.43090200

C -4.03032500 2.26551600 -0.03054300

H 1.20713300 2.69456800 0.41804300

H 2.60949500 2.50185200 1.46334900

H 3.38912400 2.66355600 -0.79150700

H 2.27949300 1.44902400 -1.39182400

H 4.64561100 0.87555300 0.52325700

H 0.21128300 1.35321100 -0.89966000

H -0.70618800 -0.13502400 -1.04963700

H 1.47918700 -0.26176600 0.46622100

H 3.18618700 0.46131600 2.53793200

H 1.86360900 -0.62672700 3.01861200

H 1.78731500 1.09626300 3.40051300

H 5.86877700 -1.03843200 0.24517800

H 4.96010900 -2.41106800 -0.42400300

H 6.01909100 -1.47326800 -1.46614600

H 2.51476200 -0.29343000 -2.42301500

H 4.02069300 -0.93210300 -3.08163800

H 3.02356900 -1.95644500 -2.06644300

H 0.42864100 -1.55490400 2.22489900

H -1.04029400 -0.62492600 2.32096200

H -0.45688200 -2.54513100 -0.01415200

H -1.14540800 -3.05673300 1.52035400

H -1.86926900 -1.87909100 -1.65254100

H -4.65617400 -0.73662200 -0.95623300

H -4.07204700 -1.20566600 -2.55015700

H -1.60511400 2.02303400 0.96475200

H -2.33033200 0.58149200 -2.48520500

H -3.96263400 1.21495800 -2.41175000

H -3.55071100 -2.45814600 2.07307600

H -3.23092300 -0.72449100 2.08248300

H -4.47674700 -1.38338300 1.02631500

H -3.78264700 2.73747000 0.92380600

H -4.29979500 3.05437900 -0.74333000

H -4.93077700 1.65538600 0.11303100 **Structure G+**

C 2.31408400 -2.06238300 0.77477800

C 2.97433200 -0.73450600 1.18430000

C 3.71524700 -0.06405500 0.05141600

C -0.01913600 0.17151900 -0.22973400

C 0.08298900 -1.25799800 0.10813500

C 1.25296500 -2.00527300 -0.40734000

C 0.96815900 -3.42911300 -0.92045200

C 3.86117600 1.25295700 -0.18621700

C 4.70376800 1.73971800 -1.34131500

C 3.27015200 2.35094800 0.66497100

C -0.97085100 -1.93993700 0.86087400

C -2.28339500 -2.18245000 -0.07212600

C -3.11533900 -0.94275600 -0.17762300

C -3.02678500 -0.17422700 -1.28562700

C -3.43631900 1.26113600 -1.41250100

C -0.74063000 1.13583000 0.67761500

C -1.65935500 2.04672200 0.29924400

C -2.19722500 2.17899000 -1.11385900

C -3.93650400 -0.60400600 1.04184900

C -2.23980800 3.00859400 1.30646200

H 1.85647100 -2.54945200 1.64278400

H 3.08445200 -2.74722800 0.40094500

H 3.68268100 -0.98454200 1.98776000

H 2.24401700 -0.05918300 1.64342600

H 4.24126200 -0.75141000 -0.61408800

H 0.96912500 0.53904500 -0.54295100

H -0.58570100 0.07505800 -1.18838700

H 1.72204800 -1.41247700 -1.20148500

H 1.89257900 -3.83983000 -1.33502300

H 0.22023500 -3.43310800 -1.71822400

H 0.63656400 -4.10679900 -0.12874800

H 5.11680300 0.91399100 -1.92673500

H 4.11901700 2.38091500 -2.01326100

H 5.53986800 2.35095600 -0.97906600

H 2.60709800 1.98944800 1.45397300

H 4.07242800 2.92243500 1.14874800

H 2.71364600 3.06667600 0.04699700

H -0.63878400 -2.91736200 1.21197600

H -1.30855700 -1.33597000 1.70661100

H -1.96812300 -2.55678900 -1.04842500

H -2.80537500 -2.99263500 0.44588000

H -2.47523000 -0.57604200 -2.13820900

H -4.25166400 1.51973400 -0.73212500

H -3.78068200 1.48413300 -2.42744400

H -0.42153000 1.12253300 1.71910700

H -1.42129200 1.95769300 -1.85616300

H -2.49709900 3.21810000 -1.28641600

H -4.63206000 -1.42324600 1.26035100

H -3.31428000 -0.46964500 1.93585100

H -4.52633000 0.30372600 0.91687900

H -1.88320000 2.81325800 2.32094000

H -1.97775100 4.04103900 1.04495700

H -3.33578000 2.95871800 1.31247500 **Structure TS^G_Q^**

C 1.85128800 -0.76072400 -2.05847000

C 2.74082000 0.44801500 -1.66550200

C 3.73014500 0.19831200 -0.55711200

C -0.36736700 0.48238100 -0.46445300

C 0.25163100 -0.69758900 0.01392400

C 1.09253500 -1.54665600 -0.94375700

C 1.98852400 -2.57386500 -0.22287900

C 3.98127900 0.95976300 0.52579700

C 5.06931700 0.57534900 1.50070300

C 3.27818800 2.25384900 0.86328300

C -0.16558900 -1.25442900 1.34566900

C -1.41394400 -2.20082800 1.10180500

C -2.64035100 -1.40071900 0.72710800

C -2.89866200 -1.13056100 -0.56949000

C -3.82962800 -0.08611100 -1.11216500

C -1.23687800 1.42484100 0.25918700

C -2.41989900 1.85913200 -0.23543400

C -3.05086900 1.24329200 -1.45838000

C -3.44004300 -0.87886600 1.89551300

C -3.23999700 2.87462500 0.51127100

H 0.82736200 0.51996100 0.07544300

H 1.14136800 -0.42341500 -2.82394300

H 2.47571500 -1.50399300 -2.56419000

H 3.28811000 0.71889400 -2.57968600

H 2.12930200 1.33040600 -1.44661200

H 4.34961800 -0.68841700 -0.69311600

H -0.20939700 0.68782700 -1.52276800

H 0.32395400 -2.12844800 -1.47953600

H 2.55440200 -3.13926200 -0.96745500

H 1.40294700 -3.29945100 0.34771100

H 2.70447600 -2.09581200 0.44896100

H 5.55881900 -0.36157100 1.22228600

H 5.83785400 1.35692400 1.55076400

H 4.67000100 0.46768500 2.51751000

H 2.46009500 2.50901800 0.18431100

H 2.88645300 2.22843800 1.88838100

H 3.98980600 3.08848400 0.83130200

H -0.46162200 -0.46151800 2.03564500

H 0.63859700 -1.82409800 1.81052500

H -1.16473500 -2.93876100 0.33320200

H -1.56487000 -2.74796700 2.03804900

H -2.28496000 -1.63133400 -1.32139300

H -4.63677200 0.14832200 -0.41352300

H -4.30149600 -0.42974900 -2.03854200

H -0.90249100 1.76920300 1.23472500

H -2.29946200 1.00301100 -2.22113100

H -3.75584200 1.94520400 -1.91432900

H -4.24978400 -0.21039400 1.60241300

H -3.88691000 -1.71964300 2.44063600

H -2.80726400 -0.34386000 2.61467800

H -2.77437000 3.17888400 1.45161600

H -3.37756400 3.76915700 -0.10895900

H -4.24435600 2.49100700 0.72731700 **Structure Q^+^**

C -1.63388900 -0.05129300 1.88425500

C -2.55629000 0.98651600 1.20167900

C -3.88872400 0.46050100 0.72942100

C 0.89873600 0.25750700 0.29130100

C -0.20760200 -0.63586900 -0.17891000

C -1.02337500 -1.19419800 1.02537600

C -2.07190300 -2.23789500 0.60380600

C -4.53068900 0.71811300 -0.42398400

C -5.90053900 0.13951600 -0.69139500

C -3.99960100 1.59380100 -1.53412600

C 0.33767600 -1.72498900 -1.16710500

C 1.55230200 -2.50734300 -0.60879800

C 2.71860300 -1.56590100 -0.49910200

C 3.10643400 -1.06326100 0.71140300

C 4.08081800 0.04652000 0.91425800

C 1.46017500 1.33415500 -0.41028000

C 2.61625700 1.96571100 0.02109500

C 3.38327900 1.45718300 1.19612100

C 3.38116400 -1.13795800 -1.78468800

C 3.20341300 3.10194100 -0.75180700

H -0.87513900 -0.01921700 -0.79927500

H -0.81677100 0.50570200 2.36527300

H -2.18195900 -0.51980100 2.71104500

H -2.74153600 1.76629800 1.95567100

H -2.02491900 1.49664300 0.39043800

H -4.40436700 -0.17141600 1.45425300

H 1.14634600 0.18152900 1.34393900

H -0.30964600 -1.70924700 1.68738800

H -2.69590800 -2.50404600 1.46183500

H -1.60998200 -3.16364900 0.24802900

H -2.73775400 -1.85860900 -0.17622200

H -6.25165400 -0.48204000 0.13674600

H -6.63702100 0.93711600 -0.85311400

H -5.89941100 -0.47218400 -1.60329400

H -2.99011900 1.97022900 -1.35145800

H -3.99692600 1.05057300 -2.48820800

H -4.65215800 2.46446800 -1.67842900

H 0.62707400 -1.23048500 -2.10093000

H -0.47548500 -2.40671200 -1.42338600

H 1.30997200 -2.95651000 0.35927300

H 1.78530000 -3.32310200 -1.30367400

H 2.66481800 -1.49563500 1.60819200

H 4.75686600 0.16033300 0.06395800

H 4.69529100 -0.14929400 1.79816300

H 1.02398500 1.62814500 -1.36278300

H 2.73883800 1.33289200 2.07279700

H 4.17228300 2.16173700 1.46851200

H 3.35462900 -0.04968200 -1.91984700

H 4.43724900 -1.43124700 -1.78480300

H 2.90967700 -1.59591200 -2.65644900

H 2.64083500 3.33466700 -1.65783500

H 3.23249700 3.99812300 -0.11701800

H 4.24762000 2.89378900 -1.01990100

**Structure TS^Q_R^**

C -1.03681800 2.22086900 -1.48907500

C -2.02918900 1.05637500 -1.38108400

C -2.45130100 0.80960800 0.05235400

C -0.61830100 0.19431900 0.89288500

C 0.14481500 1.51468000 0.66716100

C 0.32496600 2.03597300 -0.77906700

C 1.05426600 3.39200000 -0.80486900

C -3.29069100 -0.22506500 0.45155000

C -3.92362200 -0.22561700 1.80304800

C -3.54428400 -1.38837200 -0.43922400

C 1.39724000 1.56326600 1.63738400

C 2.81038000 1.11935700 1.17657600

C 2.85629800 -0.27958000 0.61436500

C 2.81751600 -0.42129700 -0.72129300

C 2.41940300 -1.63041100 -1.50613100

C -0.41064100 -1.20892100 0.54930500

C 0.12656900 -1.97749300 -0.45502500

C 0.85793400 -1.57531700 -1.70482000

C 2.89022900 -1.40880900 1.61376100

C 0.03412300 -3.47813000 -0.28814100

H -0.51742400 2.25036800 1.14314600

H -0.99829500 0.22428900 1.91308700

H -0.84842900 2.43120800 -2.54748700

H -1.52209300 3.12181100 -1.08686600

H -2.92372800 1.30007000 -1.97110700

H -1.61752800 0.14109900 -1.82133100

H -2.50513100 1.69825500 0.67965800

H 0.91921100 1.31875600 -1.34950500

H 1.08801800 3.78136400 -1.82725400

H 2.08302400 3.33127800 -0.44610000

H 0.52718100 4.13240000 -0.19102700

H -3.63634300 0.63352400 2.41286500

H -5.01680100 -0.21971500 1.68967600

H -3.69045700 -1.15397400 2.34180300

H -3.94790600 -1.06143600 -1.40559800

H -2.58436800 -1.87979800 -0.66958000

H -4.22034600 -2.12161400 0.00369700

H 1.47938900 2.60755200 1.95872900

H 1.13109400 1.00711300 2.54506400

H 3.46126500 1.21611500 2.05571300

H 3.19034600 1.82282900 0.43114200

H 2.89353500 0.48735500 -1.31741200

H 2.70513100 -2.57176100 -1.02924000

H 2.87692500 -1.62411900 -2.50030400

H -0.87448300 -1.82567500 1.31949700

H 0.58918100 -0.57292700 -2.04096700

H 0.58354100 -2.27503900 -2.50309600

H 3.84634500 -1.38711000 2.15260000

H 2.10457900 -1.31355900 2.37209400

H 2.79782500 -2.39457600 1.15375500

H -0.38557900 -3.77702800 0.67441600

H -0.58284400 -3.91002500 -1.08654300

H 1.02179700 -3.94163400 -0.39148500

**Structure R^+^**

C 1.04054400 2.21633600 1.48715000

C 1.95822500 0.99346300 1.39006300

C 2.26590600 0.69384200 -0.07977200

C 0.79073200 0.24118900 -0.84292500

C -0.08876300 1.54198100 -0.70086800

C -0.30013800 2.09121600 0.72902100

C -0.97025300 3.47714400 0.71247300

C 3.24143200 -0.30698600 -0.41333100

C 3.95318000 -0.25504700 -1.70993700

C 3.48167100 -1.44541300 0.48093000

C -1.33592400 1.51144300 -1.65918600

C -2.75595800 1.13091900 -1.15927100

C -2.84217300 -0.25470200 -0.56719900

C -2.78756300 -0.37934600 0.76913400

C -2.39209400 -1.58663900 1.56259500

C 0.42064700 -1.19764400 -0.57036900

C -0.15222500 -1.96512600 0.39703000

C -0.82762100 -1.56969800 1.68888600

C -2.92743700 -1.39694400 -1.54882200

C -0.18304200 -3.46476600 0.16143600

H 0.56015500 2.28781600 -1.18431100

H 1.07409300 0.24497100 -1.89865700

H 0.83594800 2.43244300 2.54154000

H 1.58721400 3.08911400 1.10134800

H 2.89462800 1.19860000 1.92362500

H 1.50534000 0.11949900 1.86794800

H 2.49188900 1.61027900 -0.63535000

H -0.94380500 1.40430100 1.28299600

H -1.04752400 3.87289000 1.73038700

H -1.97747600 3.45865300 0.29267000

H -0.37698400 4.19039500 0.12721200

H 3.58331200 0.51964200 -2.38346300

H 5.01564000 -0.05018800 -1.49719600

H 3.94742100 -1.23521900 -2.20359200

H 3.67764300 -1.11718300 1.50869700

H 2.51265100 -1.98368800 0.54305200

H 4.26036500 -2.12591400 0.13537900

H -1.41751900 2.52272400 -2.07506300

H -1.08173200 0.87340800 -2.51504600

H -3.42145300 1.23120900 -2.02710800

H -3.09647800 1.86433700 -0.42318100

H -2.83084100 0.53901000 1.35366700

H -2.72579400 -2.52664900 1.11511500

H -2.81229500 -1.54971800 2.57276200

H 0.79840300 -1.80665300 -1.39428600

H -0.52636300 -0.57618700 2.02661900

H -0.52794500 -2.28279900 2.46747000

H -3.87249000 -1.32768200 -2.10310500

H -2.12539300 -1.36199900 -2.29522300

H -2.90074200 -2.37778400 -1.07076400

H 0.23113000 -3.74938500 -0.80859400

H 0.37984000 -3.98678600 0.94561500

H -1.20570400 -3.85278900 0.21659200

**Structure TS^R_S^**

C 1.23765400 -2.10720700 -1.36170200

C 1.95258900 -0.74684900 -1.36682100

C 2.09212100 -0.18918500 0.03955600

C 0.75594000 -0.07829300 0.80630500

C 0.00170600 -1.46414600 0.75611700

C -0.12620400 -2.11066900 -0.64240900

C -0.62968200 -3.56243000 -0.56618800

C 3.28430800 0.45984500 0.47815200

C 4.51447400 0.52473600 -0.38206200

C 3.40274600 1.14968500 1.80104200

C -1.24251800 -1.53662900 1.70170700

C -2.68649200 -1.31446400 1.17283200

C -2.90087500 0.03503200 0.52922300

C -2.84436500 0.11942900 -0.80994300

C -2.58217000 1.33399600 -1.64750300

C 0.21219300 1.33429000 0.49755900

C -0.39817700 1.99816500 -0.51062400

C -1.02784600 1.48095100 -1.78493200

C -3.11840600 1.19489500 1.46928000

C -0.55236200 3.50140400 -0.35969700

H 0.73606300 -2.10880800 1.27279400

H 1.00052900 -0.00510600 1.86888600

H 1.10970800 -2.43924200 -2.39739100

H 1.89624700 -2.84839100 -0.88411500

H 1.36652600 -0.00605600 -1.92236000

H 2.91135700 -0.82735800 -1.88118300

H -0.83062500 -1.53052500 -1.24420000

H -0.64770400 -4.01406100 -1.56353700

H -1.63828200 -3.63750800 -0.15596300

H 0.03171900 -4.17335300 0.06061000

H 2.83946100 -0.94027100 0.67266900

H 5.40808300 0.71527100 0.21362300

H 4.67215100 -0.36351100 -0.99430500

H 4.37866600 1.36891000 -1.07356400

H 2.54635300 1.03002900 2.46032900

H 3.50951900 2.22004900 1.57281400

H 4.31997000 0.85154800 2.31701000

H -1.23518700 -2.53491300 2.15434600

H -1.06315400 -0.84492100 2.53517100

H -3.35485700 -1.45007600 2.03345500

H -2.94114400 -2.10314000 0.45875200

H -2.78269700 -0.81845100 -1.36177400

H -3.01063300 2.24742700 -1.22594000

H -3.00582800 1.21876600 -2.65062100

H 0.50454000 1.99463400 1.31447400

H -0.62791100 0.50926000 -2.08574000

H -0.80526300 2.18687800 -2.59515600

H -4.06902200 1.05641400 2.00101400

H -2.33770400 1.25630700 2.23609000

H -3.16412700 2.15879500 0.95942200

H -0.20914300 3.86430000 0.61211200

H 0.01957700 4.01910500 -1.14039600

H -1.59499700 3.81265000 -0.48668100

**Structure S^+^**

C 0.55088100 2.29905900 1.45833600

C 1.52255500 1.10302600 1.42423200

C 1.71022200 0.55415800 0.01023400

C 0.55245400 0.58929700 -0.87125200

C -0.59898500 1.59331300 -0.65904700

C -0.81835100 2.01730100 0.80823300

C -1.71422200 3.26113700 0.91958600

C 3.15964200 0.50906600 -0.48827600

C 4.01867300 -0.50002100 0.29756100

C 3.36415300 0.35836000 -2.00167900

C -1.84233400 1.37166200 -1.57026000

C -3.05176900 0.53726100 -1.07722900

C -2.66774000 -0.84619600 -0.61345000

C -2.58691200 -1.07348300 0.70826600

C -1.88601200 -2.19288200 1.40626200

C 0.84367300 -0.91505300 -0.43396600

C 0.45972600 -1.85698300 0.51678100

C -0.40356800 -1.72242900 1.72234000

C -2.34911300 -1.85368800 -1.68983200

C 0.98015800 -3.25426800 0.30963700

H -0.12013200 2.47567100 -1.11954400

H 0.80469100 0.56566000 -1.93062000

H 0.41307500 2.60447600 2.50124600

H 1.01408700 3.15514100 0.94817400

H 1.18478100 0.33136600 2.11809600

H 2.50150700 1.41397300 1.79971400

H -1.30214800 1.20052600 1.35475200

H -1.82095800 3.56408200 1.96593500

H -2.71846900 3.08692500 0.52492000

H -1.28168000 4.10757400 0.37272100

H 3.51675900 1.51716500 -0.21969700

H 5.07655900 -0.35537600 0.06349200

H 3.90137600 -0.39535900 1.37997600

H 3.75656900 -1.52732600 0.02230400

H 4.43078400 0.44584600 -2.22637700

H 2.85052500 1.13733700 -2.57256000

H 3.04478500 -0.61945100 -2.37956500

H -2.23219600 2.36335900 -1.82441400

H -1.48153800 0.95708600 -2.52046800

H -3.76817400 0.49055200 -1.90746500

H -3.55331500 1.06821200 -0.26198800

H -2.92035100 -0.27739400 1.37282400

H -1.87385000 -3.12587400 0.83765300

H -2.34943200 -2.41144100 2.37289700

H 1.41345200 -1.36715500 -1.23725400

H -0.46566100 -0.70277100 2.09983900

H 0.00058000 -2.36714800 2.51129900

H -3.25299300 -2.05607200 -2.27833800

H -1.60056700 -1.47693200 -2.39646400

H -1.99671000 -2.81077600 -1.29899200

H 1.38210100 -3.42491200 -0.69014500

H 1.78168400 -3.43979700 1.03873200

H 0.20606400 -3.99898700 0.52044700

**Structure TS^S_T^**

C 1.59520200 2.86365800 0.31103900

C 2.23286500 1.55434500 0.77610700

C 1.86898600 0.34021000 -0.02755500

C 0.61705100 0.32428700 -0.75022100

C -0.27692300 1.64201600 -0.86578200

C 0.07709500 2.72701100 0.17895600

C -0.55573200 4.08643700 -0.15556800

C 3.01324100 -0.66528000 -0.15135800

C 3.45014200 -1.21846900 1.23393000

C 2.94978500 -1.78597500 -1.19647600

C -1.81333600 1.45392300 -1.04569900

C -2.71184200 1.25547700 0.20637200

C -2.72399700 -0.19037300 0.64677500

C -1.82278700 -0.62169900 1.54364500

C -1.37520400 -2.03760600 1.75360500

C 0.02279900 -0.95229900 -1.26465100

C -0.26950200 -2.07126100 -0.56692100

C -0.06879100 -2.25648200 0.92116900

C -3.71628700 -1.06779500 -0.07592200

C -0.90062100 -3.24077300 -1.28203500

H 0.03173700 2.05580700 -1.83854300

H 1.51000800 0.59004100 -1.49898500

H 1.84542700 3.64682000 1.03314500

H 2.03784800 3.17218800 -0.64661800

H 1.85857200 1.31926600 1.78770300

H 3.31833800 1.64409300 0.86462200

H -0.30536900 2.40328700 1.15658400

H -0.22730700 4.84154400 0.56547300

H -1.64702300 4.05918800 -0.12226500

H -0.25646500 4.42731100 -1.15401300

H 3.83785400 -0.00294800 -0.46112500

H 4.40100800 -1.74088300 1.10392100

H 3.60325200 -0.43499900 1.97899500

H 2.72595600 -1.93631800 1.62293700

H 3.96422500 -2.17609600 -1.32301400

H 2.61048600 -1.43474000 -2.17561900

H 2.30828600 -2.61375600 -0.89417700

H -2.14823300 2.35997300 -1.56062100

H -1.99810900 0.64031700 -1.75316500

H -3.72380600 1.58005000 -0.06329000

H -2.38756800 1.90750600 1.02326300

H -1.20248400 0.13049100 2.03643200

H -2.13588100 -2.76417700 1.45823300

H -1.13627000 -2.23830000 2.80390600

H -0.25999200 -0.92106100 -2.31441400

H 0.67679900 -1.55672900 1.30697900

H 0.31397700 -3.26688400 1.11249600

H -4.73269300 -0.70052800 0.11298500

H -3.57346200 -1.03457900 -1.16354000

H -3.68346700 -2.11215600 0.23734700

H -1.11343100 -3.02233500 -2.33098900

H -0.23102300 -4.10962800 -1.24243000

H -1.83406600 -3.54786700 -0.79866600

**Structure T^+^**

C 2.79530400 -1.50602400 0.39693900

C 1.72856500 -1.53346500 1.51754800

C 0.40719000 -0.69812200 1.27087400

C 0.08950000 -0.88395400 -0.19582200

C 1.13281600 -0.63548500 -1.26057100

C 2.56160100 -0.44753600 -0.69708100

C 3.63817300 -0.56833000 -1.78630900

C 0.41696400 0.71434400 1.97838300

C 0.12342600 0.49607800 3.47669400

C 1.66470000 1.59605900 1.83401400

C 0.70176400 0.43484100 -2.34045900

C 0.34514700 1.85473100 -1.86906100

C -0.89609900 1.99696000 -1.00479400

C -1.87143100 1.06109900 -1.08971200

C -3.05962400 0.80536000 -0.22669400

C -1.02609800 -1.69854000 -0.56204900

C -2.32164000 -1.64277700 -0.08791800

C -2.83762000 -0.47955200 0.69761800

C -0.97419700 3.23776000 -0.15603000

C -3.33528500 -2.63354400 -0.56937400

H 1.15343500 -1.56905400 -1.84704300

H 3.78605000 -1.35493800 0.83680500

H 2.83138500 -2.48805200 -0.09251000

H 1.43079300 -2.56785900 1.70895400

H 2.15125400 -1.16123000 2.45436200

H -0.37499500 -1.24111100 1.80286400

H 2.64873700 0.54601500 -0.25333400

H 4.63427500 -0.53022600 -1.33452600

H 3.59036300 0.24012200 -2.52091300

H 3.55730900 -1.52163000 -2.32280500

H -0.43106800 1.26334700 1.55799800

H 0.02523000 1.46048800 3.98389600

H -0.80651800 -0.05968600 3.63895900

H 0.93019100 -0.05139700 3.97596100

H 1.55298500 2.47956900 2.47054400

H 1.82473000 1.95412500 0.81602100

H 2.57367100 1.08168200 2.16211000

H 1.53578600 0.50561300 -3.04269200

H -0.12440200 0.01482400 -2.92109300

H 0.22727200 2.47529400 -2.77080200

H 1.19329000 2.30132800 -1.33684800

H -1.75004500 0.32394700 -1.87292700

H -3.29432000 1.63278300 0.44645400

H -3.94500100 0.62184200 -0.84531500

H -0.85831800 -2.36718100 -1.40713300

H -2.13956200 -0.17975800 1.47961600

H -3.79345200 -0.72361000 1.16851700

H -1.05222900 4.11212000 -0.81610100

H -1.84339800 3.25333300 0.50432200

H -0.07199400 3.38704500 0.44474100

H -2.91303500 -3.38379900 -1.24056700

H -3.78086200 -3.14661300 0.29332600

H -4.16650900 -2.12673400 -1.07801400

**Structure TS^T_L^**

C 3.13430300 0.99447700 -0.46929800

C 3.03366400 -0.44560400 0.02002800

C 1.74741300 -1.17577000 -0.43497700

C 0.59811900 -0.21711400 -0.88421000

C 0.63105800 1.20656200 -0.63098900

C 1.89696500 1.82802500 -0.07011000

C 2.11087800 3.31672900 -0.39385700

C 1.29598800 -2.26887100 0.58783700

C 2.29232400 -3.44245400 0.57328900

C 1.09700400 -1.75557200 2.02532700

C -0.47778100 2.10929900 -1.09066100

C -1.31706500 2.73997200 0.08072500

C -2.00448800 1.66599300 0.90531400

C -2.88413000 0.86148200 0.28982300

C -3.30822000 -0.50813900 0.73838600

C -0.58745900 -0.89902000 -1.54902000

C -1.70699000 -1.46082900 -1.04636200

C -2.13751600 -1.48613000 0.40778300

C -1.55012400 1.52477700 2.33658700

C -2.65605500 -2.15628700 -1.99562800

H 1.09096000 0.37042800 -1.80517200

H 4.02406800 1.46964700 -0.04681300

H 3.25280300 1.04245500 -1.56237000

H 3.90611300 -1.01319800 -0.31526800

H 3.08062800 -0.42966700 1.11266200

H 1.97165400 -1.73223100 -1.35504600

H 1.77389800 1.72774500 1.02272500

H 3.01996000 3.65631300 0.10900300

H 1.29499200 3.95630000 -0.05332700

H 2.25337200 3.47714500 -1.46787900

H 0.34120200 -2.66153500 0.22667100

H 1.94546800 -4.23663900 1.24159600

H 2.39451900 -3.87308100 -0.42852200

H 3.28702500 -3.14011400 0.91805800

H 0.54379500 -2.49671700 2.61044000

H 0.53371100 -0.81731300 2.07225200

H 2.04989500 -1.59568100 2.53956300

H -0.03988900 2.92653100 -1.67276900

H -1.16272200 1.55052100 -1.72885100

H -2.03938100 3.40408500 -0.40599800

H -0.68191100 3.37169100 0.70754600

H -3.15748000 1.11039000 -0.73586700

H -3.50525800 -0.56470200 1.81310300

H -4.22717000 -0.81913900 0.23300200

H -0.43471000 -1.02251300 -2.62121100

H -1.30473200 -1.22400600 1.06046300

H -2.44084900 -2.51055700 0.66097500

H -1.75454200 2.45140100 2.88789000

H -2.05271500 0.71152100 2.86303300

H -0.46676400 1.35723000 2.41385700

H -2.32702600 -2.09606200 -3.03555200

H -2.75466000 -3.21492100 -1.72676600

H -3.66157400 -1.72447800 -1.93134800

**Structure L+**

C 1.73961100 2.45399900 0.79326200

C 2.53586400 1.15722000 0.70521500

C 2.03824300 0.27917700 -0.44630500

C 0.45228000 -0.02597000 -0.26833700

C -0.12518600 1.33668700 -0.22398200

C 0.16858300 2.18517600 0.94358900

C -0.59905500 3.49930000 1.11072800

C 2.84072000 -1.02912300 -0.72229800

C 4.02734100 -0.72124400 -1.65534800

C 3.32800200 -1.75670800 0.54193000

C -0.83542900 1.84723000 -1.40874300

C -2.42094000 1.64531600 -1.25519300

C -2.82705100 0.31286000 -0.64631800

C -2.56851500 -0.82445600 -1.31088900

C -2.60808300 -2.23140600 -0.78640600

C 0.08787600 -0.91102900 0.90853200

C -0.61237900 -2.06088300 0.84861000

C -1.16808300 -2.70500200 -0.41141500

C -3.44051900 0.39817300 0.73022500

C -0.89537100 -2.82717500 2.11993900

H 0.14498500 -0.50495000 -1.19767200

H 1.90541600 3.08136100 -0.09037800

H 2.01358200 3.04879500 1.67020300

H 2.49029500 0.61937000 1.65769100

H 3.59149100 1.40307900 0.53859700

H 2.10650200 0.87974200 -1.36485100

H 0.07867000 1.57921800 1.85227400

H -0.44772600 4.18321300 0.27027800

H -0.24389300 4.00762900 2.01097800

H -1.67164400 3.33033900 1.23289700

H 2.16921600 -1.70931400 -1.26294400

H 4.57905200 -1.63897200 -1.88100400

H 3.69701900 -0.28966700 -2.60635000

H 4.73337300 -0.02268300 -1.19156100

H 3.79758600 -2.70375300 0.25832200

H 2.51500600 -1.98698700 1.23403300

H 4.08323600 -1.17076300 1.07679800

H -0.51978200 1.29428600 -2.29693100

H -0.66714600 2.91755000 -1.55763000

H -2.80445000 1.76585500 -2.27292500

H -2.80886300 2.47493400 -0.66139000

H -2.15769700 -0.73492600 -2.31960200

H -3.26650300 -2.33018600 0.08106300

H -2.99522300 -2.90889400 -1.55563700

H 0.42641700 -0.57836200 1.88661900

H -0.50977000 -2.53615700 -1.27094300

H -1.19333100 -3.78955800 -0.25885000

H -4.35017700 1.01109500 0.69979700

H -3.71079400 -0.57697400 1.13588500

H -2.76338700 0.87902000 1.44915800

H -0.56042600 -2.29415500 3.01340200

H -1.96915500 -3.02670400 2.22319800

H -0.39933300 -3.80533400 2.09668000

**Structure TS^L_E^**

C 2.03468500 2.13849800 1.12528600

C 2.61894000 0.73096300 0.91534200

C 2.10892700 0.08933100 -0.38230500

C 0.54525500 0.03372900 -0.35319400

C 0.01511700 1.48444900 -0.31529900

C 0.71674900 2.44933000 0.45327600

C 0.29623100 3.90012600 0.50360800

C 2.78551300 -1.26669900 -0.74645600

C 4.08557500 -1.01716700 -1.53290900

C 3.06228100 -2.19984000 0.44580500

C -1.09550100 1.93069000 -1.26757100

C -2.56269100 1.82448000 -0.77676600

C -2.98925300 0.41415000 -0.38248700

C -2.72282200 -0.61577600 -1.20040700

C -2.75755900 -2.08027300 -0.87951700

C -0.02465800 -0.80093000 0.78614900

C -0.77148300 -1.92086600 0.72632200

C -1.30645600 -2.55569300 -0.54011200

C -3.67641100 0.30532100 0.95576100

C -1.15900900 -2.63109700 2.00245000

H 0.19829900 -0.37839600 -1.30234400

H 2.70346700 2.89430000 0.68537800

H 1.97857800 2.40831500 2.18661600

H 2.37766100 0.09536400 1.77109500

H 3.70981400 0.81217700 0.89806400

H 2.35433600 0.77699300 -1.20748900

H -0.30059400 1.71445900 0.93112800

H 0.80725200 4.43050700 -0.30981600

H 0.61220600 4.35838200 1.44358000

H -0.77613100 4.05053600 0.37994700

H 2.09404100 -1.78955800 -1.42255100

H 4.54859700 -1.96523200 -1.82313200

H 3.90273600 -0.44302700 -2.44795900

H 4.82039100 -0.46899200 -0.93091600

H 3.43390500 -3.16215300 0.08017800

H 2.16844700 -2.39563900 1.04259500

H 3.83423400 -1.78983100 1.10663000

H -0.97329900 1.31144000 -2.16030600

H -0.90242200 2.96081300 -1.57978400

H -3.19805400 2.22431300 -1.57682800

H -2.70365000 2.49969900 0.07648700

H -2.27288200 -0.39275100 -2.16964100

H -3.42563600 -2.30836300 -0.04470100

H -3.11219300 -2.65965100 -1.73902900

H 0.25942600 -0.45815800 1.78206700

H -0.66083900 -2.34406000 -1.39919700

H -1.31502100 -3.64494400 -0.41904200

H -4.54862400 0.97088300 0.98682200

H -4.02606800 -0.70403000 1.17552300

H -3.01490400 0.61793000 1.77532500

H -0.84437900 -2.08585400 2.89616000

H -2.24361900 -2.78209000 2.05850100

H -0.70566300 -3.62973300 2.03478800

**Structure TS^G_H^**

C 1.64101300 -0.48494700 0.95020400

C 2.68244000 -1.25228600 0.10582700

C 4.07663000 -0.72850700 0.34219000

C -2.24693700 -0.35301400 1.44653600

C -0.85714400 -0.05658500 1.44090600

C -0.03482900 -2.41246100 0.92513300

C 4.92398000 -0.17880200 -0.54604400

C 6.30069600 0.27145100 -0.11711600

C 4.63218100 0.02770800 -2.01286100

C -0.49731800 1.25782300 2.11532800

C -1.18667200 2.50219900 1.39983400

C -1.45148800 2.26797900 -0.06980500

C -2.65937800 1.79116600 -0.43403500

C -3.02181700 1.11806700 -1.72914600

C -3.09597200 -1.25945700 0.65901500

C -3.30775700 -1.25277800 -0.68259900

C -2.60012500 -0.39154900 -1.71214200

C -0.28689800 2.47078600 -1.00703000

C -4.40394900 -2.12308400 -1.24089300

H 1.77535600 0.57898800 0.73184400

H 1.87192000 -0.62003600 2.01578500

H 2.66024500 -2.31722500 0.36926000

H 2.40836400 -1.18747500 -0.95330600

H 4.42711000 -0.83112000 1.37041900

H -2.81505800 0.28610000 2.12799300

H -1.46454300 -0.91037700 2.32151000

H -1.06511300 -2.73035900 0.75812500

H 0.59232000 -2.99091300 0.24610000

H 0.25733700 -2.67526000 1.94873000

H 6.47380100 0.10477500 0.94943900

H 7.07733700 -0.26543300 -0.67655100

H 6.44703400 1.33884400 -0.32651600

H 3.62596800 -0.27567600 -2.30985900

H 4.76228700 1.08304600 -2.28409900

H 5.34495000 -0.53830200 -2.62586800

H -0.83941100 1.24710300 3.15619300

H 0.58181400 1.38004500 2.14783100

H -2.12881200 2.71098700 1.91416100

H -0.52802300 3.36024800 1.56835100

H -3.43836700 1.77632200 0.32718800

H -2.52444300 1.57992200 -2.58719300

H -4.09840200 1.19405800 -1.91212300

H -3.78670100 -1.83988600 1.26811700

H -1.51614200 -0.43262100 -1.57401400

H -2.80325500 -0.81059600 -2.70272700

H 0.01096100 3.52671600 -0.99826000

H 0.59962100 1.90330200 -0.69806800

H -0.51637400 2.20174300 -2.03939900

H -4.94171200 -2.67777600 -0.46925600

H -5.12552000 -1.51604400 -1.80160400

H -3.98247600 -2.84019500 -1.95658200

C 0.17489900 -0.89907100 0.67955800

H -0.03140000 -0.70072000 -0.38277700 **Structure H^+^**

C -2.24226500 2.03486300 -0.89498700

C -3.08605900 0.73794000 -0.97260400

C -3.20486100 -0.04845400 0.31343100

C 0.10243900 -0.17500000 -0.33699000

C 0.26953100 1.29143400 -0.23663100

C -0.74287700 1.97718300 -1.27036600

C -0.21529300 3.39920600 -1.55280400

C -3.36801100 -1.37696500 0.46800900

C -3.40604700 -2.37084000 -0.66885400

C -3.59051800 -1.97789900 1.83626400

C 0.01636300 1.74327900 1.23346800

C 1.26080500 1.62526600 2.16872300

C 2.05368600 0.36756900 1.90020700

C 3.04731000 0.46790800 0.98776000

C 3.75683500 -0.58355300 0.21892100

C 0.76540400 -1.24612000 -0.94133800

C 2.06022000 -1.44268000 -1.44121000

C 3.22345700 -0.53638400 -1.29522400

C 1.65244100 -0.88820800 2.63038000

C 2.36013000 -2.76389500 -2.07322000

H 1.26391600 1.60403900 -0.55605500

H -0.85753900 -0.48984600 0.08168900

H -2.68019200 2.74420100 -1.60606100

H -2.36940400 2.50233800 0.08931600

H -2.72734600 0.11215000 -1.79923800

H -4.10190100 1.03970900 -1.26493700

H -3.25857800 0.56465100 1.21514600

H -0.65834600 1.41131100 -2.20823500

H -0.81741800 3.86925500 -2.33596400

H 0.82382200 3.38689600 -1.89653800

H -0.27316300 4.03950200 -0.66621800

H -3.12846900 -1.94223400 -1.63452800

H -2.75002400 -3.22699300 -0.46425500

H -4.41779400 -2.78331100 -0.77480100

H -3.56590400 -1.22252300 2.62658400

H -4.56399700 -2.48237400 1.88287400

H -2.83661100 -2.74324800 2.06385200

H -0.31796700 2.78455200 1.23905700

H -0.80865100 1.15495700 1.64886300

H 0.91040500 1.67142500 3.20591000

H 1.90044300 2.49860300 2.00621000

H 3.27483100 1.47428600 0.63389600

H 3.64537800 -1.58931800 0.63129900

H 4.82902300 -0.37249700 0.15146700

H 0.14916900 -2.14243900 -0.98305000

H 2.98708100 0.50181200 -1.53430100

H 4.02772700 -0.86445200 -1.95765400

H 1.96568200 -0.80880400 3.67922300

H 0.56570600 -1.02550000 2.64268100

H 2.11004700 -1.79119000 2.21992800

H 1.53463400 -3.47416600 -2.01457500

H 2.60994000 -2.59939600 -3.13150100

H 3.25936100 -3.20928200 -1.62769300

**Structure TS^H_I^**

C -1.98908400 2.32976100 -0.95513700

C -2.85798900 1.09374100 -0.71370900

C -2.41150700 0.33452200 0.51563600

C -0.37866900 -0.17082500 0.26876000

C 0.19721600 1.18390500 -0.11816700

C -0.53946400 1.95241800 -1.26580300

C 0.27785300 3.17052700 -1.72196700

C -2.88347400 -0.92741600 0.85685800

C -3.57904800 -1.79933000 -0.13562800

C -2.71972500 -1.46384100 2.23971100

C 0.58389200 1.98121500 1.15684500

C 2.09211300 1.80034200 1.58014500

C 2.58300600 0.38383200 1.35064100

C 3.08392300 0.10175900 0.13278800

C 3.20608900 -1.19915300 -0.60341200

C -0.19523700 -1.42570900 -0.46712200

C 0.81009200 -1.91159900 -1.25427400

C 2.09093900 -1.25151900 -1.72191000

C 2.34181200 -0.62057600 2.44831600

C 0.74442300 -3.37320900 -1.63352600

H 1.13186900 0.90484300 -0.60603700

H -0.35711400 -0.35447100 1.34047500

H -2.39811000 2.89380800 -1.80060300

H -2.03617500 3.00056400 -0.08603200

H -2.84597400 0.45174200 -1.60229600

H -3.90362400 1.39502300 -0.56190000

H -2.16136900 0.97237600 1.36464000

H -0.57002000 1.24892800 -2.11171100

H -0.15716900 3.59598200 -2.63139100

H 1.31541100 2.90123500 -1.94687900

H 0.29273400 3.96403400 -0.96794400

H -3.11440900 -1.74786800 -1.12571400

H -3.63412400 -2.84007400 0.19139200

H -4.60994600 -1.43608800 -0.26490400

H -2.22715000 -0.76512300 2.91880700

H -3.71061800 -1.70332200 2.65043000

H -2.17094400 -2.41534600 2.22733300

H 0.40225700 3.05092600 1.02290600

H -0.05571400 1.67732800 1.99509900

H 2.19350900 2.10918000 2.62632700

H 2.69546000 2.49242200 0.98375200

H 3.28046900 0.96020400 -0.51384900

H 3.12441600 -2.07212100 0.05095800

H 4.17663400 -1.26614000 -1.10744700

H -0.89605400 -2.19665000 -0.15756000

H 1.92331400 -0.24279900 -2.10886500

H 2.47452300 -1.83698300 -2.56281400

H 2.99146900 -0.39384800 3.30317000

H 1.31407700 -0.57609200 2.83349300

H 2.54922200 -1.64822800 2.14164200

H -0.11749300 -3.89099000 -1.20695200

H 0.70201100 -3.47037800 -2.72590300

H 1.65567600 -3.89514900 -1.31590200

**Structure I^+^**

C -3.25807600 -0.11228600 -0.12193500

C -2.57160700 -1.36851200 0.42595100

C -1.24378600 -0.99035800 1.09927700

C -0.19504000 -0.24383100 0.01582600

C -0.98723200 1.01029800 -0.53853500

C -2.36579000 0.61651600 -1.13775100

C -3.09585600 1.79412100 -1.80045400

C -0.43617300 -2.01853800 1.70521600

C -0.37356100 -3.37777200 1.15177700

C 0.44886300 -1.69353800 2.84447100

C -0.98839300 2.20534800 0.44871300

C 0.28600700 3.10118100 0.34332700

C 1.53106800 2.30004900 0.65448500

C 2.17307200 1.65497500 -0.33594900

C 3.09705300 0.47181900 -0.20830900

C 0.30080800 -1.13995100 -1.07641100

C 1.53860000 -1.27027600 -1.61533600

C 2.87064100 -0.58726000 -1.31115100

C 1.85130900 2.14214600 2.12114600

C 1.68677200 -2.20415900 -2.80381200

H -0.37458400 1.31599700 -1.39509600

H 0.62589900 0.11438900 0.62431800

H -4.20212600 -0.39788700 -0.59900600

H -3.52308200 0.55925600 0.70660500

H -2.41098000 -2.08494600 -0.38697200

H -3.21420400 -1.86225300 1.16458500

H -1.40256700 -0.18441900 1.82473300

H -2.14882500 -0.10284600 -1.94062300

H -3.94515800 1.42757200 -2.38563900

H -2.43697700 2.34186300 -2.48286000

H -3.49117300 2.50590300 -1.06881800

H 0.22291000 -3.26721800 0.22033700

H 0.14013900 -4.08809900 1.80141900

H -1.34768200 -3.75937300 0.83537100

H 0.54004800 -0.62440300 3.03933400

H 0.00007100 -2.16680800 3.73492600

H 1.43517600 -2.16028500 2.73656600

H -1.85646600 2.84105900 0.25350000

H -1.10686500 1.86672400 1.48663300

H 0.17174700 3.94949000 1.02849000

H 0.33851100 3.51184900 -0.67132600

H 1.89647300 1.90792900 -1.36037900

H 3.00920000 0.01315600 0.78381900

H 4.14979100 0.77345300 -0.29927500

H -0.47908000 -1.70149200 -1.58934300

H 3.17640500 -0.13520500 -2.26651300

H 3.60231700 -1.39262000 -1.15584700

H 2.19164400 3.10388900 2.52526100

H 0.96381100 1.86928000 2.70950300

H 2.64187100 1.41489600 2.31975200

H 0.76001600 -2.73463700 -3.03509100

H 1.98890900 -1.64682200 -3.69879600

H 2.47276300 -2.94722800 -2.62283200

**Structure TS^I_J^**

C -2.85288000 1.40749600 -0.32714200

C -2.86557200 -0.11364600 -0.52537500

C -1.64410500 -0.81530000 0.03917100

C -0.32018700 1.41943700 -0.07359000

C -1.53785100 2.02148800 -0.82227500

C -1.57403600 3.55589300 -0.78987000

C -0.14958200 1.99109000 1.37320400

C 0.67464800 1.19387400 2.42293200

C 1.90341100 0.44164300 1.93276000

C 2.51829900 0.79288400 0.79211200

C 3.36073100 -0.05541800 -0.11476700

C 0.26382000 -0.65768200 -1.52492800

C 1.51076400 -0.64110300 -2.04955300

C 2.83633600 -0.01869700 -1.57743200

C 2.27003700 -0.77530400 2.74909100

C 1.67458500 -1.23481000 -3.43862900

H 0.55104600 1.73962000 -0.64541100

H -3.70021700 1.83223600 -0.87560900

H -3.02193000 1.64739900 0.73096600

H -2.82907700 -0.33459600 -1.60360300

H -3.79666400 -0.55008700 -0.16120200

H -1.78686600 -1.00459000 1.29728500

H -1.42453900 1.72468800 -1.87609600

H -2.36001300 3.93236400 -1.45231500

H -0.62408400 3.98174000 -1.13001400

H -1.77629500 3.94375600 0.21353400

H 0.29366000 2.98564600 1.25828300

H -1.13246900 2.17235300 1.82560300

H 0.01851200 0.43921100 2.88571300

H 0.92076600 1.87219400 3.25159500

H 2.25514100 1.75486200 0.35894900

H 3.41912200 -1.09049200 0.23690200

H 4.39407300 0.31489500 -0.16235500

H -0.48995700 -1.10834900 -2.17097600

H 2.81433700 1.03260900 -1.90421800

H 3.61728500 -0.47851100 -2.19205100

H 2.49954300 -0.47044400 3.77828000

H 1.44126500 -1.49365900 2.82754800

H 3.14765700 -1.29902200 2.36541800

H 0.73139900 -1.59162900 -3.85988600

H 2.09463200 -0.49171200 -4.12788000

H 2.38189100 -2.07309700 -3.41670400

C -1.73403400 -2.15205900 0.52056800

C -3.04849200 -2.88790100 0.61959200

H -3.26716300 -3.31387700 -0.36861600

H -2.97729500 -3.71249600 1.33071200

H -3.89311200 -2.25584400 0.89382200

C -0.52891000 -2.97956700 0.86613800

H 0.42120400 -2.46094300 0.77669700

H -0.63070600 -3.42239400 1.86144000

H -0.52510000 -3.81513700 0.15330000

C -0.28319500 -0.14473800 -0.18688600

H 0.39123600 -0.49218200 0.59015900

**Structure J^+^**

C -3.00803800 0.71343400 -0.47885100

C -2.43622800 0.03316700 0.80749000

C -1.21418900 -0.77325000 0.56892500

C -0.54351500 1.11556100 -1.00003200

C -1.96176200 0.89527000 -1.59516000

C -2.38511600 1.97146100 -2.60264500

C -0.40432600 2.47563700 -0.21472000

C 0.25302300 2.51872100 1.18928100

C 1.53254000 1.72763300 1.39146800

C 2.27541000 1.34739100 0.32984400

C 3.31713900 0.27280400 0.26049700

C 0.61460000 -1.22339900 -1.07767400

C 1.92432700 -1.36731100 -1.38559900

C 3.17615000 -0.56520300 -1.02839200

C 1.82985200 1.33116200 2.81457000

C 2.27784900 -2.48161500 -2.35458600

H 0.14722900 1.16281200 -1.84523600

H -3.83834900 0.10561500 -0.85125500

H -3.43782600 1.67734900 -0.18972900

H -3.19934300 -0.53938500 1.33785900

H -2.12973100 0.82925100 1.50330900

H -1.89793500 -0.05487700 -2.14704700

H -3.36155600 1.73108900 -3.03545000

H -1.66707700 2.04314300 -3.42595000

H -2.46769900 2.95948600 -2.13804500

H 0.13676200 3.16494200 -0.87041300

H -1.39504800 2.92675000 -0.09561900

H -0.45927300 2.16156300 1.94650600

H 0.41273200 3.57401800 1.45100400

H 2.01222500 1.76884700 -0.63880500

H 3.27879900 -0.37509800 1.14240500

H 4.32740900 0.70482800 0.23961800

H -0.04316300 -1.94212500 -1.56176000

H 3.35898800 0.09646100 -1.88962800

H 4.01312600 -1.27256400 -1.04687100

H 1.86246000 2.22687600 3.44710900

H 1.03757600 0.69366300 3.23275900

H 2.78446600 0.81471400 2.92552700

H 1.39706000 -2.99896500 -2.74138200

H 2.84304300 -2.08673000 -3.20752400

H 2.92499000 -3.22107000 -1.86688000

C -1.31983600 -2.11759000 2.71290000

H -2.34620100 -1.84020500 2.96830400

H -1.14364900 -3.12603300 3.09767800

H -0.63600100 -1.43810900 3.23044100

C -2.05585000 -3.10093900 0.47582400

H -1.88914000 -4.10199800 0.88331800

H -3.09799000 -2.82697200 0.66177000

H -1.89259300 -3.14641600 -0.60394500

C -1.07067800 -2.12280500 1.18677400

H -0.05437300 -2.48018700 0.99278300

C -0.15753200 -0.20213700 -0.24630100

H 0.55716900 0.12137400 0.57348600

**Structure TS^J_K^**

C -2.66004000 1.59490400 -0.58388900

C -2.57611200 0.49839400 0.51844000

C -1.58505400 -0.58510400 0.19999200

C -0.12223000 1.34234600 -0.49058300

C -1.33740000 1.79623400 -1.35529200

C -1.19348800 3.22679100 -1.88981800

C 0.05358500 2.22473600 0.79972700

C 0.61719600 1.55083900 2.07548400

C 1.79473000 0.61753500 1.83264000

C 2.63461700 0.88922300 0.82169300

C 3.57113300 -0.01392500 0.08853400

C 0.55048700 -1.19666100 -0.98714300

C 1.83891200 -1.25065100 -1.42294700

C 2.97284500 -0.24276800 -1.33797900

C 1.88216400 -0.59522500 2.72512300

C 2.26059300 -2.52147200 -2.12784400

H 0.77029300 1.46298100 -1.10226200

H -3.45676200 1.33392900 -1.28705400

H -2.97055300 2.52836100 -0.10401300

H -3.55401900 0.05132500 0.70470300

H -2.28920100 0.96030700 1.47098900

H -1.34667200 1.12491200 -2.22563300

H -2.03774200 3.47751300 -2.53985600

H -0.27819700 3.33947600 -2.48022400

H -1.17139700 3.96458200 -1.08122800

H 0.70374900 3.05723500 0.51622200

H -0.89784500 2.69577100 1.06730500

H -0.17699100 0.97265300 2.57353500

H 0.86594600 2.33731100 2.80102600

H 2.51260900 1.84934000 0.32237600

H 3.73111600 -0.97158700 0.59160400

H 4.55379000 0.45431900 -0.04196500

H -0.00642000 -2.10067000 -1.20858200

H 2.66120400 0.72762800 -1.74039000

H 3.77391600 -0.59447000 -1.99457300

H 1.94477900 -0.28130300 3.77483800

H 0.98806900 -1.23277200 2.65343000

H 2.75510700 -1.21561400 2.51567700

H 1.47506700 -3.27982500 -2.15735100

H 2.56687700 -2.29944000 -3.15758600

H 3.14011200 -2.95164100 -1.63204600

C -2.66779300 -2.30513200 1.74683300

H -3.62978400 -1.79550200 1.84883100

H -2.85663300 -3.37876300 1.83352800

H -2.03077000 -2.01210800 2.58752300

C -2.90953100 -2.41969600 -0.79578800

H -3.16231700 -3.47985300 -0.70594600

H -3.84467800 -1.85200800 -0.78313400

H -2.42564200 -2.26808100 -1.76518900

C -1.98810400 -2.02772600 0.39095600

H -1.09253500 -2.65223600 0.33909500

C -0.29263400 -0.17814200 -0.29114700

H -0.27773400 -0.39876900 0.90439400

**Structure K^+^**

C -2.56435400 1.77518000 -0.66414800

C -2.73660300 0.25644200 -0.67299900

C -0.04914200 1.44318900 -0.20420200

C -1.14554600 2.18545700 -1.07433700

C -0.93533600 3.70527700 -1.06992300

C -0.00041600 1.98380900 1.27157700

C 0.61194900 1.05424400 2.35576700

C 1.74643100 0.17238500 1.86626300

C 2.62576200 0.68083900 0.97803400

C 3.52287400 -0.03965100 0.03946300

C 0.47860700 -0.96864100 -1.04220300

C 1.77175800 -0.97645000 -1.57417800

C 2.86707000 0.02276500 -1.41686400

C 1.79106300 -1.23191000 2.41119400

C 2.18028400 -2.20710400 -2.32790700

H 0.90284300 1.67751900 -0.67395100

H -3.28198500 2.22685000 -1.35913300

H -2.80756000 2.17597700 0.32792200

H -2.68504400 -0.12346200 -1.69933300

H -3.71627500 -0.02600200 -0.27587300

H -0.98899500 1.83636400 -2.10541600

H -1.60720100 4.17316200 -1.79621400

H 0.08926500 3.97315300 -1.34997600

H -1.14865600 4.14736100 -0.09204100

H 0.56950000 2.91621600 1.22265900

H -1.00205700 2.27140300 1.60331300

H -0.16808400 0.40457300 2.76915300

H 0.93398200 1.67992900 3.19912000

H 2.55269400 1.74590800 0.76275100

H 3.71259400 -1.07947600 0.31565900

H 4.49231100 0.45896400 -0.06228700

H -0.02690200 -1.91589600 -1.18811500

H 2.53550400 1.04851000 -1.58803200

H 3.65186200 -0.19622000 -2.14557900

H 1.92916200 -1.19335000 3.49955800

H 0.85102500 -1.76874100 2.23923700

H 2.60994700 -1.82713900 2.00255200

H 1.43205800 -3.00047300 -2.30929000

H 2.37980000 -1.93659500 -3.37440100

H 3.13039800 -2.59642300 -1.93919200

C -2.85918800 -2.17624900 1.61793600

H -3.83050900 -1.69606700 1.45337700

H -3.04775300 -3.23995900 1.79142400

H -2.42878700 -1.76167400 2.53595700

C -2.49726400 -2.72451800 -0.81308700

H -1.91718900 -2.57880400 -1.73017100

H -2.52913500 -3.79958900 -0.61216100

H -3.52277300 -2.40613700 -1.02463300

C -1.92141300 -1.98514300 0.41091100

H -0.96681400 -2.45941900 0.67597000

C -0.30341100 -0.02974500 -0.33489100

C -1.64489100 -0.46391300 0.20031500

H -1.72663400 0.01194600 1.18681500

**Structure TS^K_L^**

C -1.52166300 2.47317800 -0.67547100

C -2.30082500 1.12921500 -0.70902600

C 0.23936900 1.29827200 0.21436000

C 0.08490300 2.39079100 -0.81113100

C 0.76699800 3.73895600 -0.60423100

C 0.36044900 1.65943200 1.69622500

C 0.71540900 0.50571200 2.70027200

C 1.54289900 -0.63967000 2.11524300

C 2.48434700 -0.36784200 1.18399500

C 3.07923600 -1.25576000 0.12365700

C 0.11991900 -0.58927400 -1.50045800

C 1.36289400 -0.96942700 -1.91661800

C 2.74051900 -0.68757300 -1.29546800

C 1.07811800 -2.02626600 2.46445400

C 1.45658000 -1.77770400 -3.18852400

H 0.94156400 0.06635700 0.37501700

H -1.86823100 3.09630000 -1.50807600

H -1.75330500 3.02254500 0.24300600

H -2.35717500 0.76802800 -1.73933500

H -3.33153300 1.31932000 -0.38848600

H 0.29984700 2.00746900 -1.81144300

H 0.46674000 4.43313500 -1.39479900

H 1.85663100 3.64309600 -0.65025500

H 0.50262300 4.19916700 0.35243400

H 1.14510000 2.42340400 1.74262600

H -0.54087500 2.16370600 2.06302400

H -0.20714000 0.07610800 3.10394000

H 1.22356000 0.96371400 3.55698200

H 2.78373700 0.67753000 1.07281000

H 2.73832300 -2.29093000 0.21023700

H 4.17280000 -1.26717700 0.20553000

H -0.70292000 -0.96897600 -2.09786200

H 2.93051600 0.39378200 -1.29052700

H 3.47853600 -1.10396100 -1.98636800

H 1.00935500 -2.12902100 3.55413800

H 0.06935600 -2.21848000 2.07213400

H 1.74708100 -2.80606000 2.09753100

H 0.48078200 -1.98956000 -3.63065100

H 2.06435800 -1.24632600 -3.93236700

H 1.96791000 -2.73045200 -2.99945800

C -3.43919200 -1.54690500 1.35716300

H -4.21349500 -0.77050600 1.33535000

H -3.94754000 -2.51554900 1.35019800

H -2.90313200 -1.46104400 2.30898100

C -3.26594500 -1.69172400 -1.14818600

H -2.64766100 -1.63604300 -2.04881400

H -3.68682100 -2.70091200 -1.11266700

H -4.10285000 -0.99651100 -1.27186900

C -2.48811600 -1.41787400 0.15261000

H -1.72257500 -2.20008600 0.24837400

C -0.29725300 0.07651300 -0.25867500

C -1.73466400 -0.05779000 0.22044500

H -1.79745400 0.31238000 1.24684200
