# Supplementary material for: Discovery of a terpene synthase synthesizing a nearly non-flexible eunicellane reveals the basis of flexibility
Source: Nat Commun. 2024 Jul 15;15:5940. doi: 10.1038/s41467-024-50209-z (PMC11250809; doi:10.1038/s41467-024-50209-z)
Supplement: Supplementary file 6 — Supplementary Data 3 [file 41467_2024_50209_MOESM6_ESM.docx]

**Supplementary data 3. The global score and each residue score of MicA predicted models.** The term “Af_MicA.pdb” represents model of MicA, which was predicted using AlphaFold computational methods. Similarly, “Tf_MicA.pdb” represents the MicA model predicted using tFold, while “Rf_MicAl.pdb” represents the MicA model predicted using RoseTTAFold. This document provides supplementary data corresponding to Figure 5a in the main text, Supplementary Fig.s 31 and 32.

| **number** | **name** | **Af_MicA.pdb** | **Tf_MicA.pdb** | **Rf_MicAl.pdb** |
| --- | --- | --- | --- | --- |
| 0 | global | 0.54678995 | 0.568838231 | 0.517744822 |
| 1 | M | 0.19997458 | 0.2175363 | 0.17958489 |
| 2 | T | 0.40520343 | 0.4137286 | 0.32067555 |
| 3 | F | 0.44226214 | 0.4799557 | 0.36601382 |
| 4 | T | 0.5576008 | 0.5763523 | 0.4888357 |
| 5 | V | 0.4657952 | 0.492053 | 0.4553723 |
| 6 | P | 0.53533095 | 0.56809825 | 0.5188886 |
| 7 | D | 0.5216396 | 0.56059545 | 0.5202811 |
| 8 | L | 0.5498682 | 0.55150884 | 0.36370552 |
| 9 | S | 0.5817129 | 0.5954651 | 0.47724387 |
| 10 | V | 0.59177 | 0.58754927 | 0.24550112 |
| 11 | P | 0.59388995 | 0.593965 | 0.16507593 |
| 12 | F | 0.5582202 | 0.5558259 | 0.01716833 |
| 13 | P | 0.5492584 | 0.5861108 | 0.35735705 |
| 14 | E | 0.52612394 | 0.5350506 | 0.4411743 |
| 15 | R | 0.5289207 | 0.5524253 | 0.48829392 |
| 16 | Q | 0.583376 | 0.590837 | 0.5382697 |
| 17 | N | 0.64368665 | 0.65039283 | 0.58594173 |
| 18 | P | 0.5847162 | 0.5980775 | 0.5604233 |
| 19 | H | 0.65132654 | 0.65704364 | 0.61281216 |
| 20 | V | 0.5986107 | 0.60739124 | 0.6051712 |
| 21 | D | 0.6249014 | 0.62588024 | 0.622031 |
| 22 | E | 0.6001982 | 0.6299458 | 0.6265759 |
| 23 | A | 0.64139974 | 0.6504333 | 0.65212476 |
| 24 | E | 0.5956051 | 0.6194403 | 0.6097406 |
| 25 | V | 0.64655864 | 0.6584468 | 0.6580644 |
| 26 | H | 0.6012856 | 0.6224806 | 0.625108 |
| 27 | V | 0.601303 | 0.6056102 | 0.6216926 |
| 28 | R | 0.53412235 | 0.5690325 | 0.57111305 |
| 29 | E | 0.57521147 | 0.58609 | 0.58857197 |
| 30 | Y | 0.59962344 | 0.6083953 | 0.61979485 |
| 31 | L | 0.580669 | 0.6142875 | 0.6088692 |
| 32 | R | 0.48029393 | 0.5035724 | 0.5258274 |
| 33 | E | 0.5393378 | 0.5415121 | 0.5602201 |
| 34 | F | 0.51731855 | 0.53842914 | 0.5258898 |
| 35 | G | 0.54541487 | 0.54422 | 0.5619371 |
| 36 | L | 0.55193275 | 0.5783587 | 0.557741 |
| 37 | L | 0.5409345 | 0.5594418 | 0.5364478 |
| 38 | R | 0.48052216 | 0.48819166 | 0.48554513 |
| 39 | S | 0.5097862 | 0.53541434 | 0.5311207 |
| 40 | E | 0.46630418 | 0.49978027 | 0.4883483 |
| 41 | E | 0.4626374 | 0.4849224 | 0.46822873 |
| 42 | A | 0.54706144 | 0.57281286 | 0.5695176 |
| 43 | K | 0.54007536 | 0.5650645 | 0.544276 |
| 44 | F | 0.48977306 | 0.51318705 | 0.52970296 |
| 45 | H | 0.5071209 | 0.5371247 | 0.51314485 |
| 46 | Y | 0.5108136 | 0.5499791 | 0.5278967 |
| 47 | D | 0.5110839 | 0.58232063 | 0.5582605 |
| 48 | R | 0.24610925 | 0.52371794 | 0.4834217 |
| 49 | T | 0.55461013 | 0.5773242 | 0.5379382 |
| 50 | R | 0.497995 | 0.50995076 | 0.50501454 |
| 51 | F | 0.5459188 | 0.55578697 | 0.5340841 |
| 52 | G | 0.6148364 | 0.63895506 | 0.60725737 |
| 53 | E | 0.6087729 | 0.6280789 | 0.58560807 |
| 54 | L | 0.6185134 | 0.64911693 | 0.6166868 |
| 55 | V | 0.62733716 | 0.635276 | 0.59659743 |
| 56 | A | 0.6788678 | 0.69479054 | 0.64477044 |
| 57 | R | 0.6112178 | 0.6362824 | 0.56776 |
| 58 | A | 0.6127211 | 0.6157742 | 0.551679 |
| 59 | Y | 0.61127925 | 0.6192591 | 0.5554328 |
| 60 | P | 0.6083041 | 0.634454 | 0.5722721 |
| 61 | F | 0.56254345 | 0.5693072 | 0.54632753 |
| 62 | A | 0.62688696 | 0.6213983 | 0.5730439 |
| 63 | A | 0.644603 | 0.6406537 | 0.61472356 |
| 64 | L | 0.6386281 | 0.6430404 | 0.6199863 |
| 65 | E | 0.5905191 | 0.5893798 | 0.59033066 |
| 66 | E | 0.5987051 | 0.5909012 | 0.5873444 |
| 67 | L | 0.6710999 | 0.67370707 | 0.64263463 |
| 68 | C | 0.6656742 | 0.66478616 | 0.6392205 |
| 69 | V | 0.6601154 | 0.65515476 | 0.6218918 |
| 70 | I | 0.6320303 | 0.6339406 | 0.6017425 |
| 71 | T | 0.6506227 | 0.66776687 | 0.6260485 |
| 72 | D | 0.6727994 | 0.69109255 | 0.6445989 |
| 73 | W | 0.5927772 | 0.6083447 | 0.45627293 |
| 74 | M | 0.5911538 | 0.6131426 | 0.5799013 |
| 75 | A | 0.6230029 | 0.63999486 | 0.61243355 |
| 76 | V | 0.5702507 | 0.586415 | 0.56054145 |
| 77 | W | 0.50580597 | 0.5233032 | 0.38560185 |
| 78 | A | 0.6053377 | 0.6288434 | 0.5978856 |
| 79 | I | 0.5428866 | 0.55449665 | 0.53713775 |
| 80 | F | 0.53929967 | 0.5532259 | 0.5251071 |
| 81 | D | 0.538452 | 0.56894034 | 0.52907544 |
| 82 | D | 0.5335926 | 0.51114166 | 0.5077711 |
| 83 | Y | 0.49400342 | 0.50449306 | 0.4688197 |
| 84 | L | 0.5011503 | 0.52350557 | 0.5018982 |
| 85 | E | 0.40335348 | 0.43634441 | 0.38326645 |
| 86 | R | 0.30952474 | 0.38627973 | 0.30897138 |
| 87 | I | 0.31342554 | 0.39563397 | 0.40079665 |
| 88 | P | 0.34335256 | 0.388844 | 0.31568426 |
| 89 | D | 0.3240241 | 0.38200414 | 0.37376153 |
| 90 | A | 0.22415088 | 0.4119333 | 0.40345013 |
| 91 | Q | 0.23382588 | 0.42178404 | 0.41038218 |
| 92 | D | 0.3594441 | 0.51252437 | 0.47204322 |
| 93 | D | 0.4549796 | 0.556571 | 0.5021957 |
| 94 | E | 0.47148097 | 0.5675436 | 0.5439385 |
| 95 | R | 0.26853392 | 0.41875052 | 0.47347775 |
| 96 | F | 0.44946298 | 0.48718894 | 0.49854323 |
| 97 | V | 0.54338616 | 0.57443017 | 0.56596303 |
| 98 | A | 0.5614846 | 0.5796014 | 0.5757863 |
| 99 | L | 0.4194728 | 0.49458945 | 0.5258721 |
| 100 | I | 0.55073553 | 0.55291605 | 0.5273559 |
| 101 | H | 0.5055611 | 0.5182565 | 0.5133988 |
| 102 | E | 0.54009205 | 0.549921 | 0.5485386 |
| 103 | T | 0.5429534 | 0.5489002 | 0.5475484 |
| 104 | V | 0.5591614 | 0.56483513 | 0.5542039 |
| 105 | S | 0.5642179 | 0.56446415 | 0.5192691 |
| 106 | W | 0.49154392 | 0.4939247 | 0.33108705 |
| 107 | F | 0.52416706 | 0.5242737 | 0.35714442 |
| 108 | P | 0.53778726 | 0.5457421 | 0.34395105 |
| 109 | L | 0.5025998 | 0.49822202 | 0.38971335 |
| 110 | T | 0.3382161 | 0.32244095 | 0.25882575 |
| 111 | P | 0.40712252 | 0.42089564 | 0.21503063 |
| 112 | P | 0.39104158 | 0.42153484 | 0.25474423 |
| 113 | G | 0.37167218 | 0.37254733 | 0.3140145 |
| 114 | A | 0.24668667 | 0.2639883 | 0.26823068 |
| 115 | V | 0.32995054 | 0.37556916 | 0.28549388 |
| 116 | A | 0.2513303 | 0.25644013 | 0.3063194 |
| 117 | R | 0.3377994 | 0.2634476 | 0.32552657 |
| 118 | S | 0.41033617 | 0.39796868 | 0.40565065 |
| 119 | G | 0.402326 | 0.44408098 | 0.4239251 |
| 120 | N | 0.39574632 | 0.47256872 | 0.4683473 |
| 121 | P | 0.5220141 | 0.5482437 | 0.54675674 |
| 122 | I | 0.5212077 | 0.5262727 | 0.53817254 |
| 123 | E | 0.49983978 | 0.5264137 | 0.505041 |
| 124 | L | 0.573235 | 0.5926294 | 0.5750691 |
| 125 | A | 0.6080251 | 0.61584824 | 0.6089279 |
| 126 | I | 0.55209047 | 0.5658609 | 0.55093384 |
| 127 | R | 0.51950914 | 0.5411344 | 0.46253955 |
| 128 | D | 0.5794448 | 0.5800856 | 0.5740789 |
| 129 | I | 0.6265423 | 0.6199075 | 0.59492457 |
| 130 | W | 0.5697112 | 0.5764596 | 0.5345295 |
| 131 | D | 0.6030453 | 0.6081105 | 0.5956091 |
| 132 | R | 0.59486395 | 0.6052106 | 0.5596781 |
| 133 | L | 0.61193925 | 0.6002837 | 0.59807825 |
| 134 | T | 0.61216486 | 0.6043696 | 0.55929524 |
| 135 | A | 0.58351797 | 0.5915745 | 0.57411206 |
| 136 | R | 0.49185434 | 0.5008976 | 0.483365 |
| 137 | S | 0.5965617 | 0.6007469 | 0.58690333 |
| 138 | S | 0.6080765 | 0.6287766 | 0.5961235 |
| 139 | L | 0.63133866 | 0.6281387 | 0.5958797 |
| 140 | T | 0.6131952 | 0.6367401 | 0.58722734 |
| 141 | W | 0.6065312 | 0.61202705 | 0.5741623 |
| 142 | R | 0.62830096 | 0.61993843 | 0.5198738 |
| 143 | R | 0.572299 | 0.5816585 | 0.55706805 |
| 144 | R | 0.5947338 | 0.6184162 | 0.5837843 |
| 145 | F | 0.6313574 | 0.6402451 | 0.59687257 |
| 146 | V | 0.6429617 | 0.6515211 | 0.62878686 |
| 147 | R | 0.5378459 | 0.5317808 | 0.53728604 |
| 148 | H | 0.5782135 | 0.6112718 | 0.5637835 |
| 149 | L | 0.62521154 | 0.6328991 | 0.5990634 |
| 150 | T | 0.6308168 | 0.63651633 | 0.610023 |
| 151 | D | 0.6143579 | 0.6336368 | 0.6000297 |
| 152 | Y | 0.5931263 | 0.6296536 | 0.58209693 |
| 153 | L | 0.6178302 | 0.6343823 | 0.6088492 |
| 154 | E | 0.58732814 | 0.6027445 | 0.5829233 |
| 155 | G | 0.5966928 | 0.6202063 | 0.58267474 |
| 156 | C | 0.59717166 | 0.6039495 | 0.5253677 |
| 157 | H | 0.55696446 | 0.5873476 | 0.5401051 |
| 158 | W | 0.5763864 | 0.5941234 | 0.539268 |
| 159 | E | 0.62790745 | 0.6388401 | 0.5984739 |
| 160 | S | 0.560738 | 0.5891857 | 0.57112765 |
| 161 | H | 0.53771156 | 0.56068754 | 0.5337987 |
| 162 | N | 0.58668095 | 0.5889853 | 0.5672062 |
| 163 | R | 0.52594554 | 0.53263205 | 0.52669084 |
| 164 | R | 0.4797576 | 0.5277494 | 0.49451873 |
| 165 | R | 0.5131964 | 0.52907586 | 0.50301164 |
| 166 | G | 0.5844965 | 0.60170305 | 0.5560352 |
| 167 | I | 0.58256555 | 0.5888248 | 0.5875223 |
| 168 | A | 0.62241095 | 0.6318216 | 0.6365075 |
| 169 | P | 0.5985109 | 0.60294306 | 0.61369985 |
| 170 | D | 0.6150434 | 0.634402 | 0.6167689 |
| 171 | L | 0.6173232 | 0.63764817 | 0.6179734 |
| 172 | P | 0.62560004 | 0.63936734 | 0.61389506 |
| 173 | T | 0.6142733 | 0.6337077 | 0.6134188 |
| 174 | Y | 0.64607114 | 0.6759931 | 0.60759276 |
| 175 | I | 0.6041932 | 0.62170213 | 0.61080134 |
| 176 | R | 0.5216145 | 0.5101888 | 0.52695656 |
| 177 | T | 0.5814685 | 0.6254501 | 0.5941879 |
| 178 | R | 0.5639028 | 0.610617 | 0.5899641 |
| 179 | R | 0.510059 | 0.52793527 | 0.53733975 |
| 180 | R | 0.46830693 | 0.48950362 | 0.47282225 |
| 181 | F | 0.49470693 | 0.5192977 | 0.39893207 |
| 182 | G | 0.6045018 | 0.63806134 | 0.5640151 |
| 183 | G | 0.5594319 | 0.5554666 | 0.54847777 |
| 184 | M | 0.51042265 | 0.5530826 | 0.49664676 |
| 185 | R | 0.51600295 | 0.5243521 | 0.52533823 |
| 186 | P | 0.60715127 | 0.634298 | 0.568538 |
| 187 | S | 0.57904714 | 0.58255637 | 0.5885927 |
| 188 | M | 0.5891929 | 0.5974538 | 0.58968115 |
| 189 | D | 0.5958258 | 0.6101327 | 0.57193196 |
| 190 | L | 0.6244105 | 0.6205716 | 0.5925248 |
| 191 | S | 0.60655147 | 0.6061499 | 0.5556445 |
| 192 | E | 0.6316424 | 0.6516029 | 0.6028019 |
| 193 | I | 0.600407 | 0.6085469 | 0.5697722 |
| 194 | G | 0.5883924 | 0.596867 | 0.5286305 |
| 195 | L | 0.607186 | 0.6180392 | 0.57248646 |
| 196 | G | 0.579949 | 0.6026552 | 0.5548835 |
| 197 | I | 0.5771276 | 0.58832765 | 0.4316141 |
| 198 | E | 0.5966416 | 0.6368372 | 0.57068 |
| 199 | L | 0.57322323 | 0.6065684 | 0.5499769 |
| 200 | T | 0.57110316 | 0.6238946 | 0.5459826 |
| 201 | D | 0.49697712 | 0.55255145 | 0.44688058 |
| 202 | D | 0.5637278 | 0.57856935 | 0.5316156 |
| 203 | V | 0.6090274 | 0.61670387 | 0.5016591 |
| 204 | H | 0.5766172 | 0.5823402 | 0.54313314 |
| 205 | A | 0.62869114 | 0.6107547 | 0.5997193 |
| 206 | H | 0.602898 | 0.58801335 | 0.5925706 |
| 207 | P | 0.61780673 | 0.63152784 | 0.651767 |
| 208 | R | 0.57934326 | 0.59593725 | 0.59981066 |
| 209 | I | 0.6456012 | 0.6546813 | 0.6359608 |
| 210 | Q | 0.6390804 | 0.6579631 | 0.63055754 |
| 211 | Q | 0.6029169 | 0.6079805 | 0.6228717 |
| 212 | L | 0.7026865 | 0.7125187 | 0.68077374 |
| 213 | L | 0.66344905 | 0.6664537 | 0.64173466 |
| 214 | D | 0.6316456 | 0.6415351 | 0.6218282 |
| 215 | N | 0.6435375 | 0.6543237 | 0.60224396 |
| 216 | T | 0.6569389 | 0.6665815 | 0.6228078 |
| 217 | A | 0.6544719 | 0.6735244 | 0.6300839 |
| 218 | D | 0.64341724 | 0.67691535 | 0.62849087 |
| 219 | L | 0.6681042 | 0.6823655 | 0.58596176 |
| 220 | V | 0.6673107 | 0.681173 | 0.60269403 |
| 221 | L | 0.62534636 | 0.6478938 | 0.59528345 |
| 222 | W | 0.5891322 | 0.58659464 | 0.57009584 |
| 223 | A | 0.65116984 | 0.6575753 | 0.6048926 |
| 224 | N | 0.616418 | 0.61921775 | 0.6103426 |
| 225 | D | 0.6816183 | 0.70461243 | 0.63228303 |
| 226 | V | 0.66347563 | 0.67603767 | 0.6408515 |
| 227 | F | 0.5644633 | 0.57237273 | 0.52449375 |
| 228 | S | 0.63312054 | 0.6438238 | 0.5639897 |
| 229 | V | 0.6358618 | 0.6415913 | 0.60156745 |
| 230 | E | 0.53011215 | 0.5140616 | 0.5010784 |
| 231 | A | 0.57749885 | 0.6117387 | 0.46537176 |
| 232 | E | 0.60051405 | 0.6099937 | 0.5559244 |
| 233 | K | 0.5545263 | 0.53762525 | 0.49166435 |
| 234 | R | 0.4788651 | 0.49717364 | 0.45759895 |
| 235 | E | 0.4935273 | 0.47487113 | 0.47129425 |
| 236 | G | 0.47080877 | 0.4909599 | 0.4573051 |
| 237 | N | 0.59475213 | 0.603001 | 0.55249226 |
| 238 | V | 0.6345803 | 0.6440326 | 0.5934198 |
| 239 | N | 0.6333075 | 0.63332903 | 0.51829624 |
| 240 | N | 0.66482586 | 0.65162504 | 0.60840917 |
| 241 | I | 0.6615003 | 0.6615954 | 0.6330613 |
| 242 | V | 0.7097858 | 0.7086747 | 0.69671404 |
| 243 | L | 0.69184786 | 0.68113184 | 0.66008824 |
| 244 | V | 0.6771692 | 0.67280084 | 0.6719597 |
| 245 | V | 0.6777918 | 0.66523224 | 0.66229045 |
| 246 | Q | 0.615864 | 0.61858284 | 0.5942249 |
| 247 | R | 0.55556285 | 0.5524926 | 0.55807 |
| 248 | T | 0.61783916 | 0.6087769 | 0.59444237 |
| 249 | R | 0.52431196 | 0.5550667 | 0.506352 |
| 250 | G | 0.6010877 | 0.6249471 | 0.5879974 |
| 251 | G | 0.57490736 | 0.61967677 | 0.53512776 |
| 252 | S | 0.66464555 | 0.6970398 | 0.6249058 |
| 253 | M | 0.6077913 | 0.63139284 | 0.61160153 |
| 254 | R | 0.5305722 | 0.58858675 | 0.5311204 |
| 255 | E | 0.6611168 | 0.68541116 | 0.6479848 |
| 256 | A | 0.71341157 | 0.72956264 | 0.7101015 |
| 257 | A | 0.69207084 | 0.70663357 | 0.655008 |
| 258 | D | 0.64617205 | 0.6879811 | 0.6307992 |
| 259 | E | 0.65448827 | 0.668344 | 0.655837 |
| 260 | V | 0.6556695 | 0.65977234 | 0.64792484 |
| 261 | A | 0.641115 | 0.66193634 | 0.632874 |
| 262 | A | 0.6565632 | 0.6754769 | 0.6475613 |
| 263 | M | 0.6132702 | 0.60726976 | 0.60622525 |
| 264 | L | 0.6315812 | 0.646971 | 0.58090675 |
| 265 | R | 0.52036476 | 0.539604 | 0.47363526 |
| 266 | G | 0.59492844 | 0.6027366 | 0.5521388 |
| 267 | R | 0.56374025 | 0.5873532 | 0.5206984 |
| 268 | C | 0.64188874 | 0.64776254 | 0.518224 |
| 269 | A | 0.6689942 | 0.6708594 | 0.58577645 |
| 270 | D | 0.64841074 | 0.6494557 | 0.58710647 |
| 271 | F | 0.6755141 | 0.6905322 | 0.5838254 |
| 272 | V | 0.65551114 | 0.66010195 | 0.5272511 |
| 273 | A | 0.669492 | 0.6690105 | 0.6007251 |
| 274 | A | 0.6861954 | 0.6909092 | 0.6446503 |
| 275 | S | 0.68058383 | 0.67770135 | 0.63191605 |
| 276 | R | 0.57904553 | 0.5859548 | 0.5487471 |
| 277 | S | 0.6629853 | 0.6569885 | 0.65695703 |
| 278 | A | 0.6726235 | 0.6759136 | 0.66630745 |
| 279 | V | 0.6489418 | 0.6522785 | 0.6306502 |
| 280 | A | 0.5959728 | 0.6035701 | 0.5740799 |
| 281 | F | 0.57425123 | 0.5662209 | 0.5125232 |
| 282 | F | 0.6239611 | 0.6330808 | 0.57105017 |
| 283 | A | 0.56244004 | 0.56681156 | 0.5501458 |
| 284 | S | 0.5580035 | 0.56593674 | 0.5285994 |
| 285 | S | 0.5360984 | 0.5317374 | 0.4951296 |
| 286 | G | 0.5803464 | 0.58622557 | 0.4871739 |
| 287 | G | 0.6185227 | 0.6329971 | 0.5267564 |
| 288 | Y | 0.532658 | 0.55632436 | 0.32696688 |
| 289 | T | 0.5662417 | 0.6031383 | 0.528543 |
| 290 | A | 0.5827034 | 0.61046 | 0.5722975 |
| 291 | E | 0.55202574 | 0.5703717 | 0.5381701 |
| 292 | Q | 0.6007551 | 0.6374329 | 0.509624 |
| 293 | A | 0.656293 | 0.6648069 | 0.636285 |
| 294 | H | 0.58010167 | 0.59858644 | 0.5670184 |
| 295 | Q | 0.5663435 | 0.5835035 | 0.5447847 |
| 296 | V | 0.64394504 | 0.6568172 | 0.60518783 |
| 297 | S | 0.6439146 | 0.6538674 | 0.623976 |
| 298 | R | 0.56684184 | 0.5919418 | 0.5399826 |
| 299 | Y | 0.5797932 | 0.5953706 | 0.5634928 |
| 300 | I | 0.6421193 | 0.6356873 | 0.5888525 |
| 301 | A | 0.64483297 | 0.64631546 | 0.6076159 |
| 302 | A | 0.62852716 | 0.626446 | 0.5847573 |
| 303 | M | 0.6519744 | 0.6552236 | 0.5934249 |
| 304 | E | 0.65772665 | 0.6584257 | 0.6099781 |
| 305 | S | 0.64146495 | 0.6374513 | 0.58809406 |
| 306 | W | 0.5887849 | 0.6010321 | 0.455381 |
| 307 | V | 0.6960574 | 0.6951989 | 0.6161261 |
| 308 | R | 0.5869824 | 0.58550155 | 0.12294774 |
| 309 | G | 0.64111316 | 0.64912665 | 0.5716963 |
| 310 | N | 0.60098803 | 0.60896873 | 0.56975603 |
| 311 | I | 0.6058516 | 0.61127025 | 0.5402859 |
| 312 | D | 0.5986585 | 0.61012733 | 0.5162502 |
| 313 | W | 0.5572185 | 0.57557046 | 0.49786422 |
| 314 | S | 0.59078634 | 0.603688 | 0.5715384 |
| 315 | R | 0.5005216 | 0.51225173 | 0.4350767 |
| 316 | G | 0.5850333 | 0.62738717 | 0.5587749 |
| 317 | N | 0.5274916 | 0.55629176 | 0.4612568 |
| 318 | E | 0.2724258 | 0.522411 | 0.43992326 |
| 319 | R | 0.42845017 | 0.43670997 | 0.3528106 |
| 320 | Y | 0.49985757 | 0.5514985 | 0.37760812 |
| 321 | R | 0.3354725 | 0.3672154 | 0.36602455 |
| 322 | S | 0.35561132 | 0.38718134 | 0.35999012 |
| 323 | E | 0.28153774 | 0.33582488 | 0.27723527 |
| 324 | H | 0.22330411 | 0.27679995 | 0.23552002 |
| 325 | L | 0.20629914 | 0.24119285 | 0.25275382 |
| 326 | R | 0.1746536 | 0.24691907 | 0.21553385 |
| 327 | T | 0.20177616 | 0.27010584 | 0.24844858 |
| 328 | G | 0.29595792 | 0.3410509 | 0.28713375 |
| 329 | E | 0.21526448 | 0.26737887 | 0.21613127 |
| 330 | D | 0.2277927 | 0.2850415 | 0.21456538 |
| 331 | Q | 0.20005666 | 0.23222525 | 0.18127699 |
| 332 | P | 0.23382793 | 0.24694793 | 0.14178282 |
| 333 | N | 0.22948933 | 0.34376267 | 0.20401303 |
| 334 | F | 0.19149718 | 0.31196773 | 0.060191132 |
| 335 | L | 0.21066952 | 0.2692168 | 0.031149033 |
| 336 | E | 0.22695065 | 0.39976564 | 0.21805996 |
| 337 | R | 0.20472698 | 0.35837045 | 0.05587231 |
| 338 | A | 0.2215744 | 0.31336612 | 0.02904718 |
| 339 | G | 0.2363409 | 0.38292766 | 0.046624474 |
| 340 | Q | 0.16825615 | 0.35265827 | 0.15488286 |
| 341 | P | 0.16575511 | 0.27428117 | 0.17771174 |
